# Supplementary material for: TopBP1 biomolecular condensates as a new therapeutic target in advanced-stage colorectal cancer
Source: eLife. 2025 Oct 21;14:RP106196. doi: 10.7554/eLife.106196 (PMC12539802; doi:10.7554/eLife.106196)
Supplement: Figure 2—source data 1. [file elife-106196-fig2-data1.zip › Fig 2B, D and E- Source Data 1/Fig2B -Source Data 1.pdf]

Figure 2.B

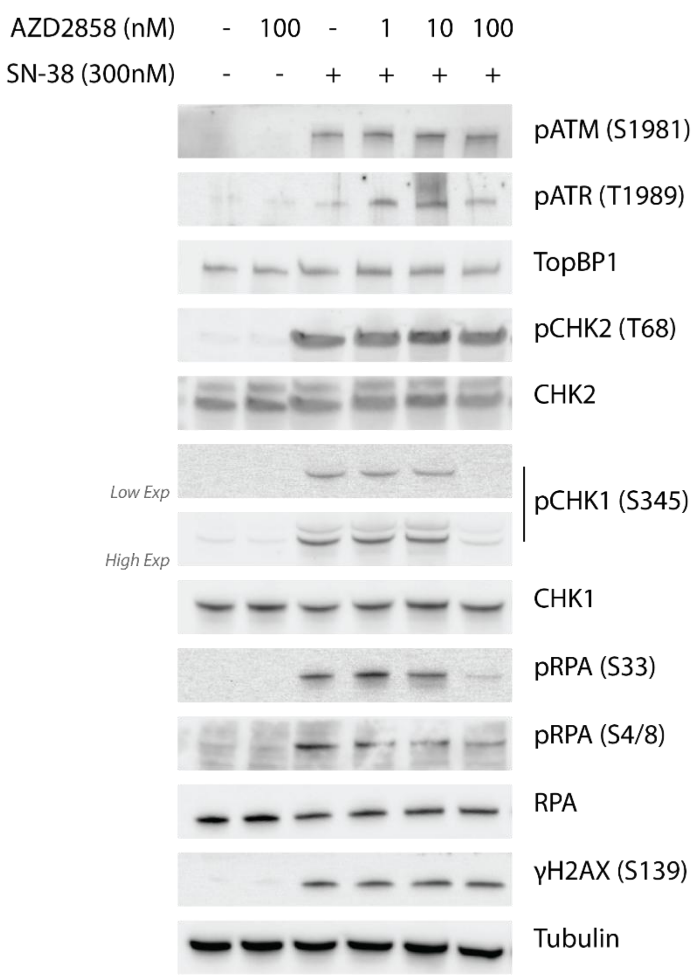

Figure 2B, Source Data 1. Below are the Original membranes corresponding to Figure 2B. Immunoblot of the indicated proteins after incubation of HCT116 cells with AZD2858 at the indicated concentrations and/or SN-38 (300 nM) for 2 h. The experiment was replicated 3 times, and a representative replicate is shown.

Merge chemiluminescence bands/colorimetric for CHK1, CHK2

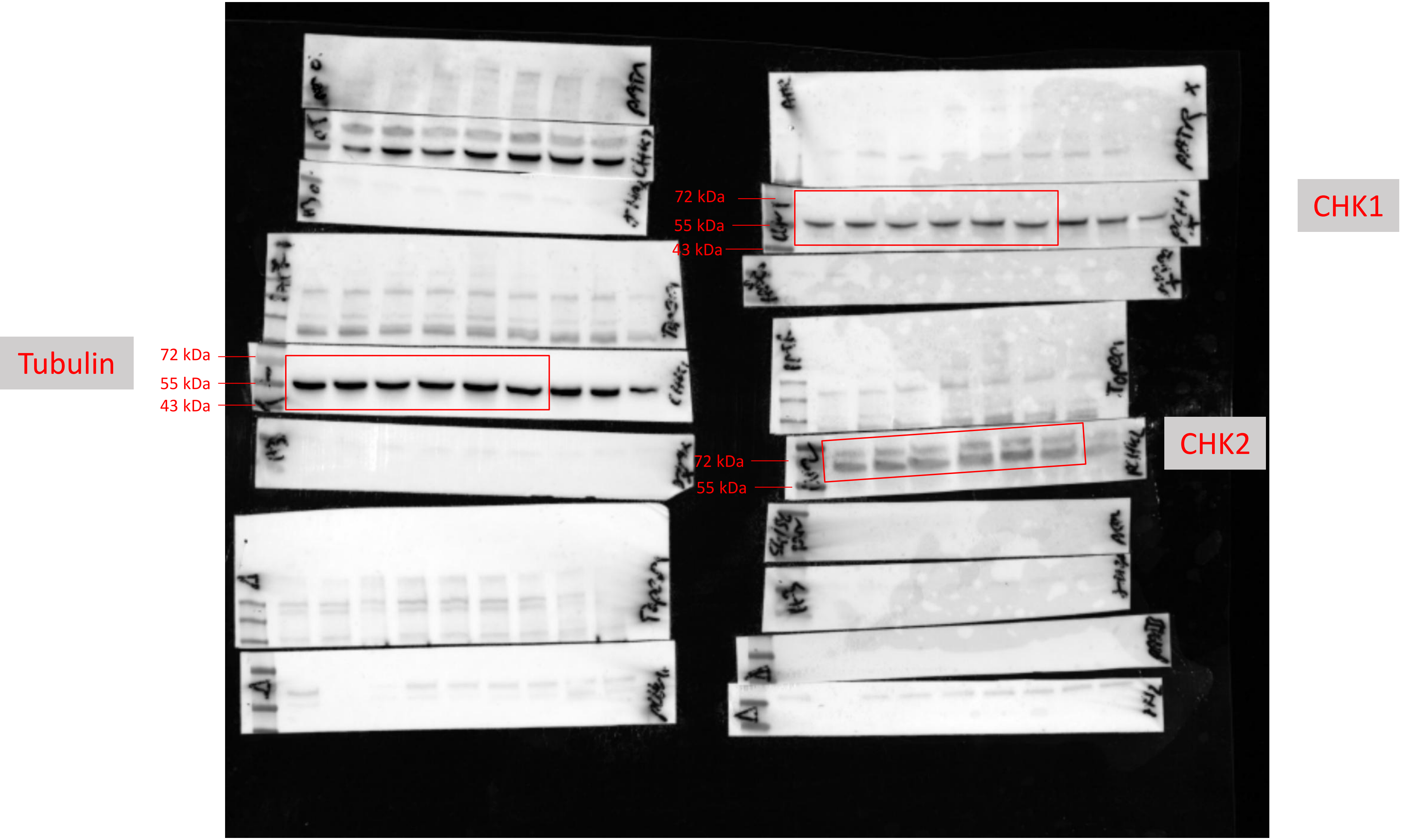

Chemiluminescence bands for CHK1 & CHK2

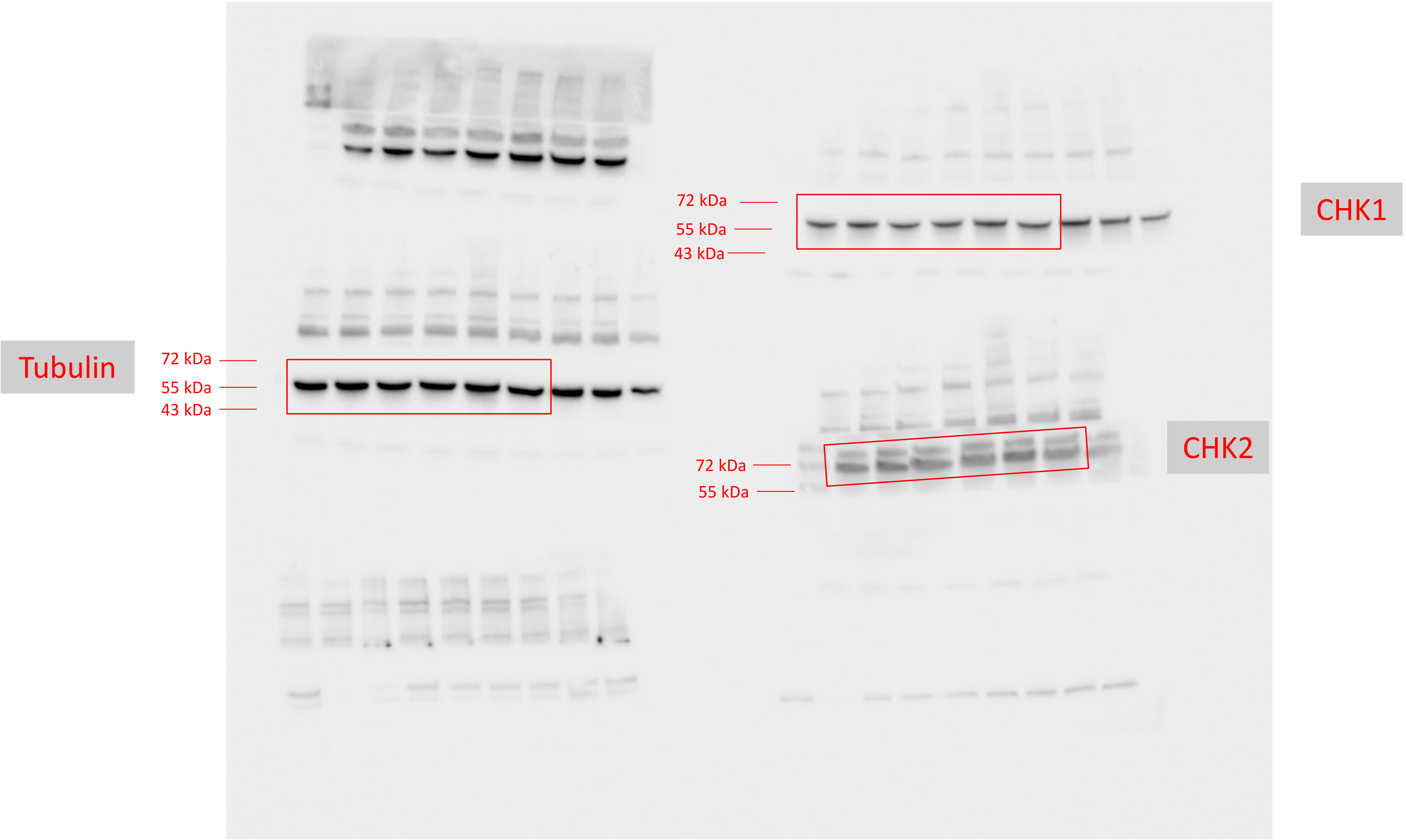

Colorimetric for CHK1, CHK2

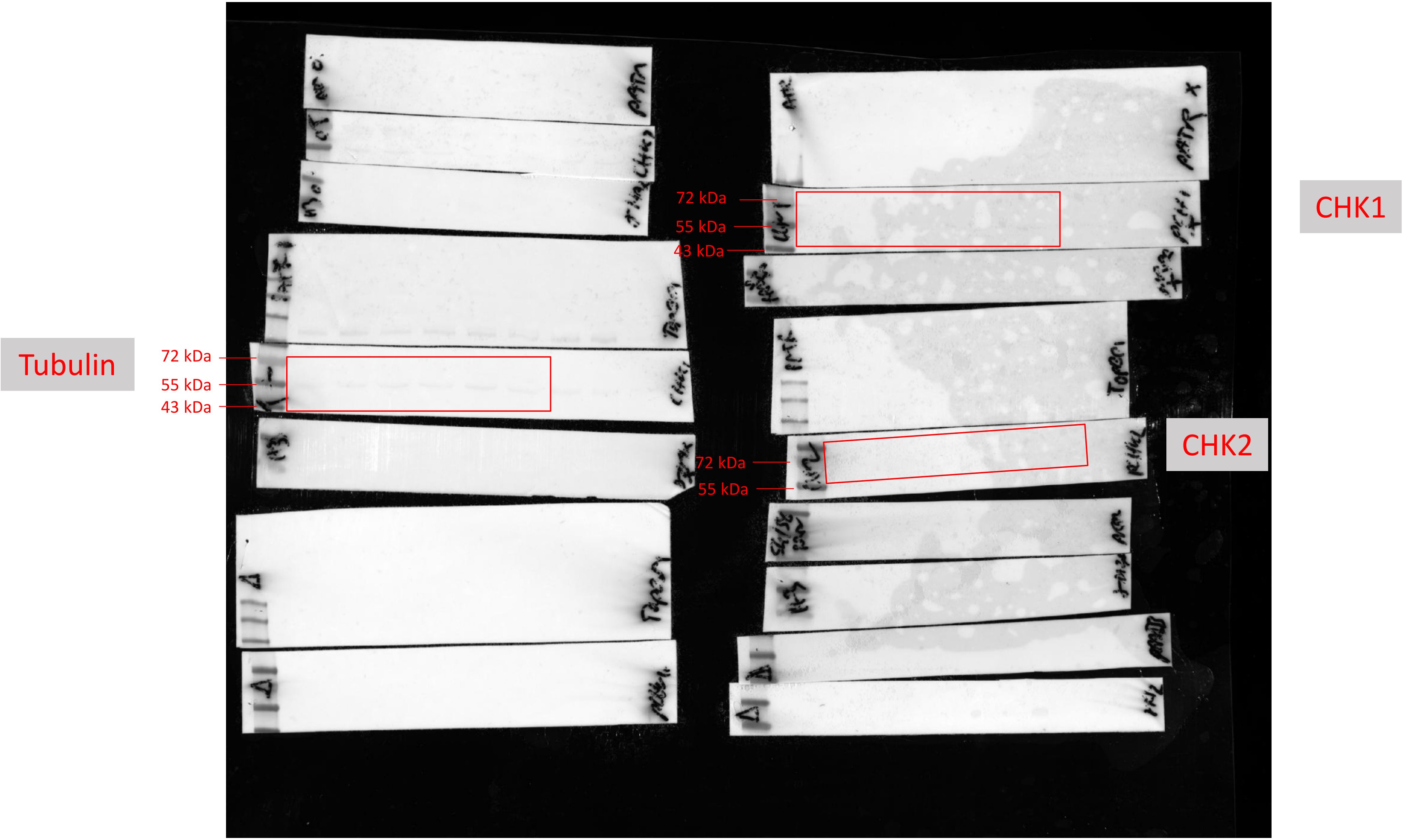

Merge chemiluminescence bands/colorimetric for pATM, TopBP1, RPA

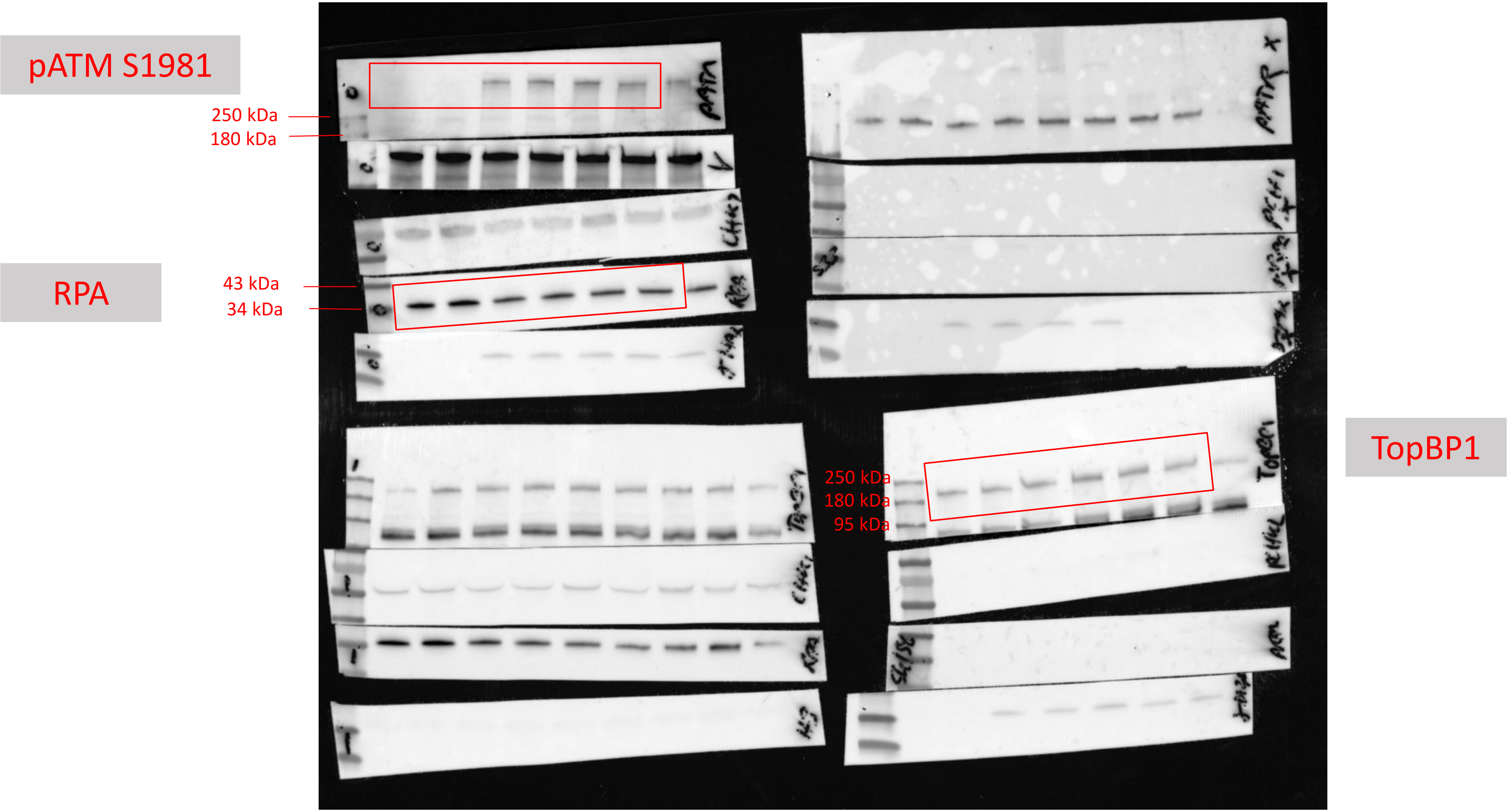

Chemiluminescence bands for pATM, TopBP1, RPA

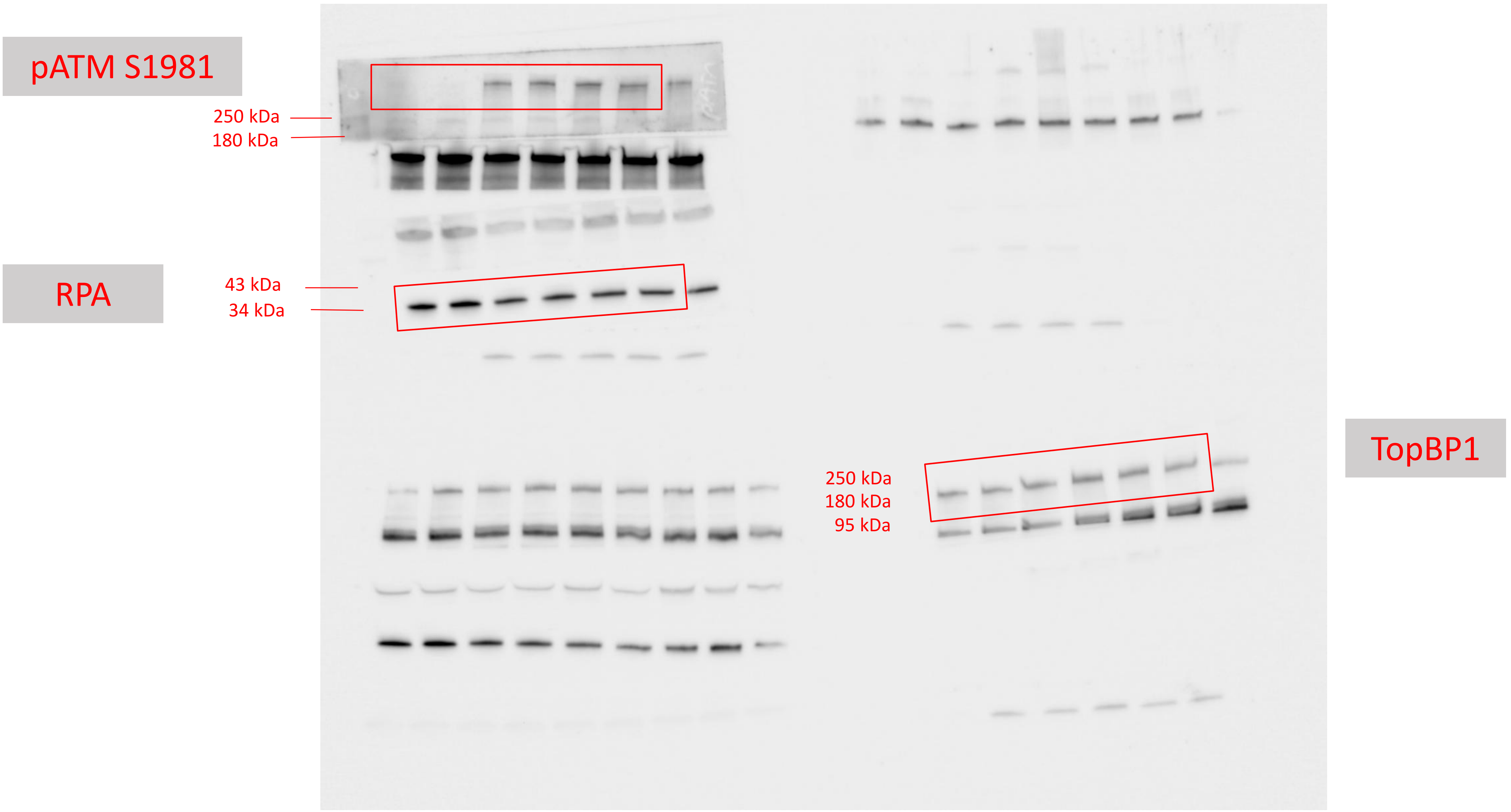

Colorimetric for pATM, TopBP1, RPA

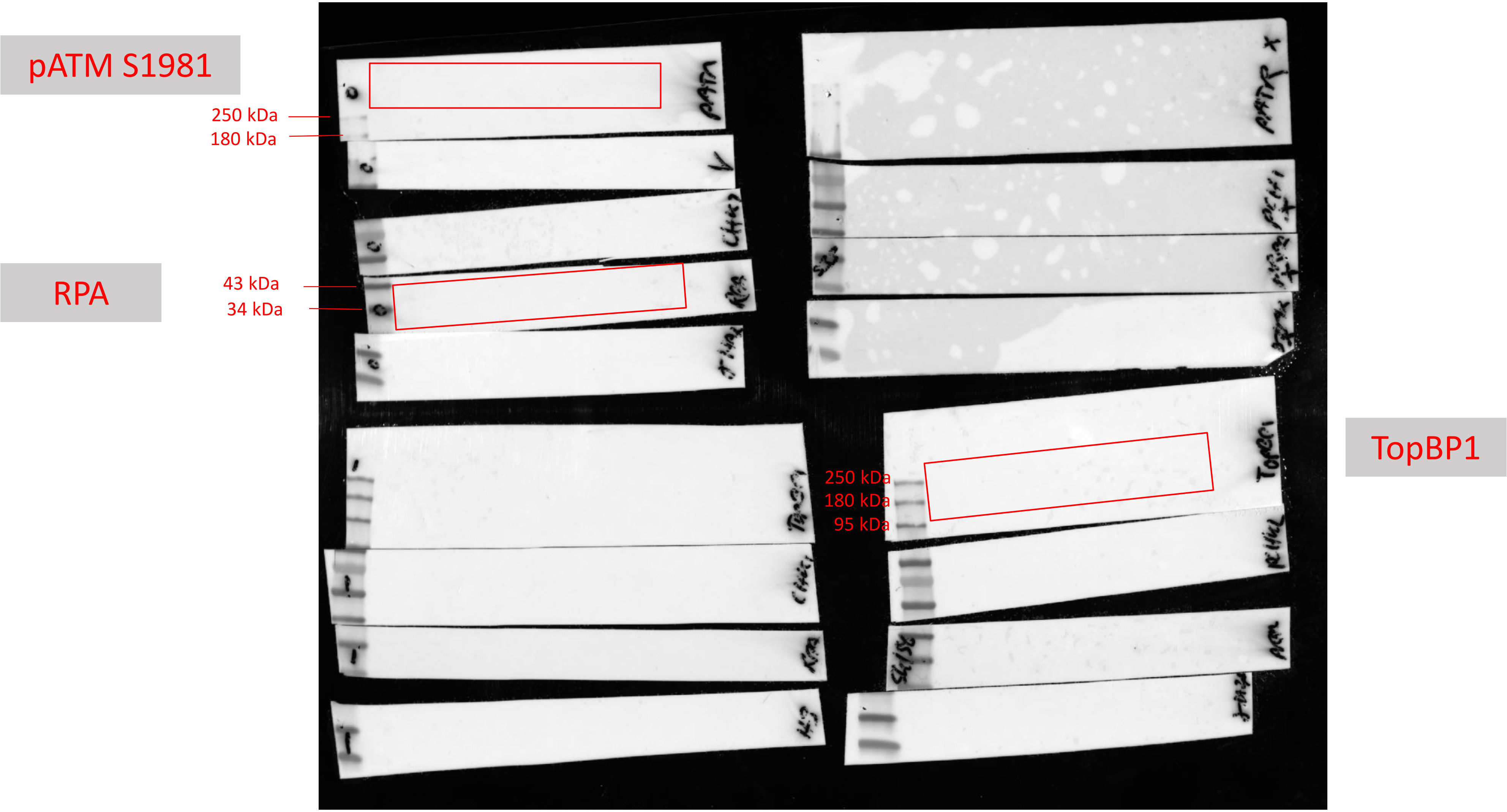

Merge chemiluminescence bands/colorimetric for pATR & γH2AX

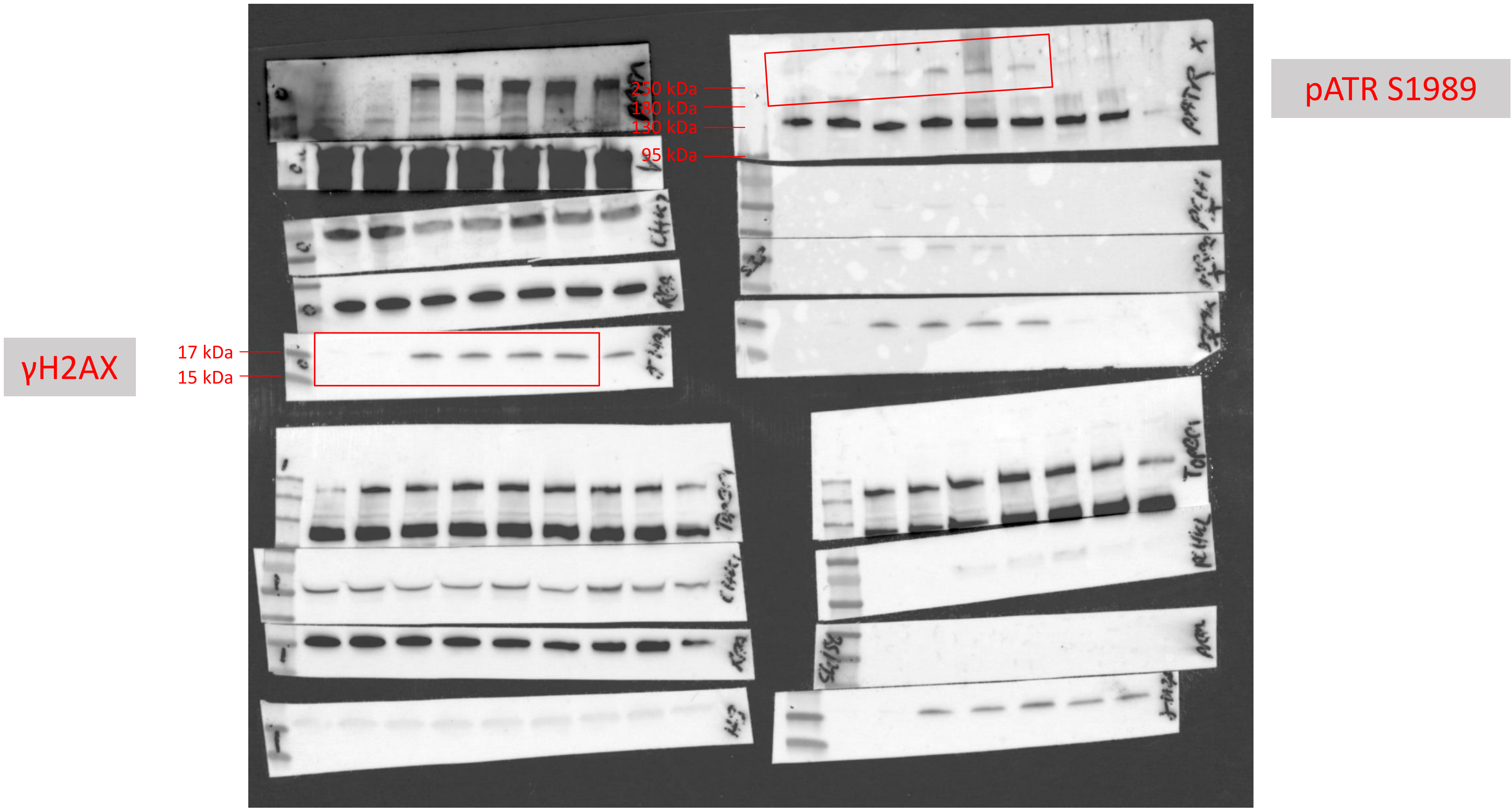

Chemiluminescence bands for pATR & γH2AX

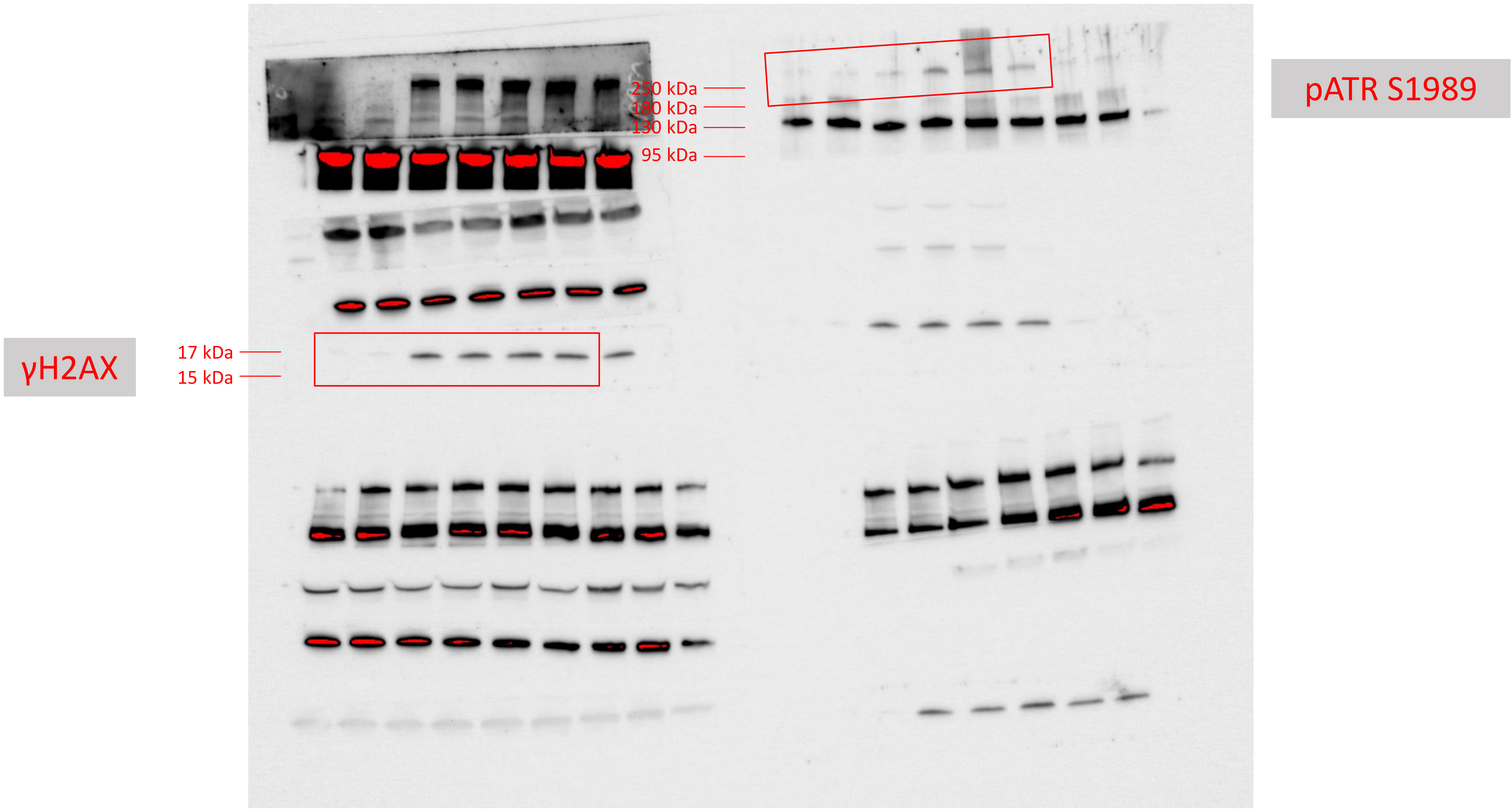

Colorimetric for pATR & γH2AX

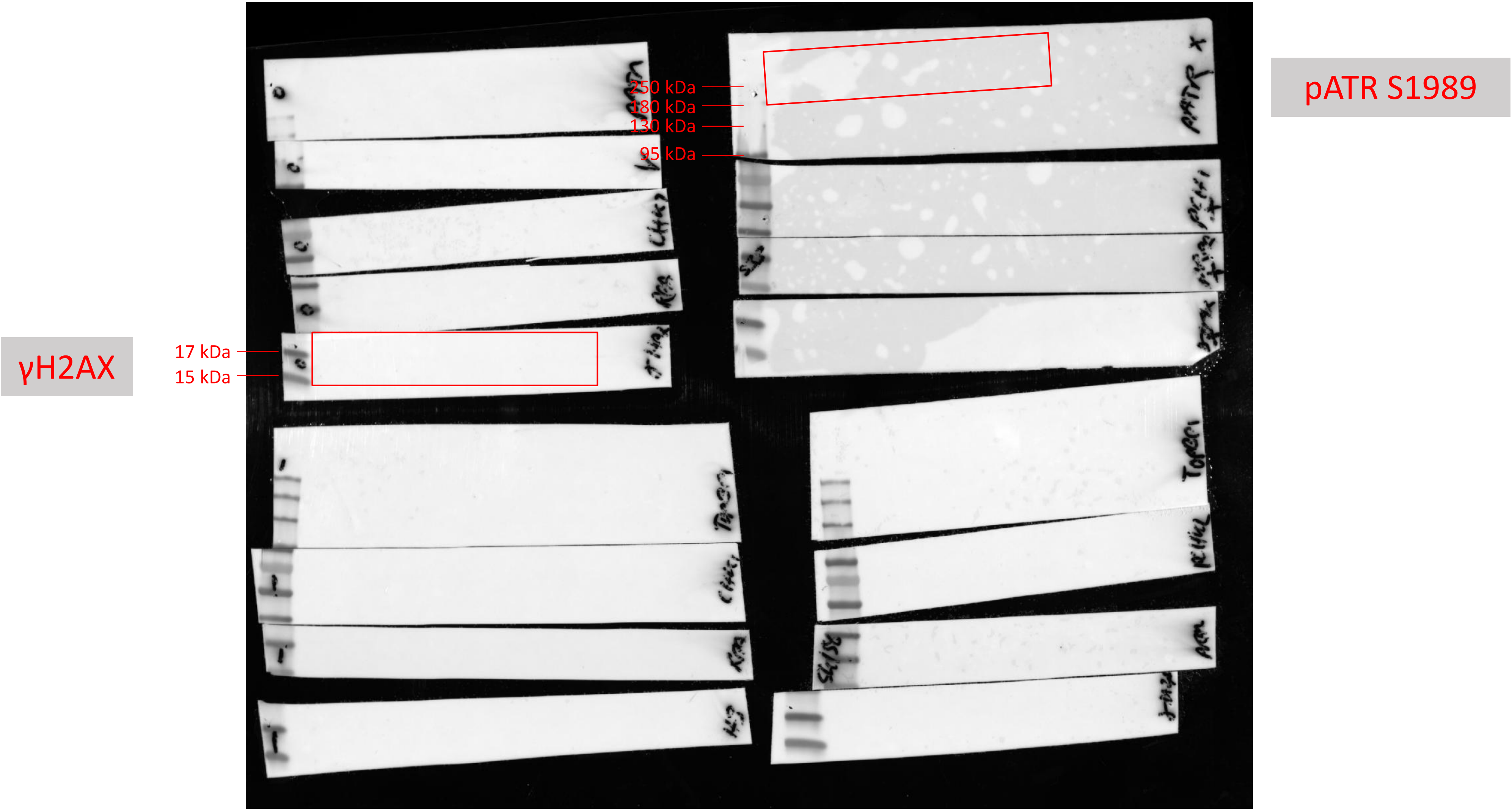

Merge chemiluminescence bands/colorimetric for pRPA32 S4/8, pCHK1 S345, pCHK2 T68

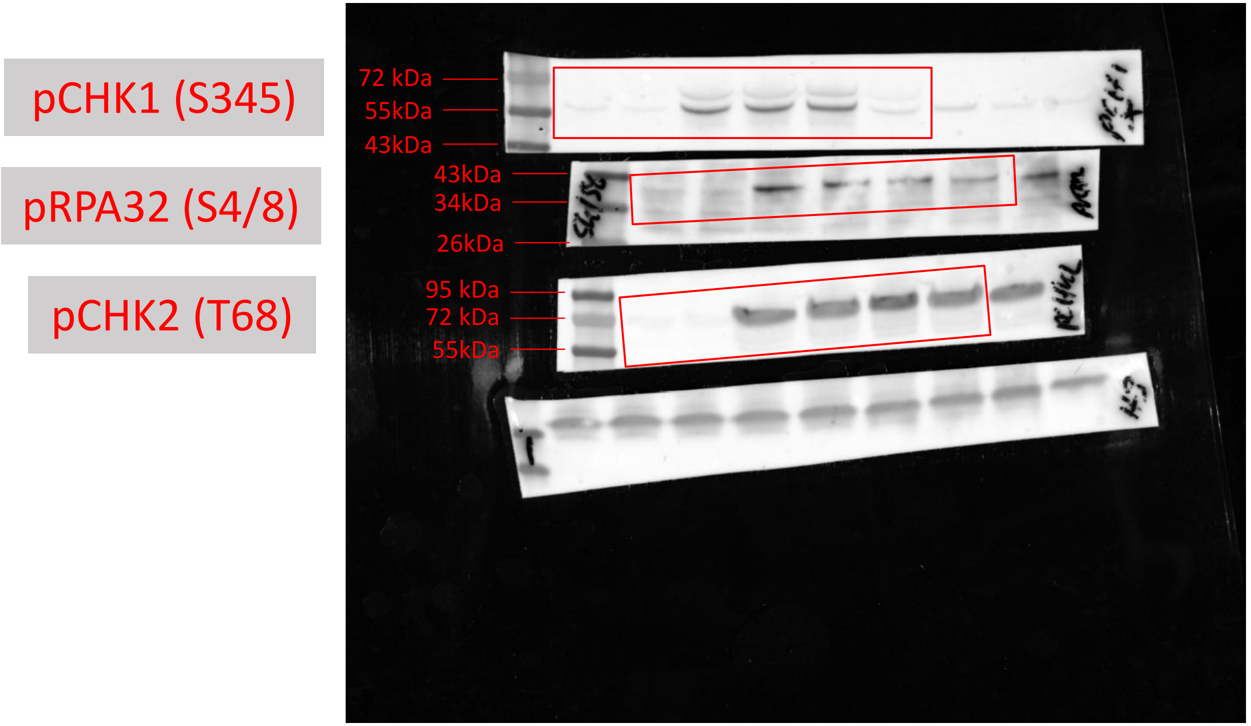

Chemiluminescence bands for pRPA32 S4/8, pCHK1 S345, pCHK2 T68

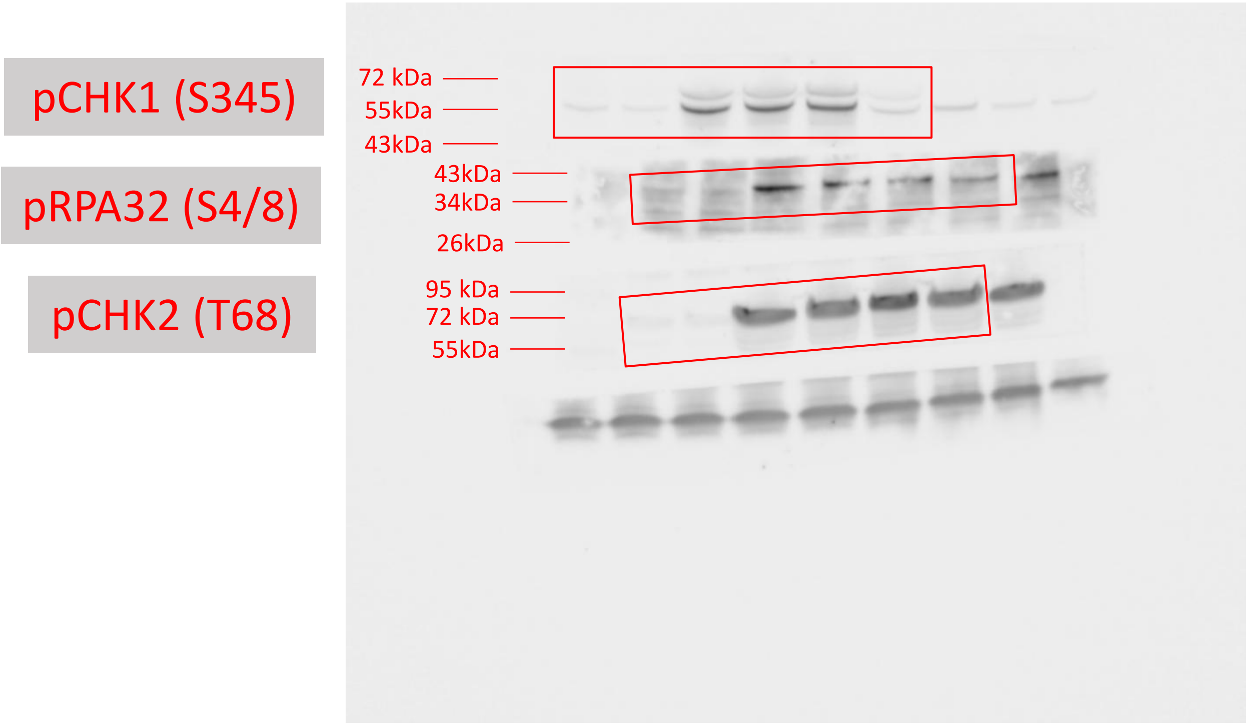

Colorimetric for pRPA32 S4/8, pCHK1 S345, pCHK2 T68

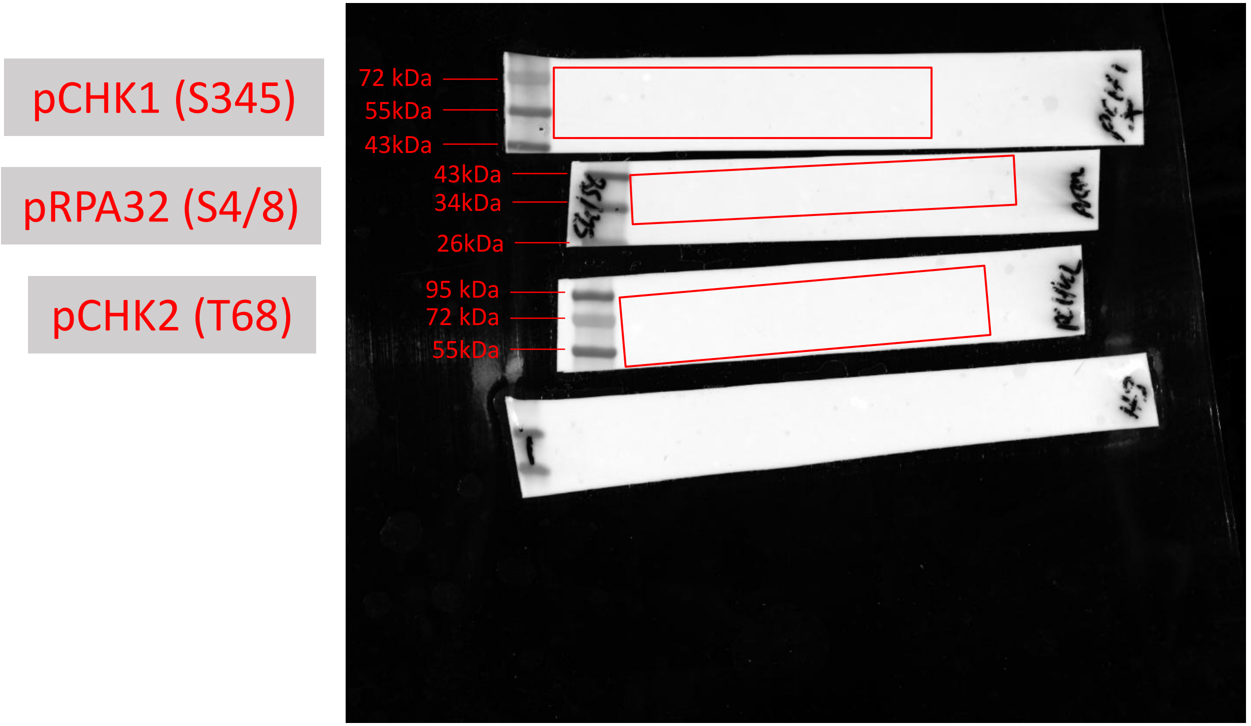

Colorimetric for pRPA32 S33, pCHK1 S345 low exposure

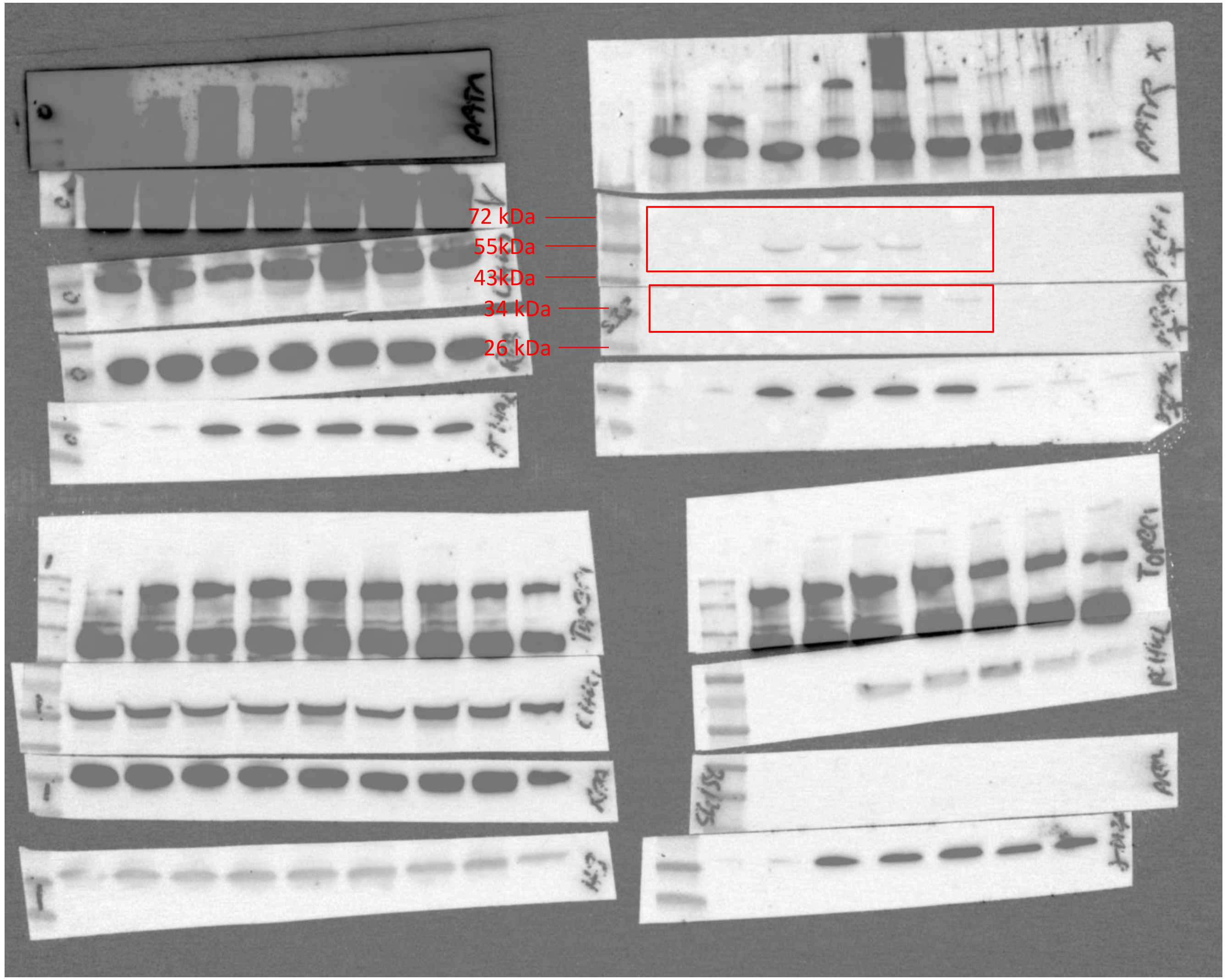

pCHK1 (S345)

pRPA32 (S33)

Chemiluminescence bands for pRPA32 S33, pCHK1 S345 low exposure

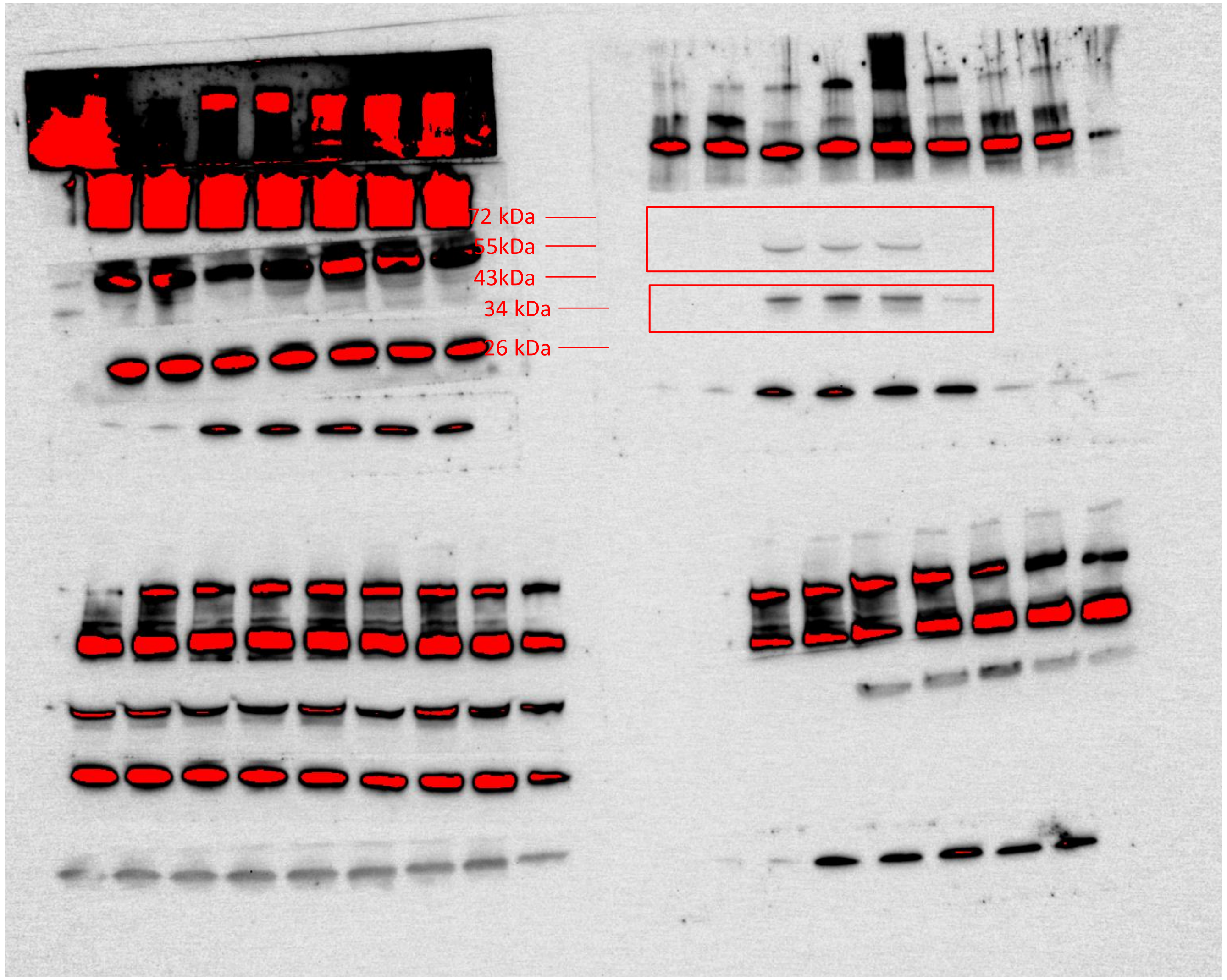

pCHK1 (S345)

pRPA32 (S33)

Colorimetric for pRPA32 S33, pCHK1 S345 low exposure

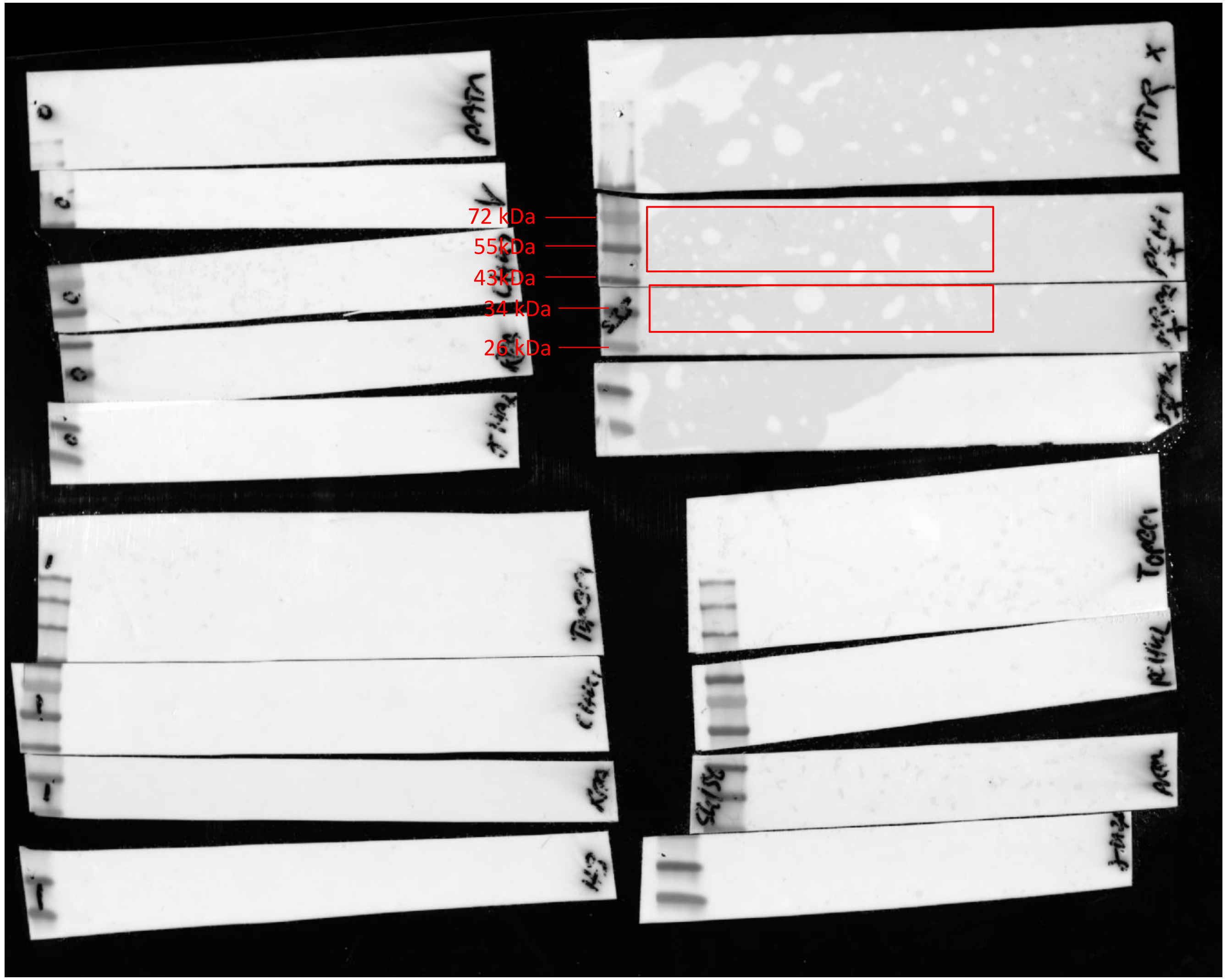

pCHK1 (S345)

pRPA32 (S33)
