# Supplementary material for: TopBP1 biomolecular condensates as a new therapeutic target in advanced-stage colorectal cancer
Source: eLife. 2025 Oct 21;14:RP106196. doi: 10.7554/eLife.106196 (PMC12539802; doi:10.7554/eLife.106196)
Supplement: Figure 2—source data 1. [file elife-106196-fig2-data1.zip › Fig 2B, D and E- Source Data 1/Fig2D -Source Data 1.pdf]

Figure 2.D

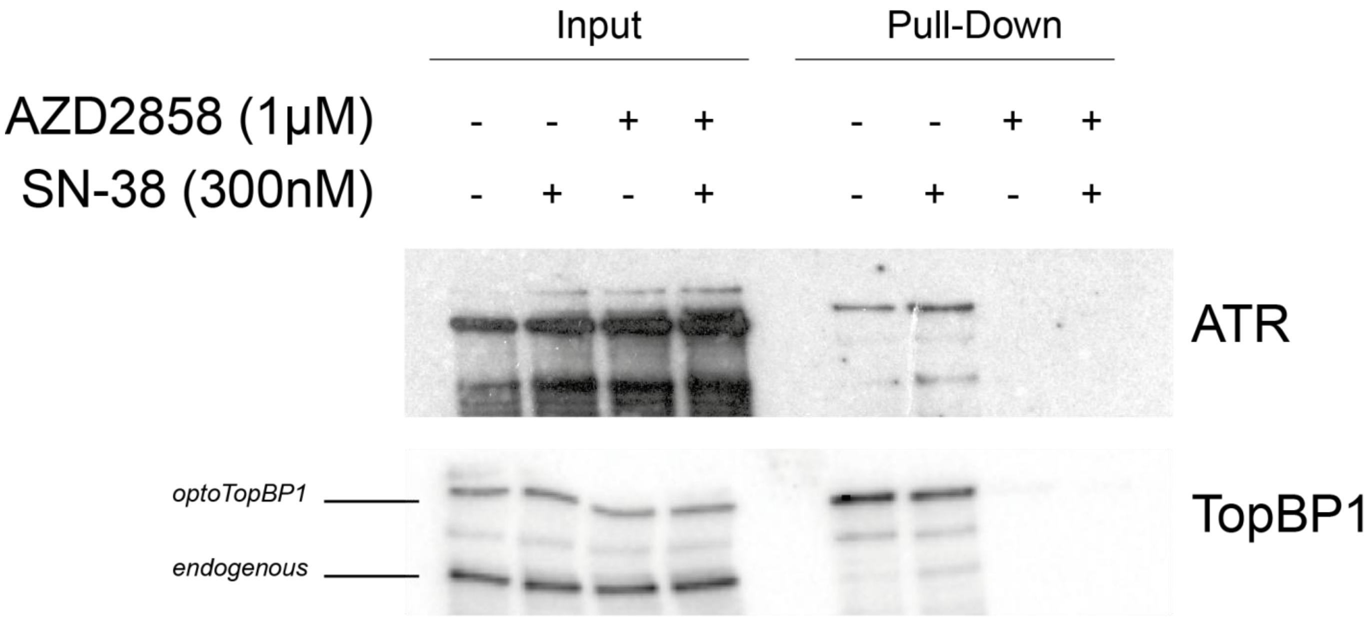

Figure 2D, Source Data 1. Below are the Original membranes corresponding to Figure 2D.
Immunoblotting of TopBP1 and ATR isolated with streptavidin beads from optoTopBP1-expressing cells incubated with doxycycline for 16 h to induce optoTopBP1 expression, and also with AZD2858 (1 µM) and/or SN-38 (300 nM) for the last 2 h. Biotin was added to the medium in all conditions for the last 30 min. Bands that correspond to endogenous and optoTopBP1 proteins are indicated with one and two stars (* and **), respectively

Chemiluminescence bands for ATR

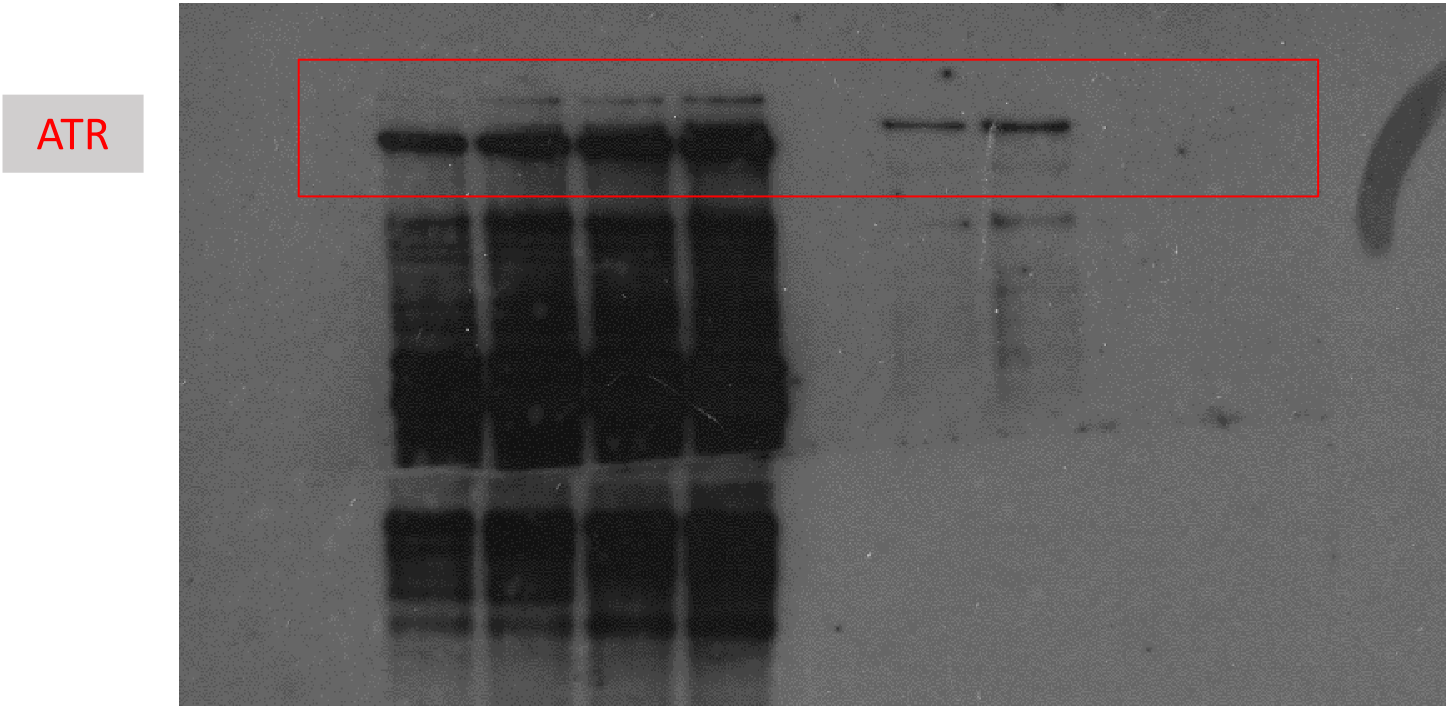

Colorimetric for TopBP1

TopBP1

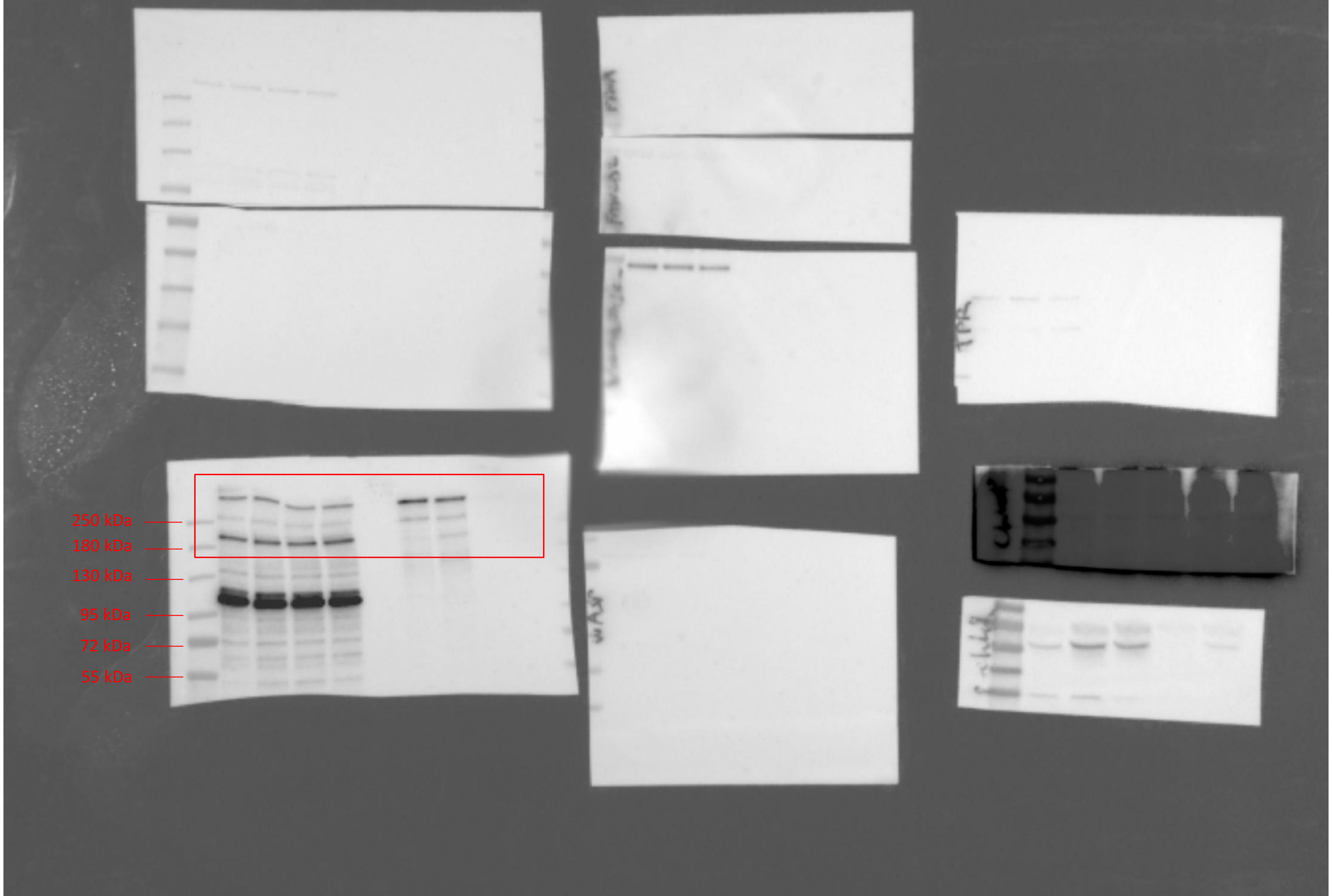

Chemiluminescence bands for TopBP1

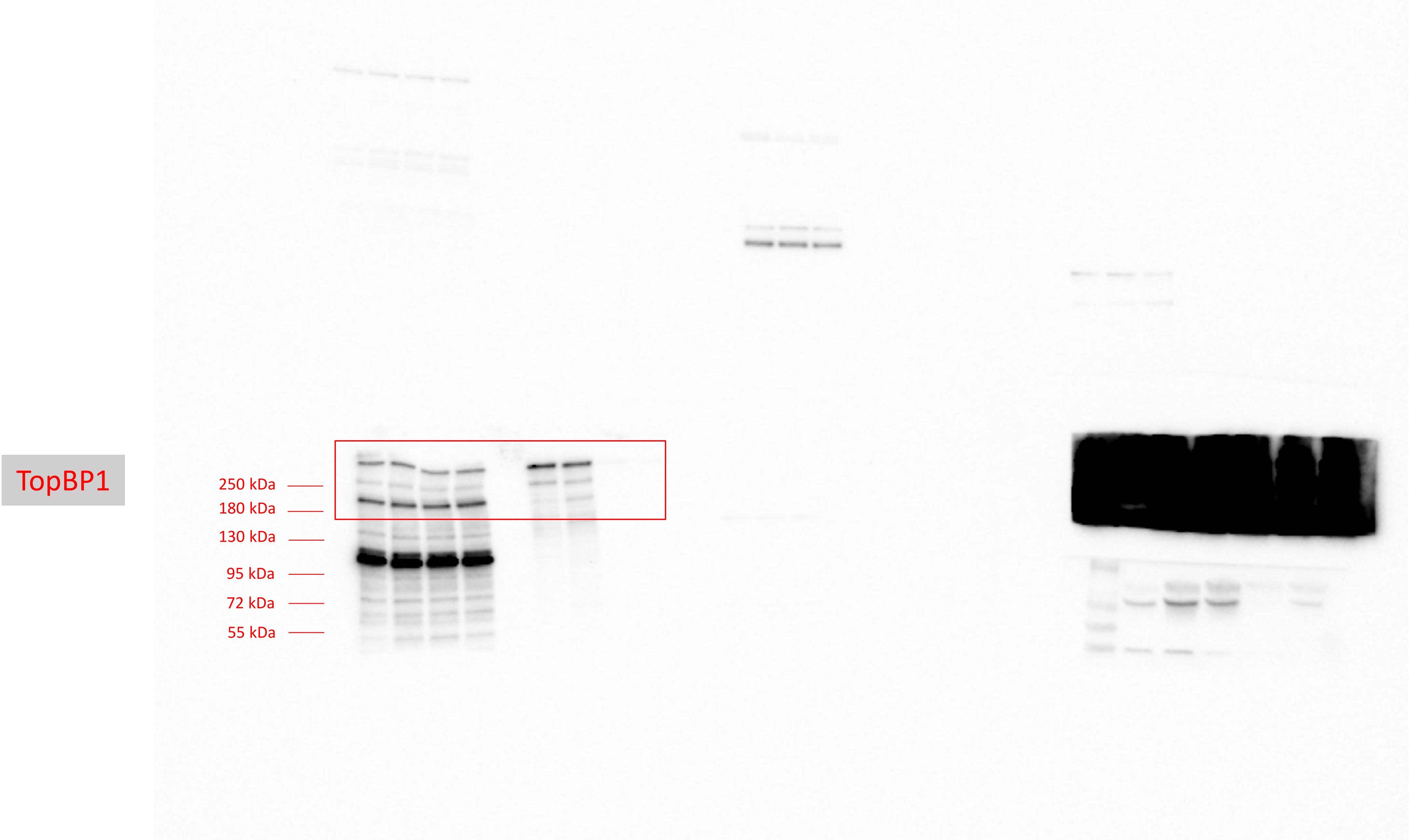

TopBP1

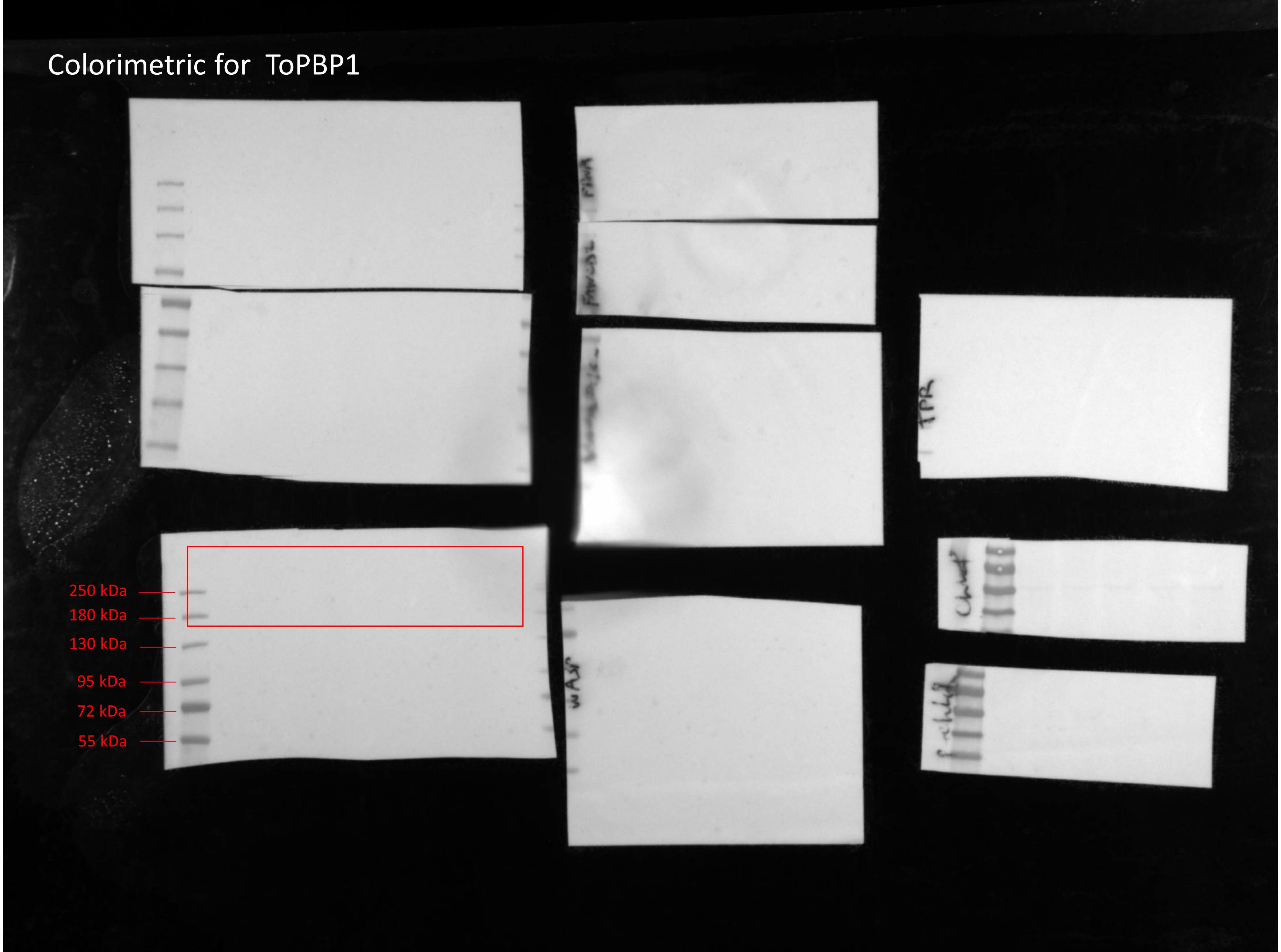

Colorimetric for ToPBP1
250 kDa
180 kDa
130 kDa
95 kDa
72 kDa
55 kDa
TPR
wASP
