# Supplementary figures and images for: TopBP1 biomolecular condensates as a new therapeutic target in advanced-stage colorectal cancer
Source: eLife. 2025 Oct 21;14:RP106196. doi: 10.7554/eLife.106196 (PMC12539802; doi:10.7554/eLife.106196)

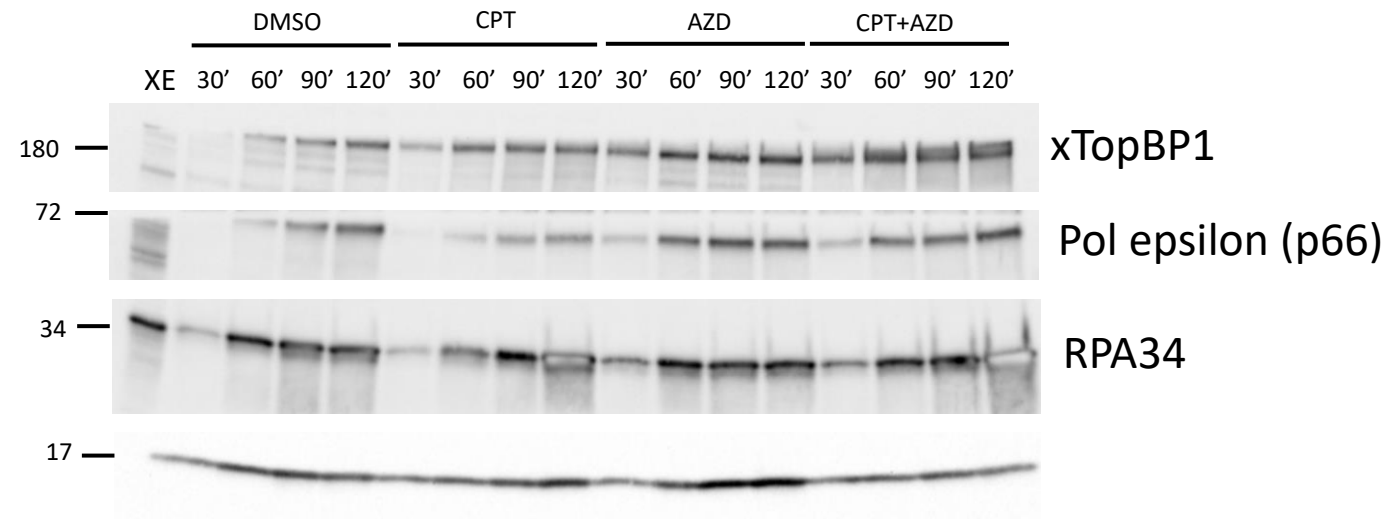

Supplement: Figure 2—source data 1. [file elife-106196-fig2-data1.zip › Fig 2B, D and E- Source Data 1/Fig2E -Source Data 1.pdf]

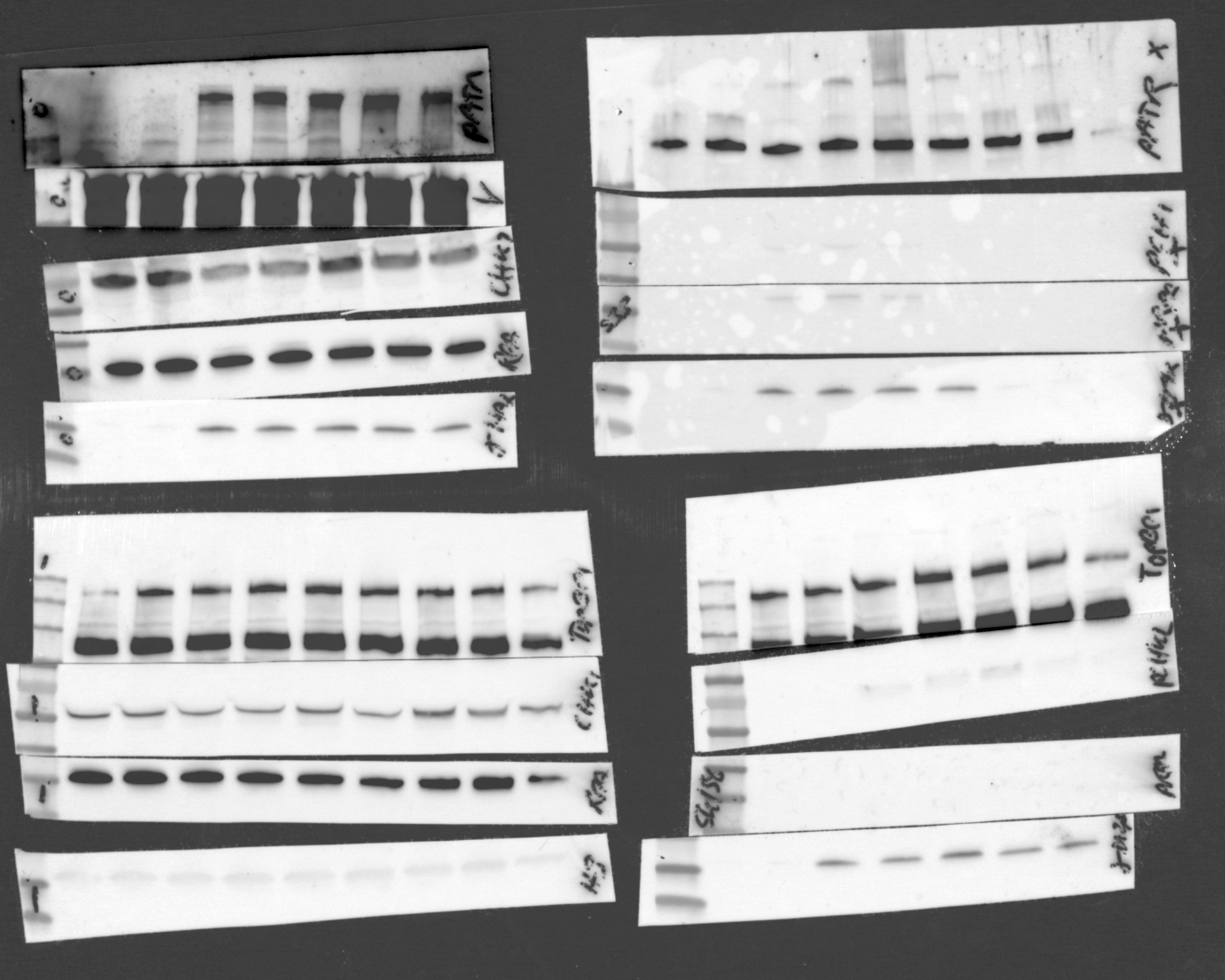

Supplement: Figure 2—source data 2. [file elife-106196-fig2-data2.zip › Fig 2B, D and E- Source Data 2/Fig 2B- Source Data 2/colo_lm334_gh2AX_pRPAS33_RPA_CHk1_CHK2_Vincu_TopBP1_pATR_pATM+lm334_pATR_gH2AX_laura m 2023-12-14 13h12m20s.tif]

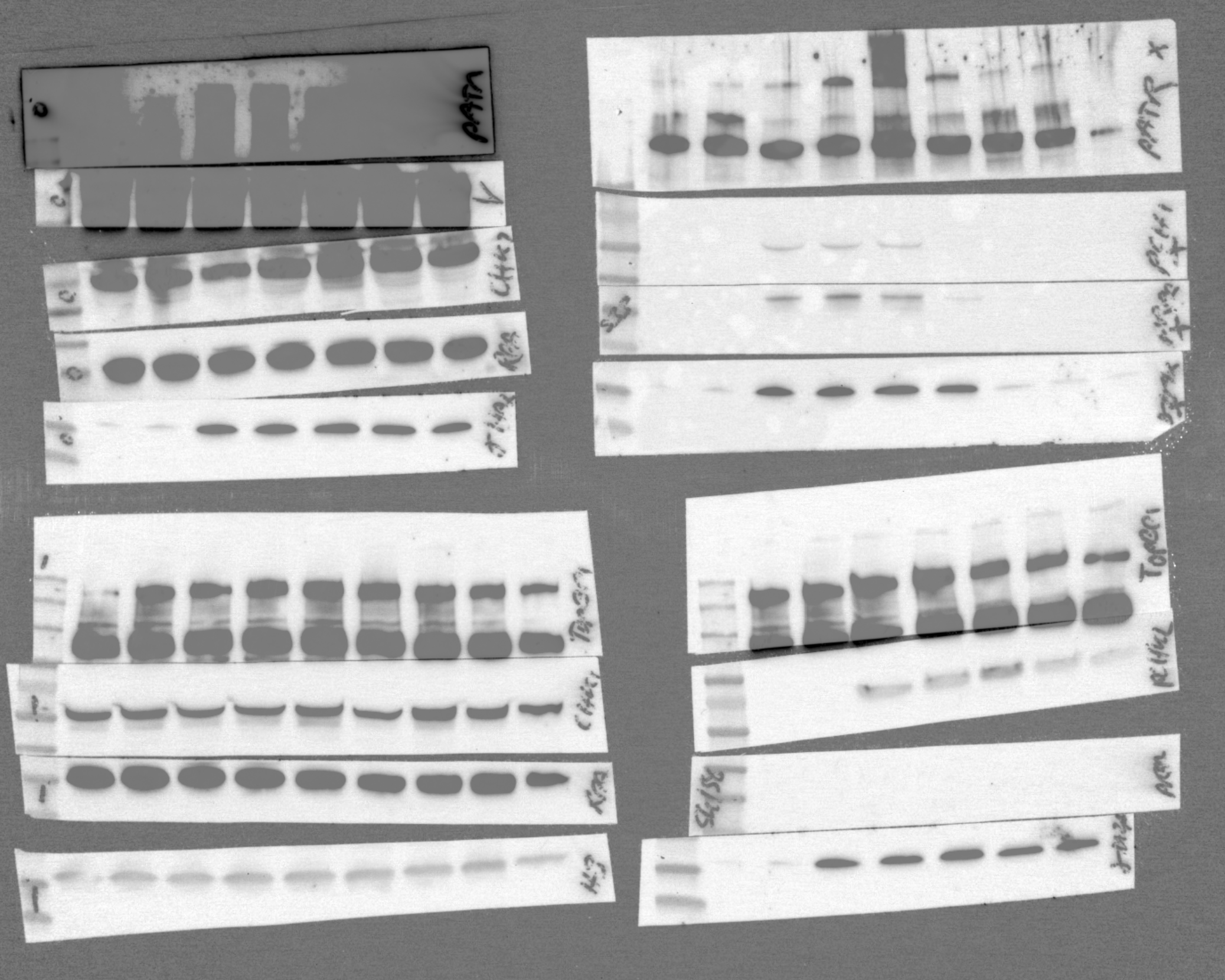

Supplement: Figure 2—source data 2. [file elife-106196-fig2-data2.zip › Fig 2B, D and E- Source Data 2/Fig 2B- Source Data 2/colo_lm334_gh2AX_pRPAS33_RPA_CHk1_CHK2_Vincu_TopBP1_pATR_pATM+lm334_pRPAS33_laura m 2023-12-14 13h18m14s.tif]

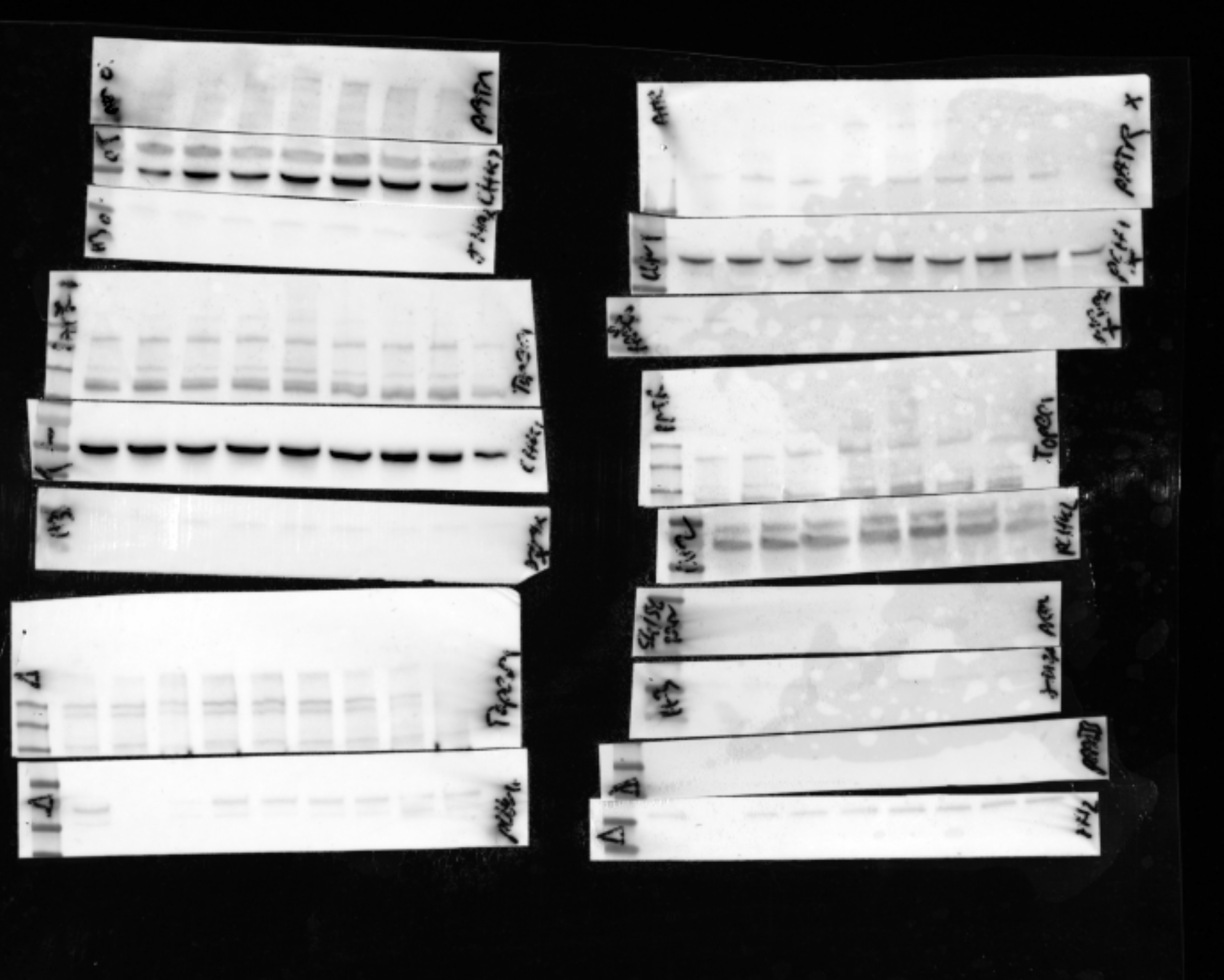

Supplement: Figure 2—source data 2. [file elife-106196-fig2-data2.zip › Fig 2B, D and E- Source Data 2/Fig 2B- Source Data 2/colo_lm334_h3_chk_chk2_tubu_patr_lm336_gh2ax_prpaS33_pchk1_topbp1+lm334_CHK1_CHK2_Tubu_laura m 2023-12-15 16h31m59s.tif]

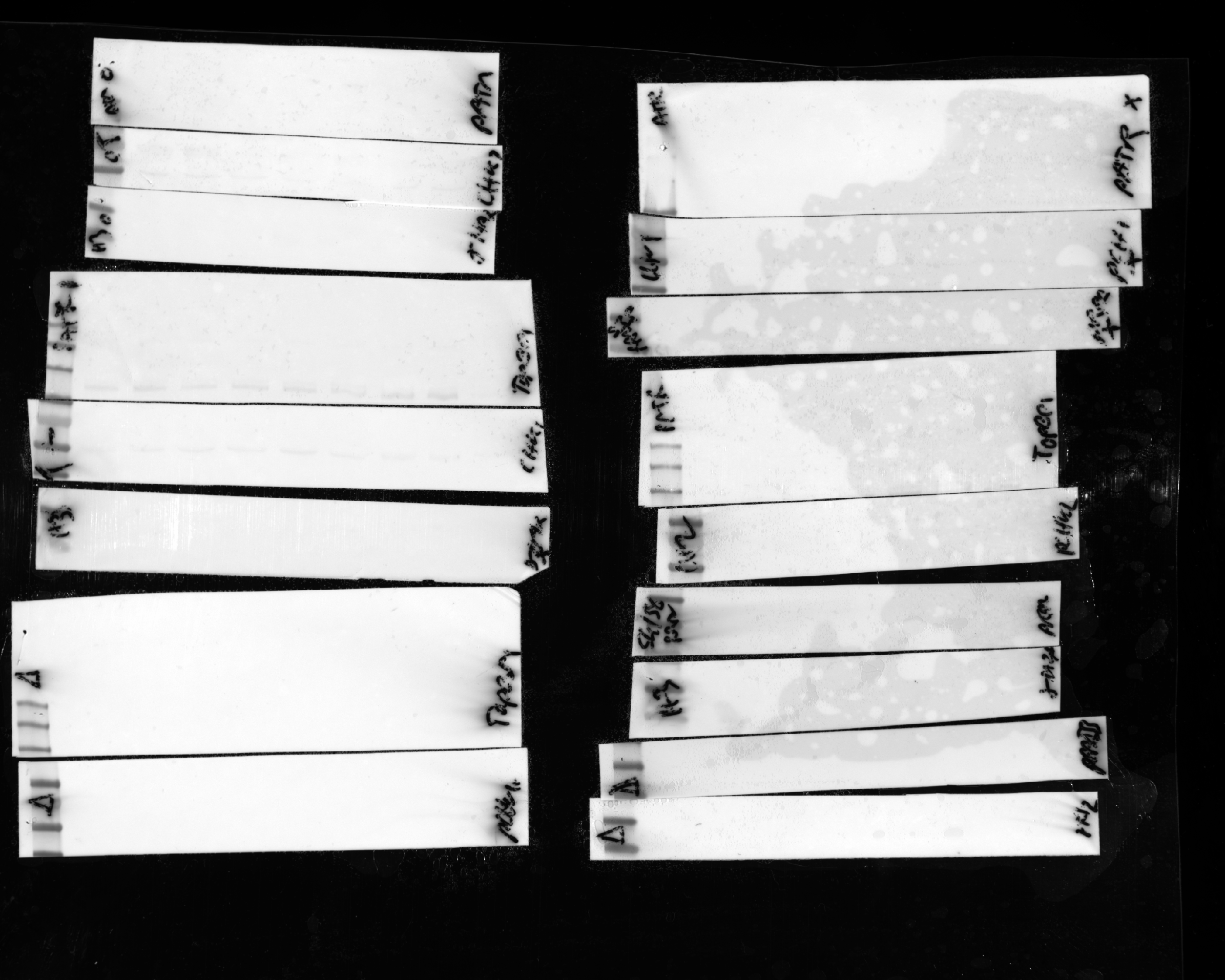

Supplement: Figure 2—source data 2. [file elife-106196-fig2-data2.zip › Fig 2B, D and E- Source Data 2/Fig 2B- Source Data 2/colo_lm334_h3_chk_chk2_tubu_patr_lm336_gh2ax_prpaS33_pchk1_topbp1.tif]

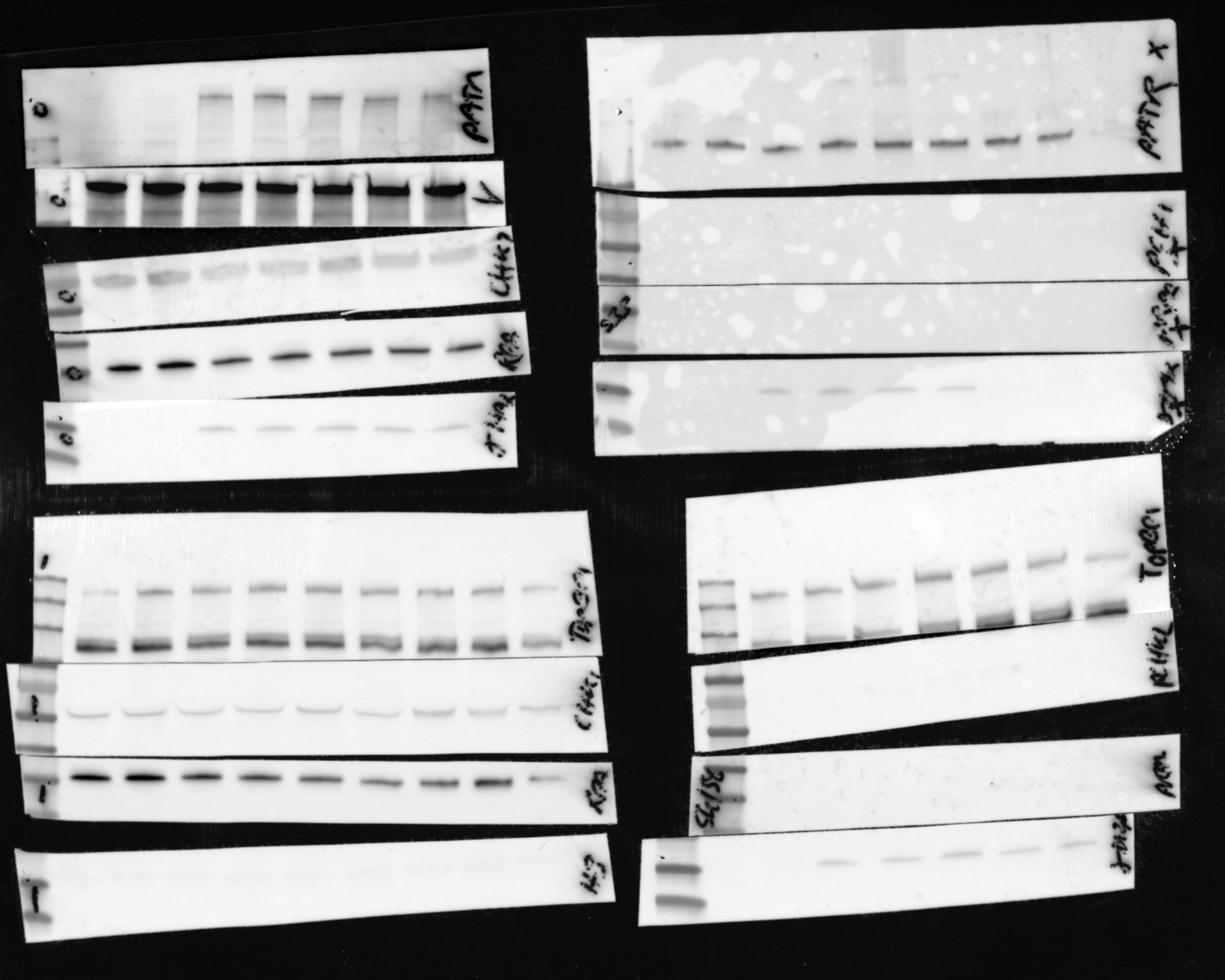

Supplement: Figure 2—source data 2. [file elife-106196-fig2-data2.zip › Fig 2B, D and E- Source Data 2/Fig 2B- Source Data 2/lm334_pATM_TopBP1_RPA_laura m 2023-12-14 13h11m14s+colo_lm334_gh2AX_pRPAS33_RPA_CHk1_CHK2_Vincu_TopBP1_pATR_pATM.tif]

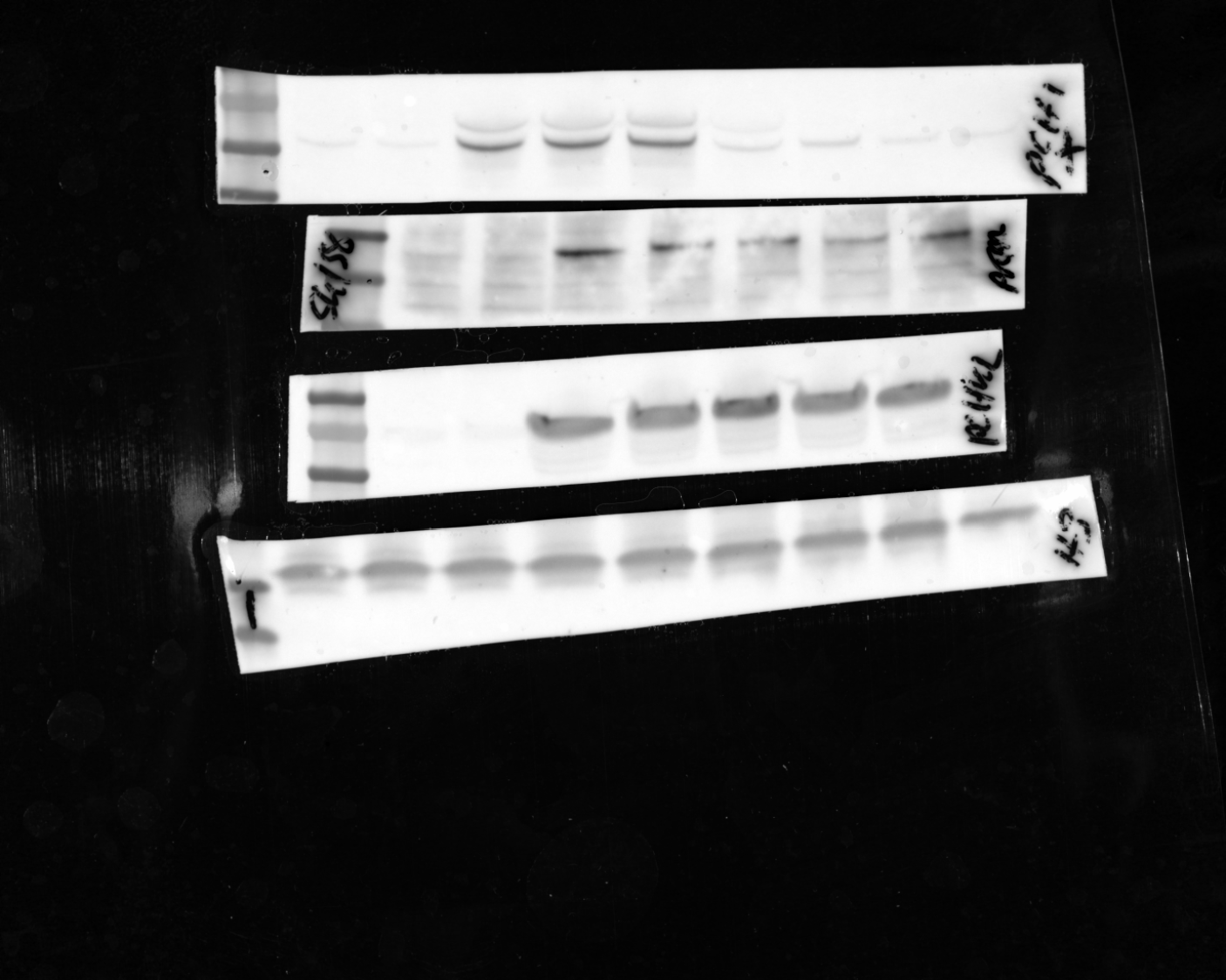

Supplement: Figure 2—source data 2. [file elife-106196-fig2-data2.zip › Fig 2B, D and E- Source Data 2/Fig 2B- Source Data 2/lm334_pCHK2_pCHK1_pRPAS4-8_H3_laura m 2023-12-14 13h33m29s+colo_lm334_h3_prpaS4-8_pCHK1_pCHK2.tif]

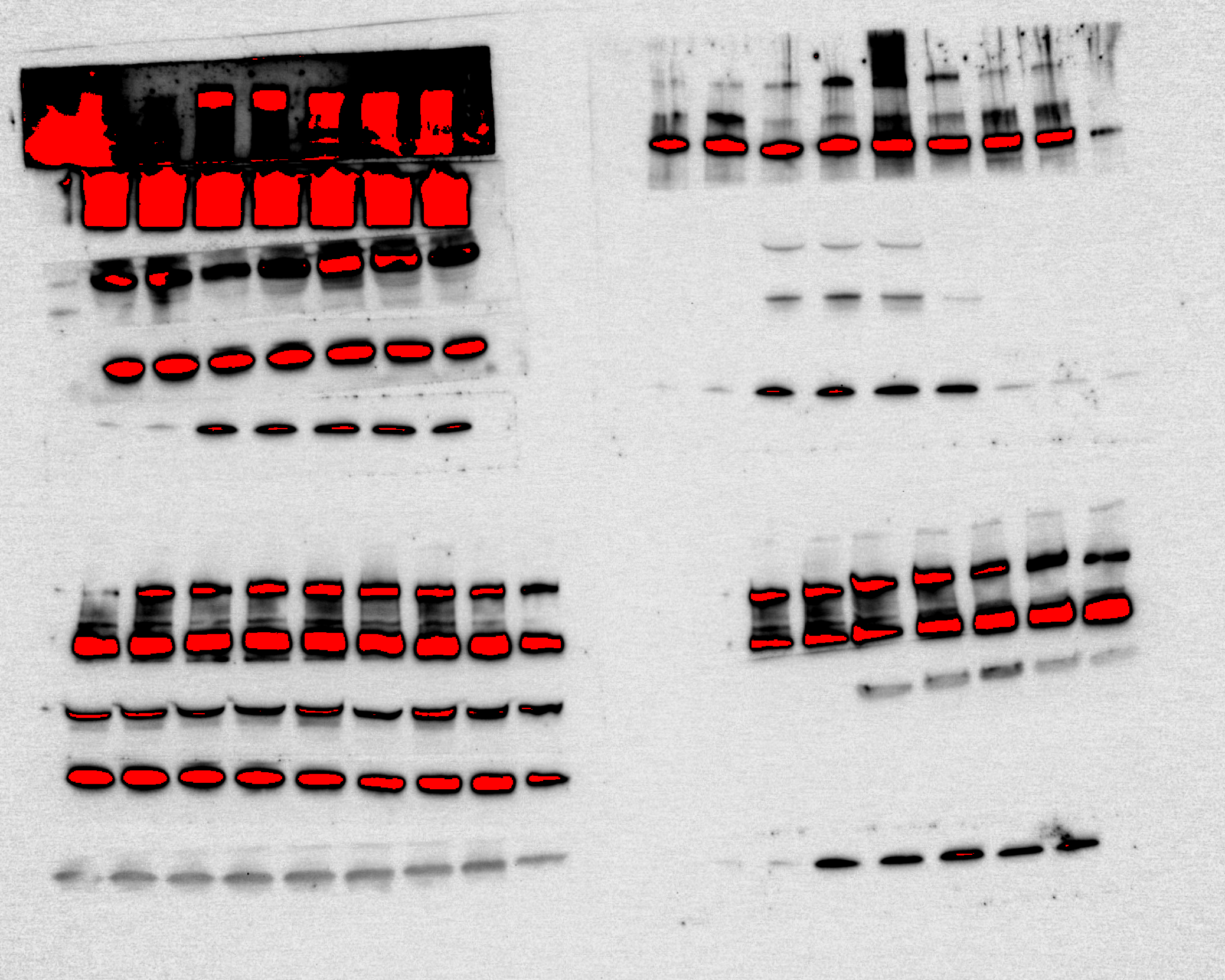

Supplement: Figure 2—source data 2. [file elife-106196-fig2-data2.zip › Fig 2B, D and E- Source Data 2/Fig 2B- Source Data 2/lm334_pRPAS33_pCHK1low_laura m 2023-12-14 13h18m14s.tif]

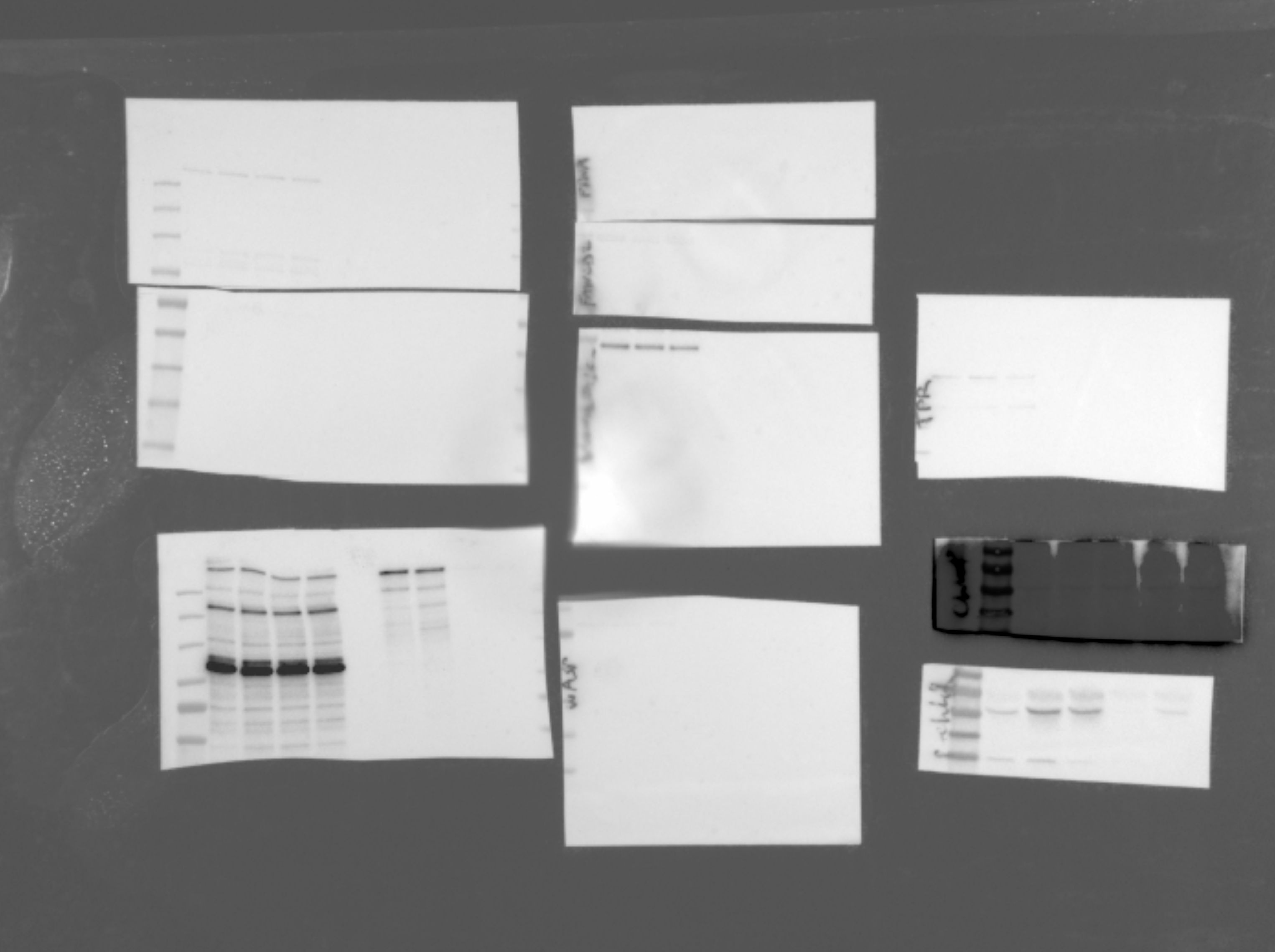

Supplement: Figure 2—source data 2. [file elife-106196-fig2-data2.zip › Fig 2B, D and E- Source Data 2/Fig 2D- Source Data 2/colo+lm380_topbp1_24-06-13 12hr 17min_Exposure_29.7sec.tif]

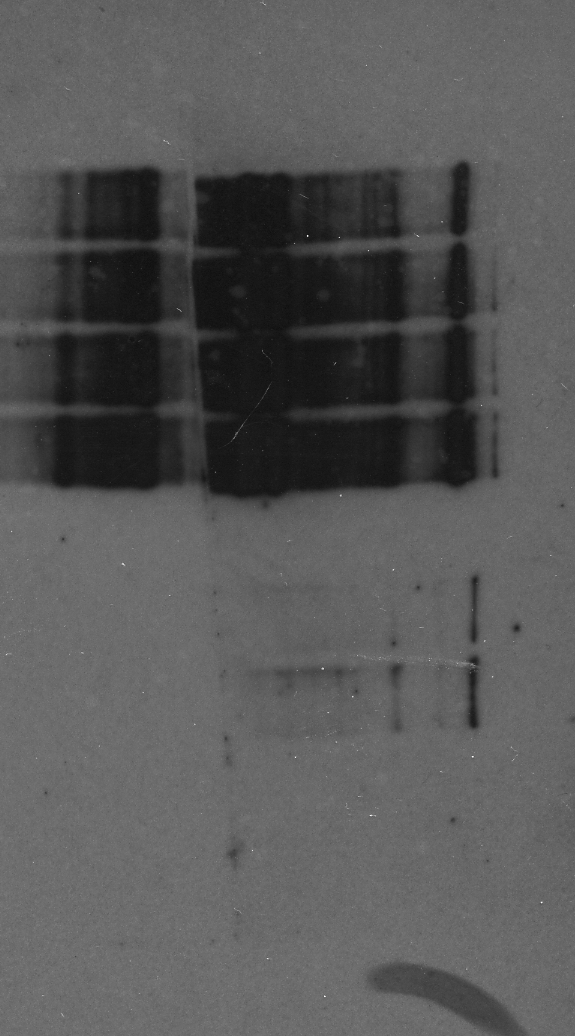

Supplement: Figure 2—source data 2. [file elife-106196-fig2-data2.zip › Fig 2B, D and E- Source Data 2/Fig 2D- Source Data 2/lm380_ATR_2024-05-24 sg3004.tif]

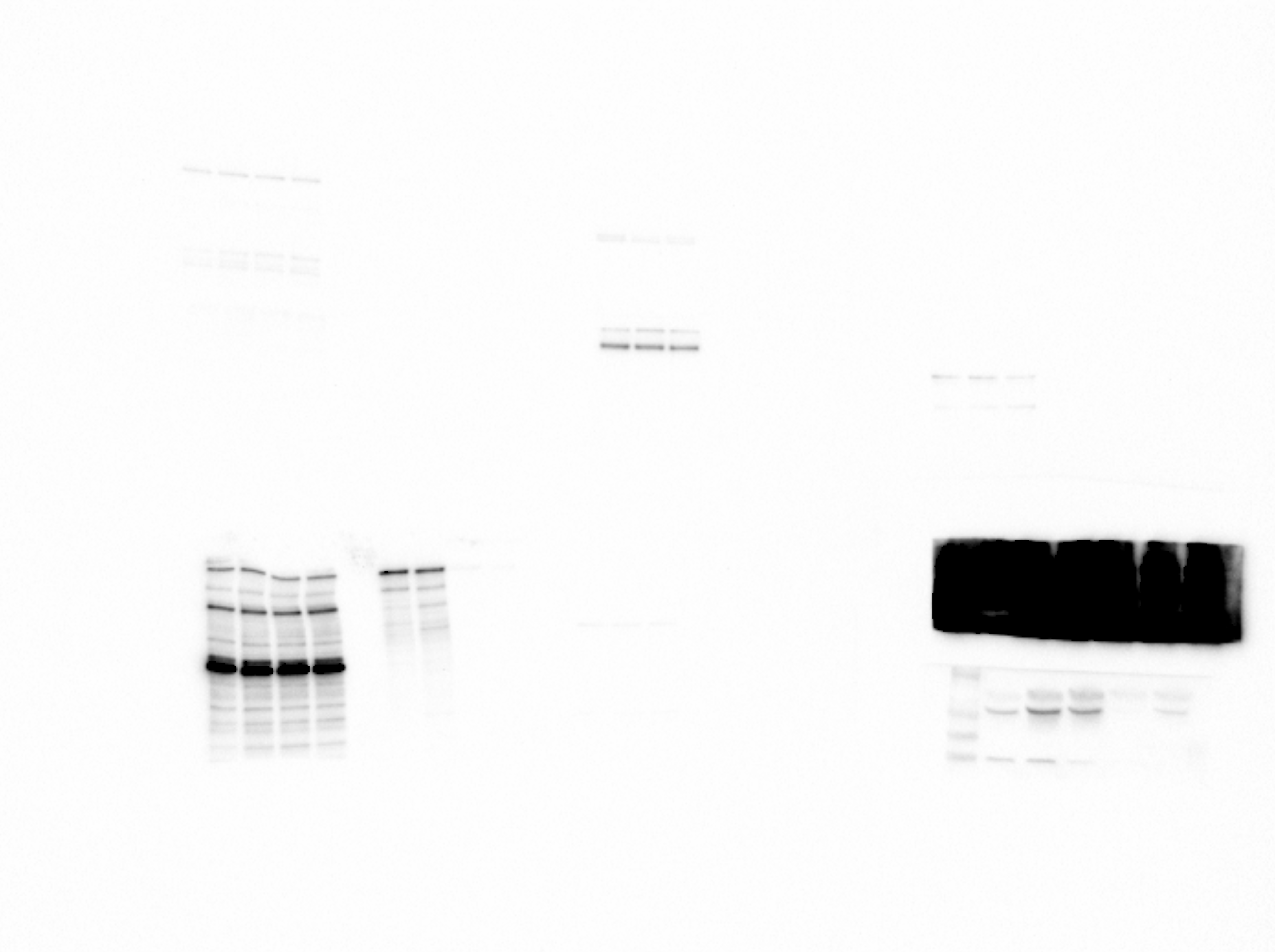

Supplement: Figure 2—source data 2. [file elife-106196-fig2-data2.zip › Fig 2B, D and E- Source Data 2/Fig 2D- Source Data 2/lm380_topbp1_24-06-13 12hr 17min_Exposure_29.7sec.tif]

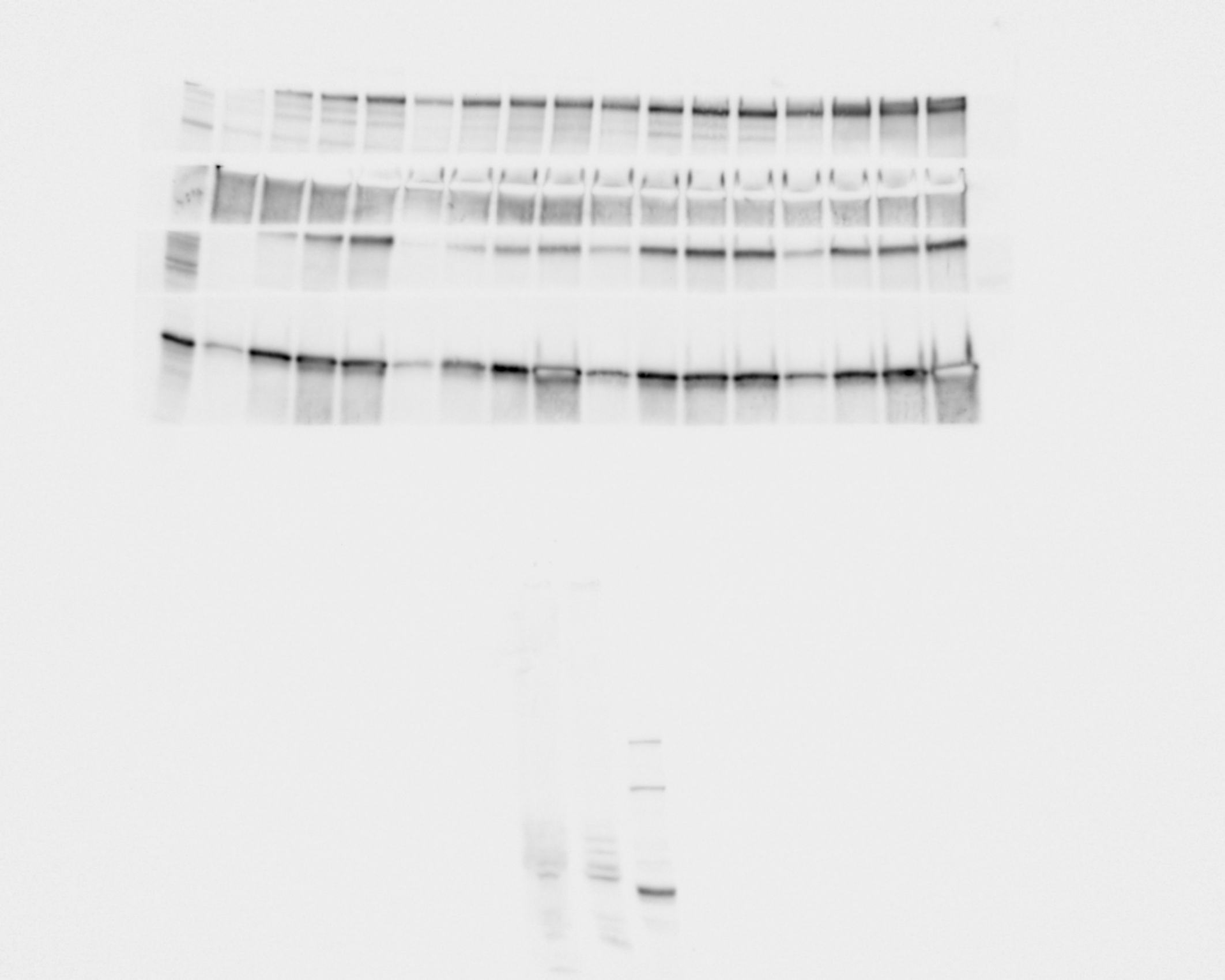

Supplement: Figure 2—source data 2. [file elife-106196-fig2-data2.zip › Fig 2B, D and E- Source Data 2/Fig 2E- Source Data 2/antoine a 2024-06-20 13h06m23s(Chemiluminescence).tif]

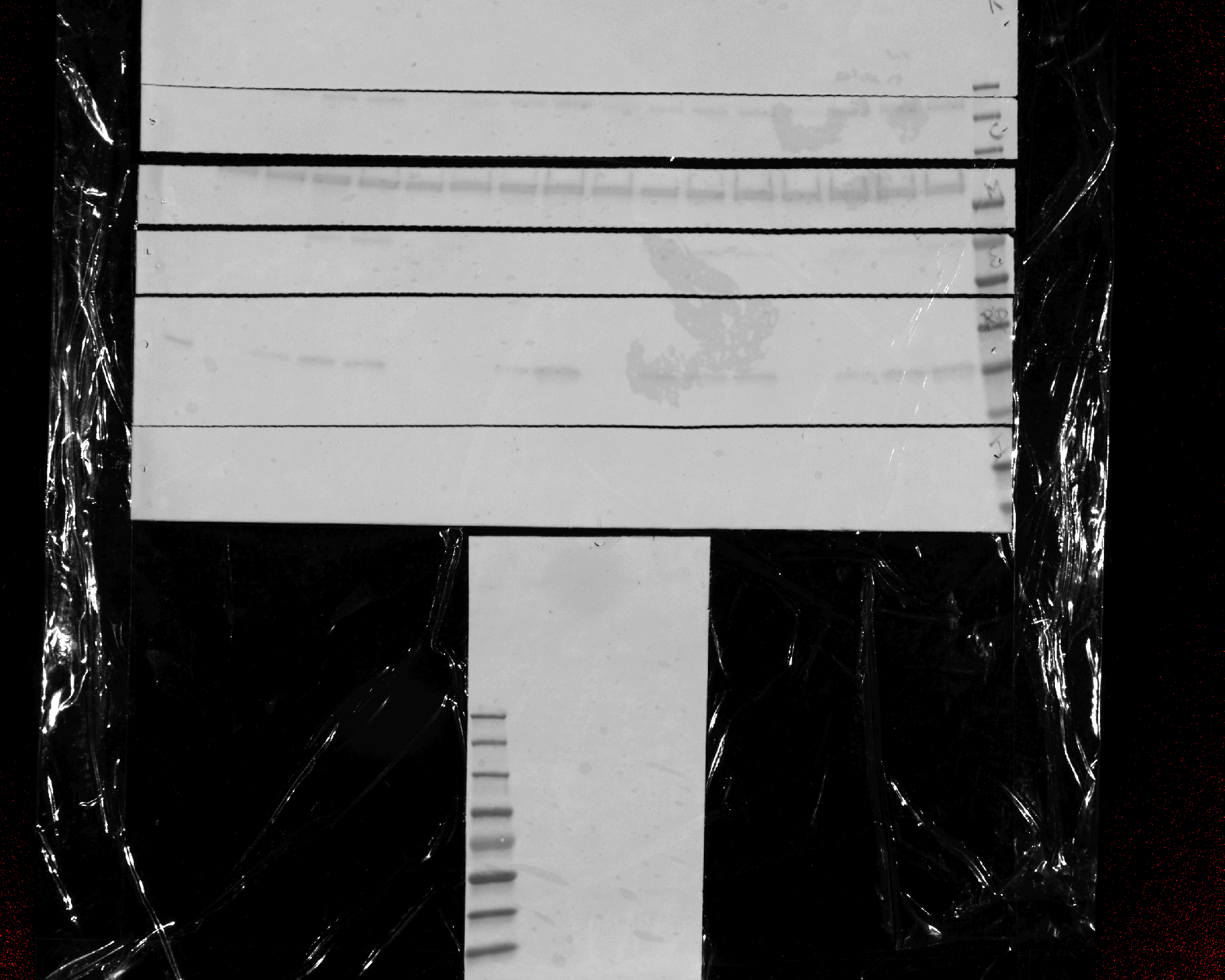

Supplement: Figure 2—source data 2. [file elife-106196-fig2-data2.zip › Fig 2B, D and E- Source Data 2/Fig 2E- Source Data 2/antoine a 2024-06-20 13h17m18s(Ponceau S).tif]

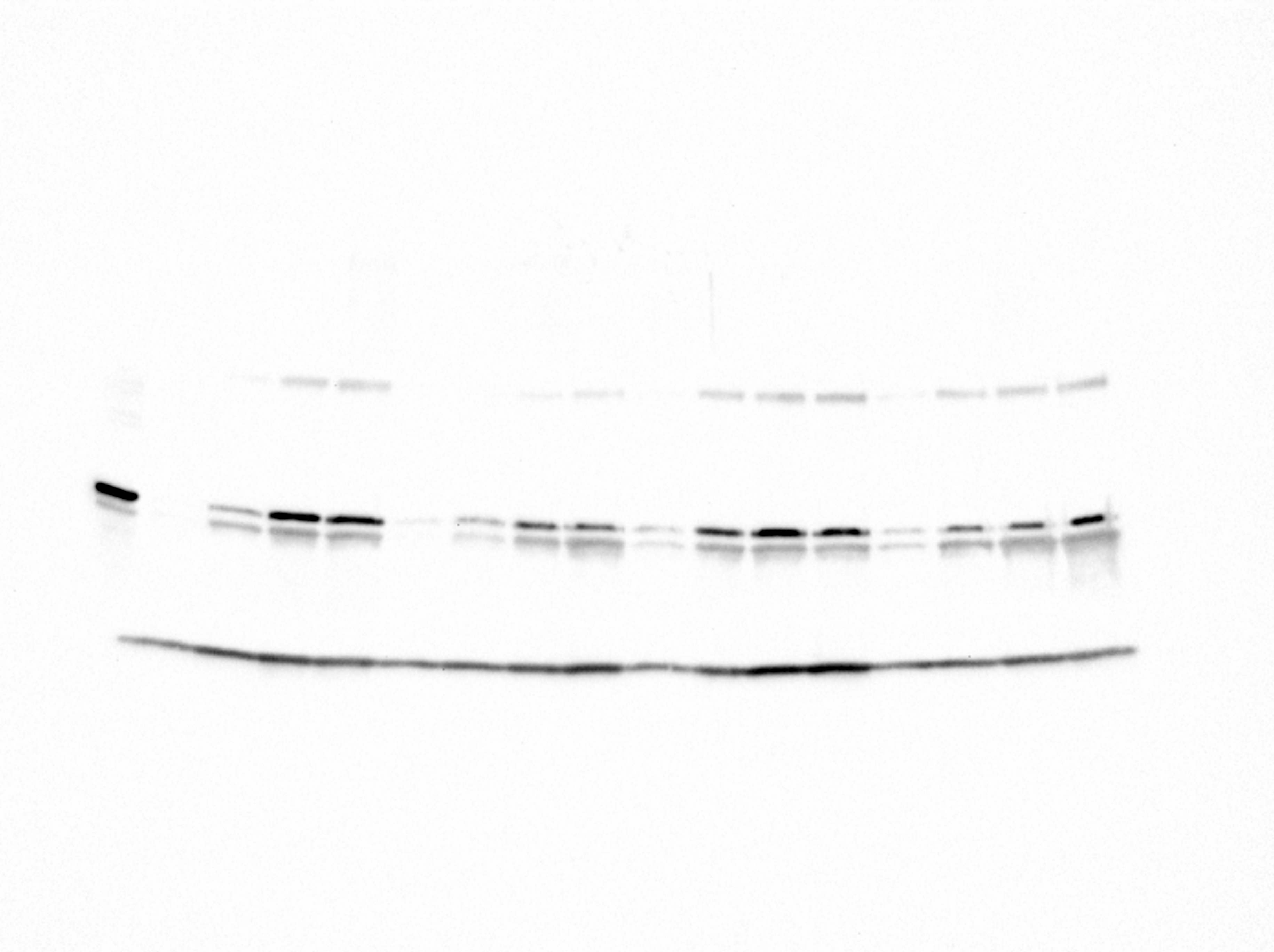

Supplement: Figure 2—source data 2. [file elife-106196-fig2-data2.zip › Fig 2B, D and E- Source Data 2/Fig 2E- Source Data 2/commun igh 2024-06-21 17hr 46min_Exposure_42.2sec.tif]

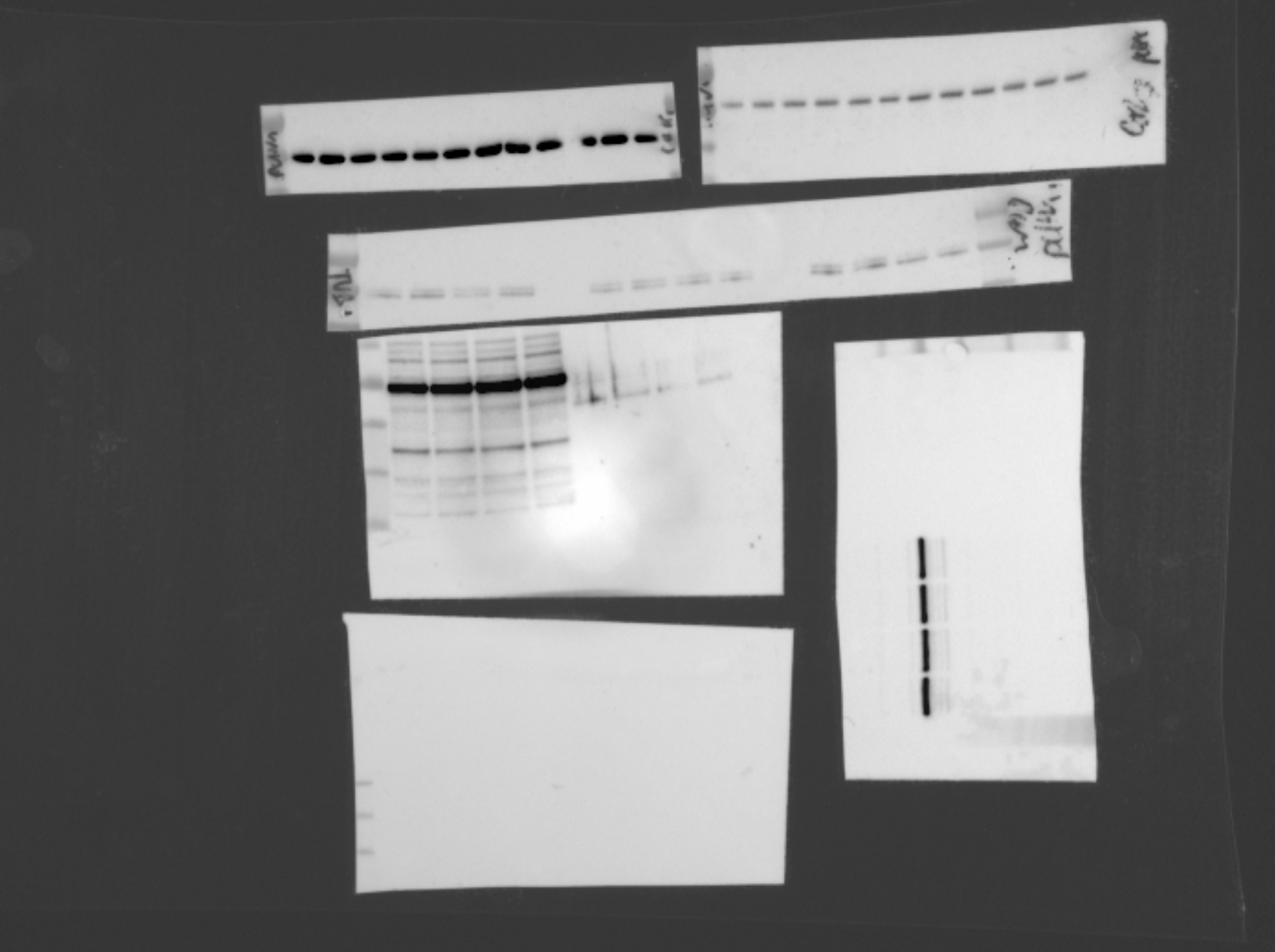

Supplement: Figure 2—figure supplement 3—source data 2. [file elife-106196-fig2-figsupp3-data2.zip › Figure 2-figure supplement 3A and 3B-Source Data 2/Figure 2-figure supplement 3A-Source Data2/colo-Chk1_commun igh 2024-03-08 12hr 24min+lm361_CHK1_commun igh 2024-03-08 12hr 23min_Exposure_11.2sec.tif]

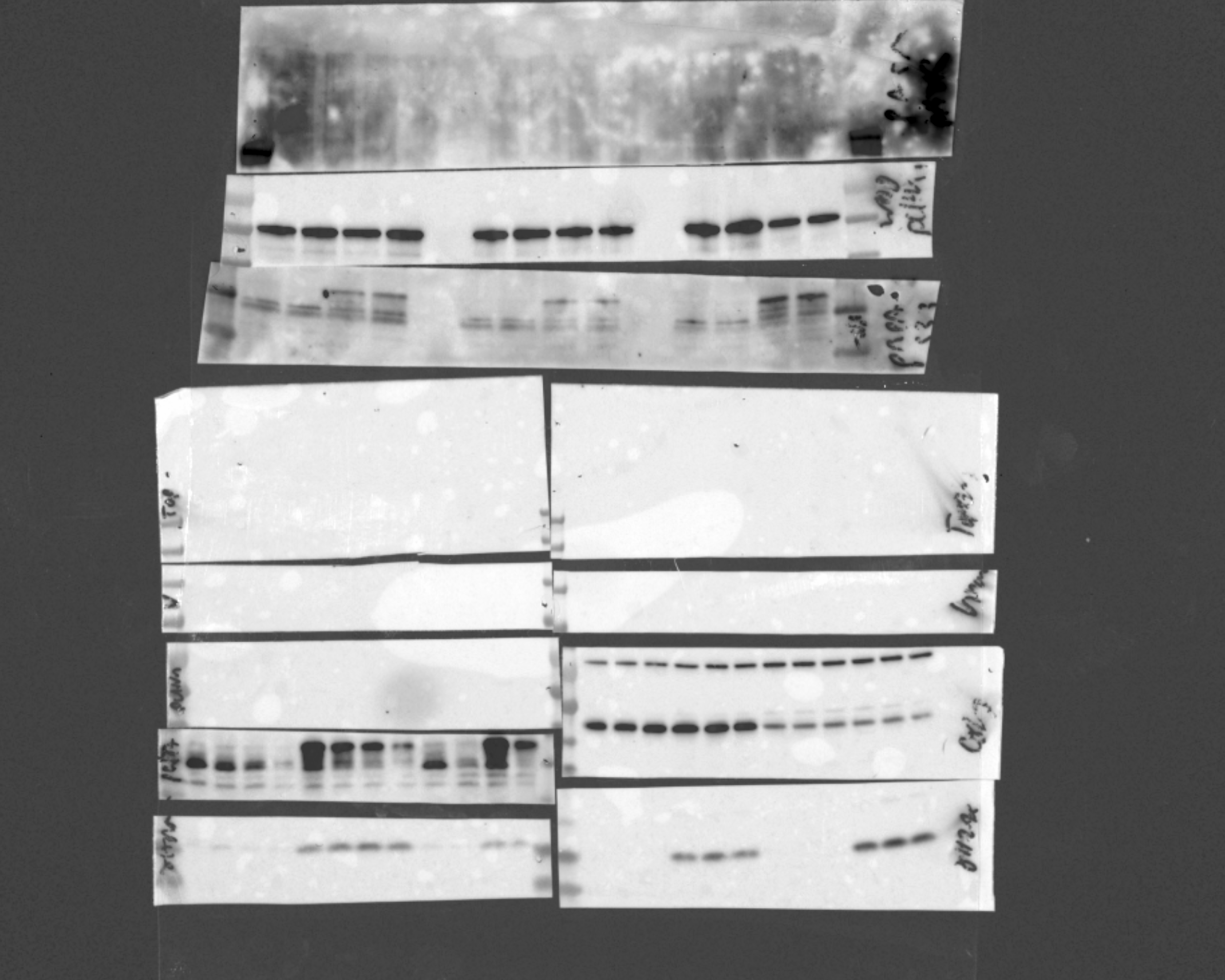

Supplement: Figure 2—figure supplement 3—source data 2. [file elife-106196-fig2-figsupp3-data2.zip › Figure 2-figure supplement 3A and 3B-Source Data 2/Figure 2-figure supplement 3A-Source Data2/lm360_lm361_gh2ax_laura m 2024-03-06 14h04m43s+colo_pGSK3_gH2AX_laura m 2024-03-06 14h11m48s.tif]

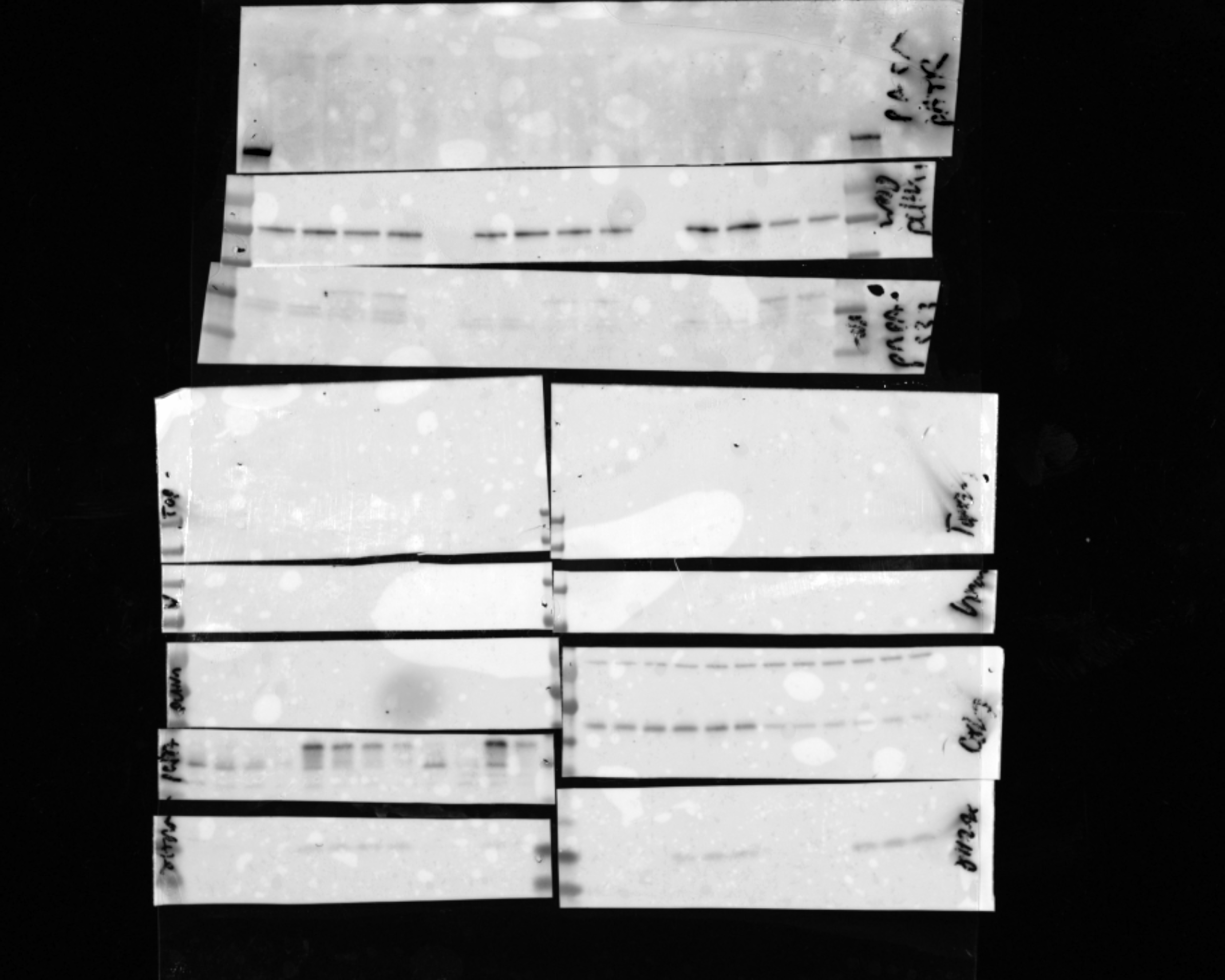

Supplement: Figure 2—figure supplement 3—source data 2. [file elife-106196-fig2-figsupp3-data2.zip › Figure 2-figure supplement 3A and 3B-Source Data 2/Figure 2-figure supplement 3A-Source Data2/lm361_gsk3_laura m 2024-03-06 14h03m10s+colo_pGSK3_gH2AX_laura m 2024-03-06 14h11m48s.tif]

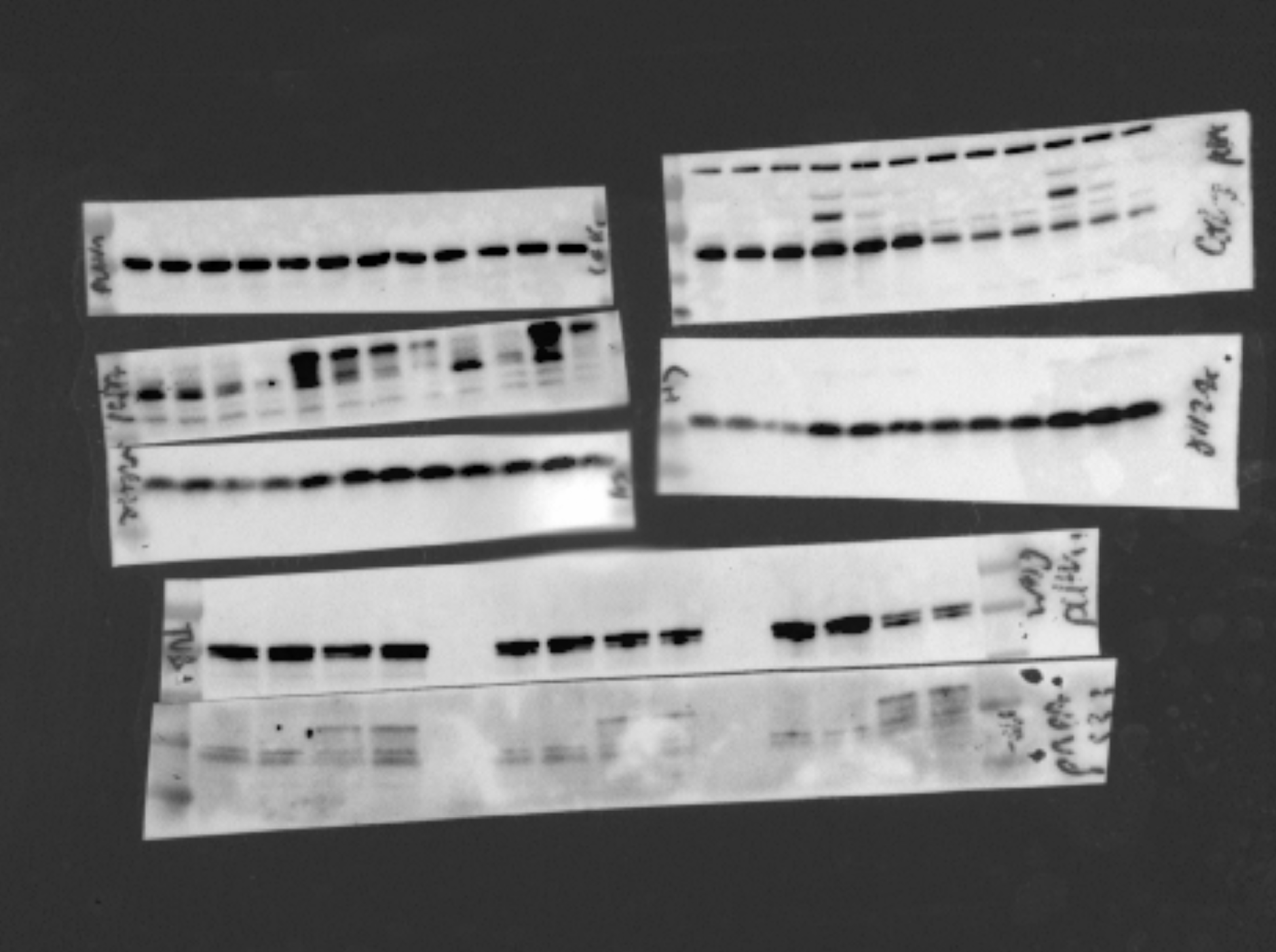

Supplement: Figure 2—figure supplement 3—source data 2. [file elife-106196-fig2-figsupp3-data2.zip › Figure 2-figure supplement 3A and 3B-Source Data 2/Figure 2-figure supplement 3A-Source Data2/lm361_pCHK1_commun igh 2024-03-07 11hr 22min_Exposure_62.4sec+colopChk1_commun igh 2024-03-07 11hr 28min.tif]

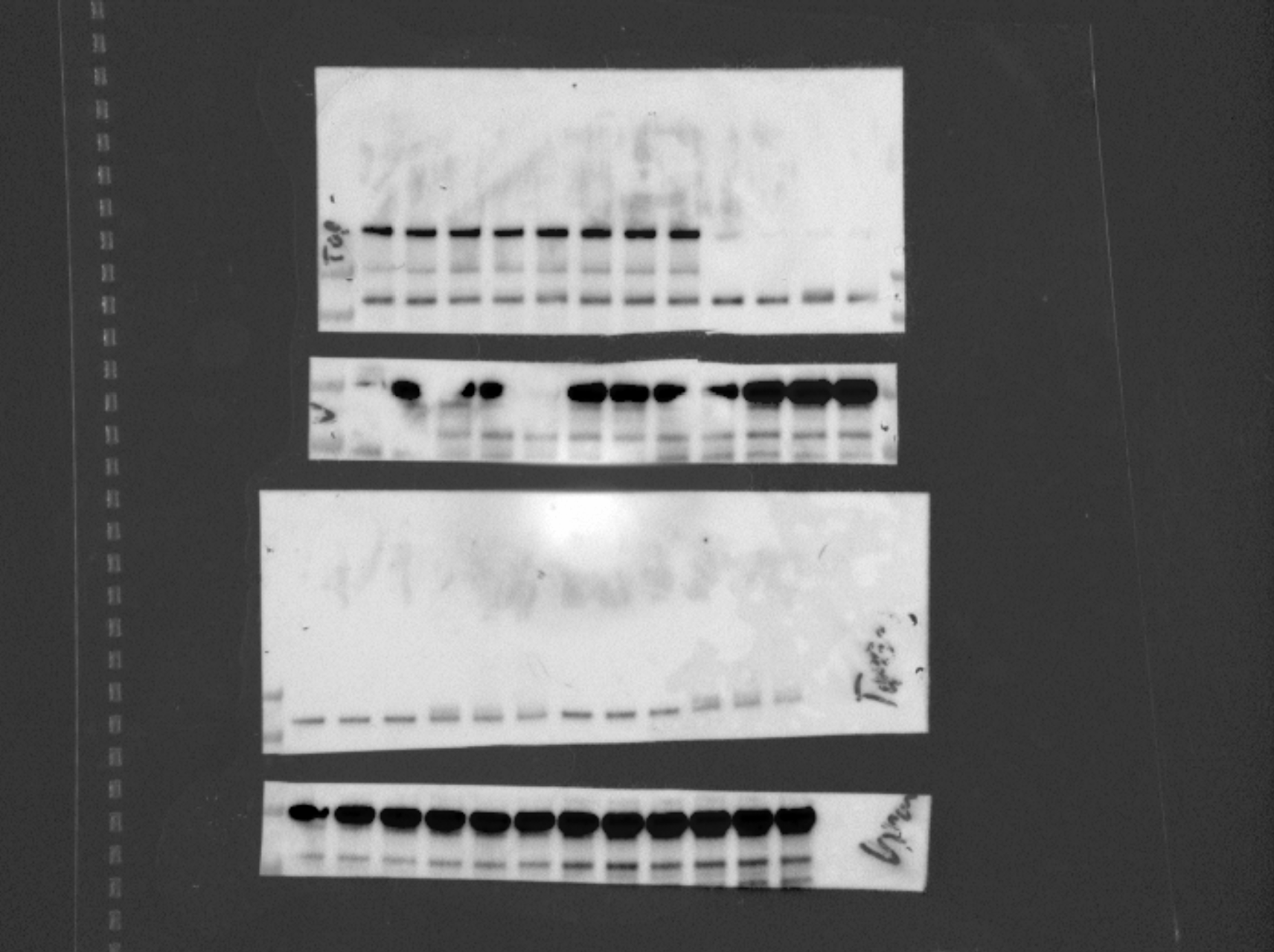

Supplement: Figure 2—figure supplement 3—source data 2. [file elife-106196-fig2-figsupp3-data2.zip › Figure 2-figure supplement 3A and 3B-Source Data 2/Figure 2-figure supplement 3A-Source Data2/lm361_TopBP1_commun igh 2024-03-06 16hr 07min_Exposure_34.0sec+colo_vincu_top_2024-03-06 16hr 08min.tif]

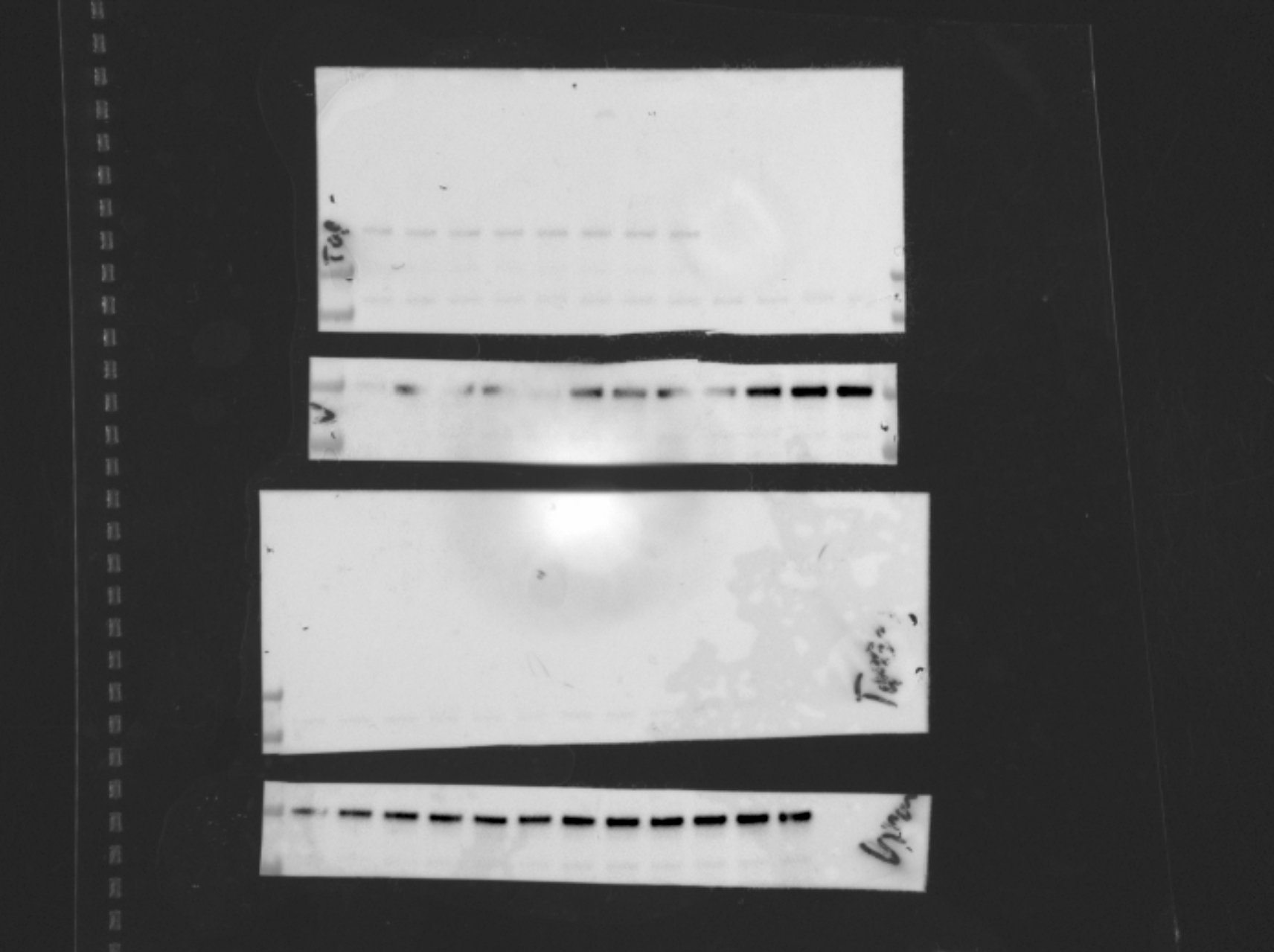

Supplement: Figure 2—figure supplement 3—source data 2. [file elife-106196-fig2-figsupp3-data2.zip › Figure 2-figure supplement 3A and 3B-Source Data 2/Figure 2-figure supplement 3A-Source Data2/lm361_vincu_commun igh 2024-03-06 16hr 07min_Exposure_1.0sec+colo_vincu_top_2024-03-06 16hr 08min.tif]

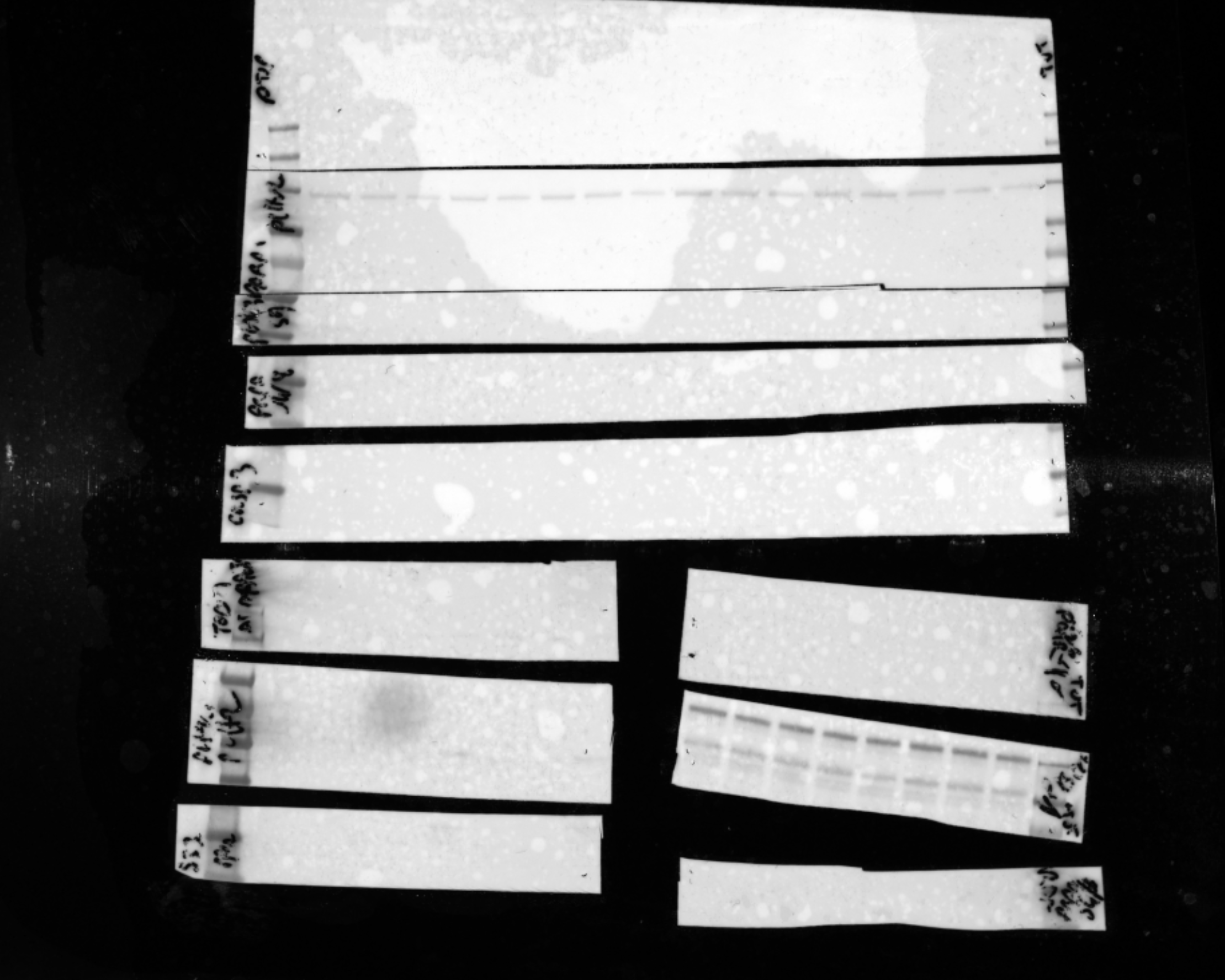

Supplement: Figure 2—figure supplement 3—source data 2. [file elife-106196-fig2-figsupp3-data2.zip › Figure 2-figure supplement 3A and 3B-Source Data 2/Figure 2-figure supplement 3B-Source Data2/c-vincu m 2024-03-27 11h16m56s+lm366_vincu_laura m 2024-03-27 11h03m49s.tif]

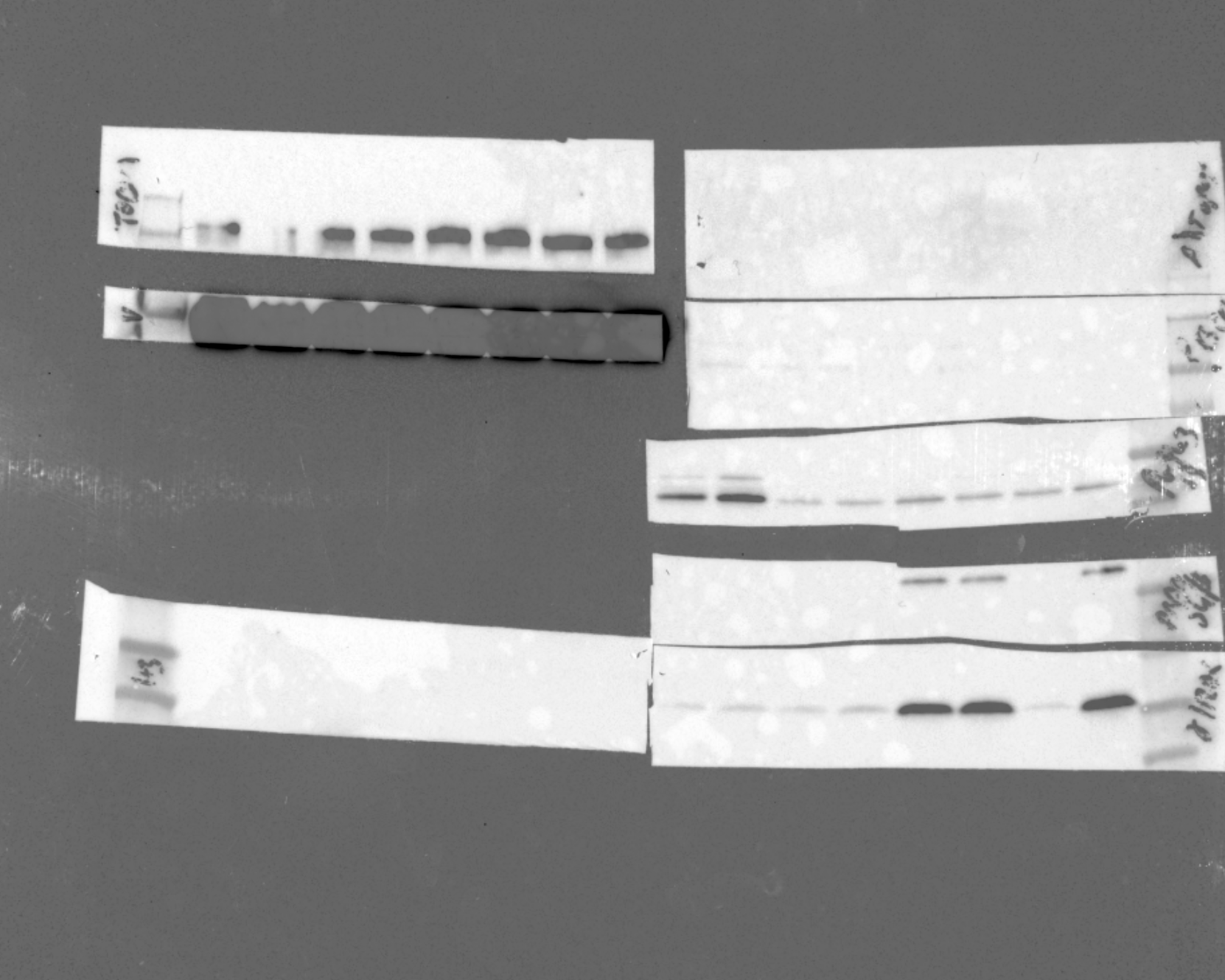

Supplement: Figure 2—figure supplement 3—source data 2. [file elife-106196-fig2-figsupp3-data2.zip › Figure 2-figure supplement 3A and 3B-Source Data 2/Figure 2-figure supplement 3B-Source Data2/colo_d1_pGSK3_vincu_laura 2024-03-25 18h34m52s+lm366_pGSK3Ser9_pRPAS4-8_laura 2024-03-25 18h29m35s.tif]

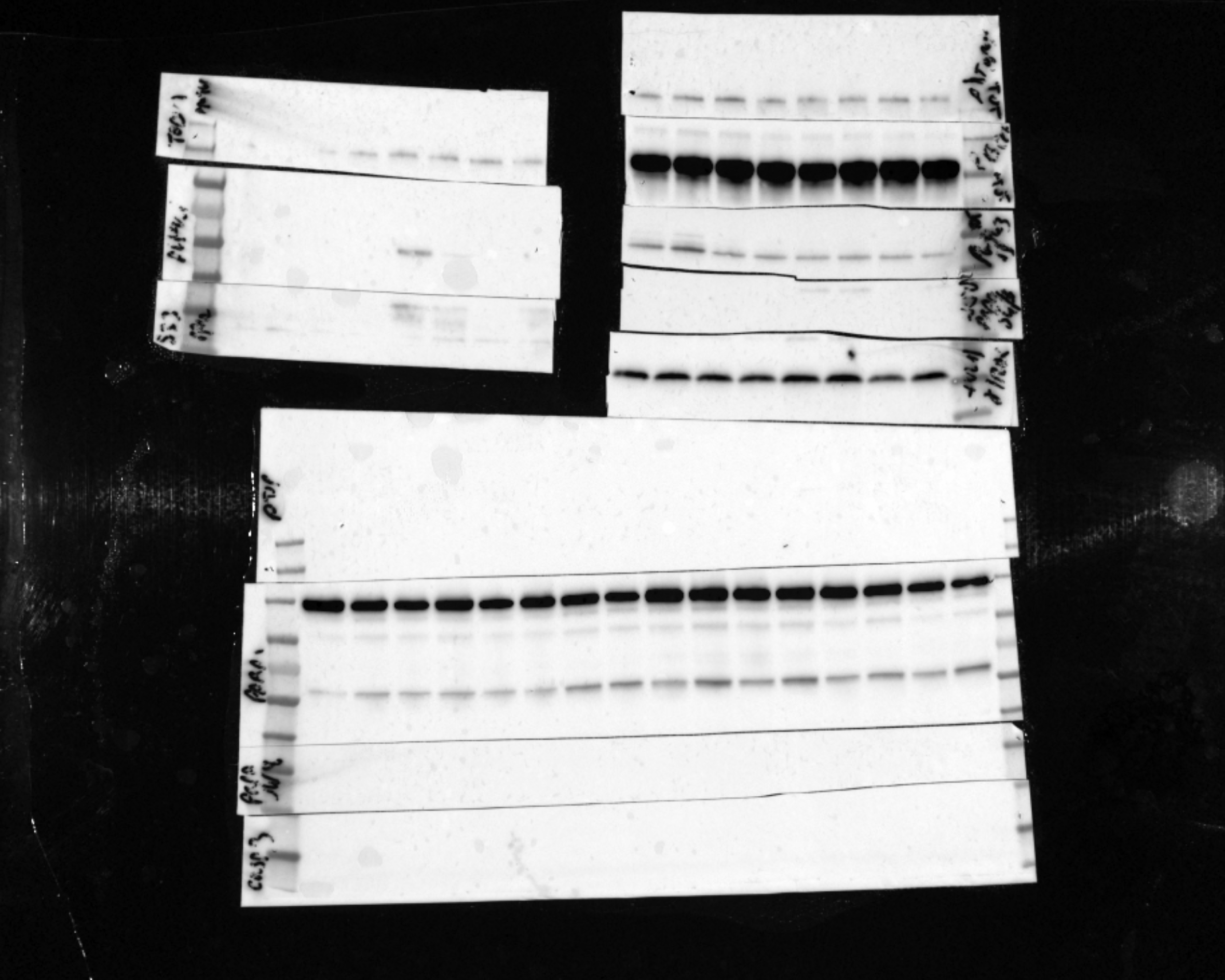

Supplement: Figure 2—figure supplement 3—source data 2. [file elife-106196-fig2-figsupp3-data2.zip › Figure 2-figure supplement 3A and 3B-Source Data 2/Figure 2-figure supplement 3B-Source Data2/colo_d2_lm366laura m 2024-03-26 11h51m18s+lm366_h2ax_gsk3_laura m 2024-03-26 11h42m20s.tif]

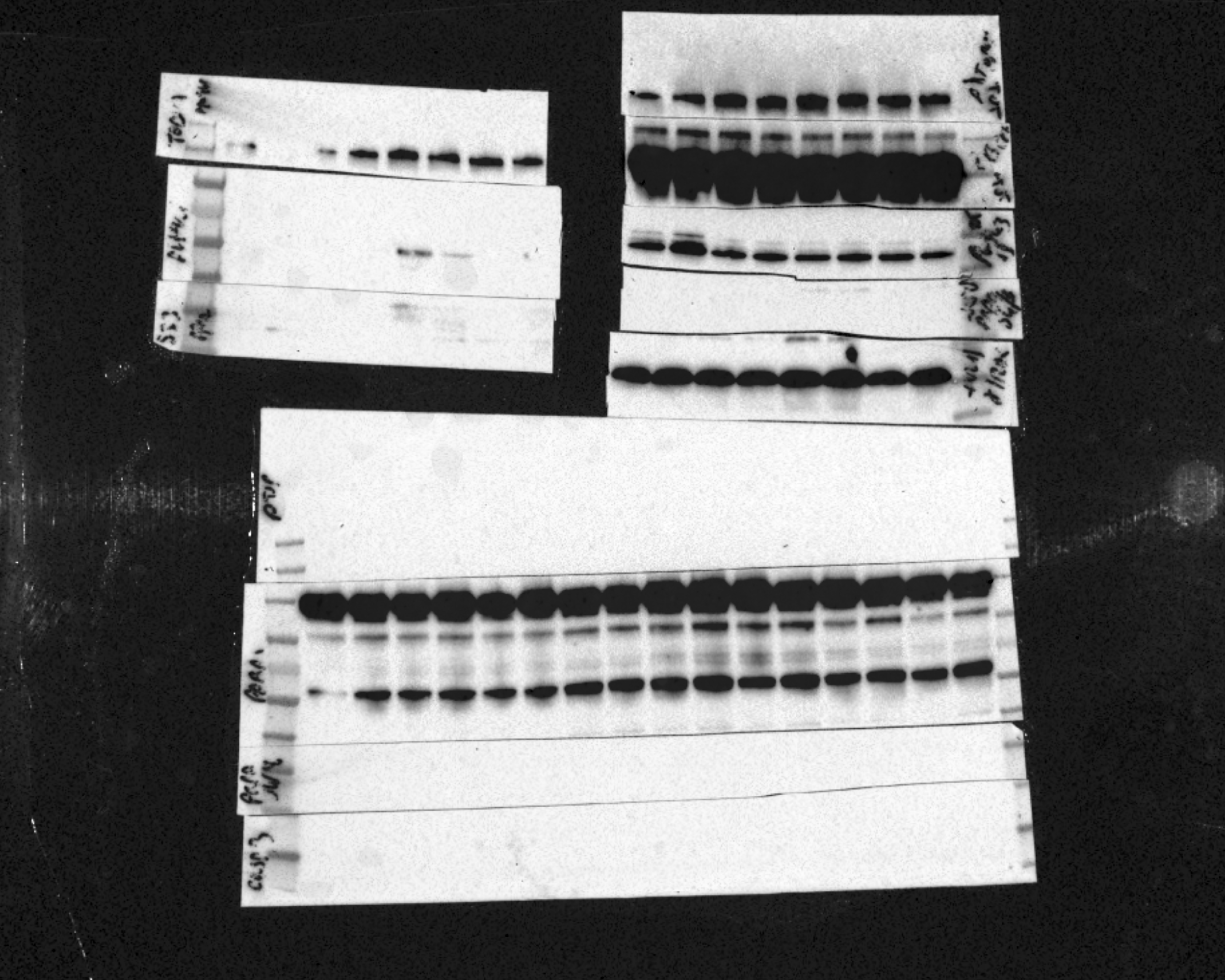

Supplement: Figure 2—figure supplement 3—source data 2. [file elife-106196-fig2-figsupp3-data2.zip › Figure 2-figure supplement 3A and 3B-Source Data 2/Figure 2-figure supplement 3B-Source Data2/colo_d2_lm366laura m 2024-03-26 11h51m18s+lm366_pchk1_low_laura m 2024-03-26 11h48m56s.tif]

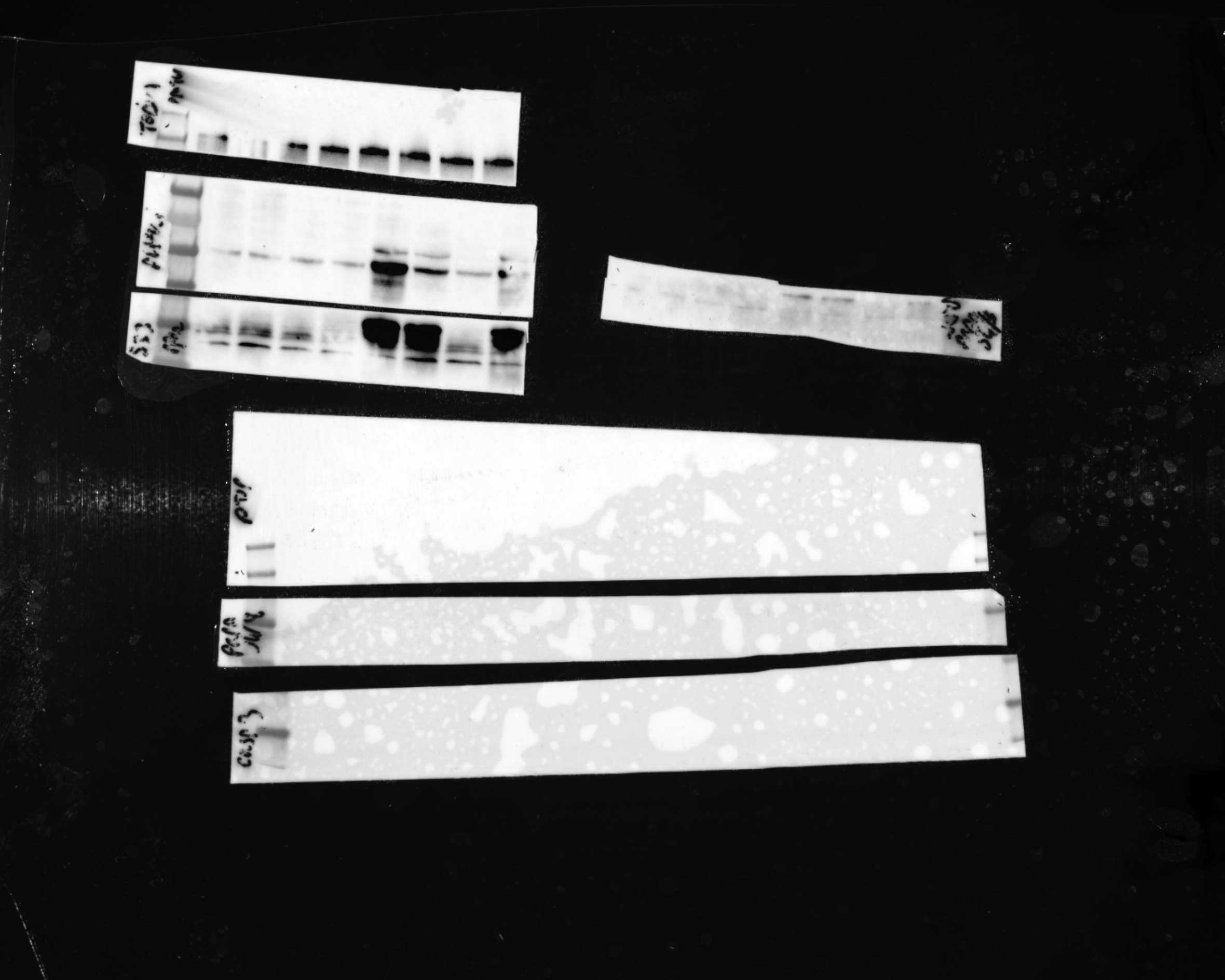

Supplement: Figure 2—figure supplement 3—source data 2. [file elife-106196-fig2-figsupp3-data2.zip › Figure 2-figure supplement 3A and 3B-Source Data 2/Figure 2-figure supplement 3B-Source Data2/colo_d2_max_lm366_laura m 2024-03-26 12h12m08s+lm366_pCHK1_laura m 2024-03-26 12h03m44s.tif]

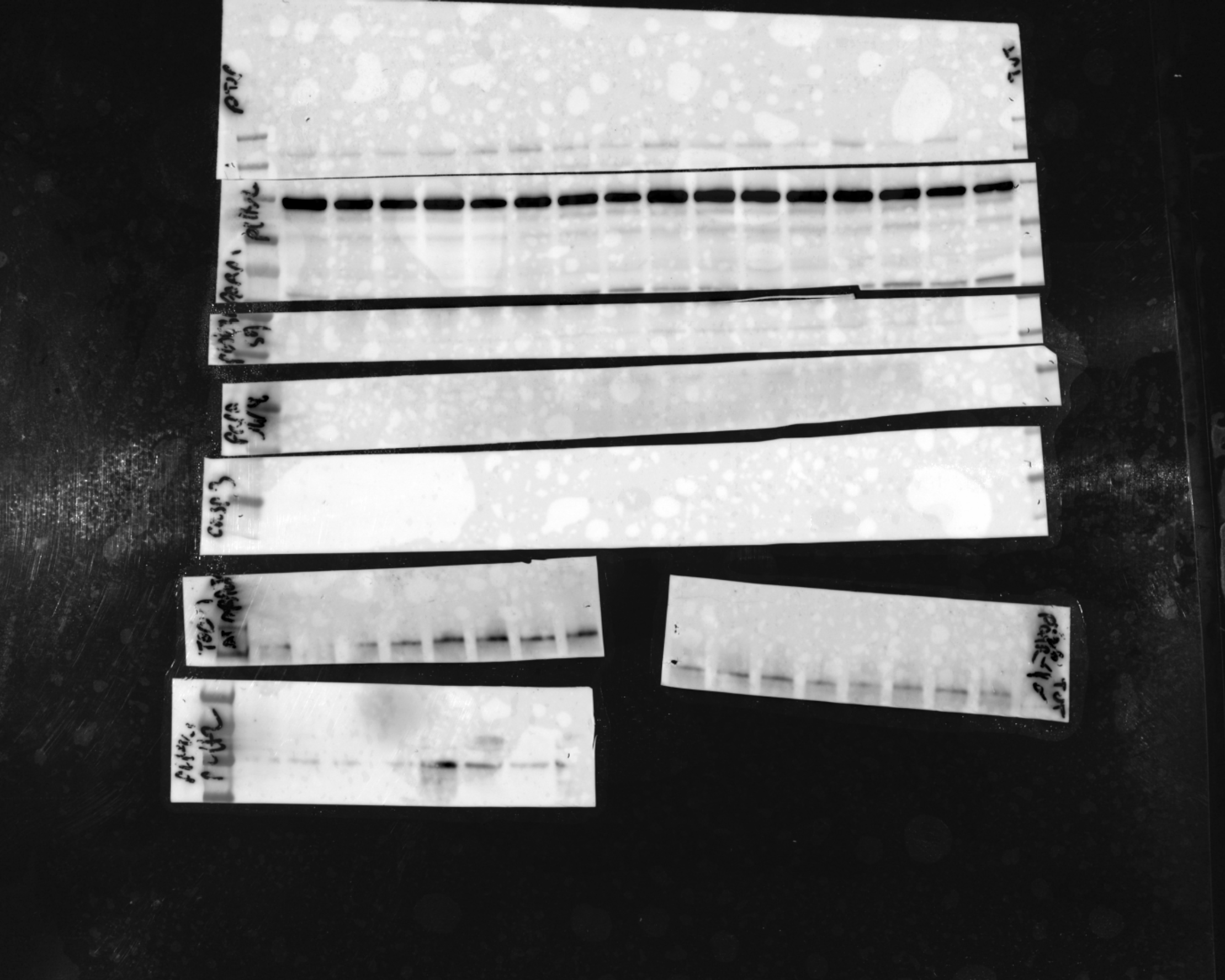

Supplement: Figure 2—figure supplement 3—source data 2. [file elife-106196-fig2-figsupp3-data2.zip › Figure 2-figure supplement 3A and 3B-Source Data 2/Figure 2-figure supplement 3B-Source Data2/lm366_TopBP1_laura m 2024-03-27 11h27m59s+colo Top m 2024-03-27 11h36m24s.tif]

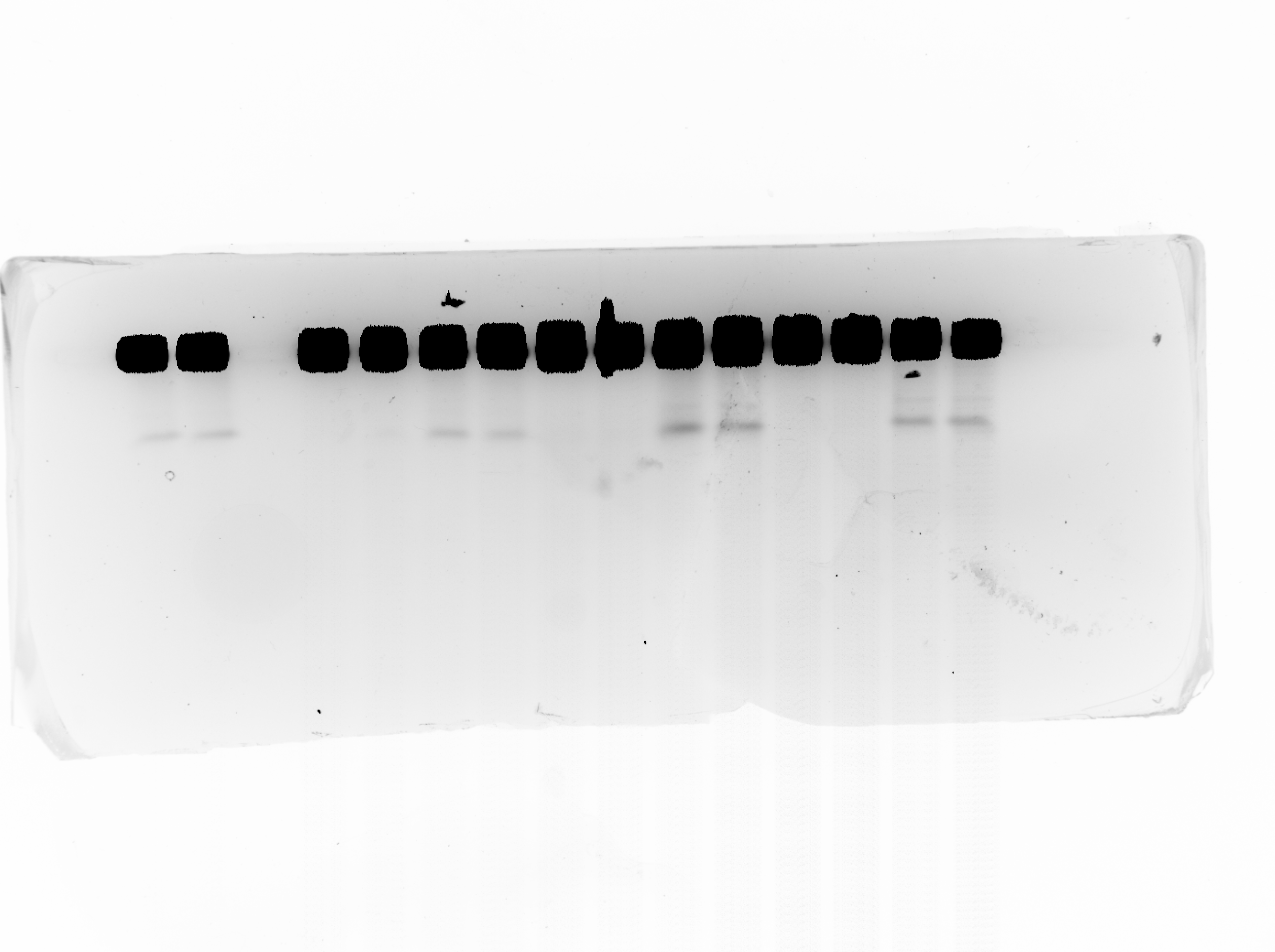

Supplement: Figure 3—source data 2. [file elife-106196-fig3-data2.zip › Fig 3D and E- Source Data 2/Raw data Fig 3D/Fig 3D Low exposure.tif]

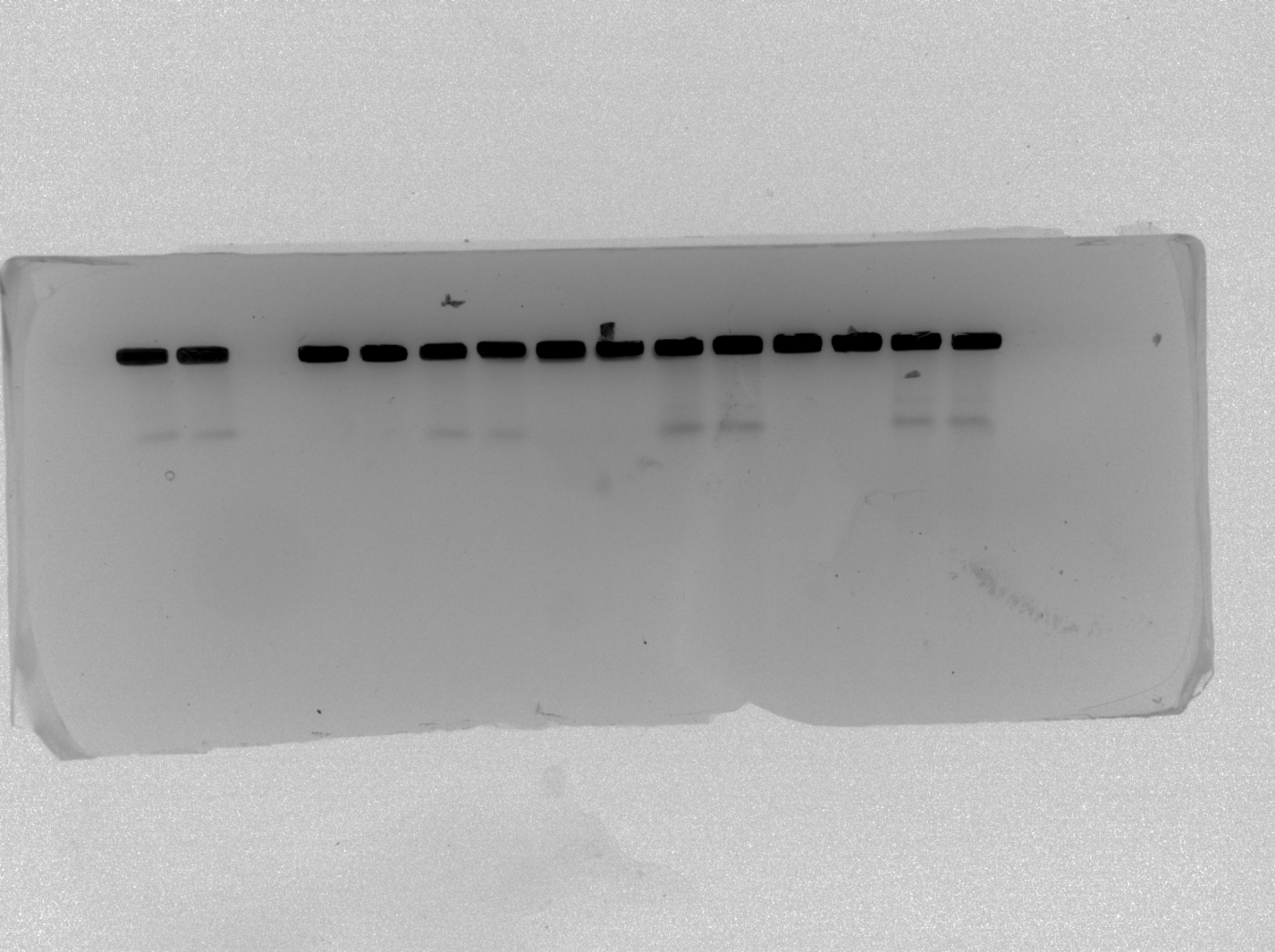

Supplement: Figure 3—source data 2. [file elife-106196-fig3-data2.zip › Fig 3D and E- Source Data 2/Raw data Fig 3D/Fig3D High exp.tif]

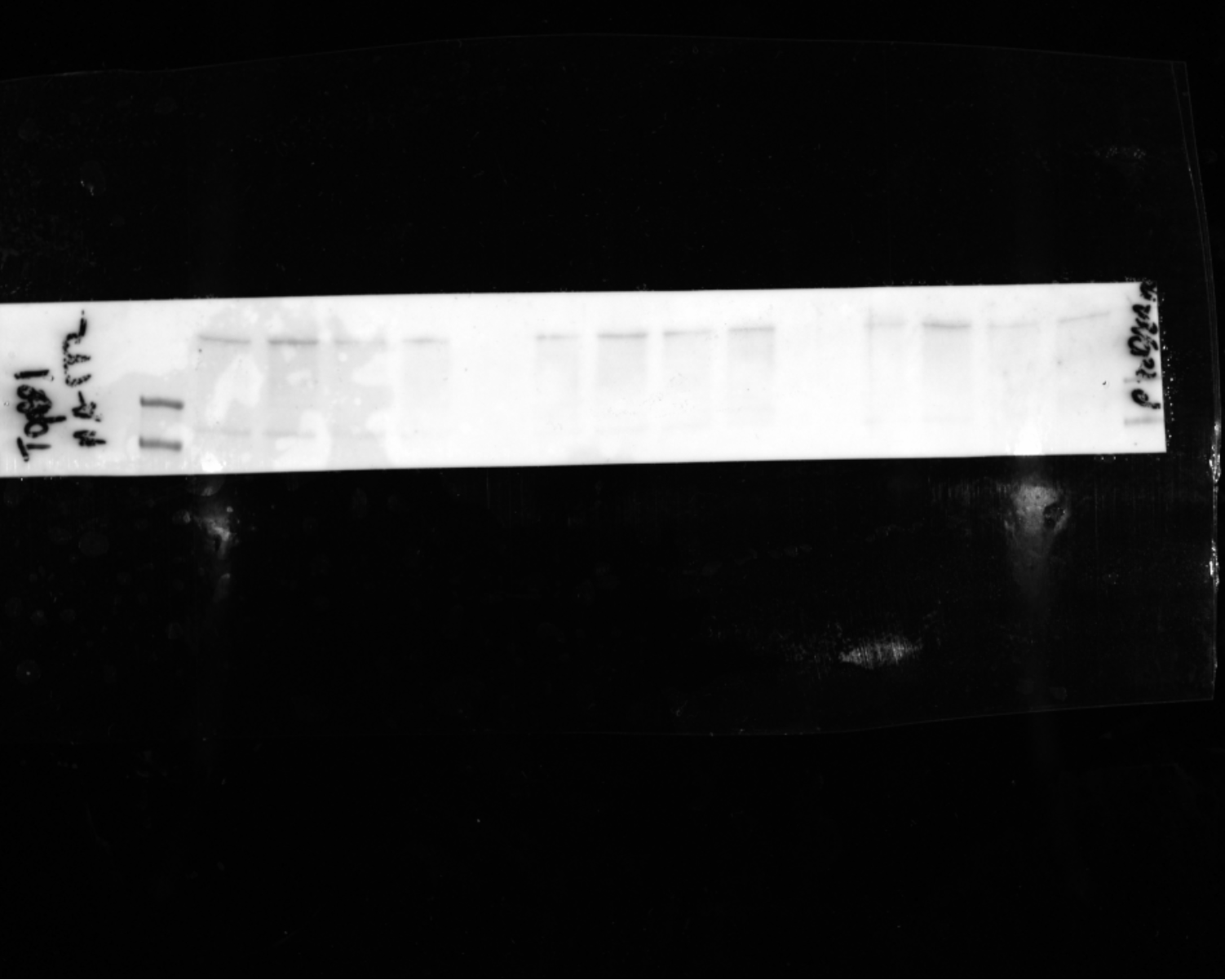

Supplement: Figure 3—source data 2. [file elife-106196-fig3-data2.zip › Fig 3D and E- Source Data 2/Raw data Fig 3E/lm353L1_DNAPK_laura m 2024-02-13 12h20m52s+colo_DNAPK_laura m 2024-02-13 12h22m20s.tif]

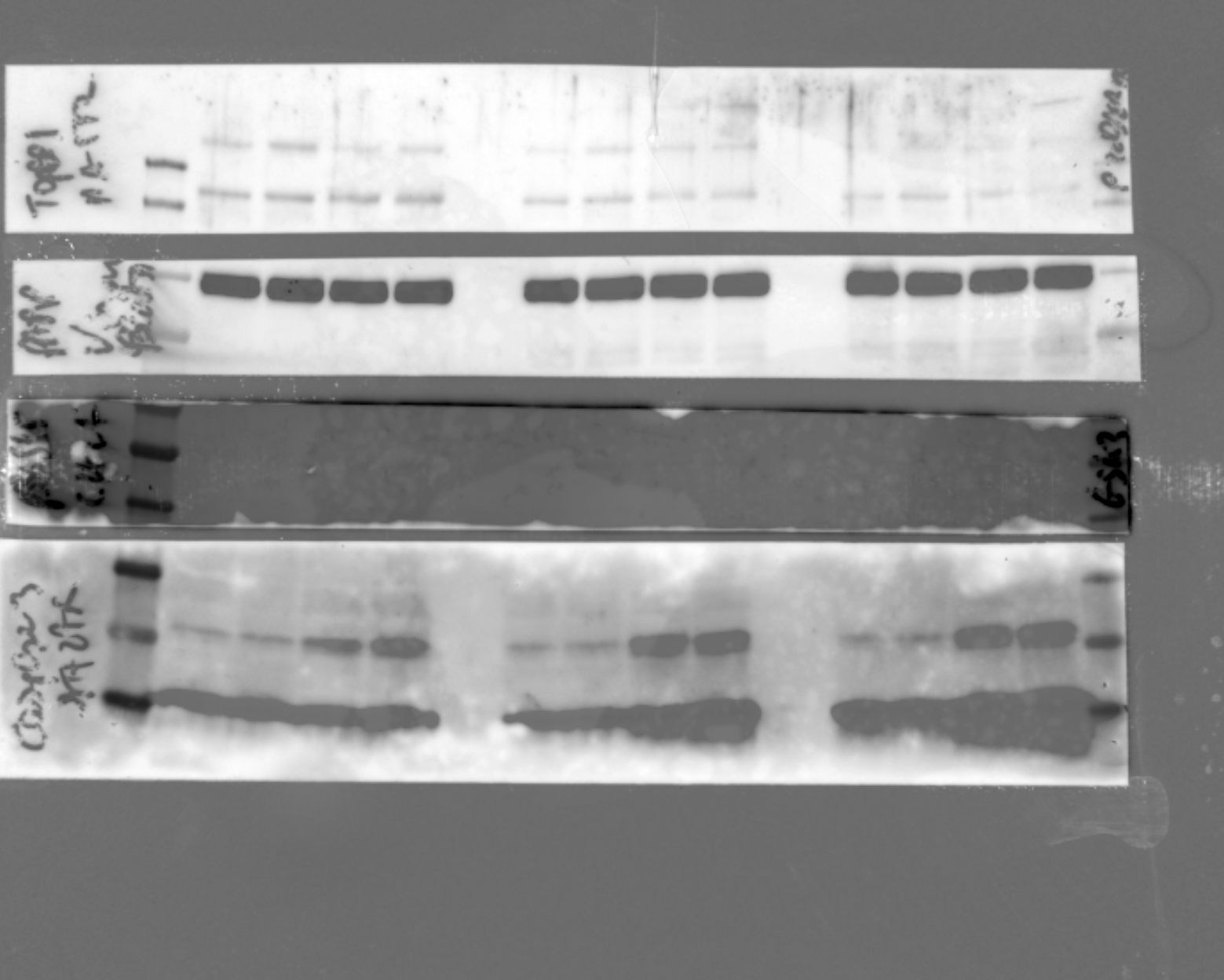

Supplement: Figure 3—source data 2. [file elife-106196-fig3-data2.zip › Fig 3D and E- Source Data 2/Raw data Fig 3E/lm353L1_pDNAPK_laura m 2024-02-10 15h33m16s+colo_pDNAPK_laura m 2024-02-10 15h57m12s.tif]

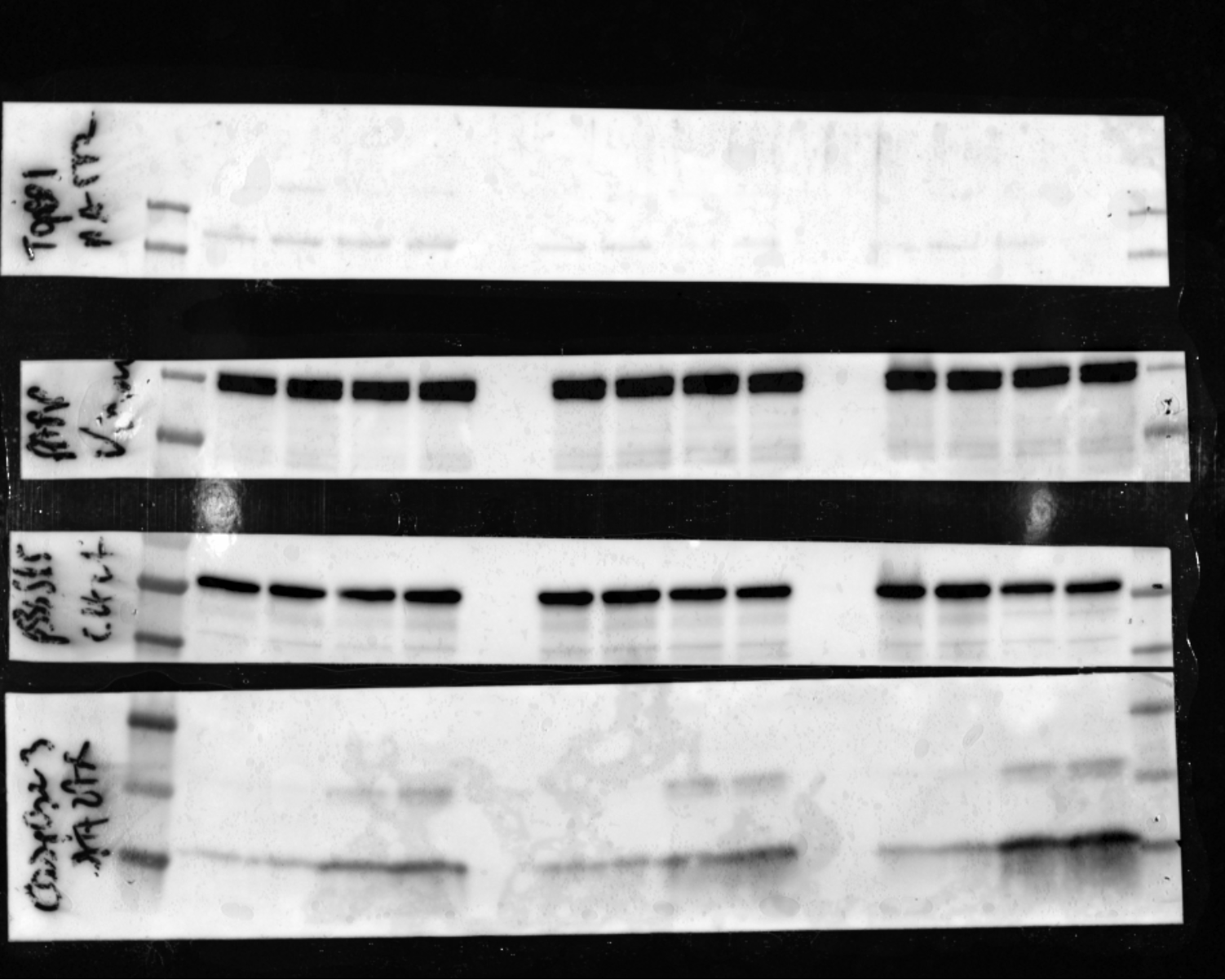

Supplement: Figure 3—source data 2. [file elife-106196-fig3-data2.zip › Fig 3D and E- Source Data 2/Raw data Fig 3E/lm353_gh2AX_vincu_laura m 2024-02-09 17h28m49s+colo_vincu_CHK1_gH2AX_laura m 2024-02-09 17h40m41s.tif]

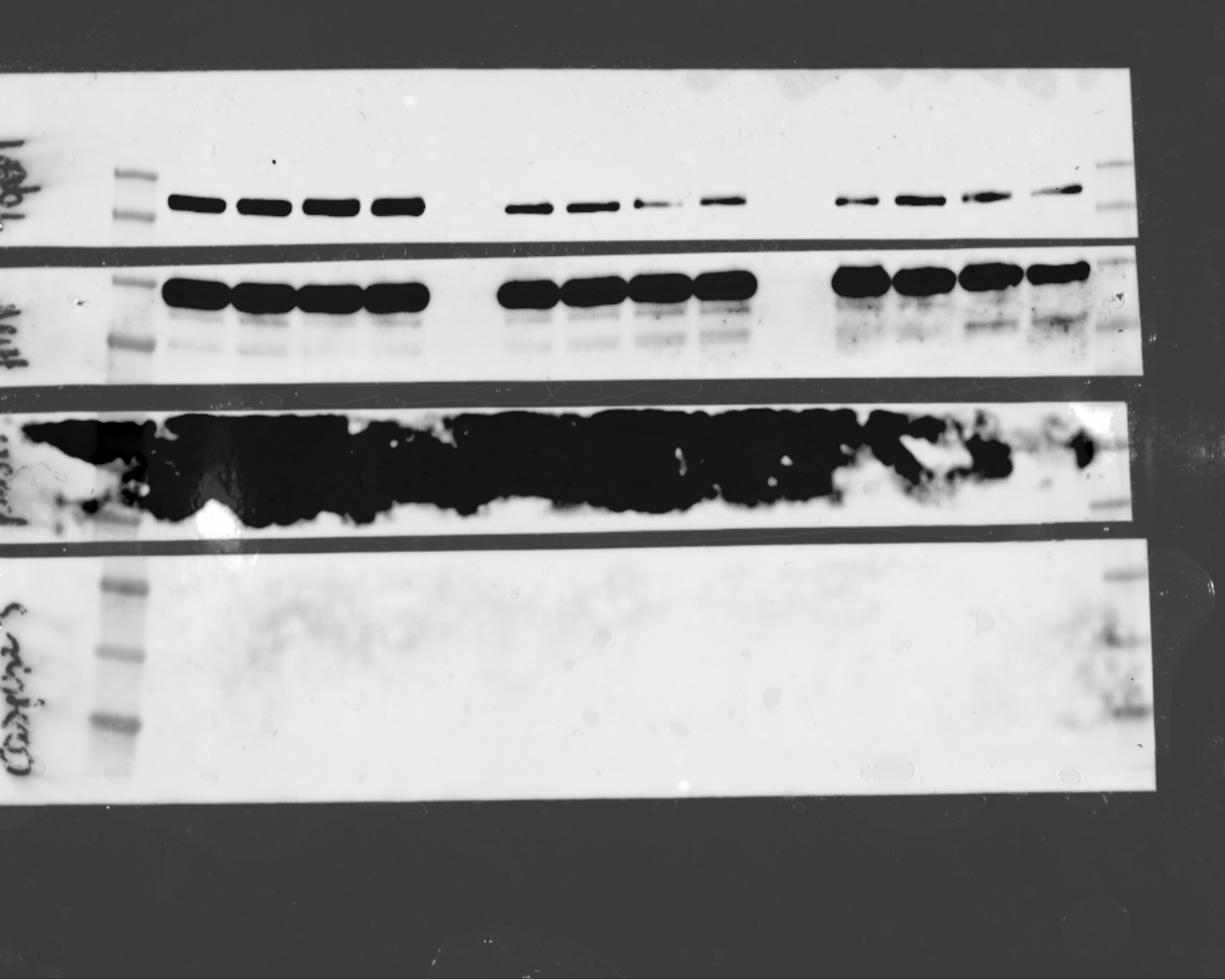

Supplement: Figure 3—source data 2. [file elife-106196-fig3-data2.zip › Fig 3D and E- Source Data 2/Raw data Fig 3E/lm353_PARPcleaved_laura m 2024-02-08 18h21m26s+colo_TOpBP1_PARP_laura m 2024-02-08 18h29m23s.tif]

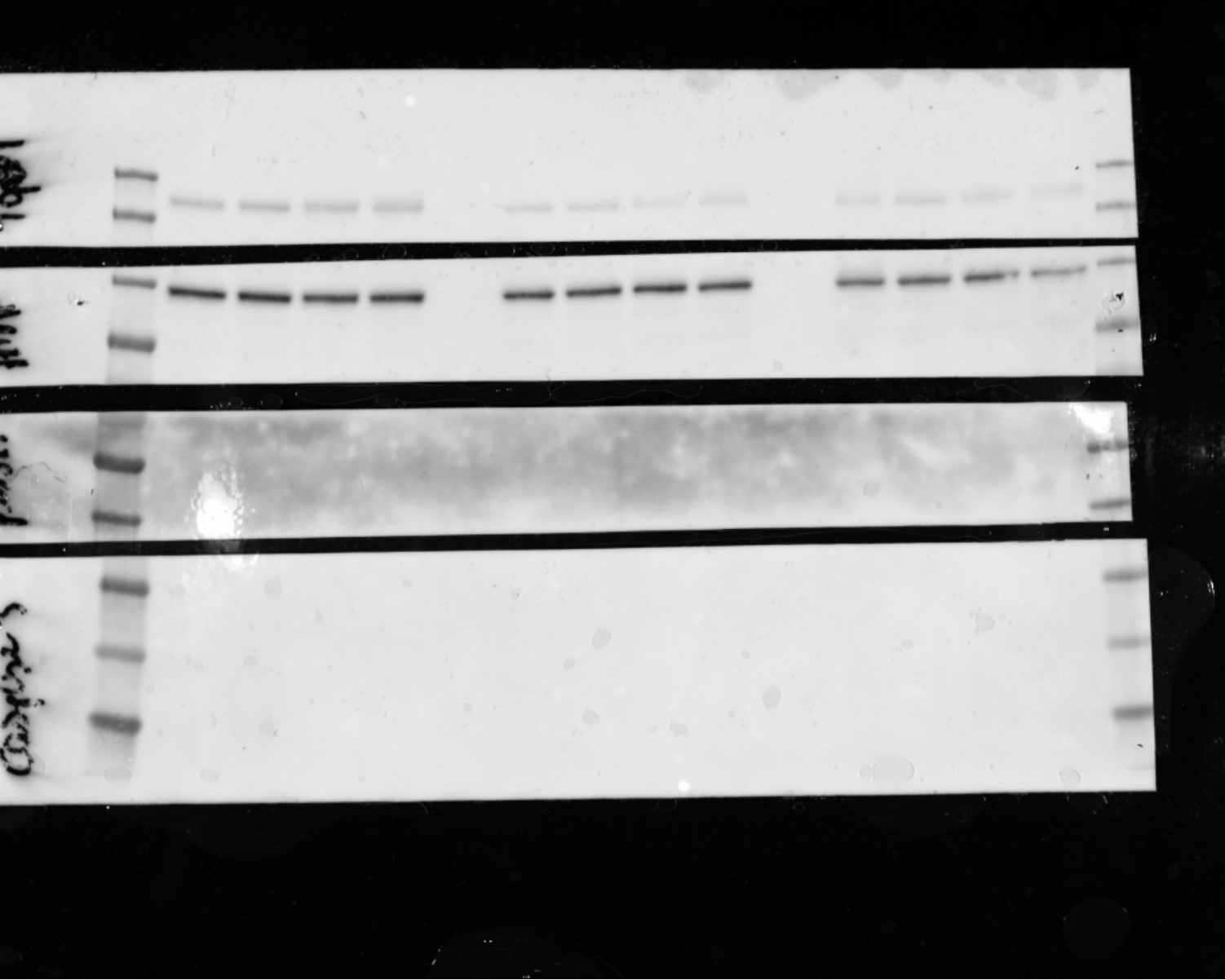

Supplement: Figure 3—source data 2. [file elife-106196-fig3-data2.zip › Fig 3D and E- Source Data 2/Raw data Fig 3E/lm353_PARP_laura m 2024-02-08 18h20m04s+colo_TOpBP1_PARP_laura m 2024-02-08 18h29m23s.tif]

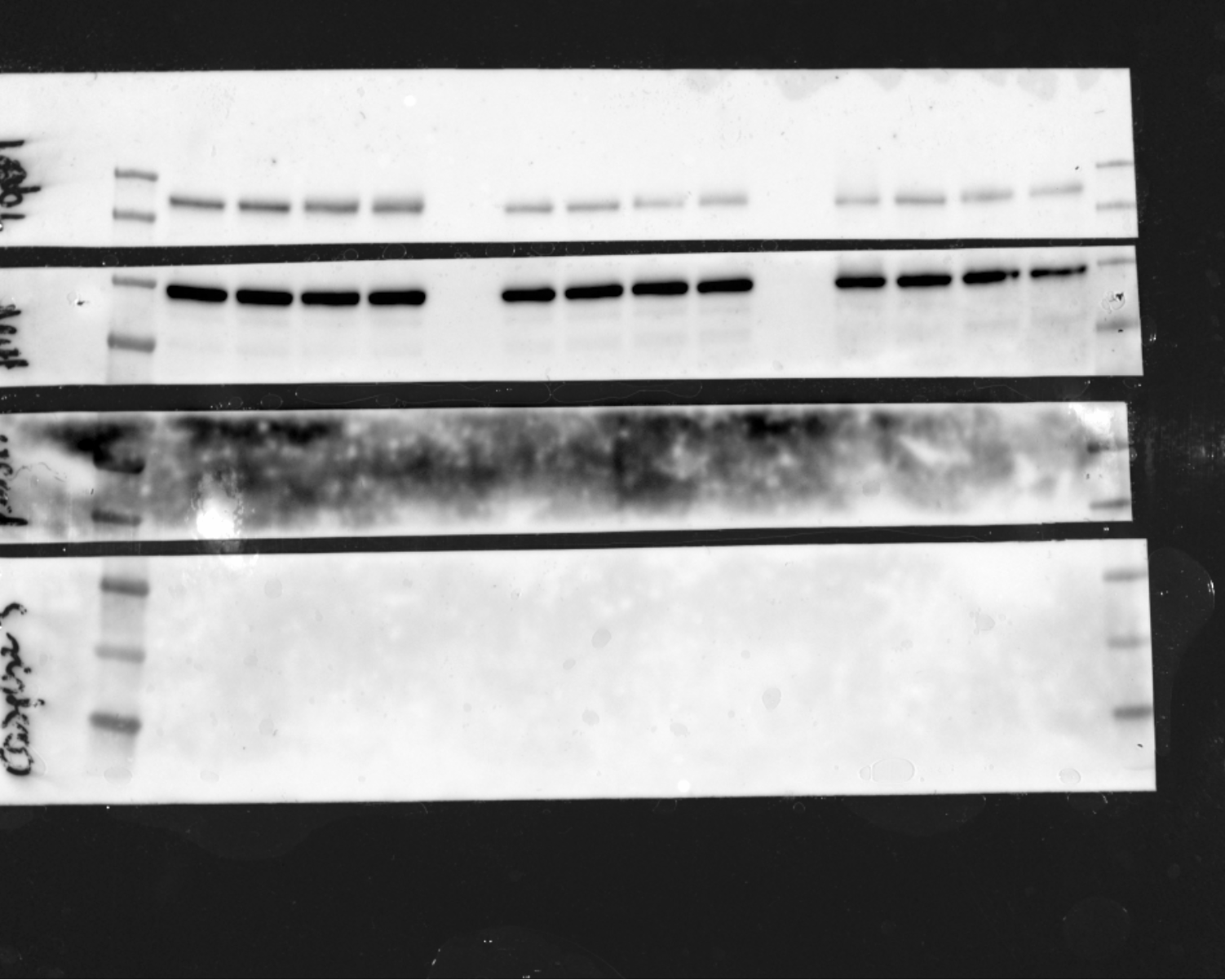

Supplement: Figure 3—source data 2. [file elife-106196-fig3-data2.zip › Fig 3D and E- Source Data 2/Raw data Fig 3E/lm353_TopBP1_laura m 2024-02-08 18h20m10s+colo_TOpBP1_PARP_laura m 2024-02-08 18h29m23s.tif]

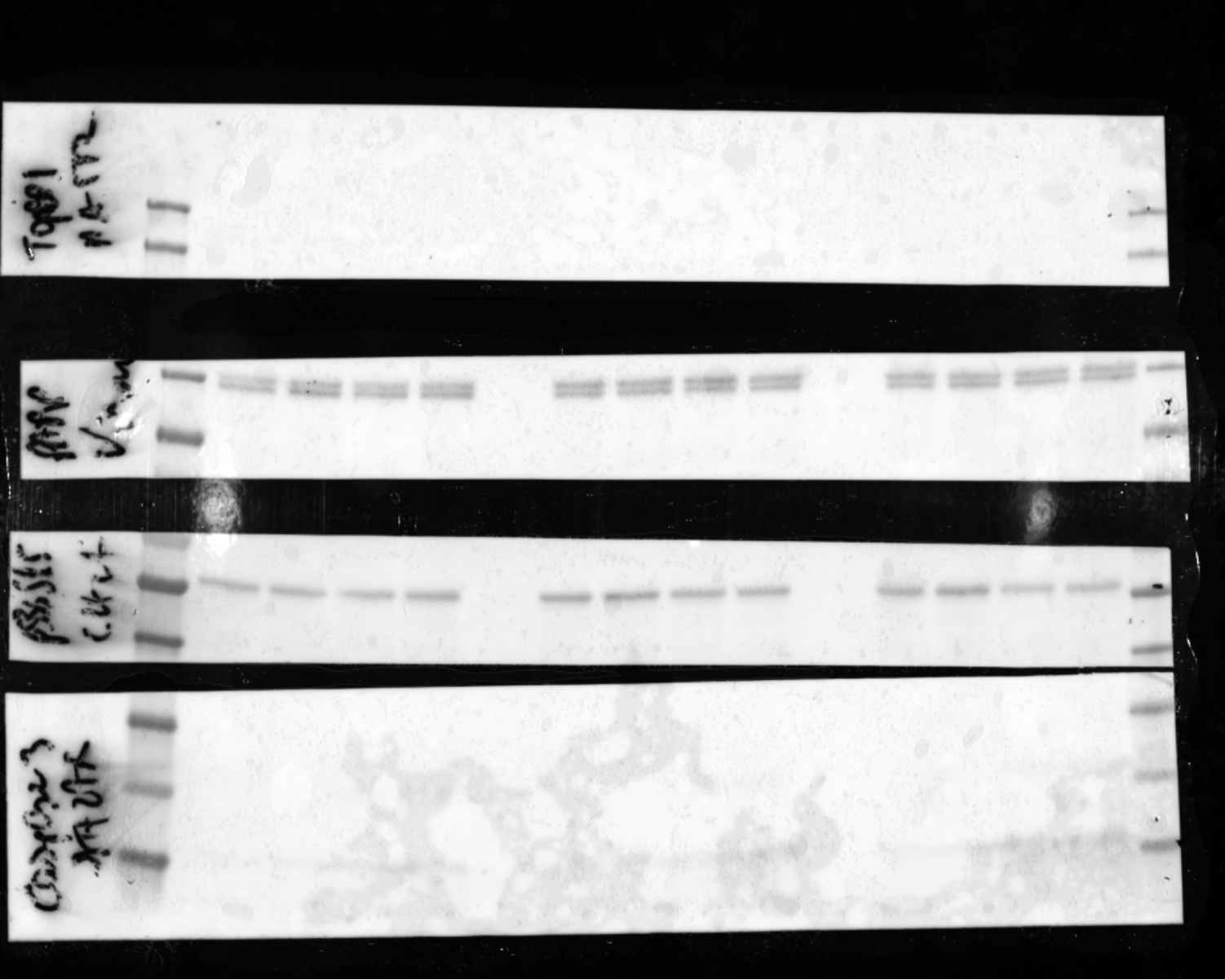

Supplement: Figure 3—source data 2. [file elife-106196-fig3-data2.zip › Fig 3D and E- Source Data 2/Raw data Fig 3E/lm353_Vincu_CHK1_laura m 2024-02-09 17h28m38s+colo_vincu_CHK1_gH2AX_laura m 2024-02-09 17h40m41s.tif]

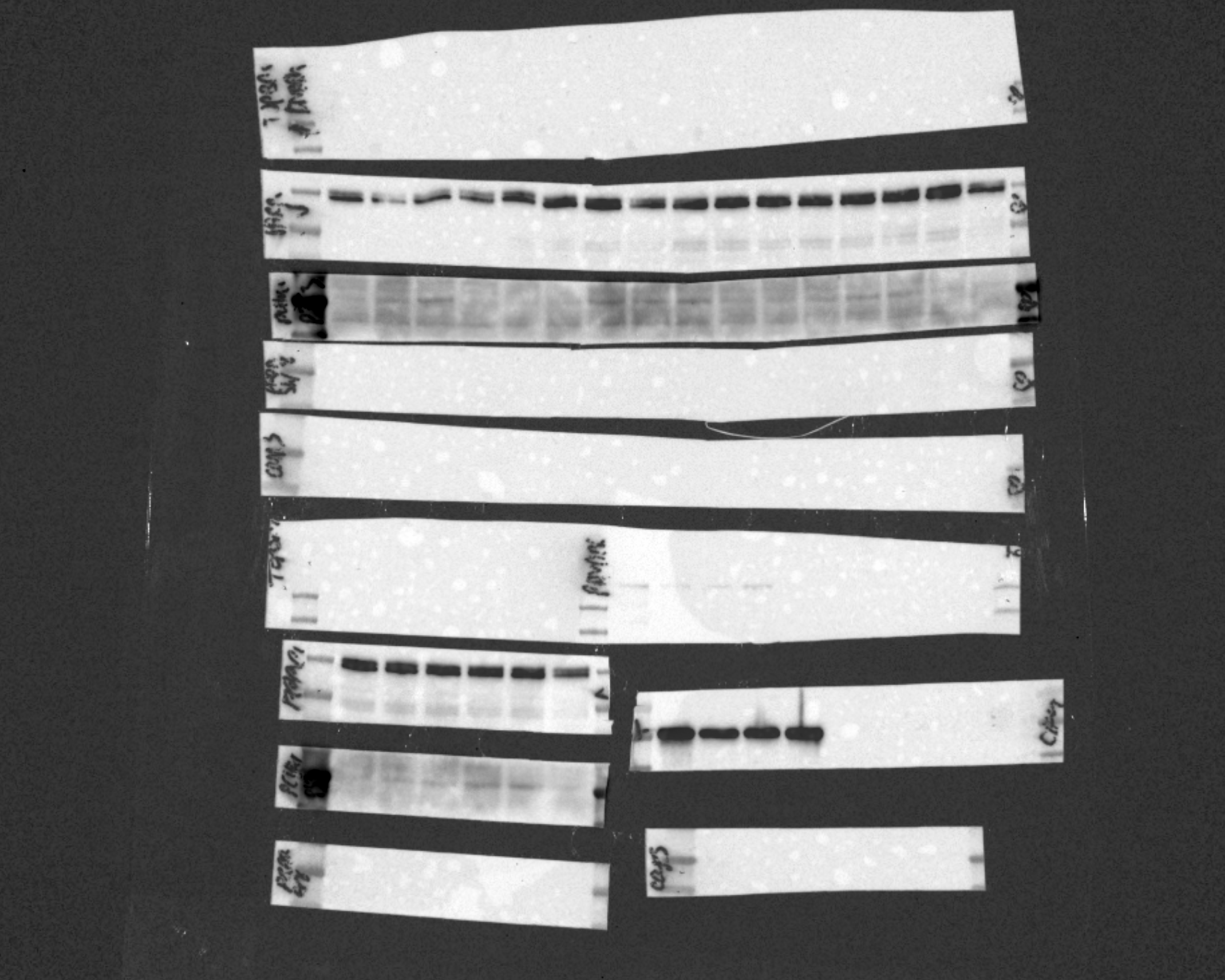

Supplement: Figure 4—source data 2. [file elife-106196-fig4-data2.zip › Fig 4C and D- Source Data 2/Fig 4C-Source Data 2/colo_vincu_laura m 2024-04-11 12h18m25s+lm373_lm377_vincu_laura m 2024-04-11 12h11m05s.tif]

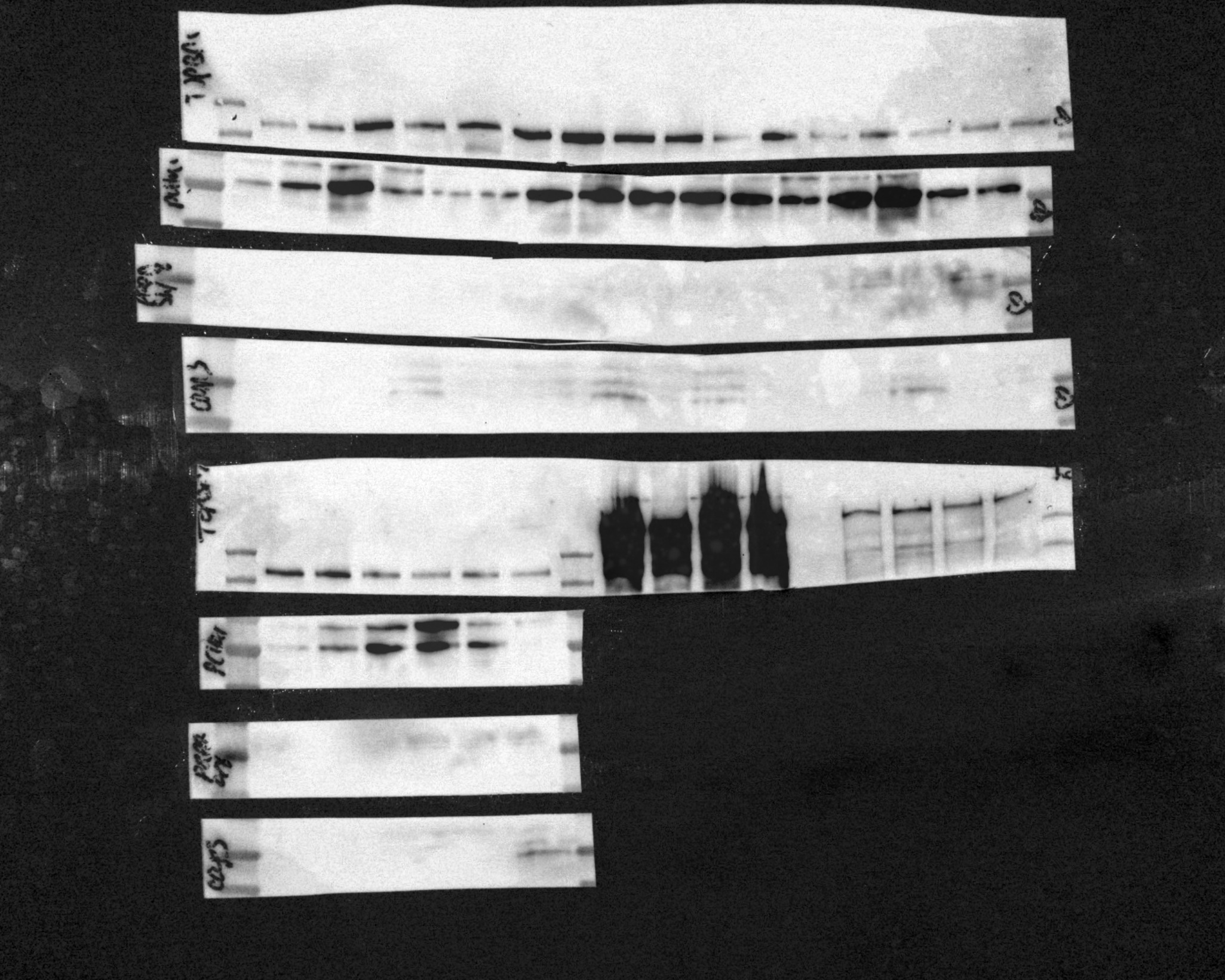

Supplement: Figure 4—source data 2. [file elife-106196-fig4-data2.zip › Fig 4C and D- Source Data 2/Fig 4C-Source Data 2/lm373_lm377_cleavedcasp3laura m 2024-04-10 11h12m27s+colo_casp3TopBP1_laura m 2024-04-10 11h17m00s.tif]

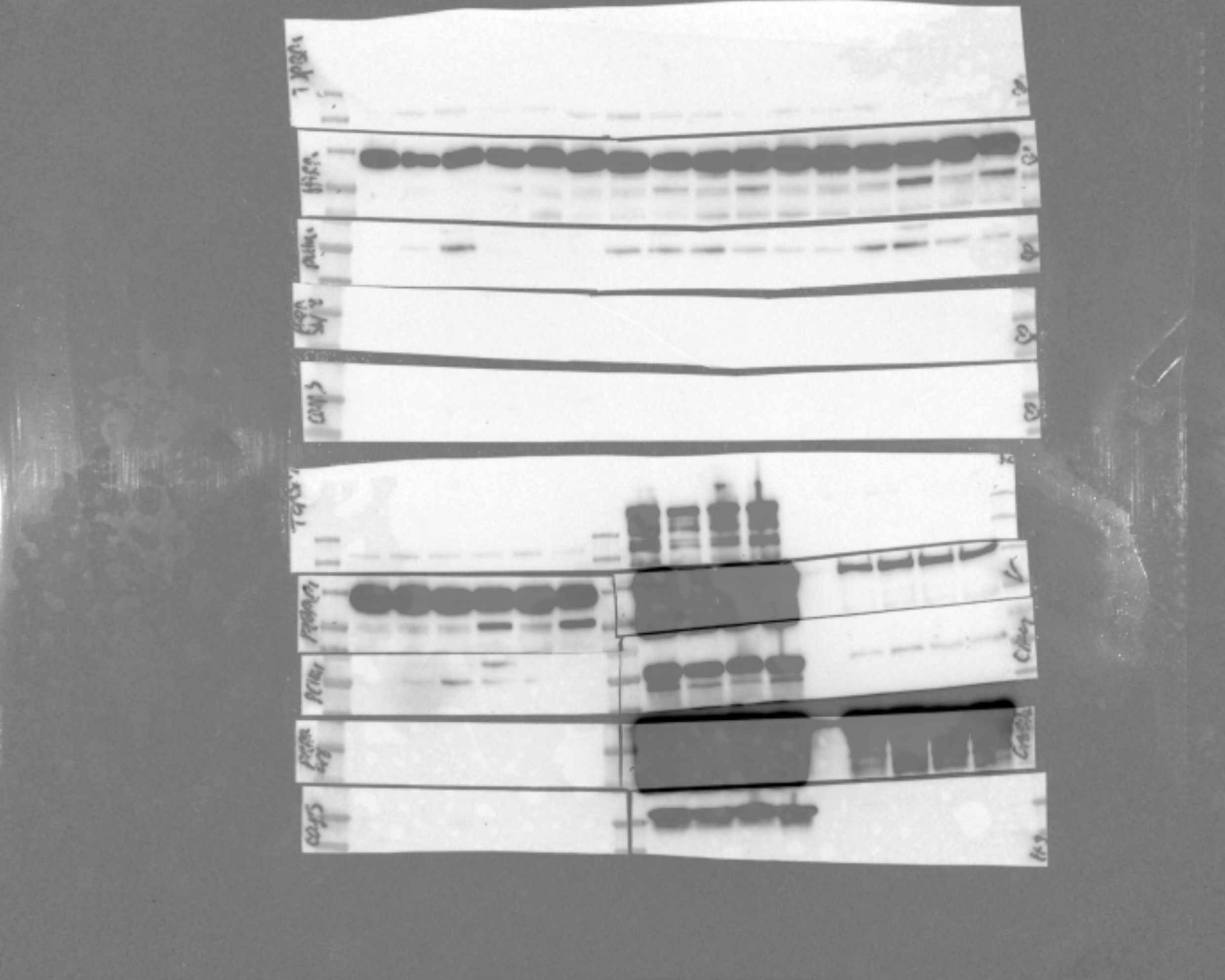

Supplement: Figure 4—source data 2. [file elife-106196-fig4-data2.zip › Fig 4C and D- Source Data 2/Fig 4C-Source Data 2/lm373_lm377_PARP1cleaved_laura m 2024-04-10 10h33m11s+colo_PARP_laura m 2024-04-10 10h47m03s.tif]

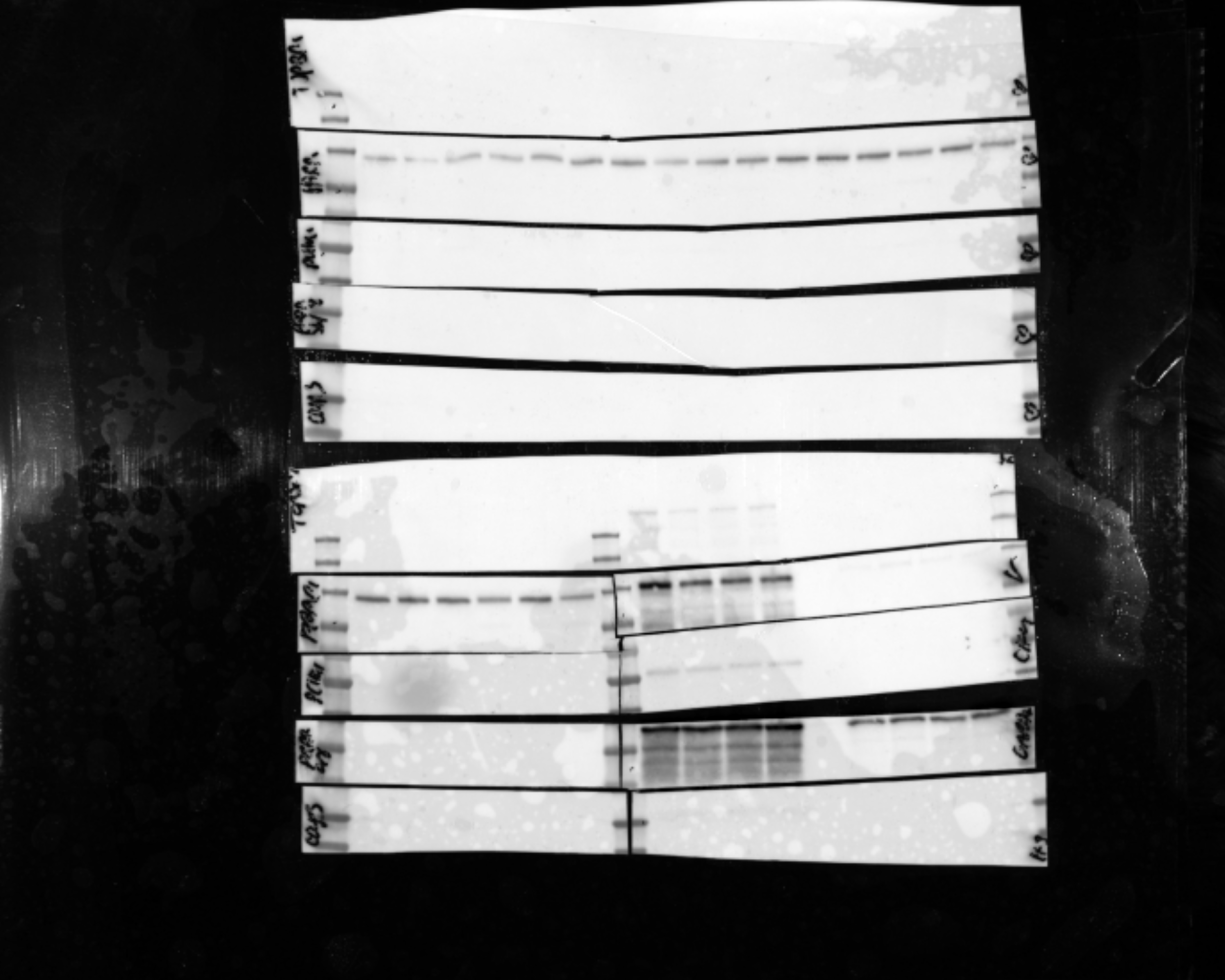

Supplement: Figure 4—source data 2. [file elife-106196-fig4-data2.zip › Fig 4C and D- Source Data 2/Fig 4C-Source Data 2/lm373_lm377_PARP_laura m 2024-04-10 10h30m36s+colo_PARP_laura m 2024-04-10 10h47m03s.tif]

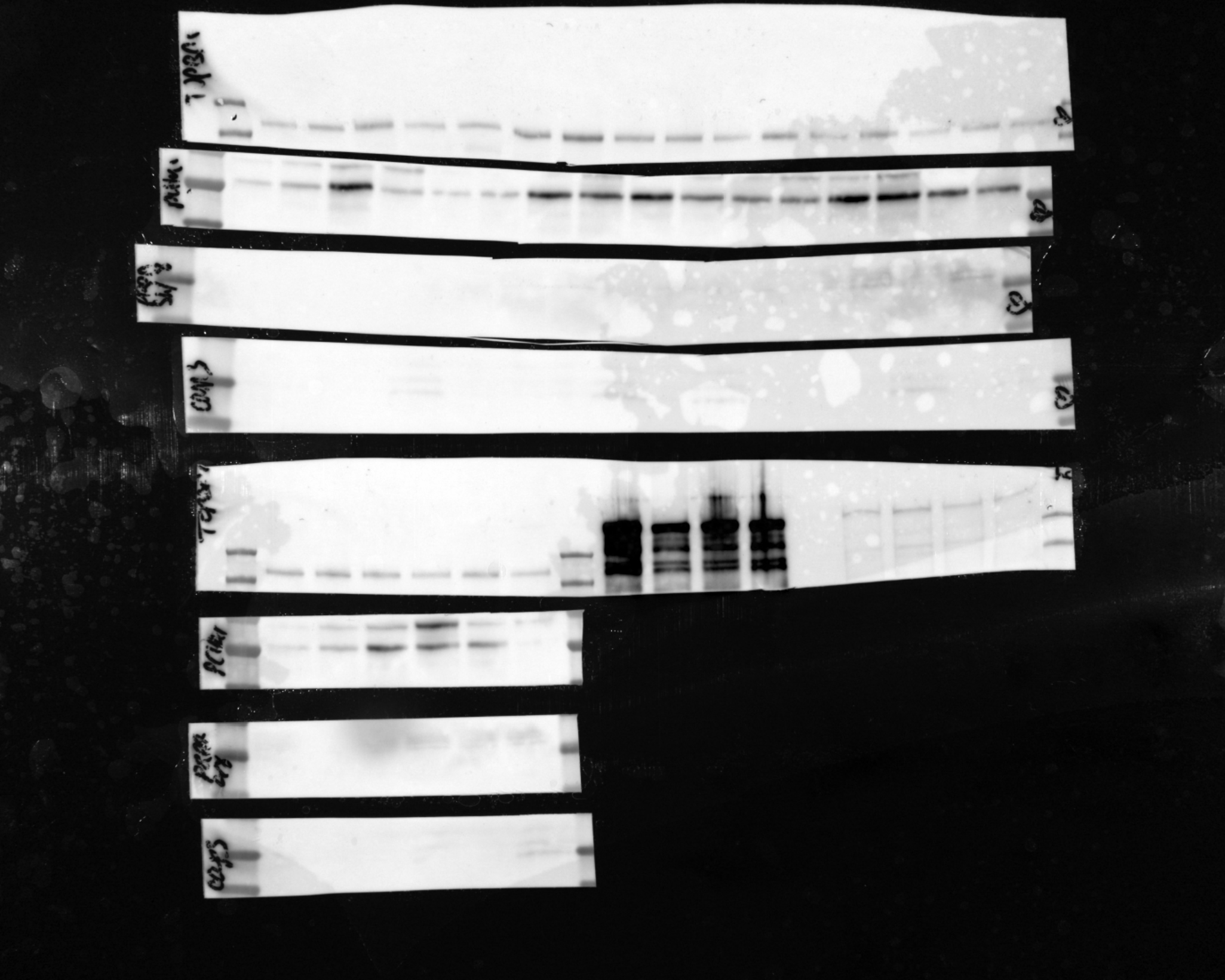

Supplement: Figure 4—source data 2. [file elife-106196-fig4-data2.zip › Fig 4C and D- Source Data 2/Fig 4C-Source Data 2/lm373_lm377_TopBP1_laura m 2024-04-10 11h04m35s+colo_casp3TopBP1_laura m 2024-04-10 11h17m00s.tif]

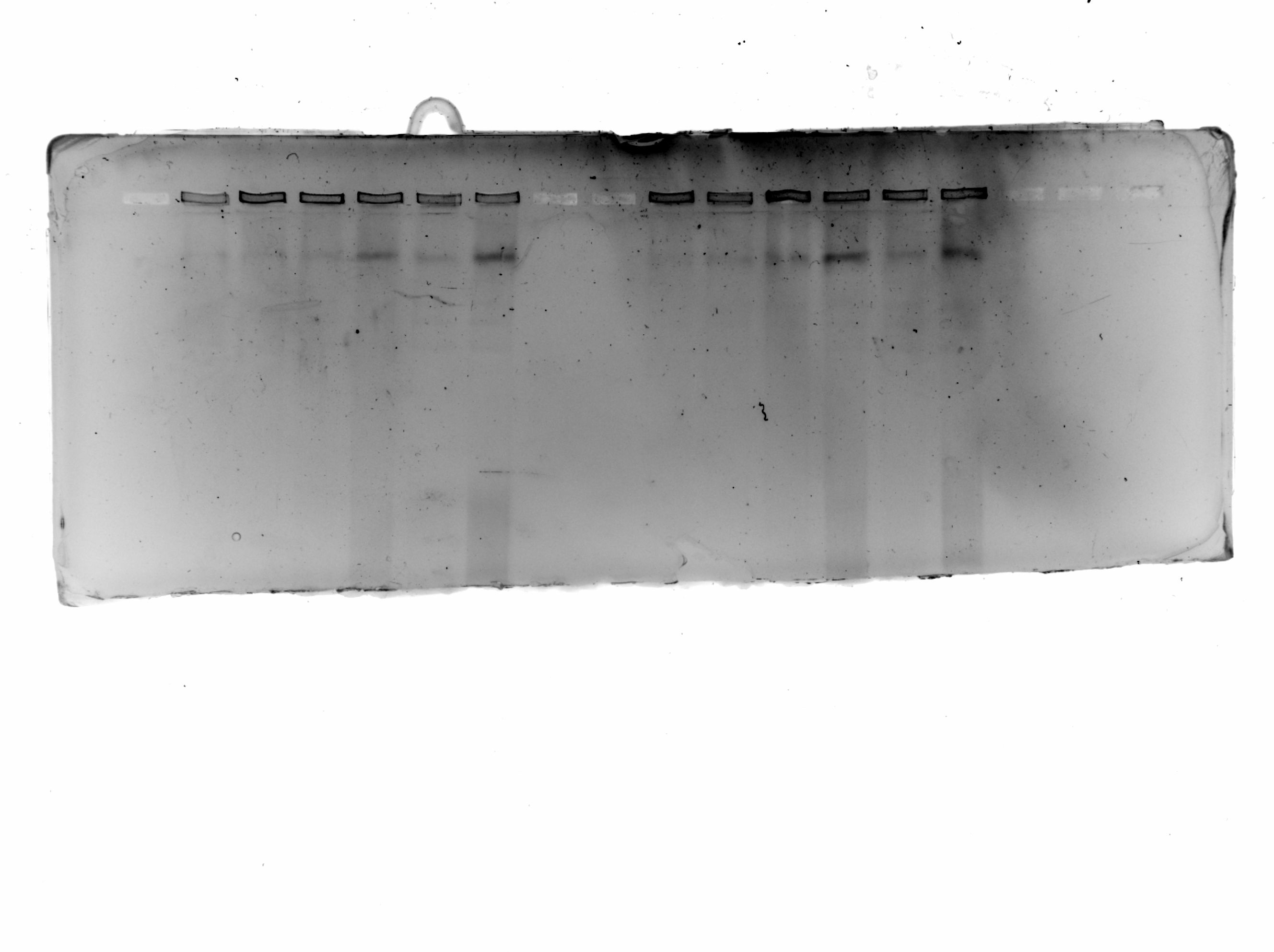

Supplement: Figure 4—source data 2. [file elife-106196-fig4-data2.zip › Fig 4C and D- Source Data 2/Fig 4D-Source Data 2/selected_3s_commun igh 2024-04-12 17hr 41min.tif]
