# Supplementary material for: TopBP1 biomolecular condensates as a new therapeutic target in advanced-stage colorectal cancer
Source: eLife. 2025 Oct 21;14:RP106196. doi: 10.7554/eLife.106196 (PMC12539802; doi:10.7554/eLife.106196)

Figure 2-figure supplement 3A

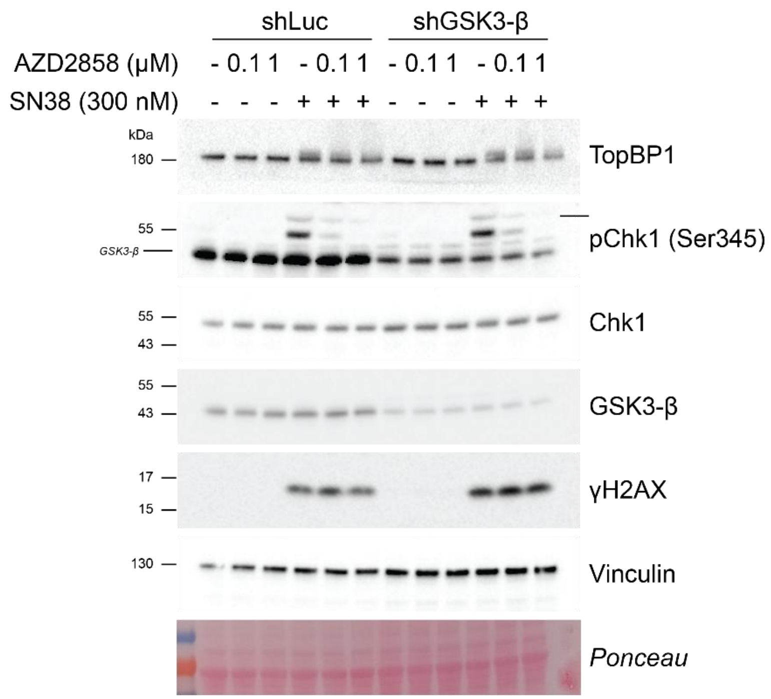

Figure 2-figure supplement 3A: Below are the Original membranes corresponding to Figure 2-figure supplement 3 Immunoblot of the indicated proteins after incubation of SW620 cells that express shLuc or shGSK-3β with AZD2858 (100 nM) or/and SN-38 (300 nM) for 2 h.

Merge chemiluminescence bands/colorimetric for pChk1

pChk1(S345)

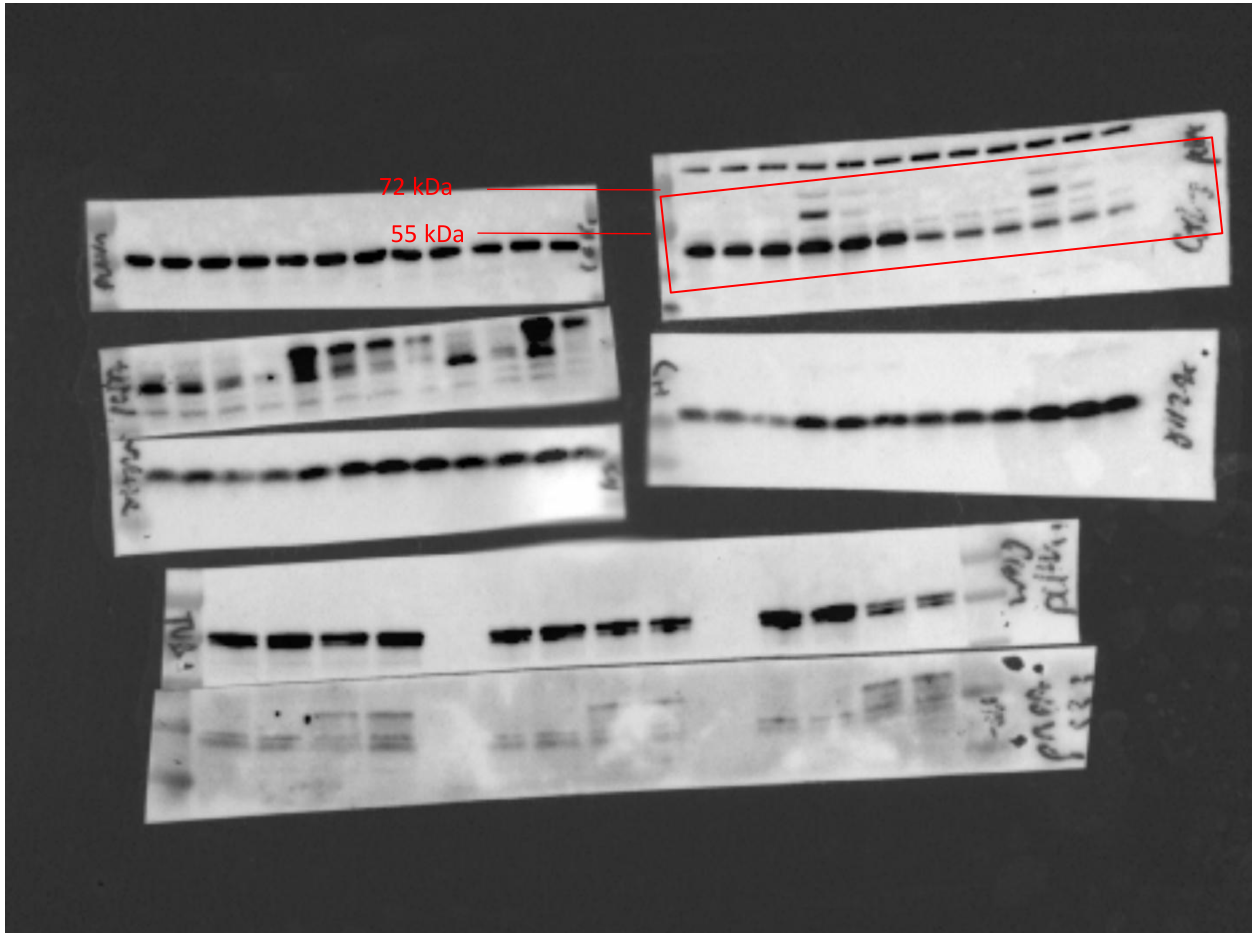

Chemiluminescence bands for pChk1

pChk1(S345)

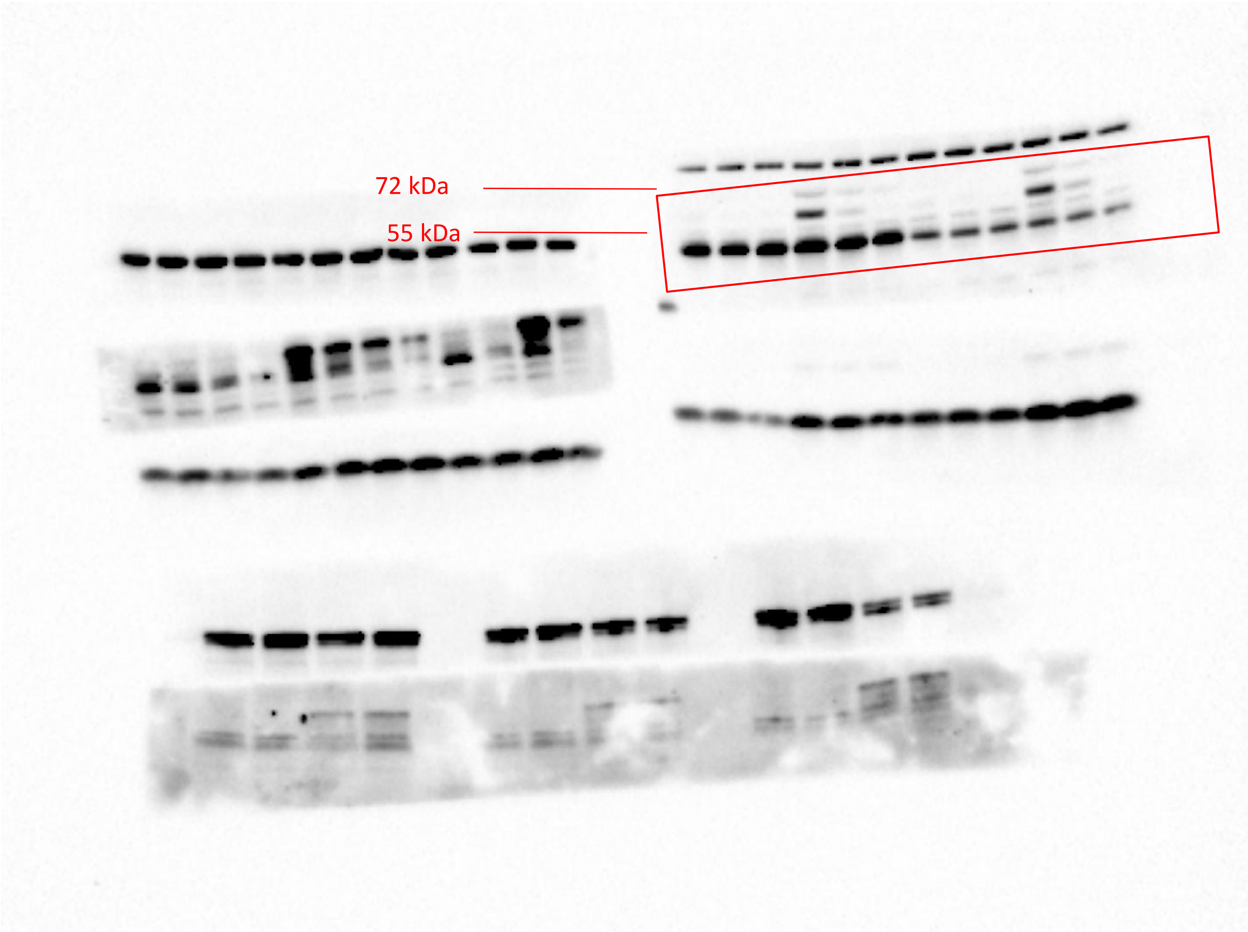

Colorimetric for pChk1

pChk1(S345)

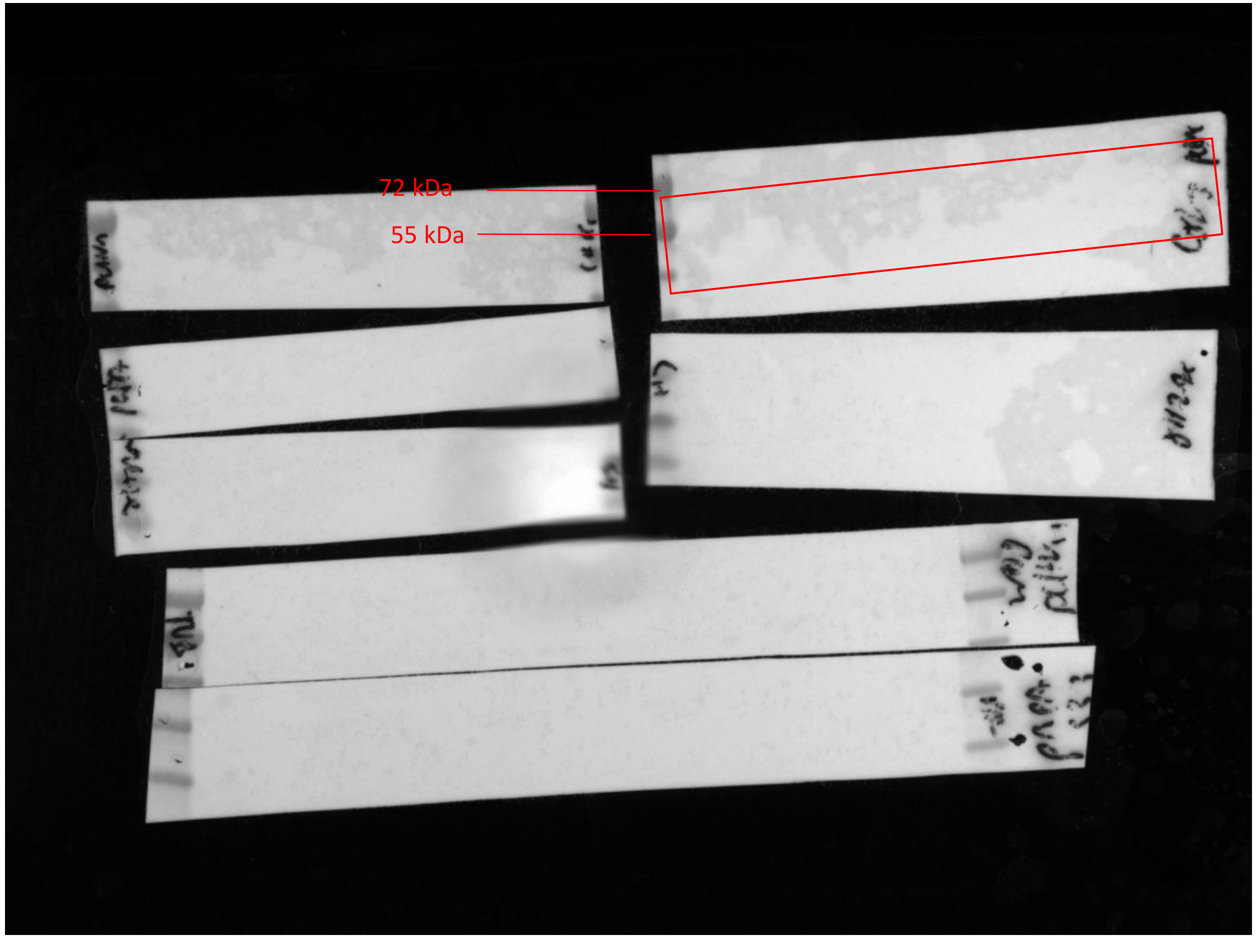

Merge chemiluminescence bands/colorimetric for Chk1

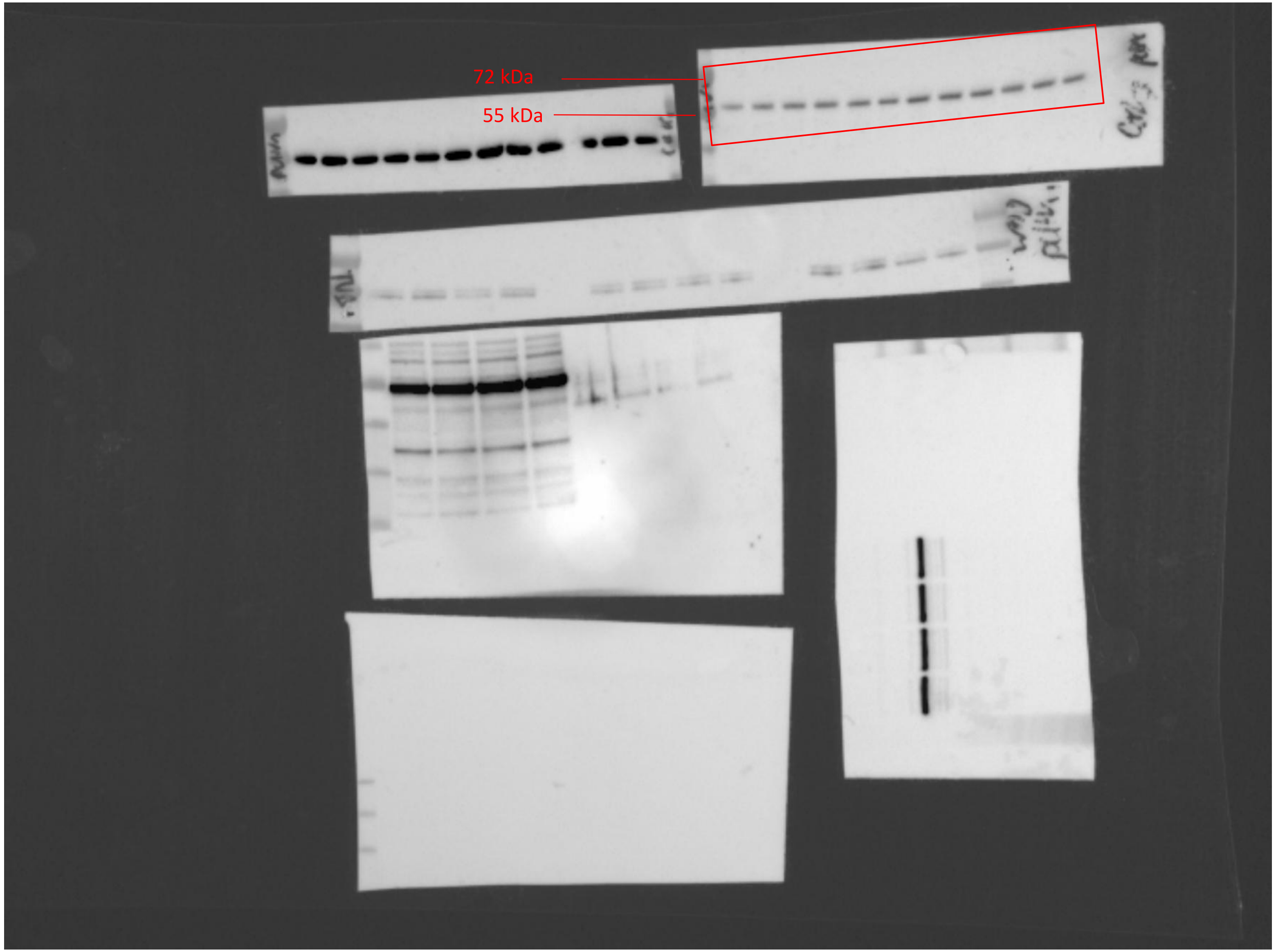

Chk1

chemiluminescence for Chk1

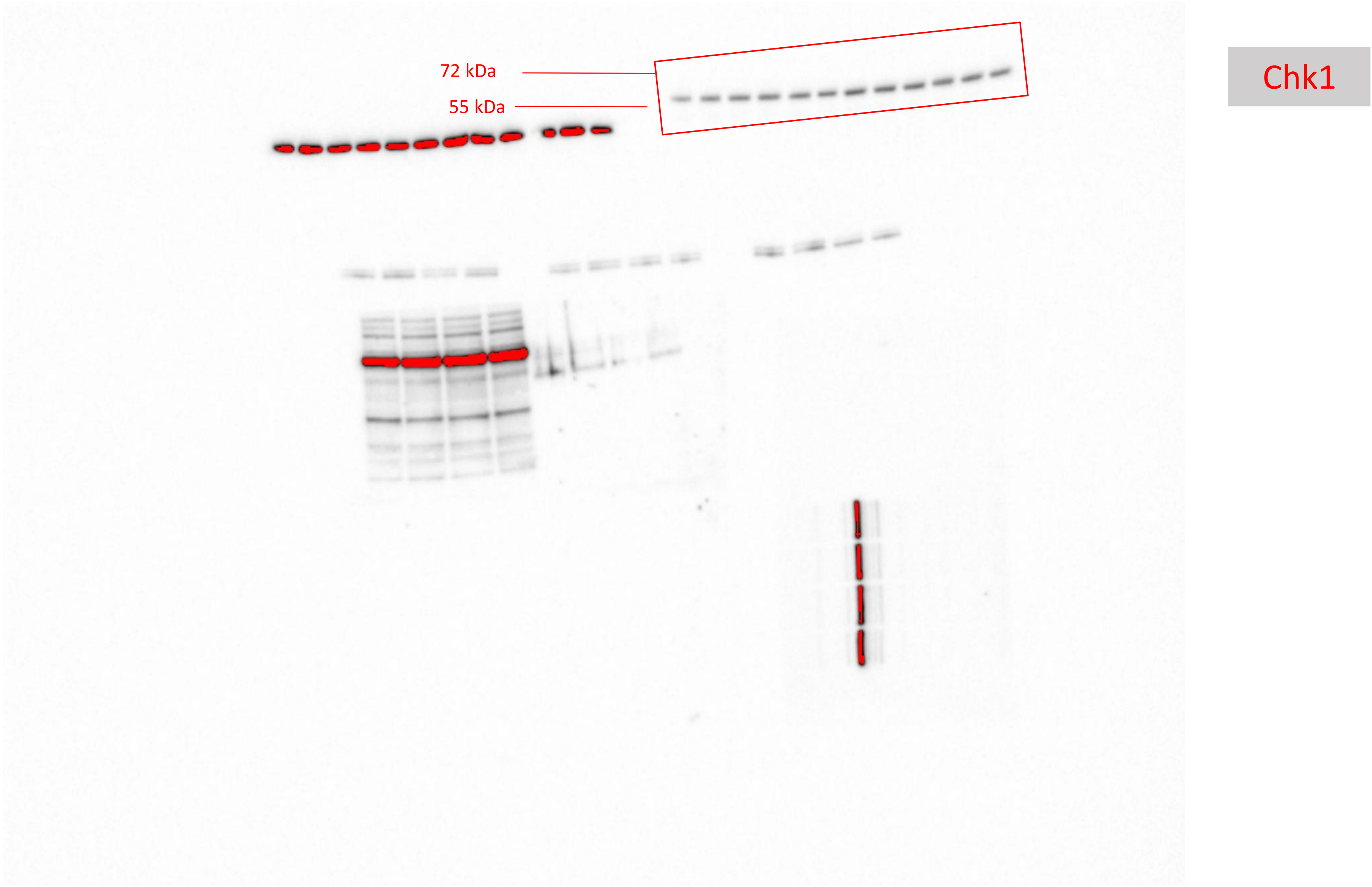

Merge chemiluminescence bands/colorimetric for Chk1

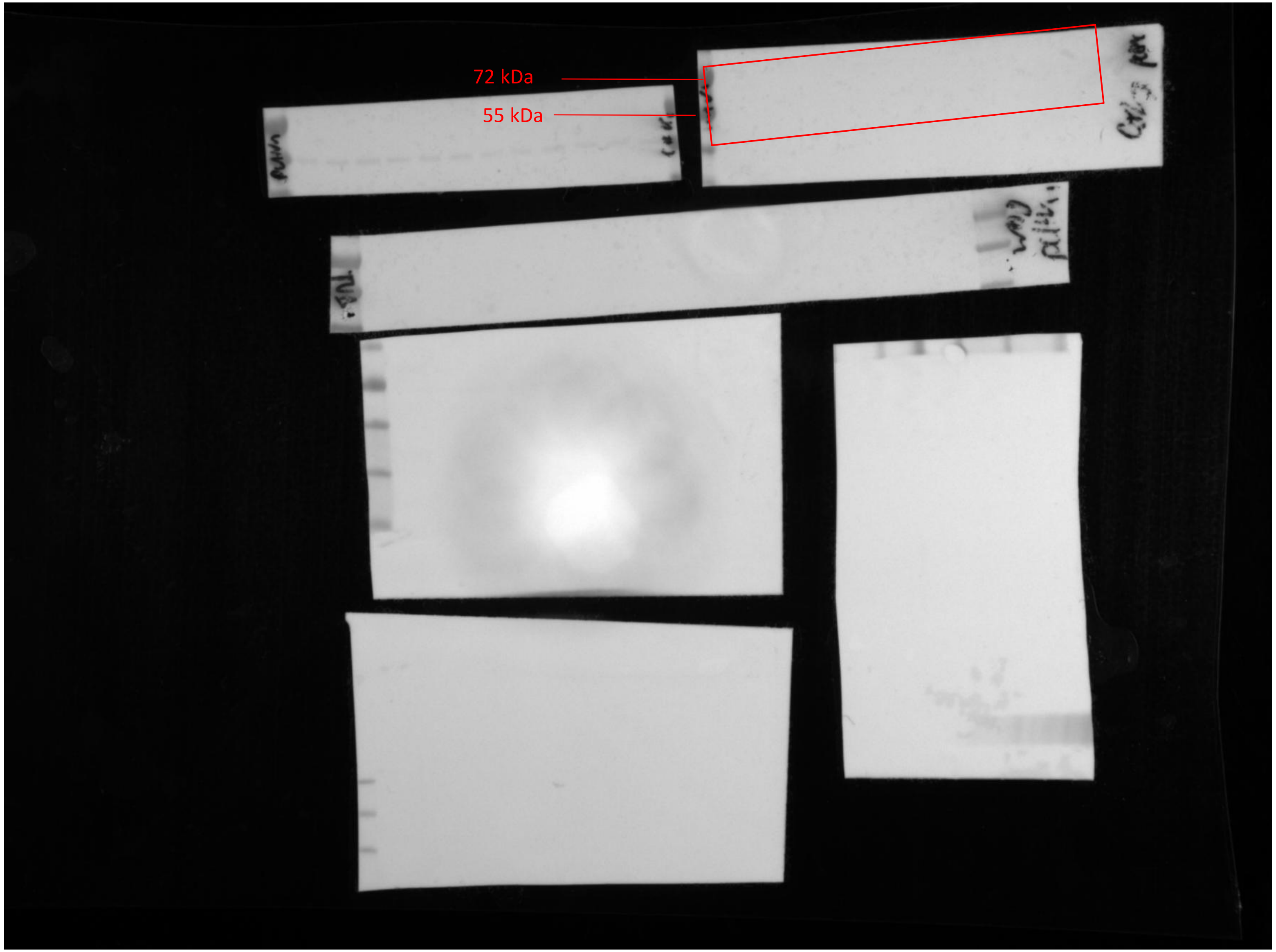

Merge chemiluminescence bands/colorimetric for TopBP1

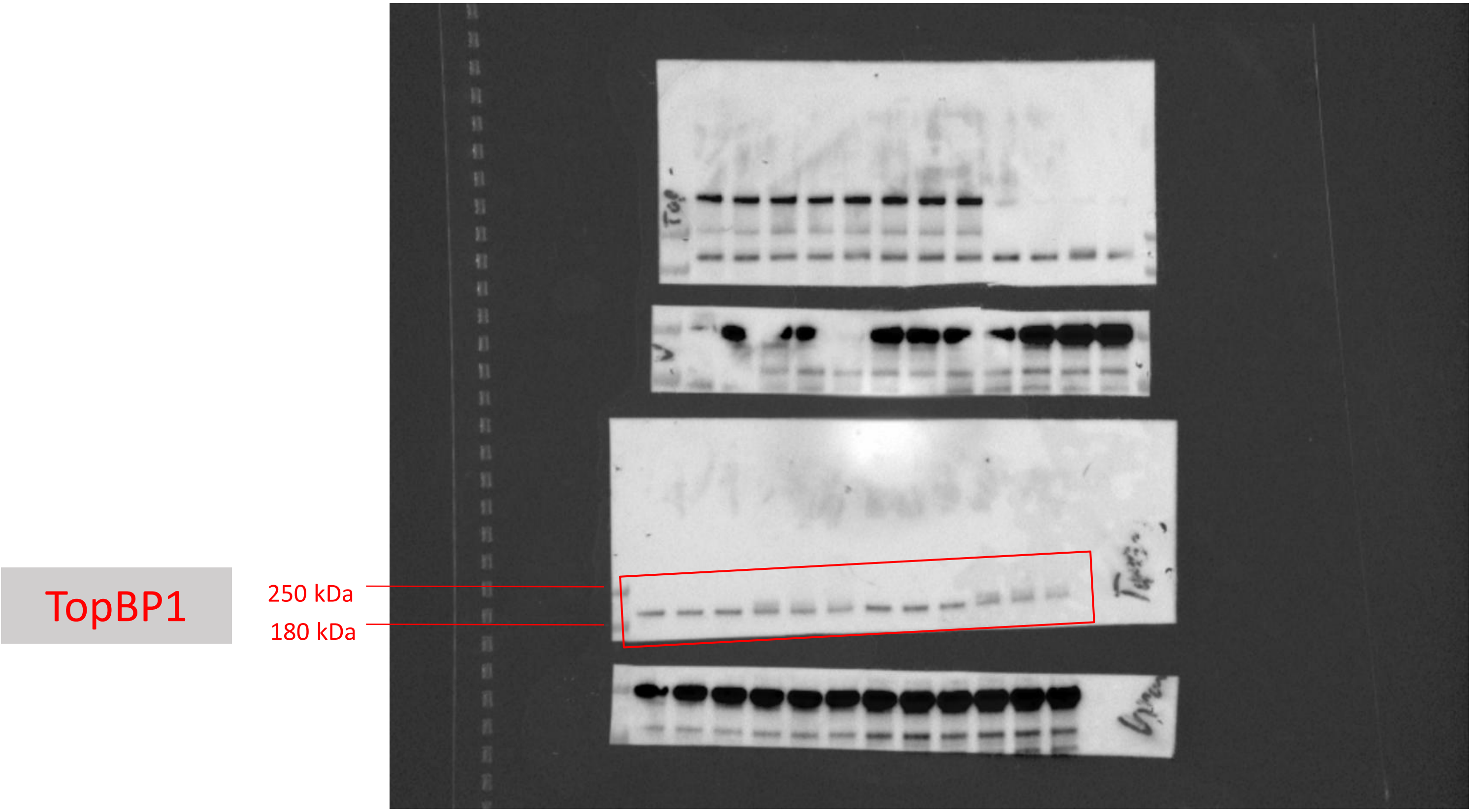

Chemiluminescence bands for ToPBP1

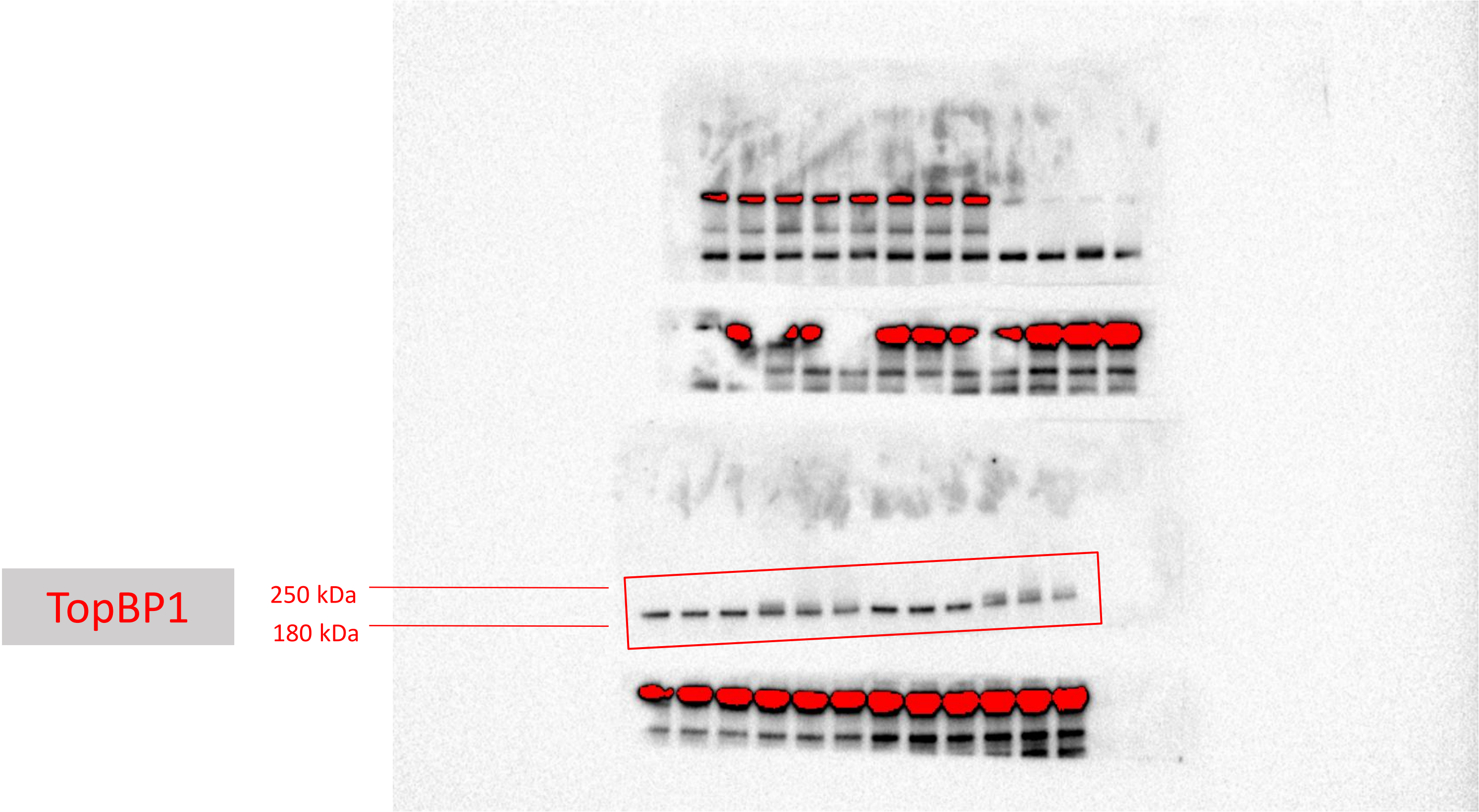

Merge chemiluminescence bands/colorimetric for vinculin

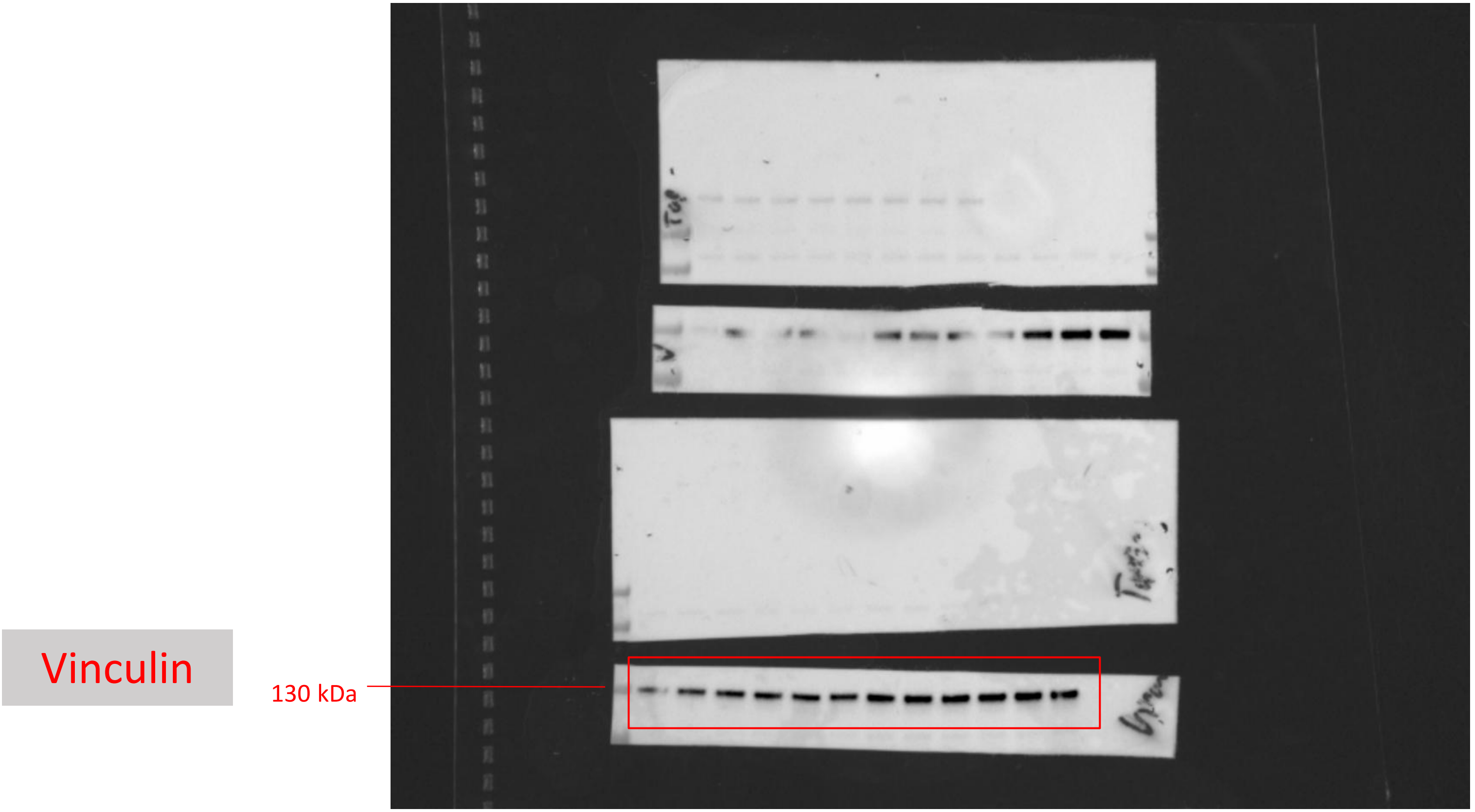

Chemiluminescence bands for vinculin

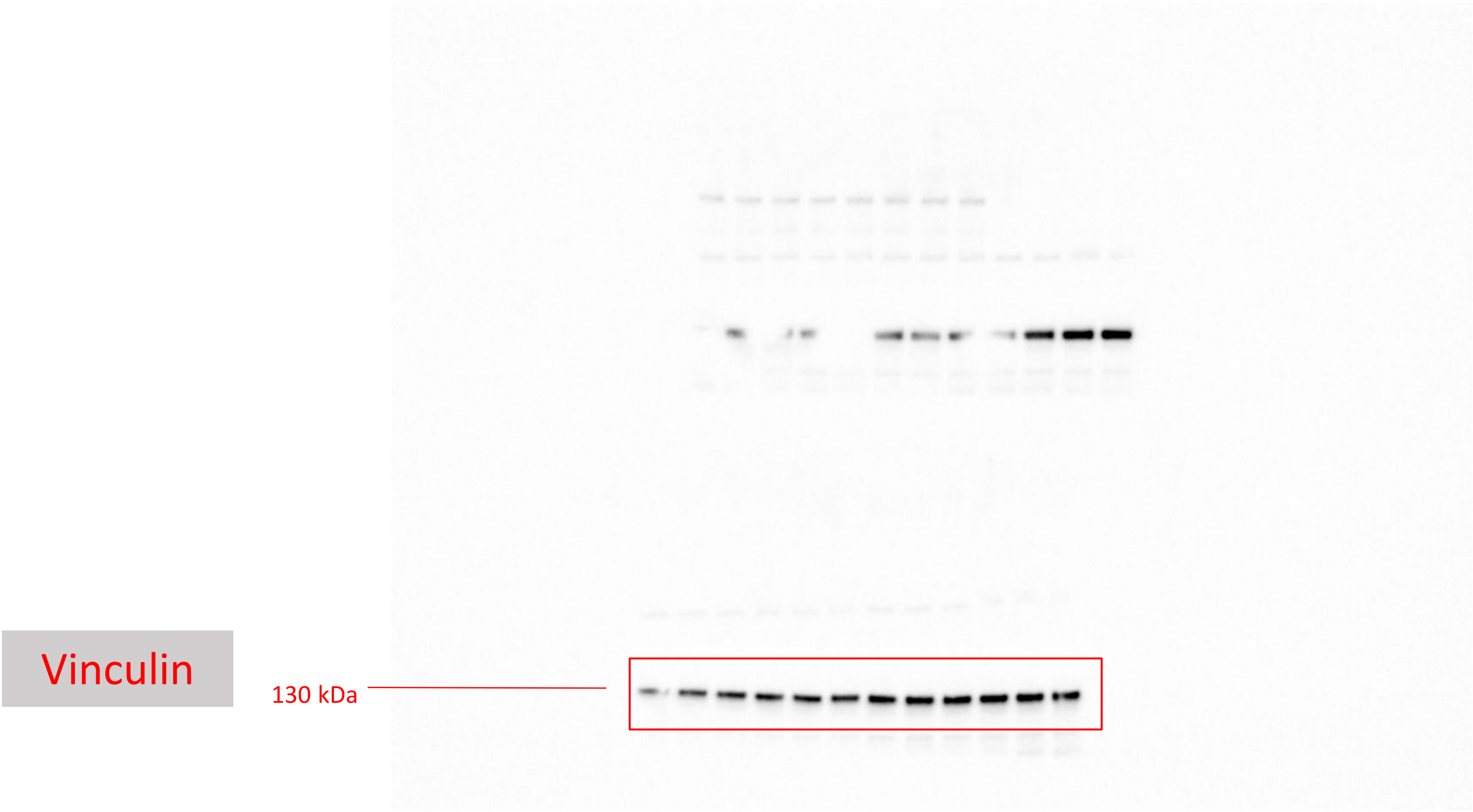

Colorimetric for TopBP1 & vincu

TopBP1
Vinculin

250 kDa
180 kDa
130 kDa

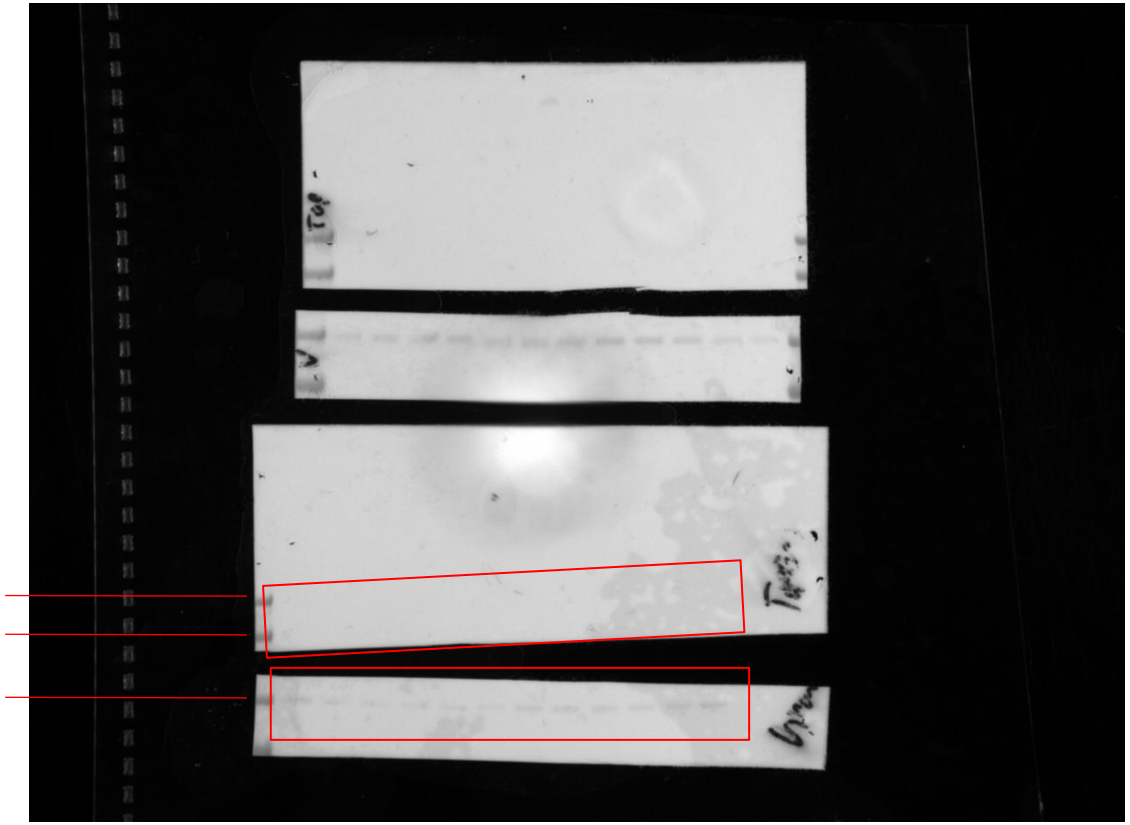

Merge chemiluminescence bands/colorimetric for GSK3-b

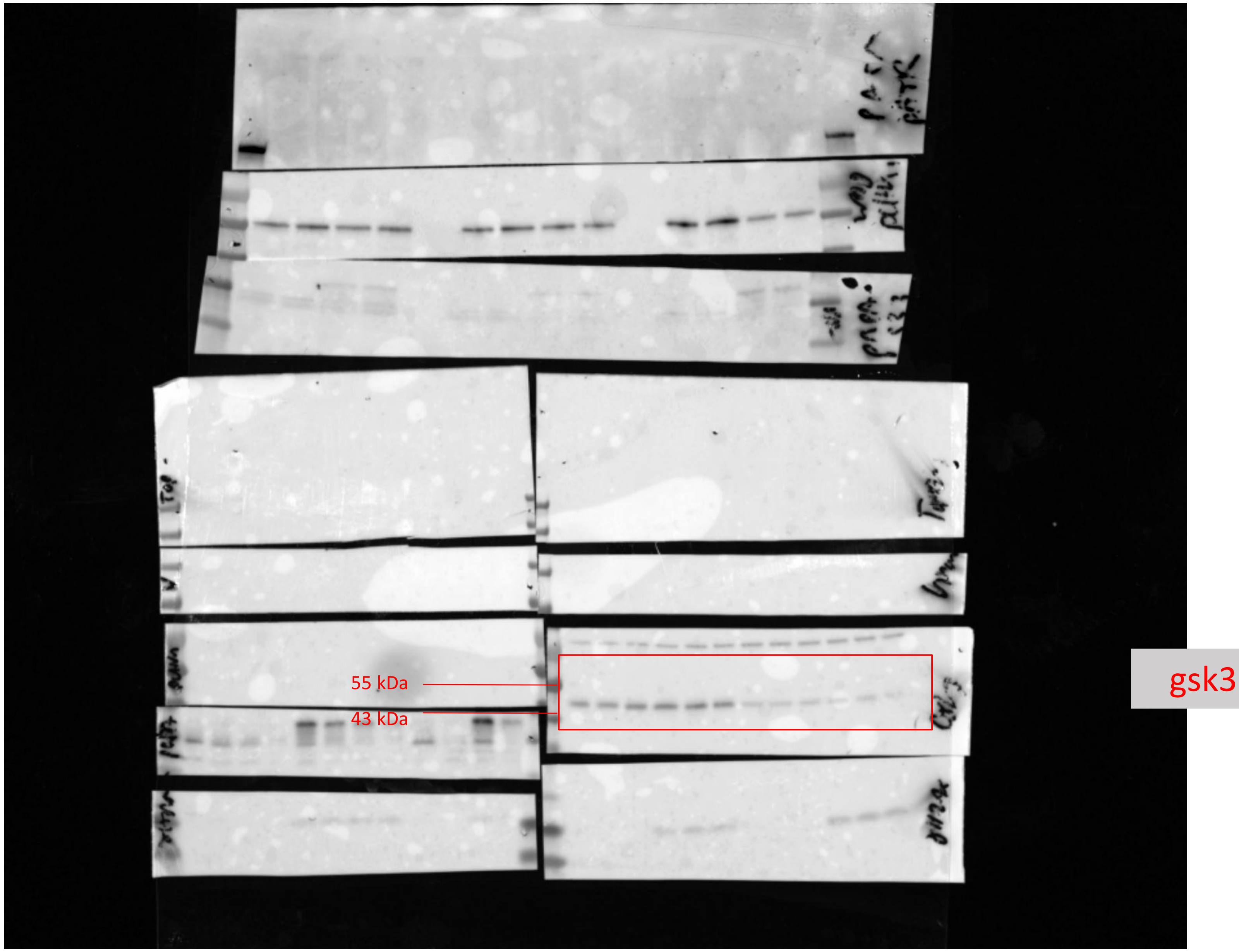

gsk3

chemiluminescence for GSK3-b

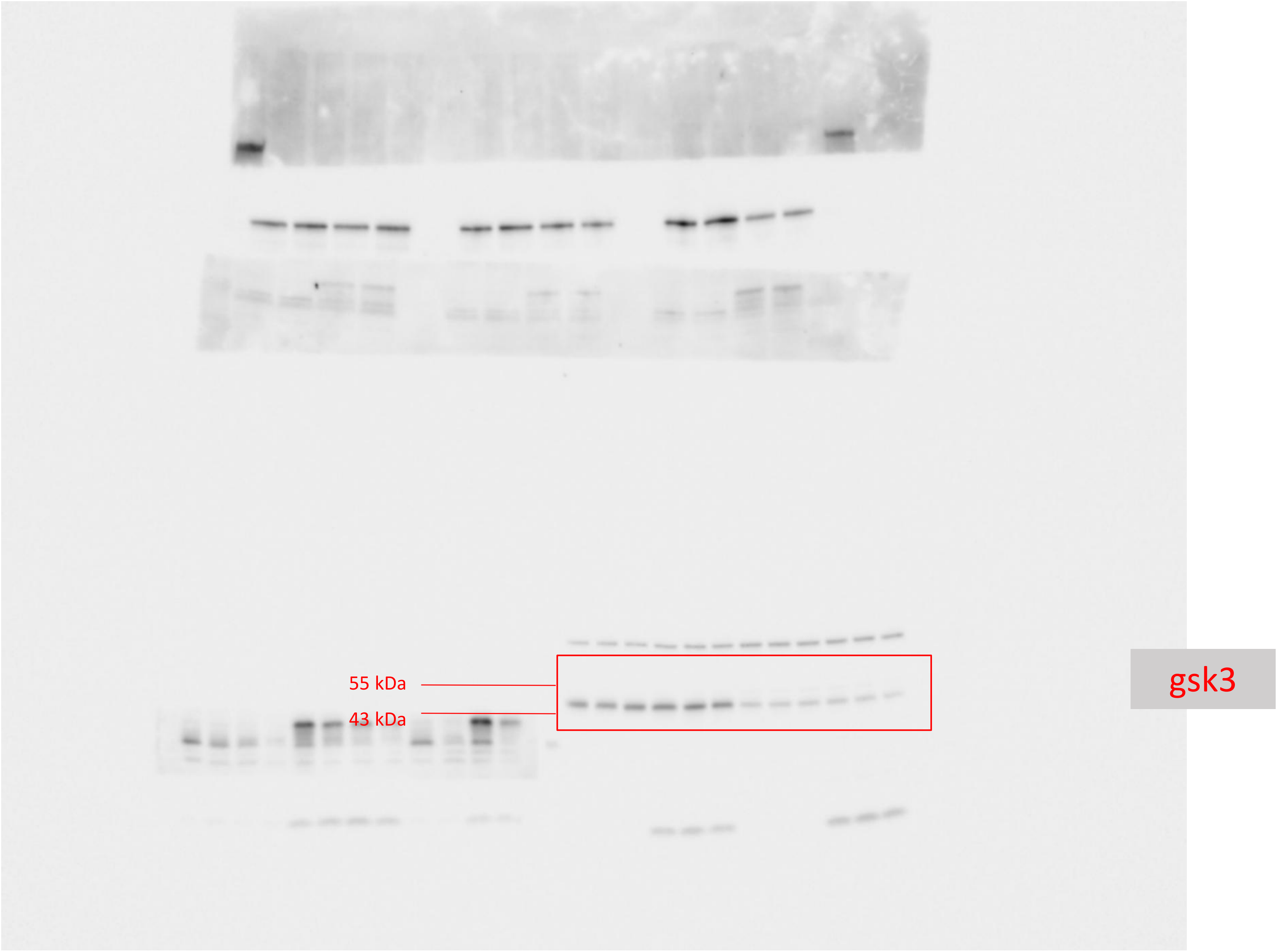

Merge chemiluminescence bands/colorimetric for gH2AX

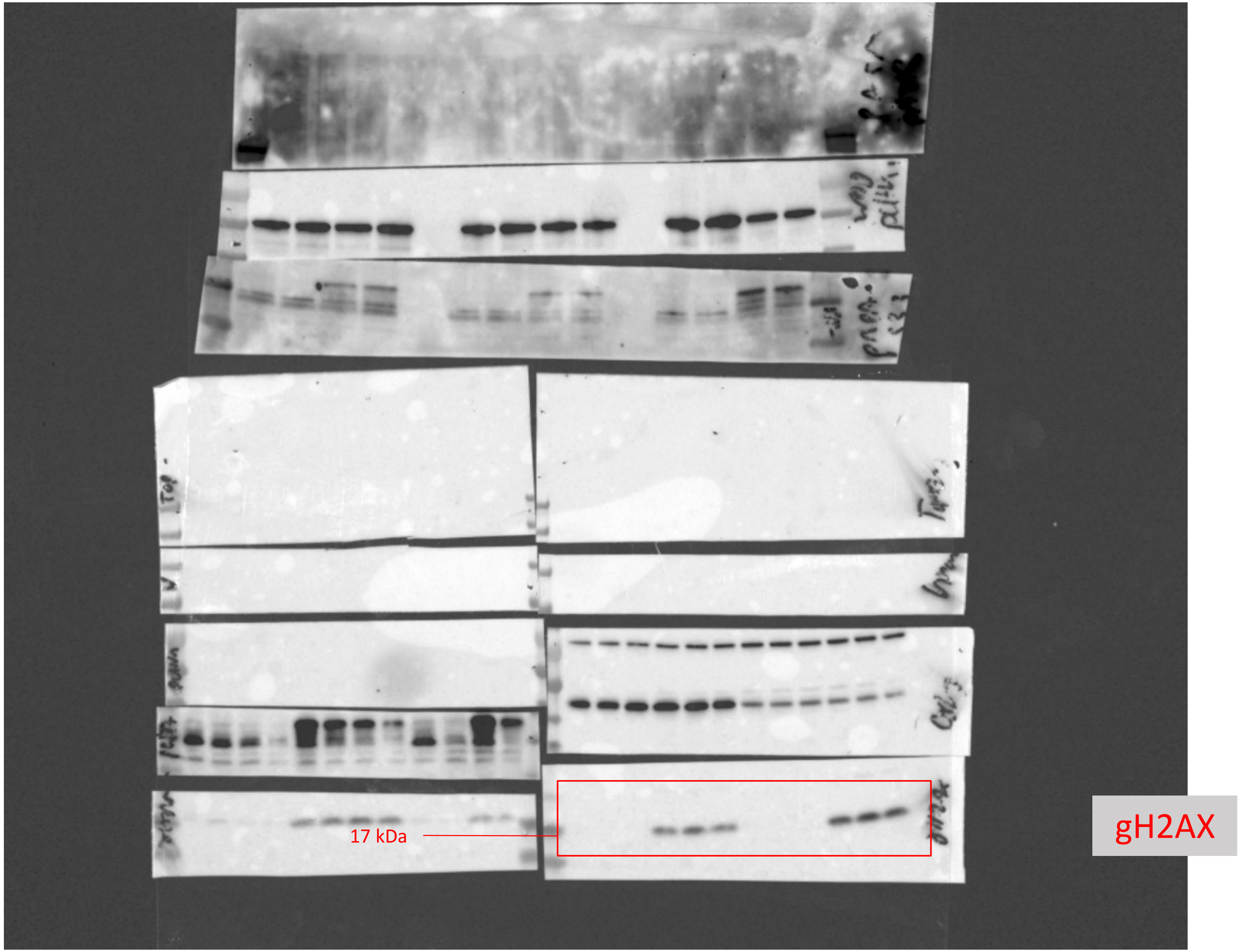

chemiluminescence for gH2AX

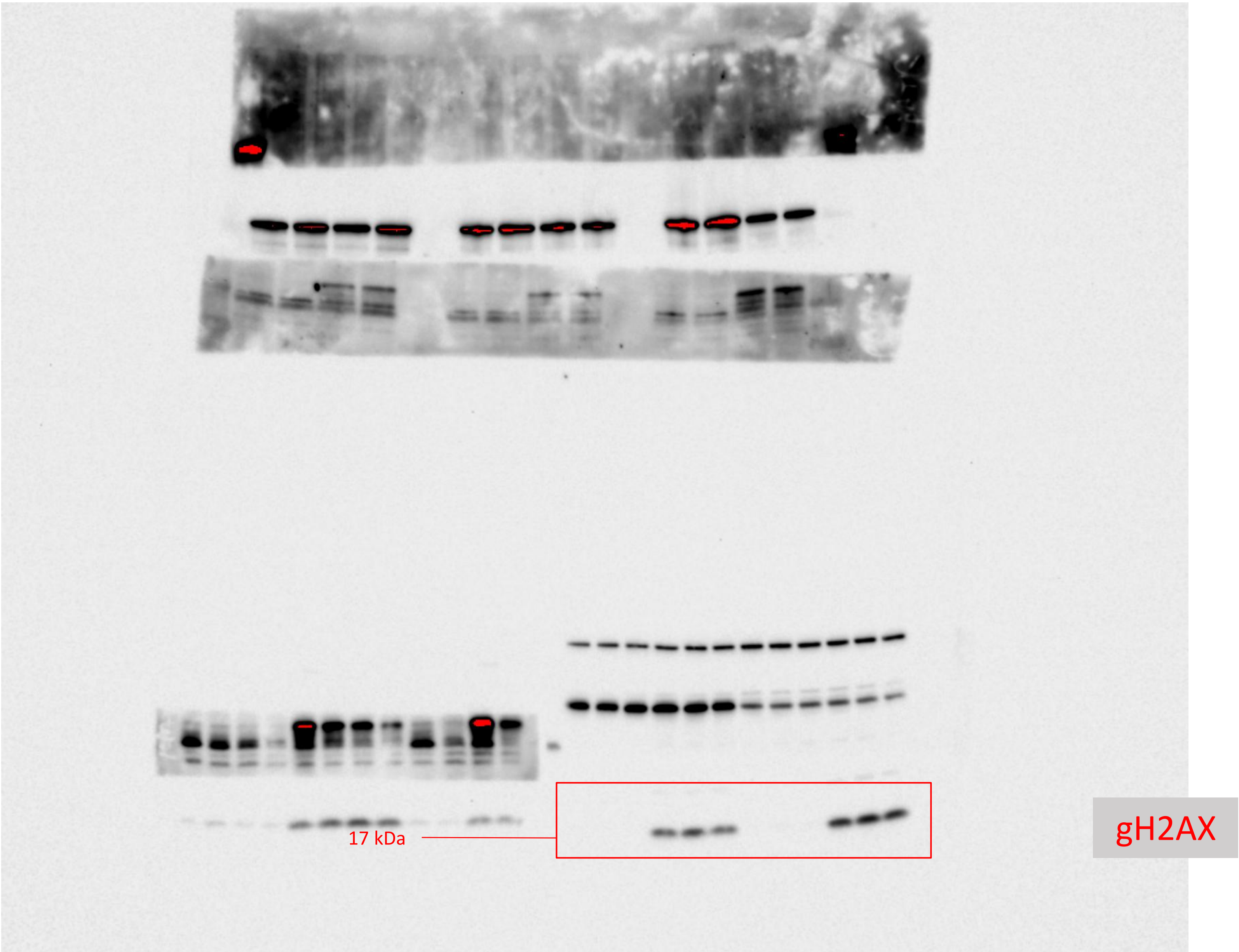

Merge chemiluminescence bands/colorimetric for GSK3-b+gH2AX

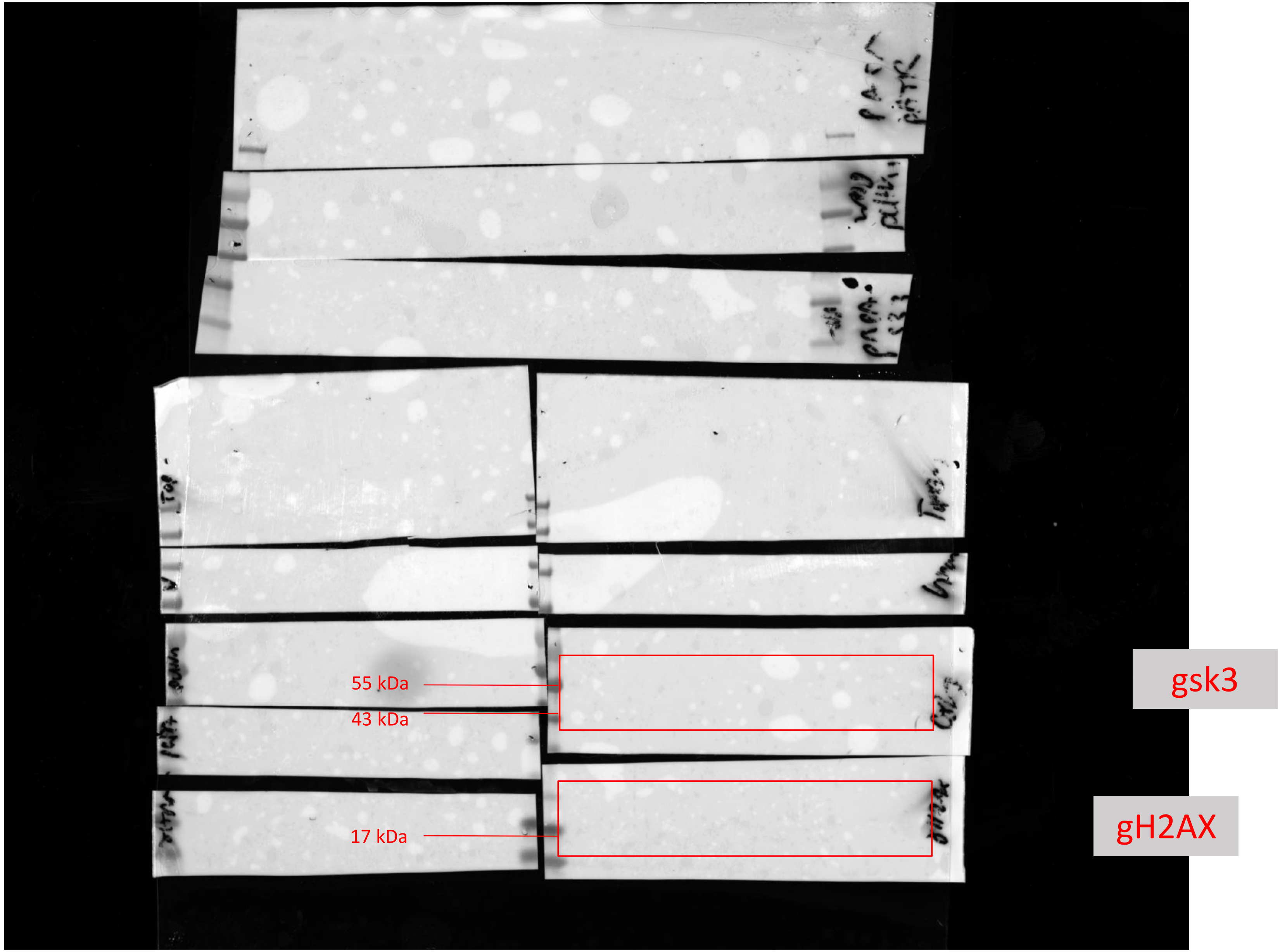

Supplement: Figure 2—figure supplement 3—source data 1. [file elife-106196-fig2-figsupp3-data1.zip › Figure 2-figure supplement 3A and 3B-Source Data 1/Figure 2-figure supplement 3A-Source Data 1.pdf]
