# Supplementary material for: TopBP1 biomolecular condensates as a new therapeutic target in advanced-stage colorectal cancer
Source: eLife. 2025 Oct 21;14:RP106196. doi: 10.7554/eLife.106196 (PMC12539802; doi:10.7554/eLife.106196)

Figure 2-figure supplement 3B

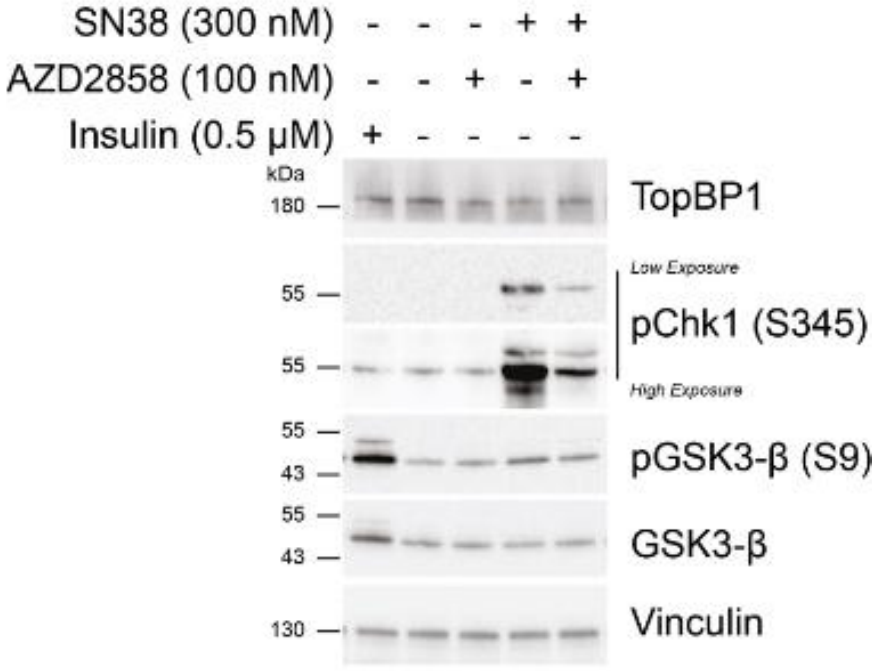

Figure 2-figure supplement 3B: Below are the Original membranes corresponding to Figure 2-figure supplement 3B Immunoblot of indicated proteins after incubation of HCT116 cells with AZD2858 (100 nM) or/and SN-38 (300 nM), or insulin (0.5 µM) (positive control of GSK-3β inhibition).

Merge chemiluminescence bands/colorimetric for pChk1 low

pChk1(S345)

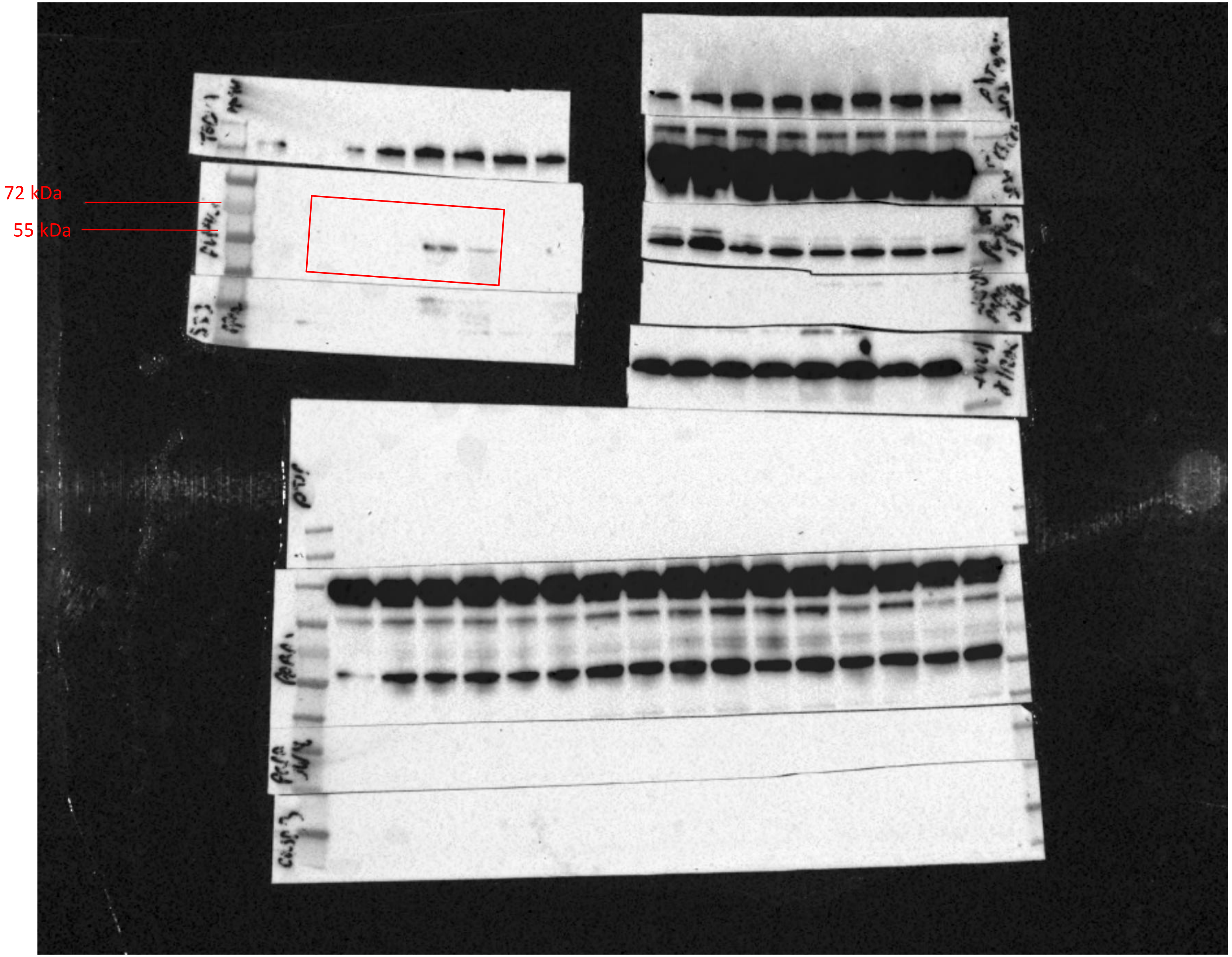

Chemiluminescence bands for pChk1 low

pChk1(S345)

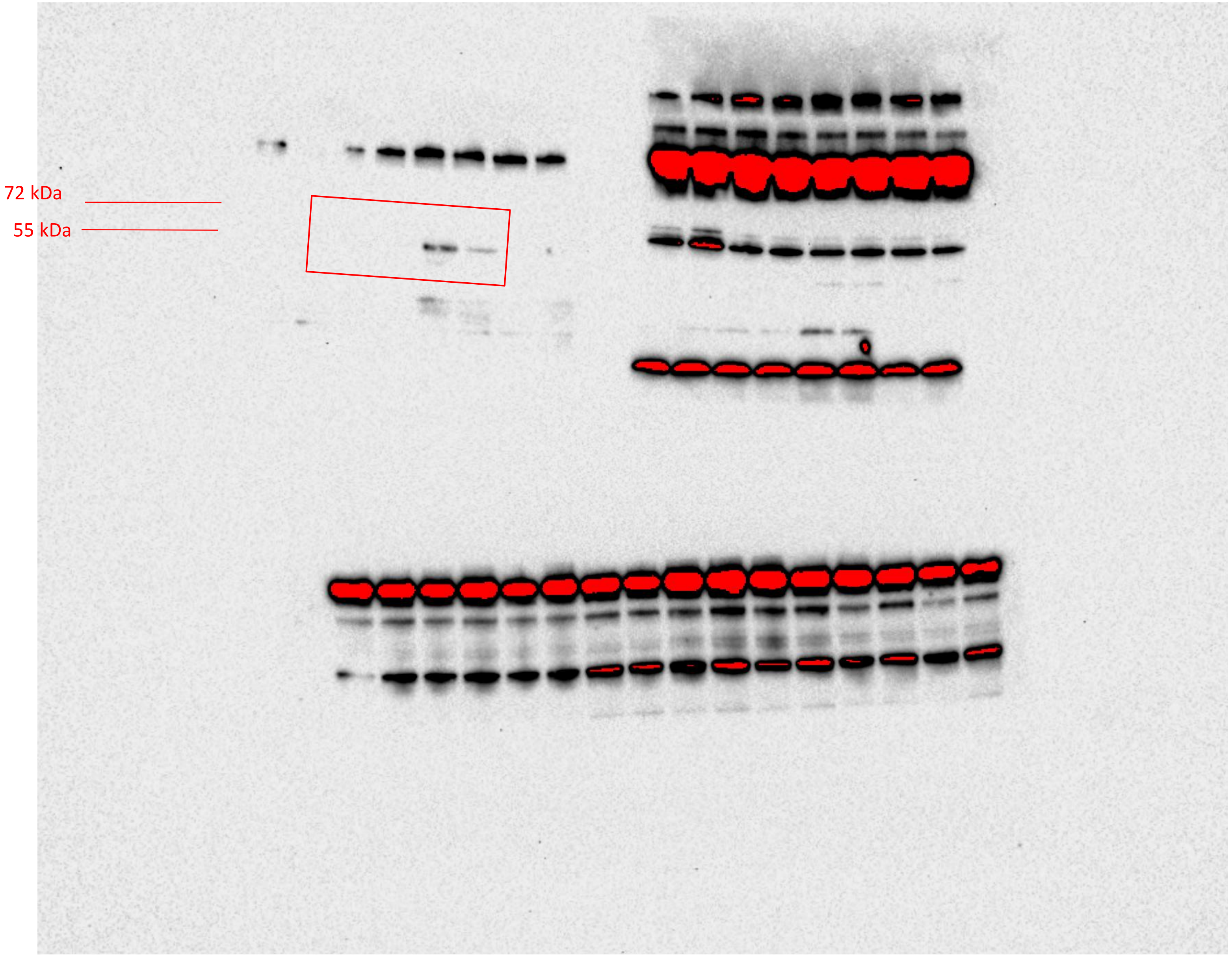

Merge chemiluminescence bands/colorimetric for GSK3b

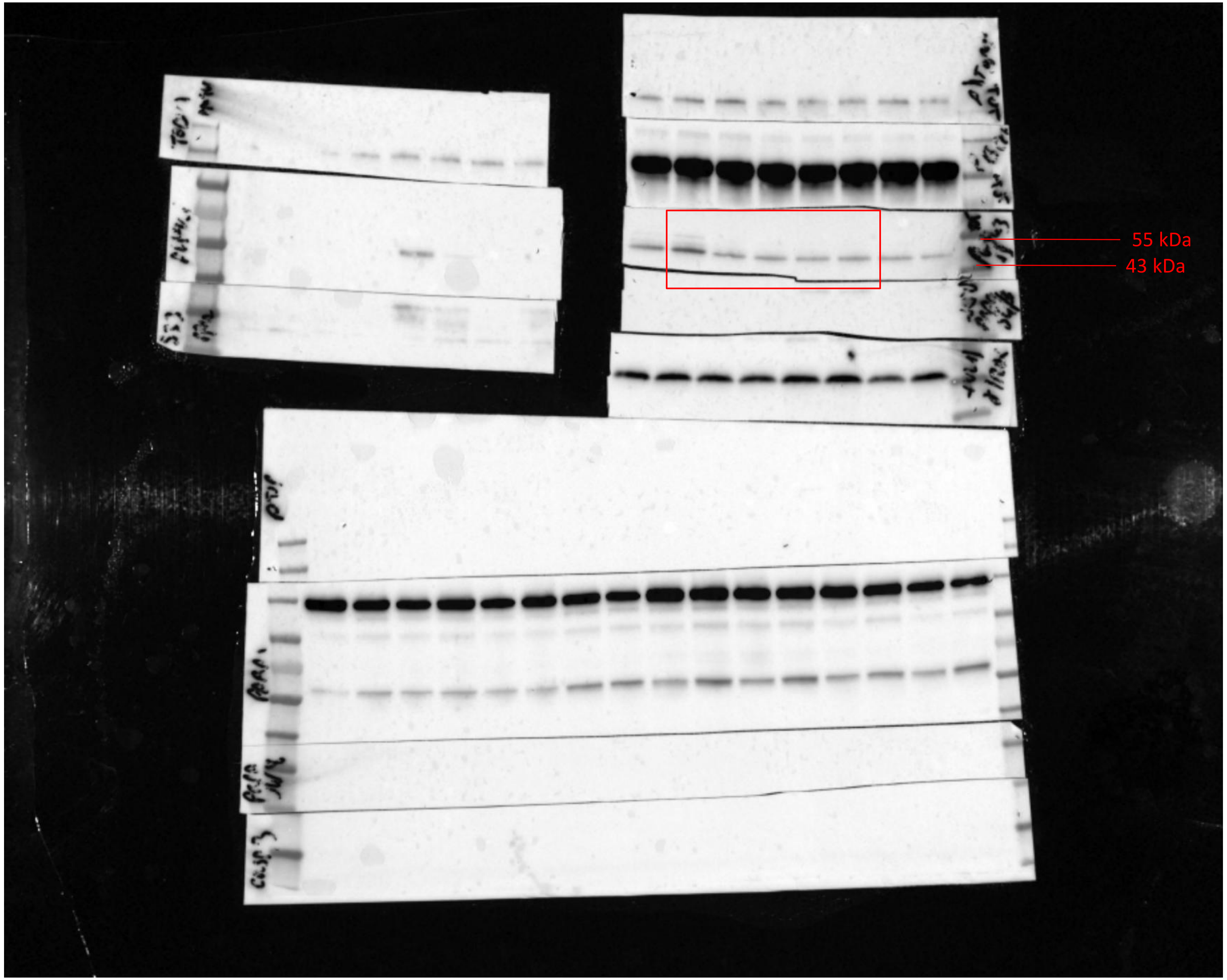

Chemiluminescence bands for GSK3b

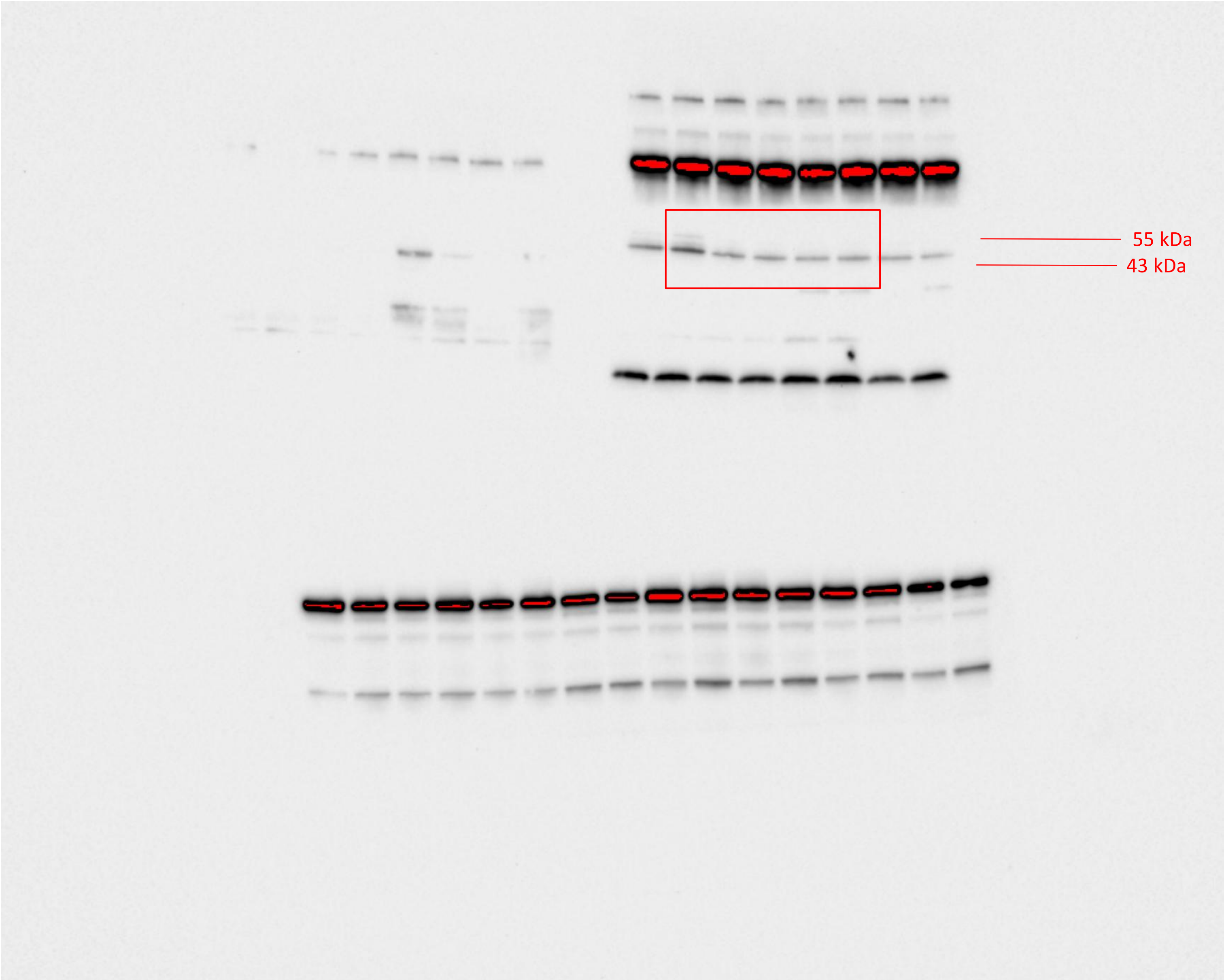

Colorimetric for pChk1 low

pChk1(S345)

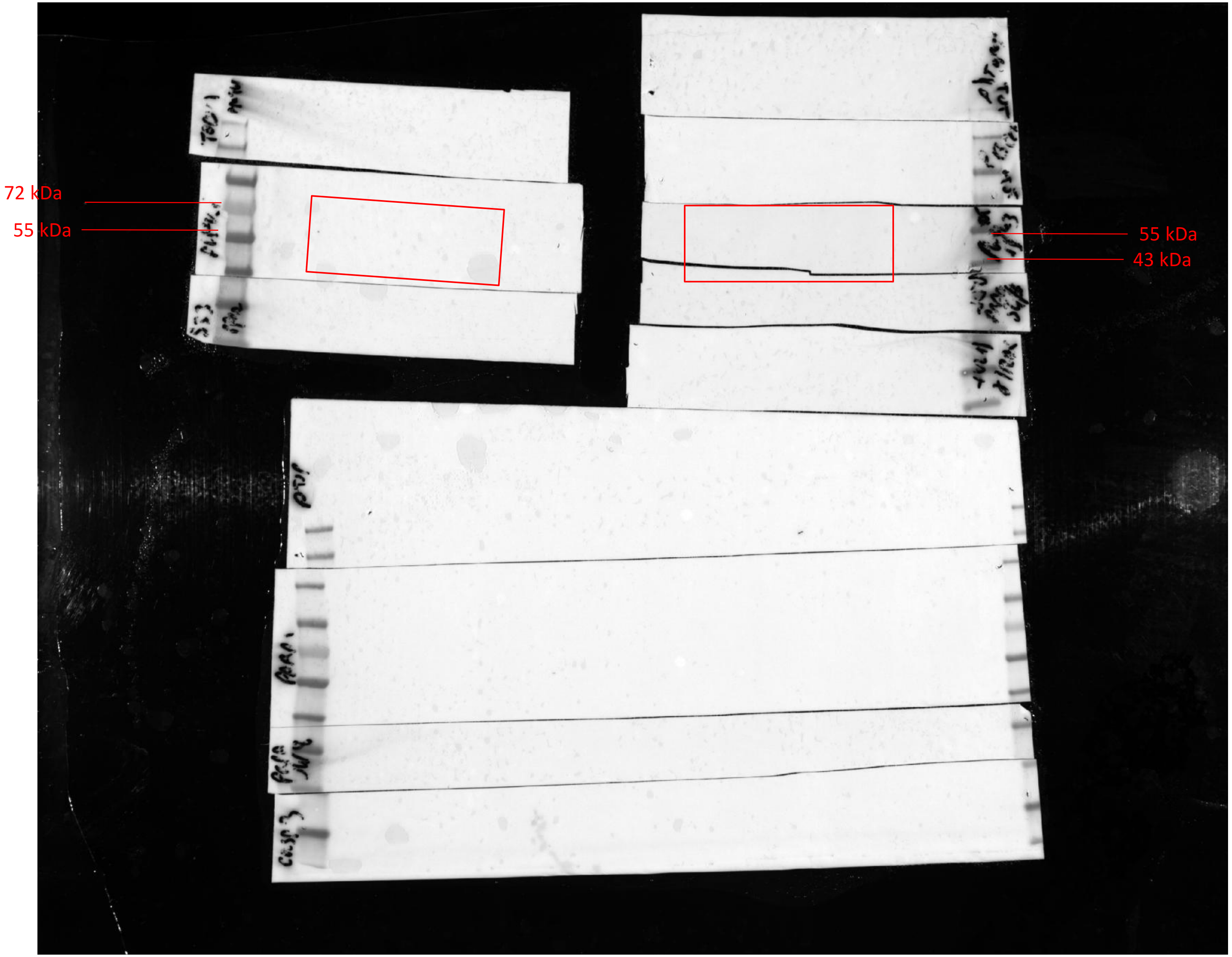

Merge chemiluminescence bands/colorimetric for pChk1 high

pChk1(S345)

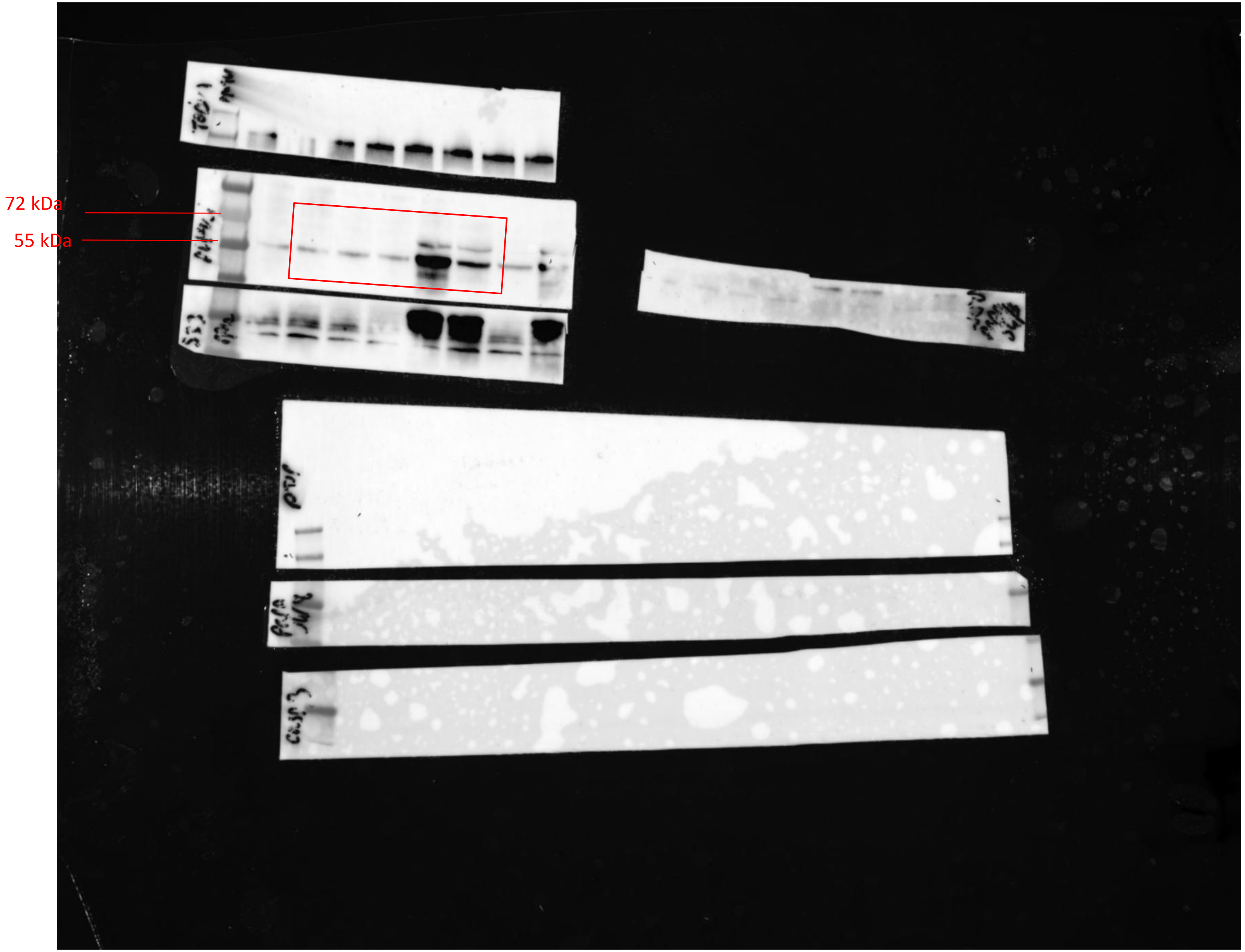

Chemiluminescence bands for pChk1 high

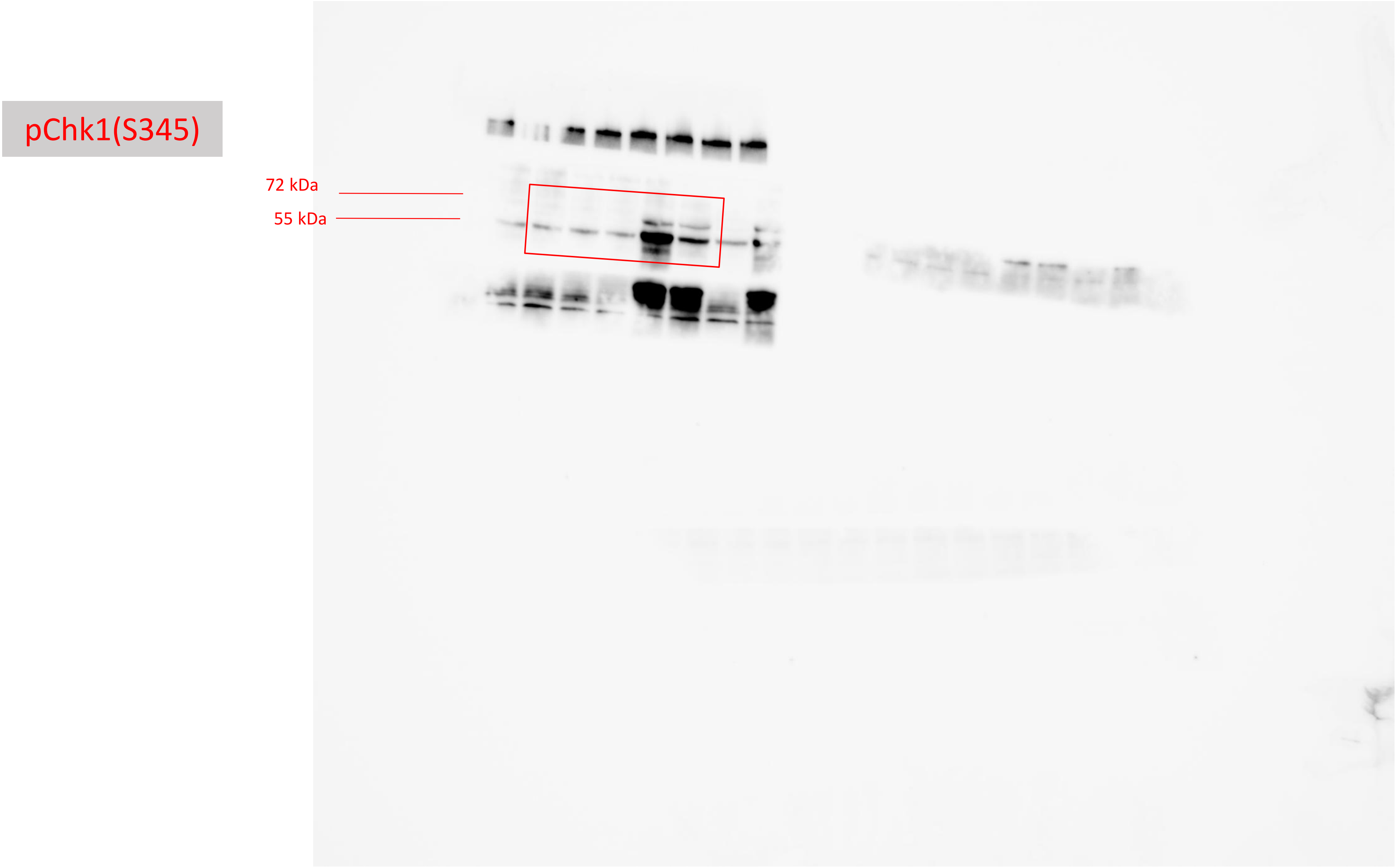

Colorimetric for pChk1 high

pChk1(S345)

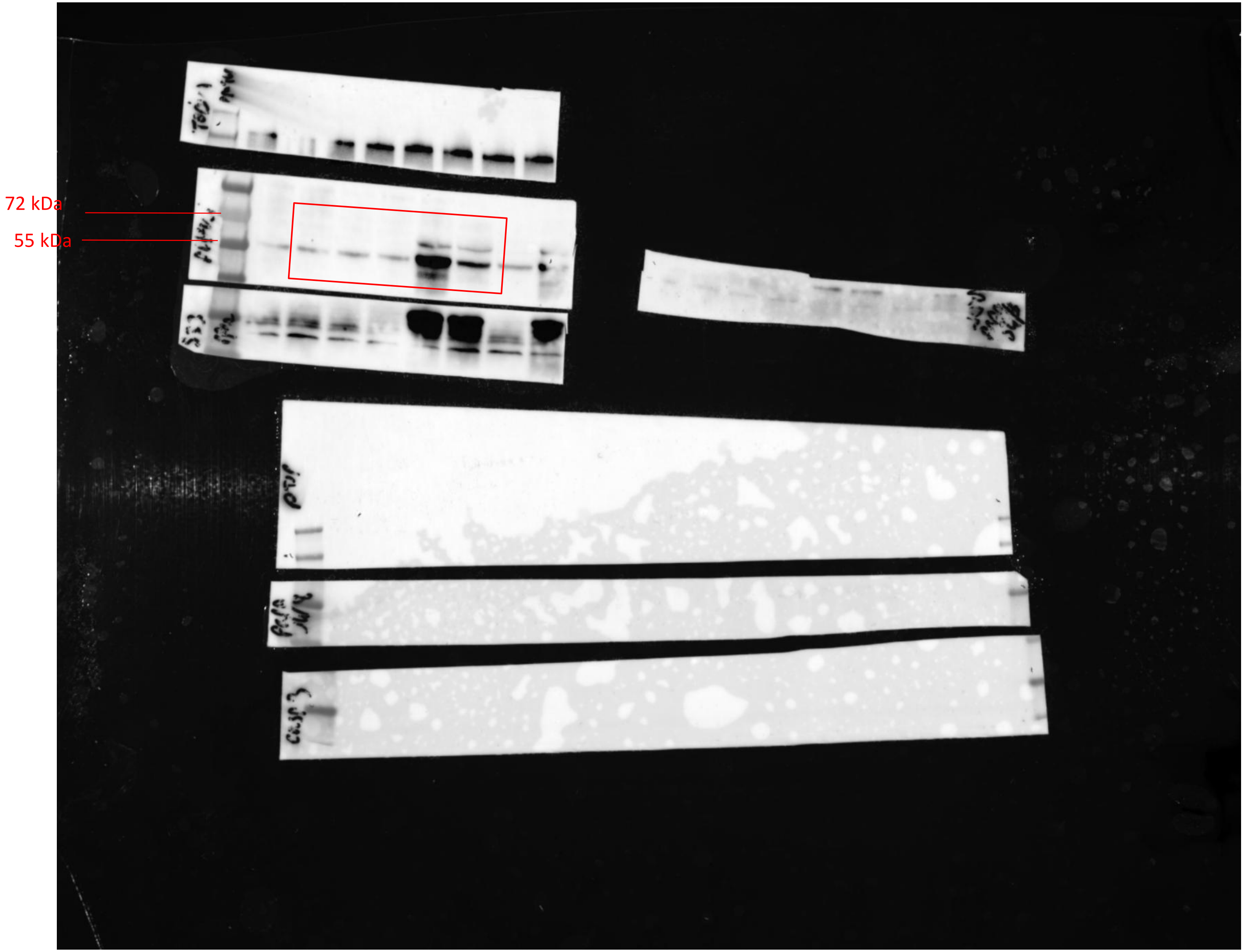

Merge chemiluminescence bands/colorimetric for TopBP1

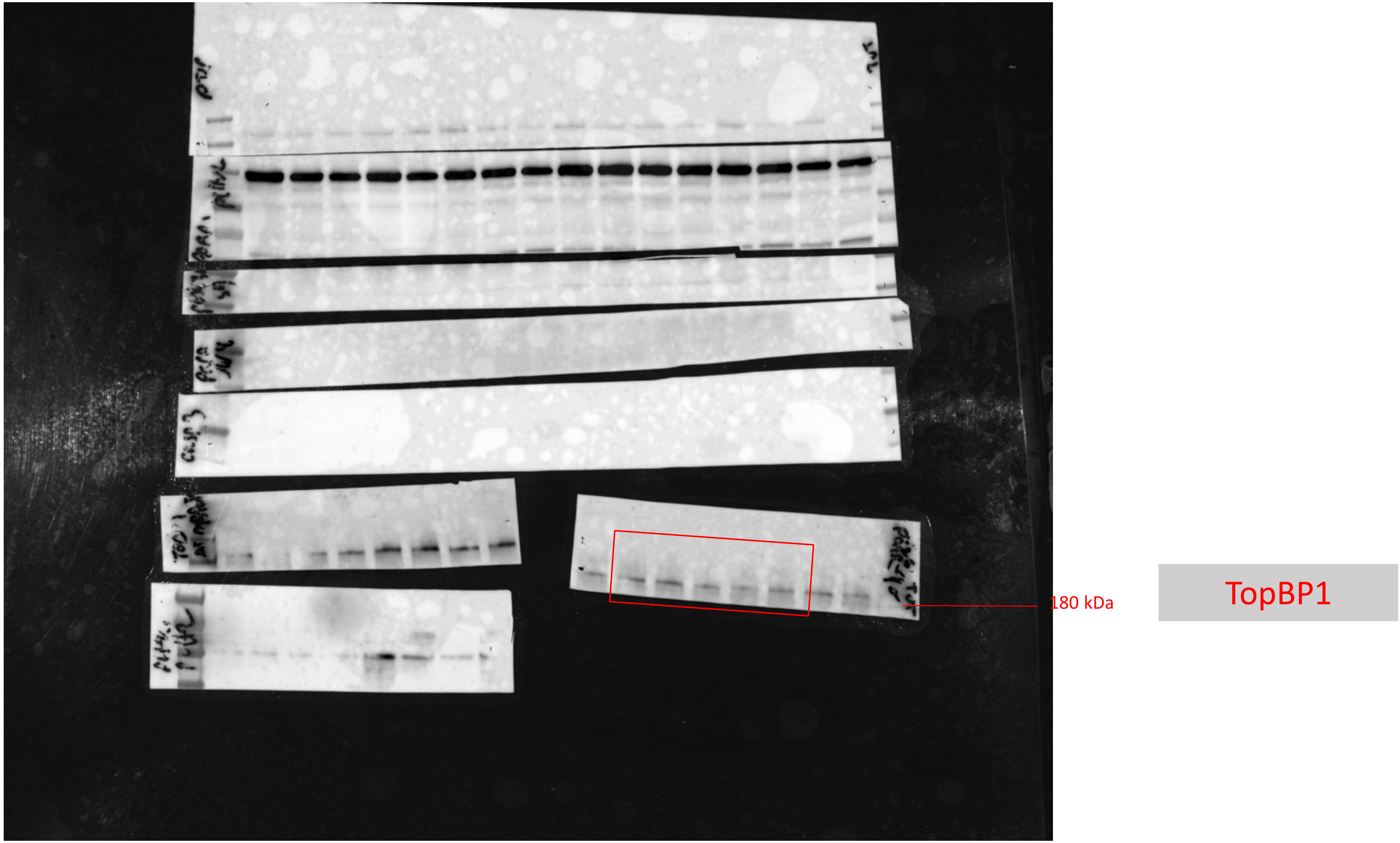

chemiluminescence for TopBP1

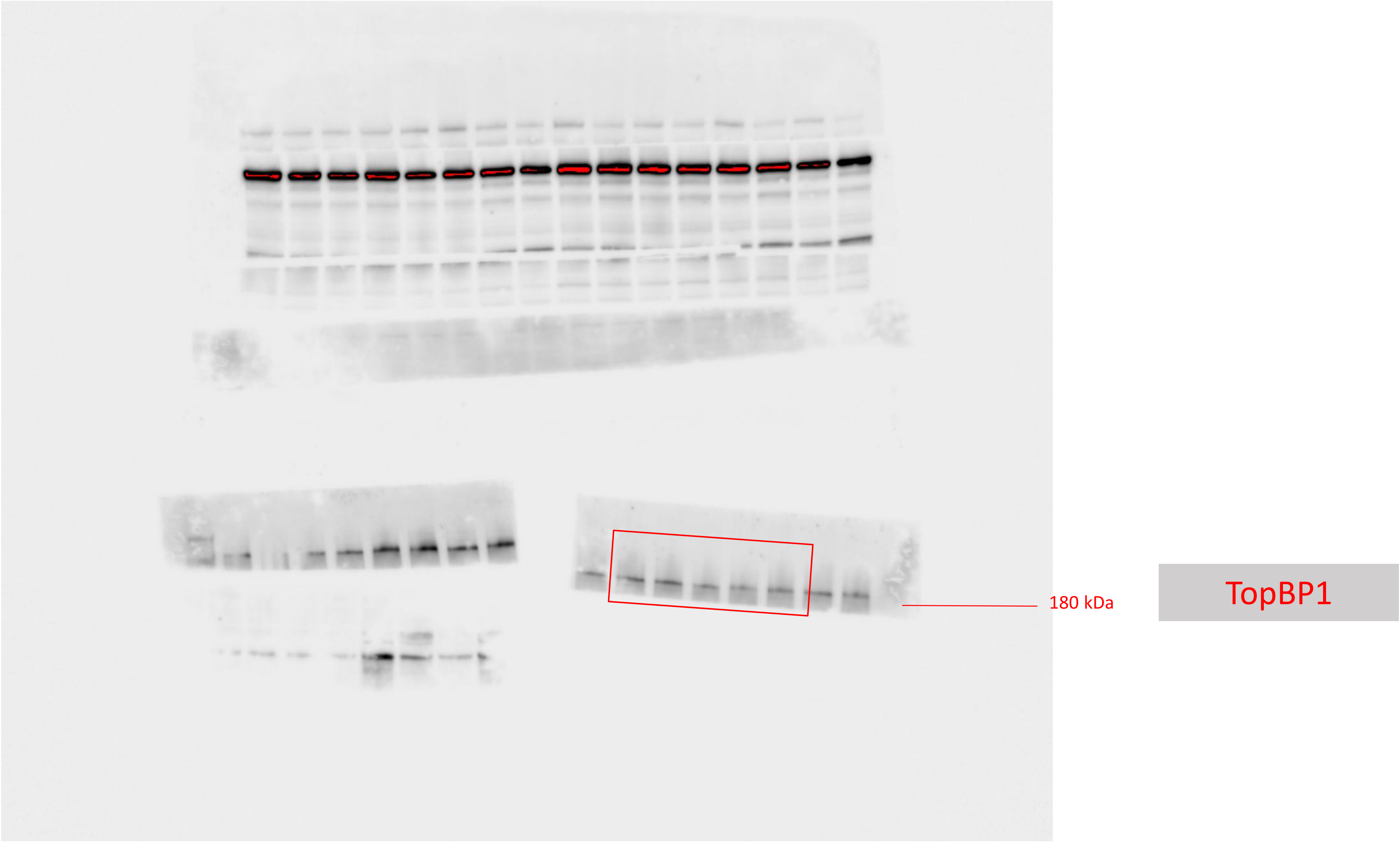

Merge chemiluminescence bands/colorimetric for TopBP1

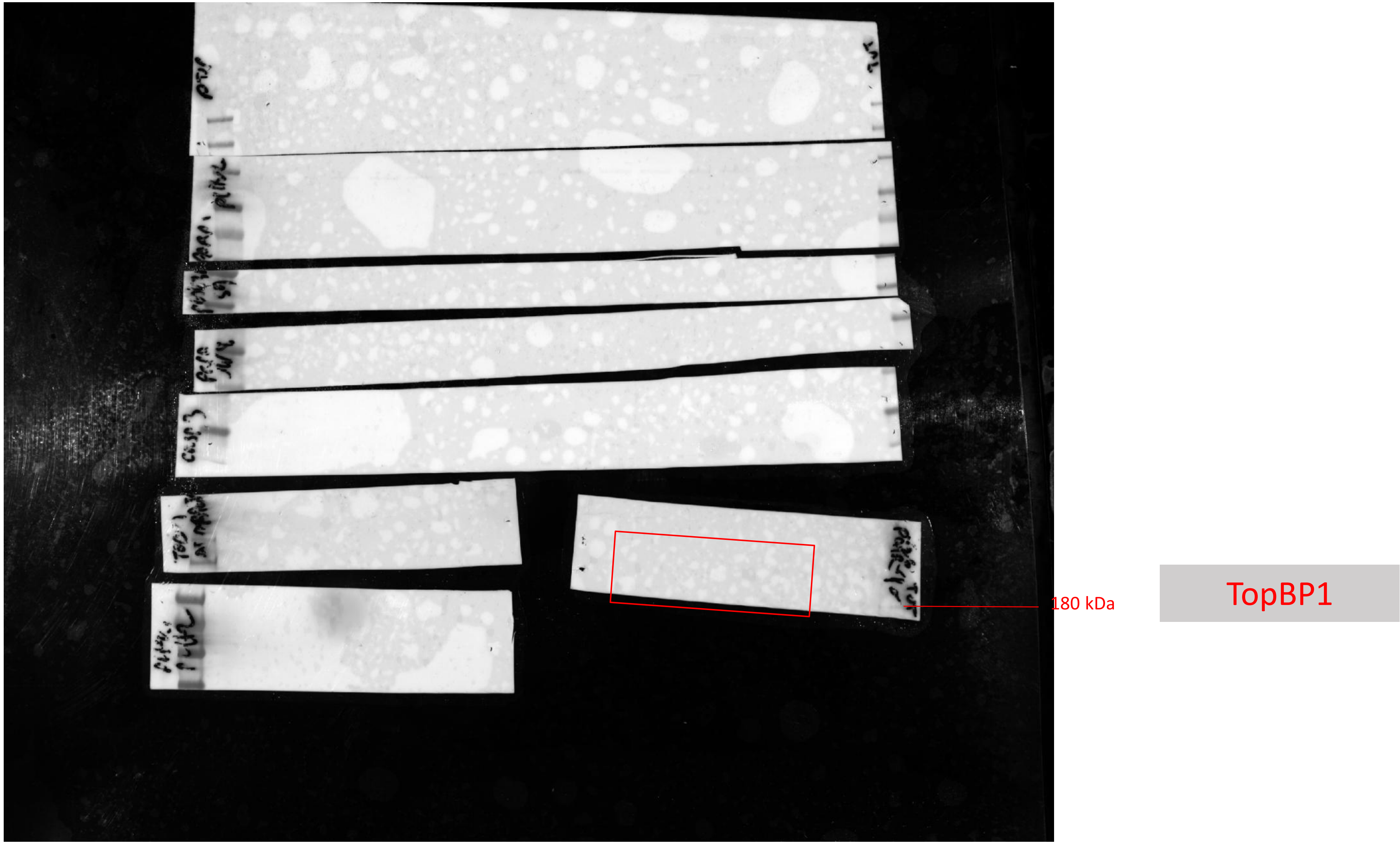

Merge chemiluminescence bands/colorimetric for pGSK3 S9

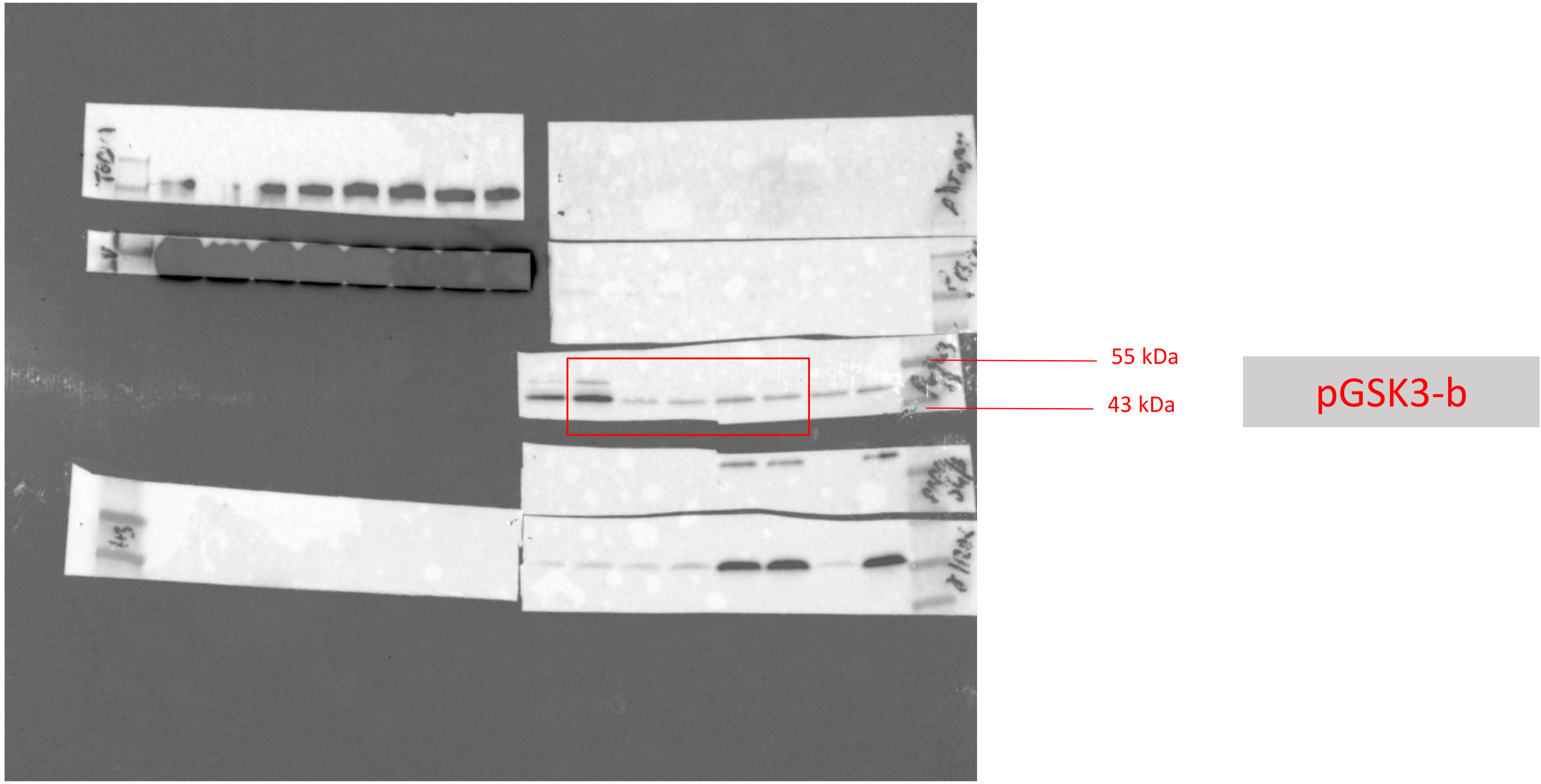

Chemiluminescence bands for pGSK3 S9

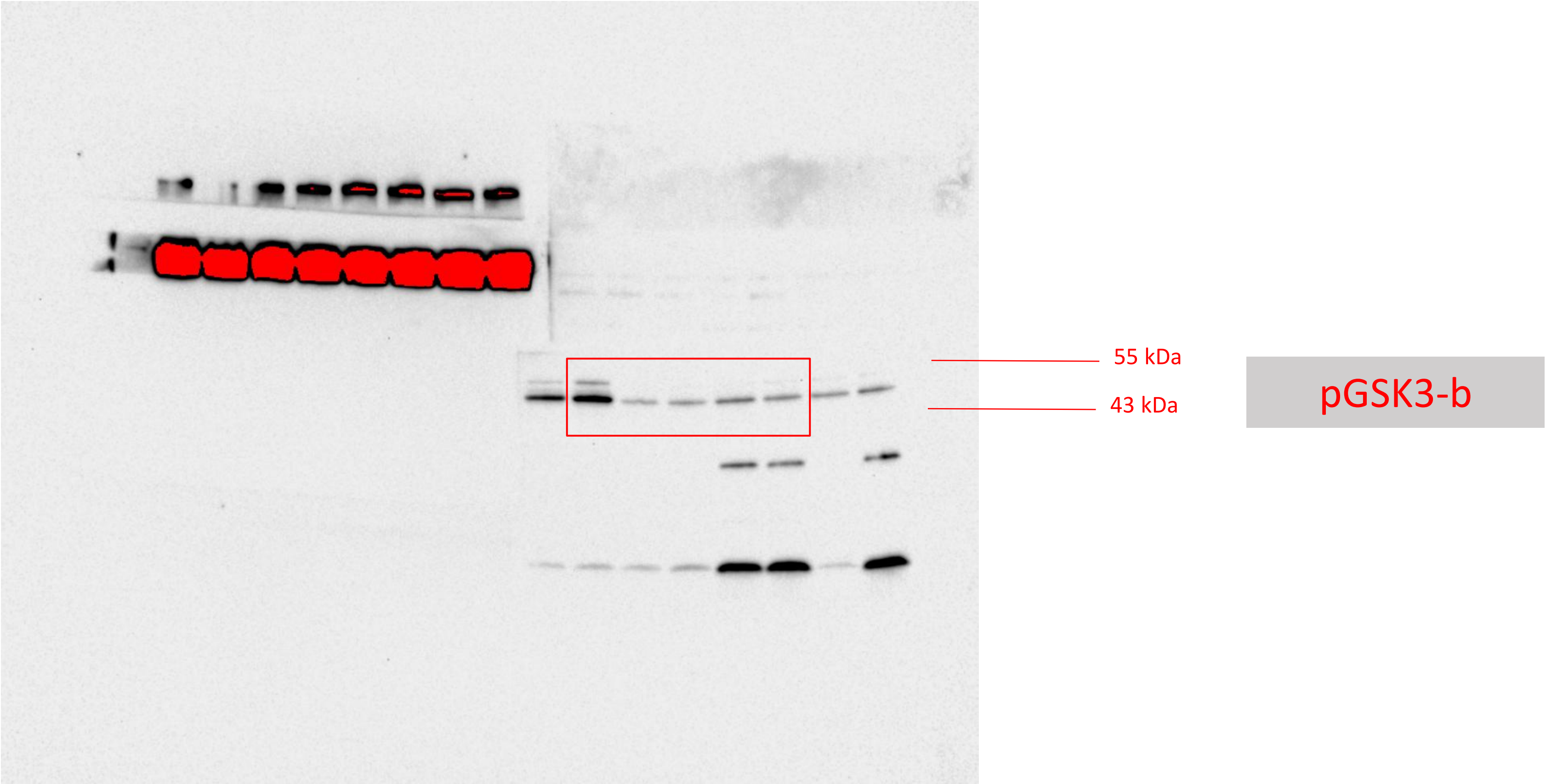

Merge chemiluminescence bands/colorimetric for pGSK3

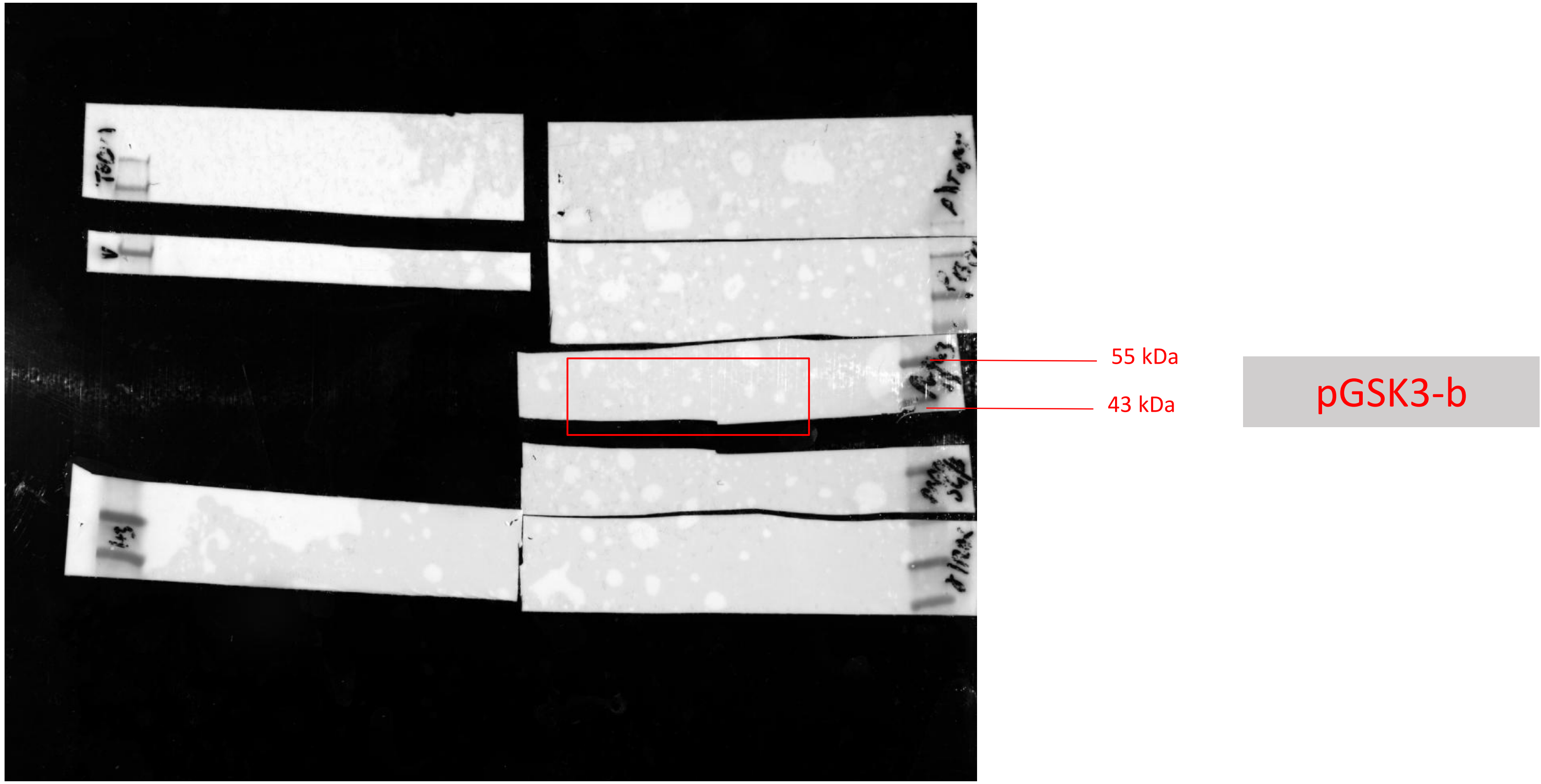

Merge chemiluminescence bands/colorimetric for Vinculin

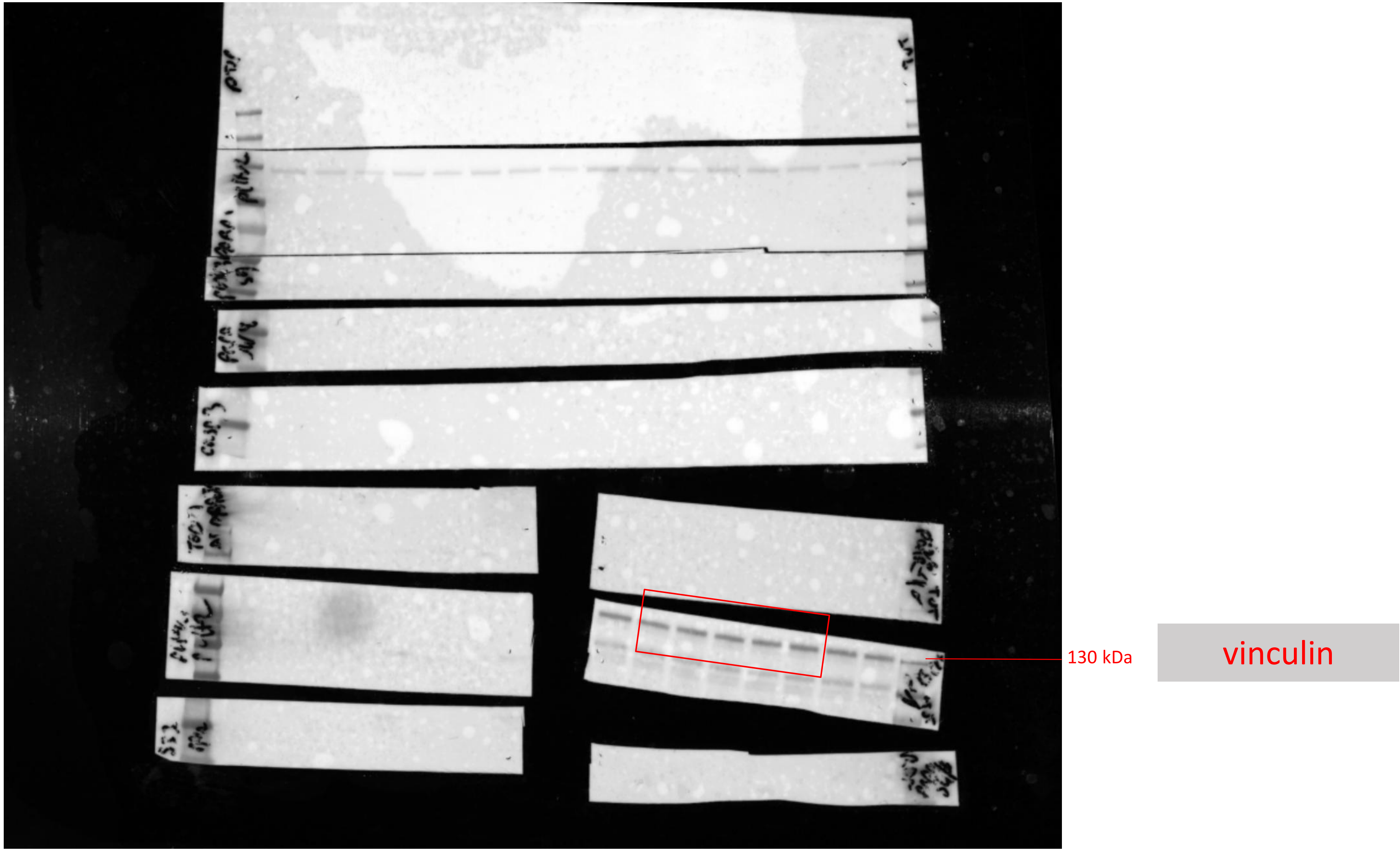

Chemiluminescence bands for Vincu

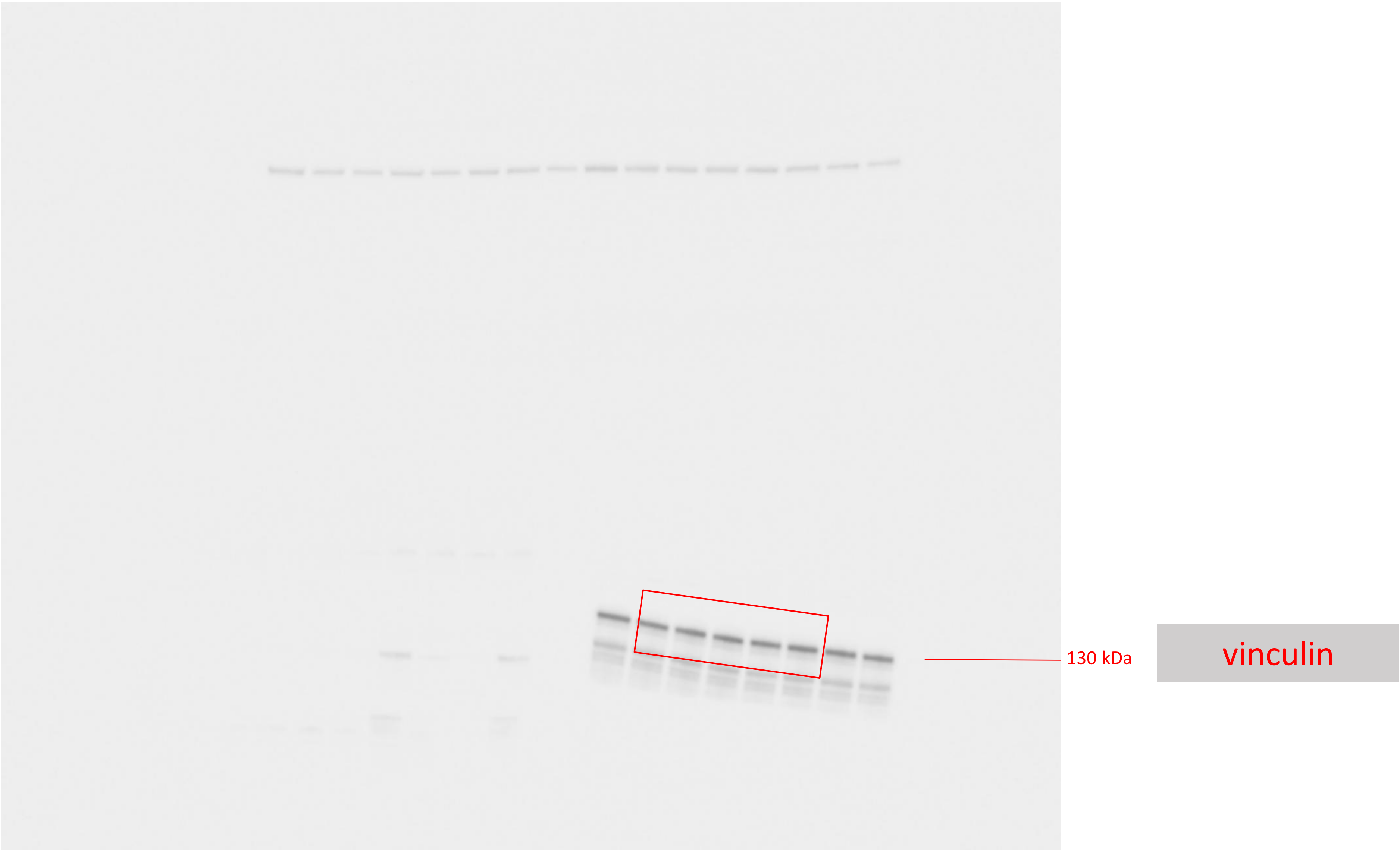

Supplement: Figure 2—figure supplement 3—source data 1. [file elife-106196-fig2-figsupp3-data1.zip › Figure 2-figure supplement 3A and 3B-Source Data 1/Figure 2-figure supplement 3B-Source Data 1.pdf]
