# Supplementary material for: TopBP1 biomolecular condensates as a new therapeutic target in advanced-stage colorectal cancer
Source: eLife. 2025 Oct 21;14:RP106196. doi: 10.7554/eLife.106196 (PMC12539802; doi:10.7554/eLife.106196)

Figure 3.E

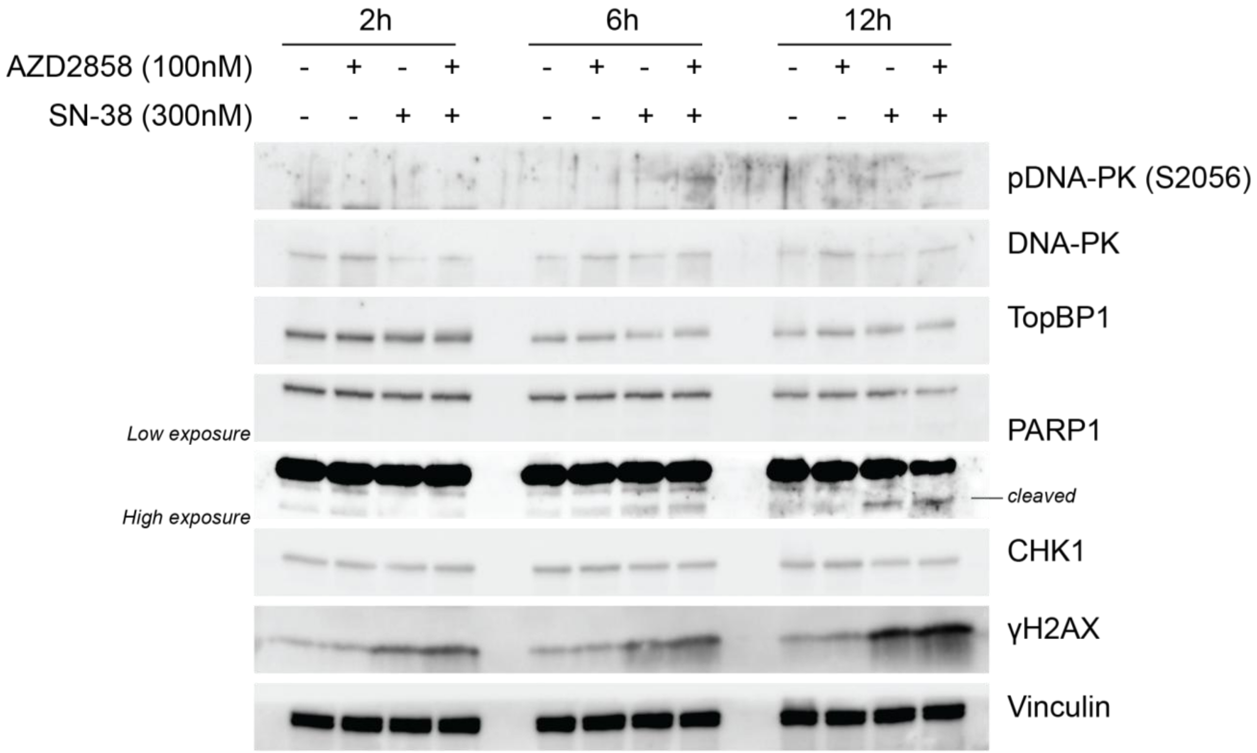

Figure 3E, Source Data 1. Below are the Original membranes corresponding to Figure 3E Immunoblot of the indicated proteins after incubation with AZD2858 (100 nM) or/and SN-38 (300 nM) for the indicated times. The experiment was replicated 3 times, and a representative replicate is shown.

Merge chemiluminescence bands/colorimetric for DNA-PK

DNA-PK

250 kDa

180 kDa

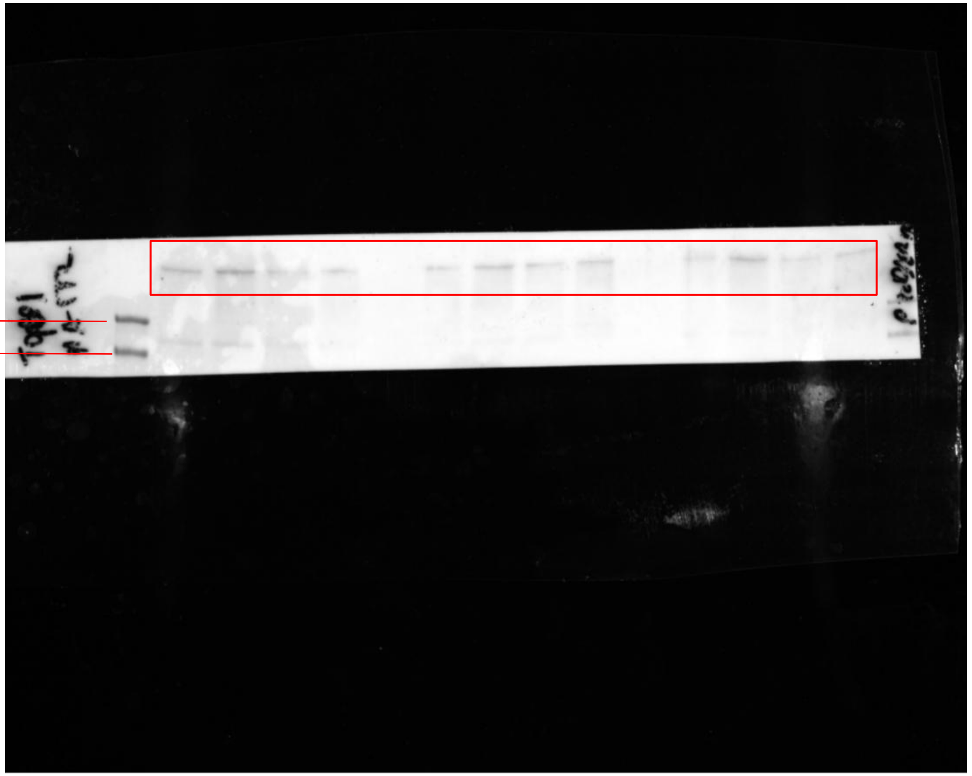

Chemiluminescence bands for DNA-PK

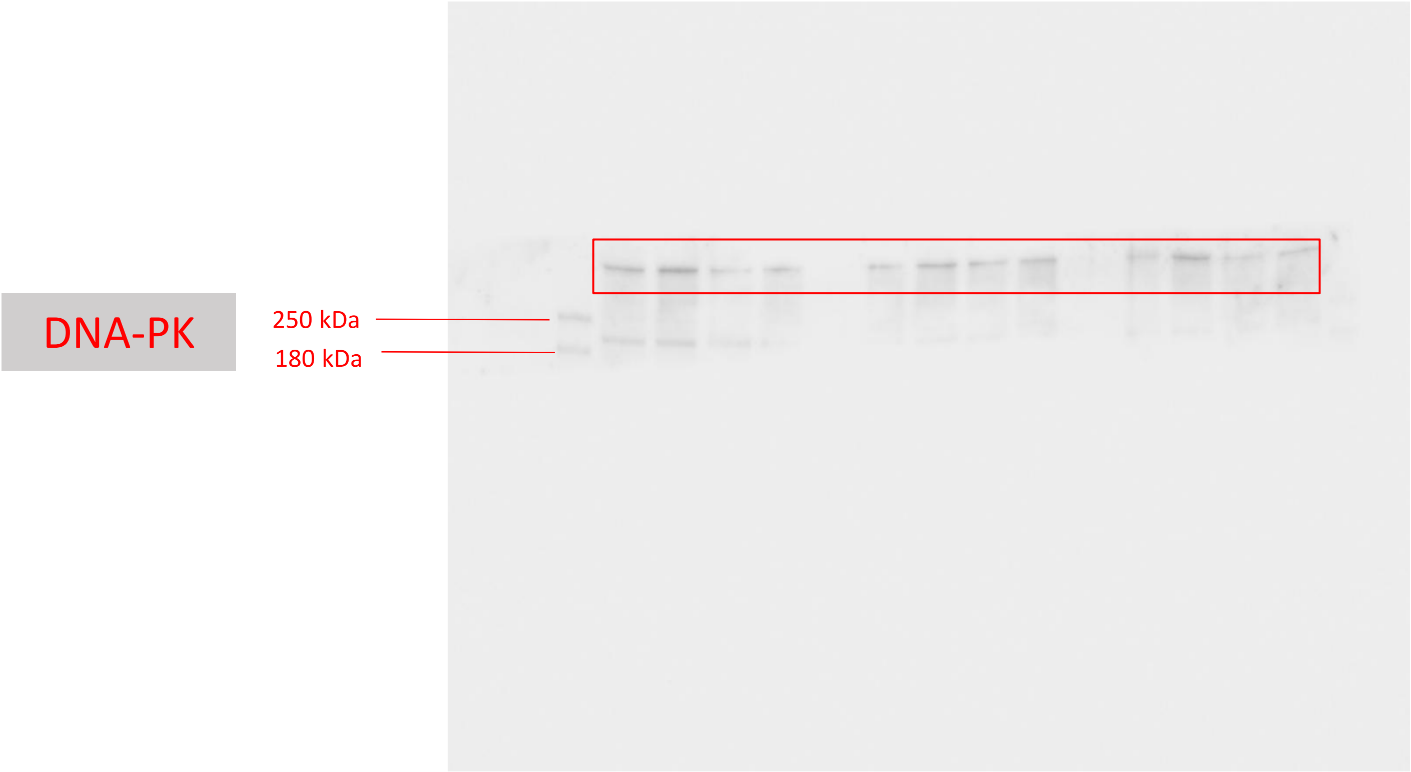

Colorimetric for DNA-PK

DNA-PK

250 kDa

180 kDa

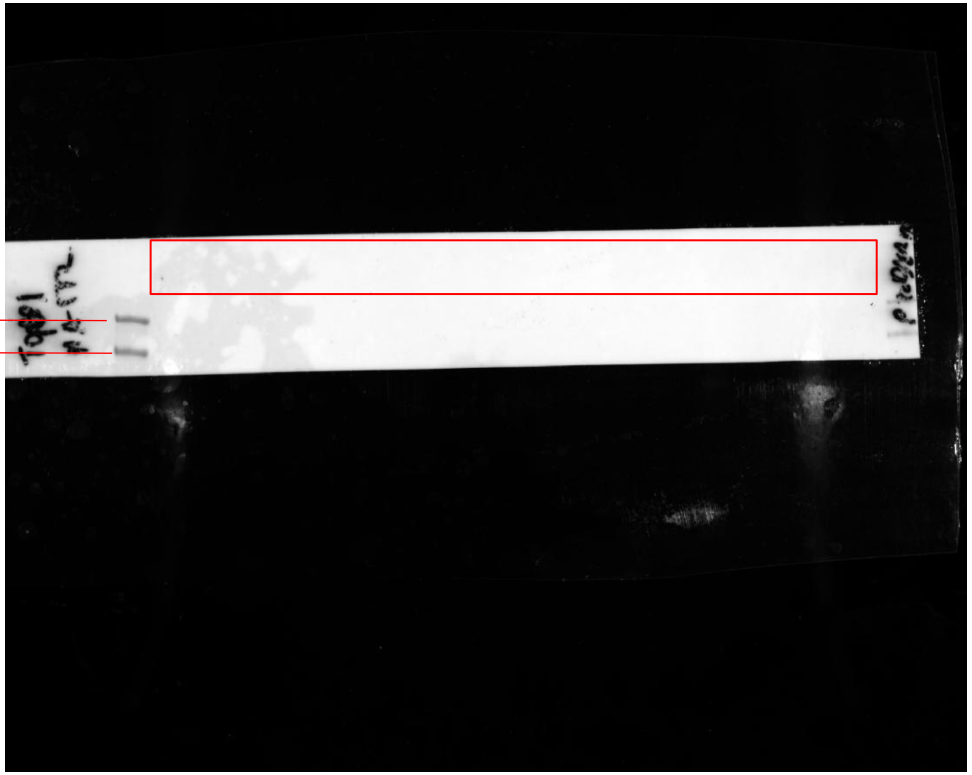

Merge chemiluminescence bands/colorimetric for pDNAPK

pDNA-PK (S2056)

250 kDa

180 kDa

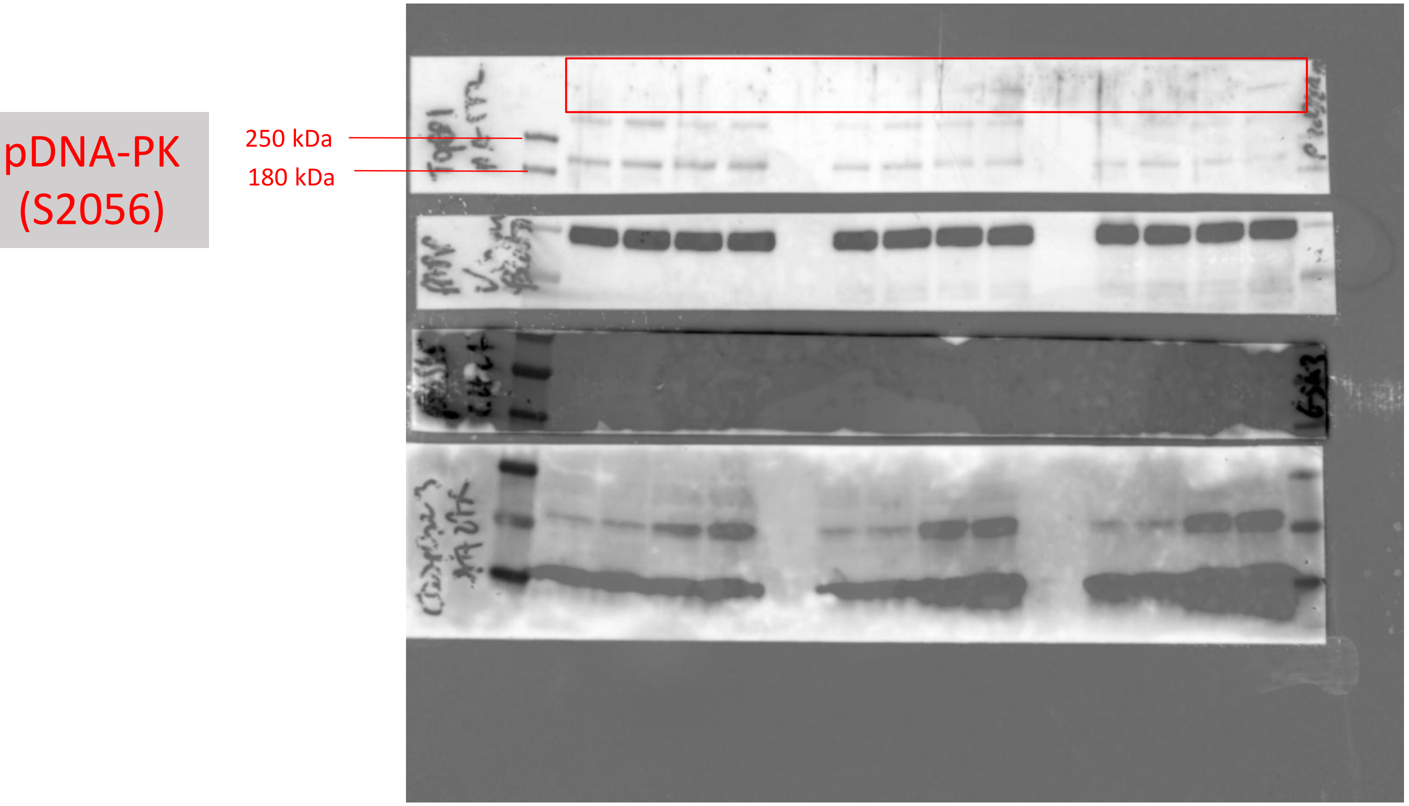

Chemiluminescence bands for pDNA-PK

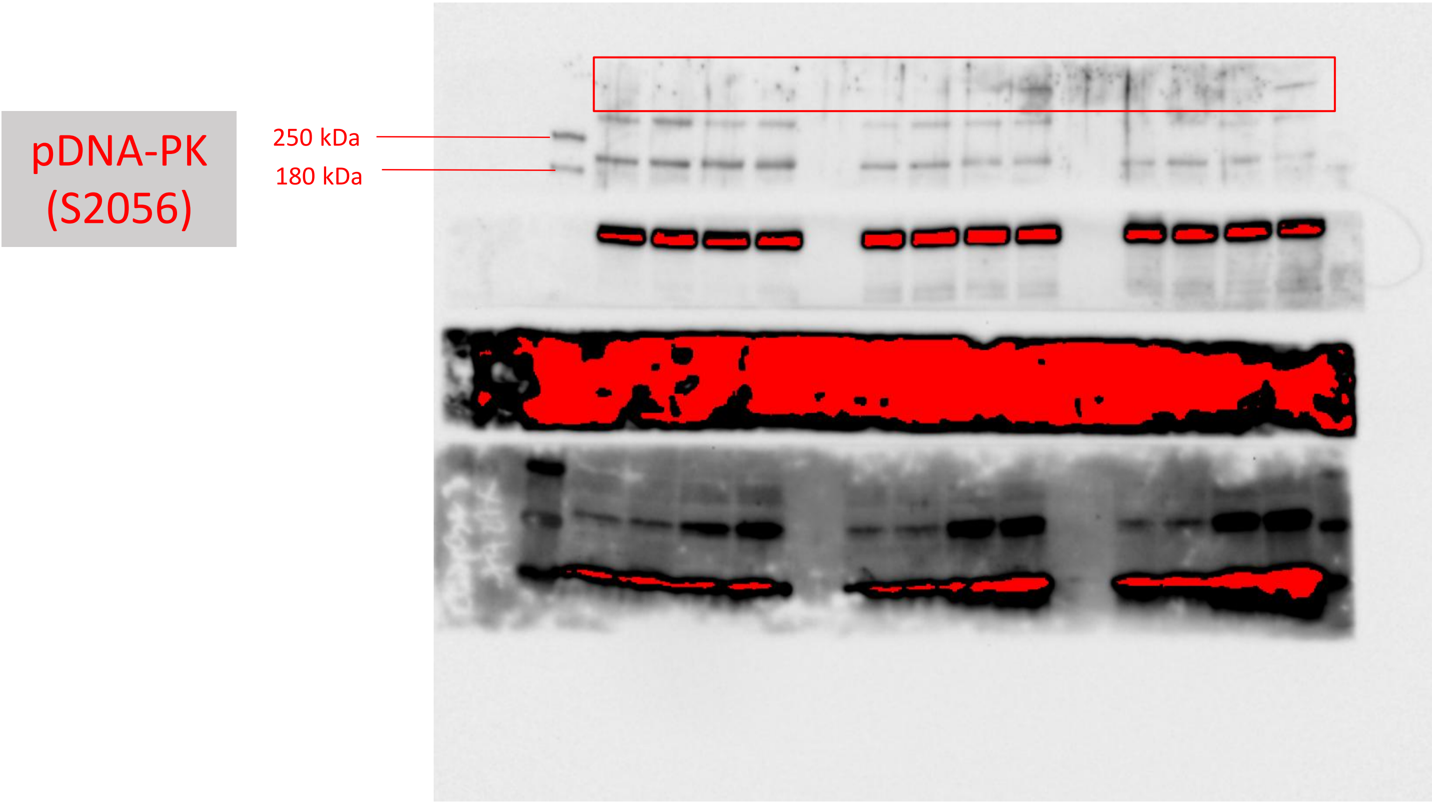

Colorimetric for pDNA-PK

pDNA-PK (S2056)

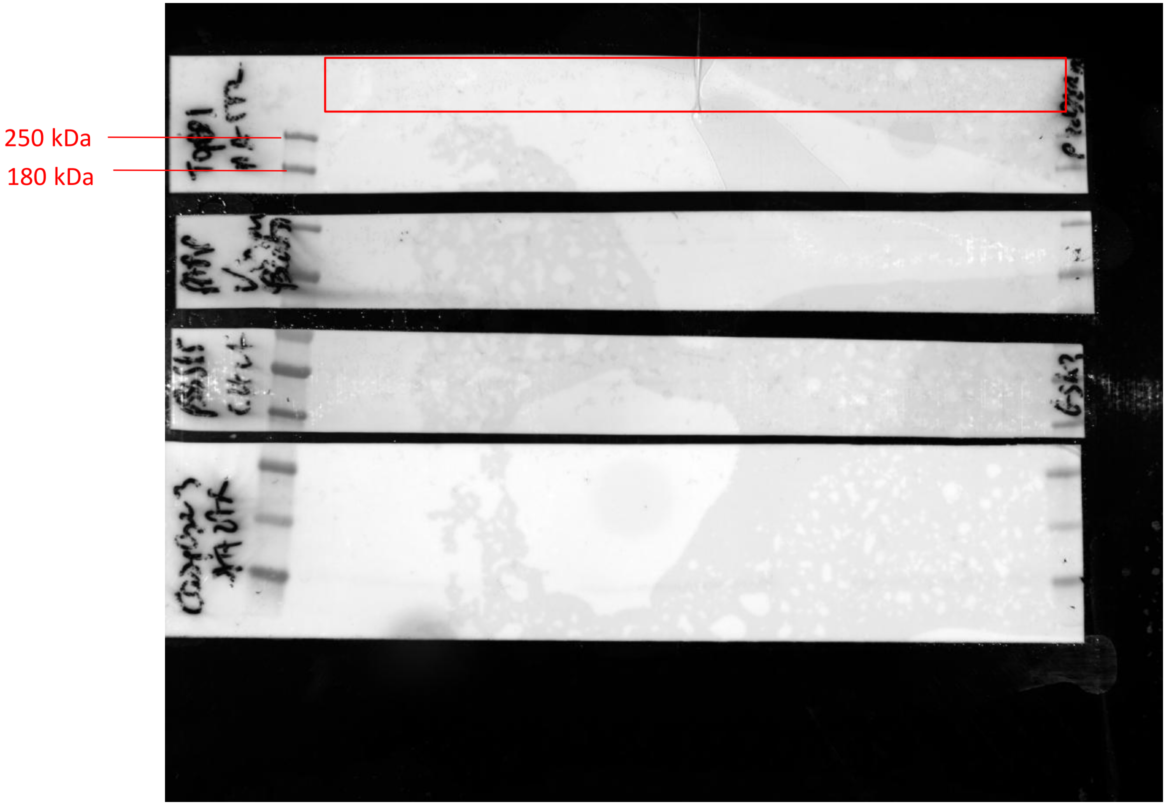

Merge chemiluminescence bands/colorimetric for TopBP1

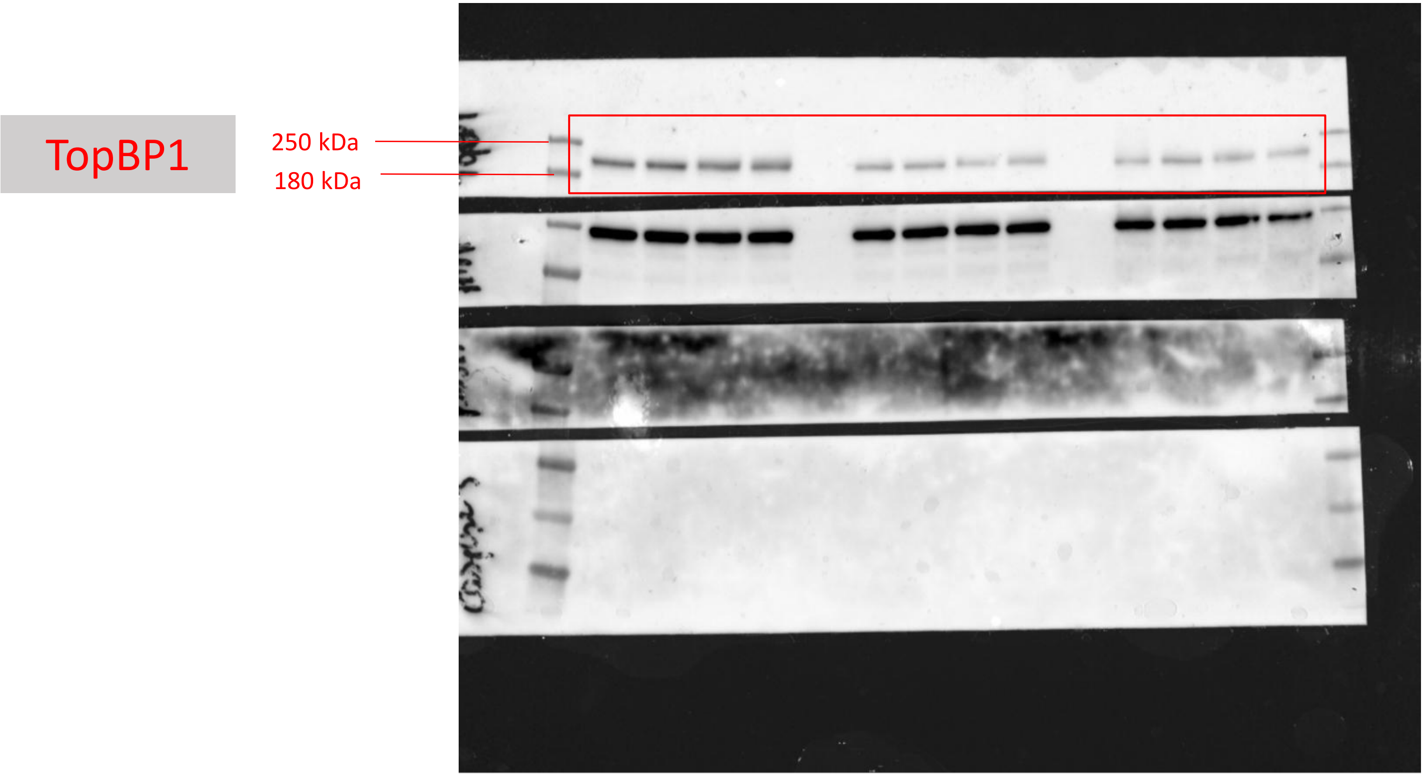

Chemiluminescence bands for ToPBP1

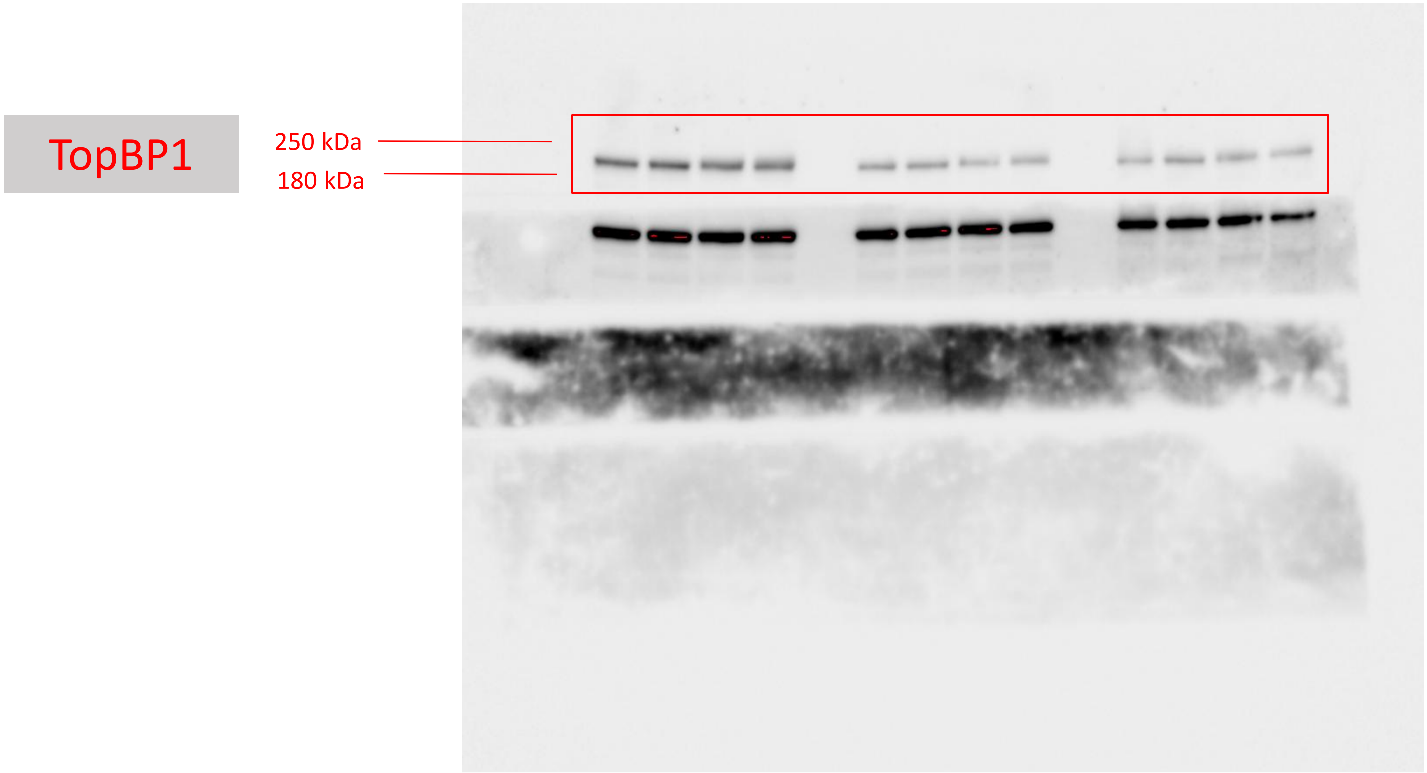

Merge chemiluminescence bands/colorimetric for PARP (low exp)

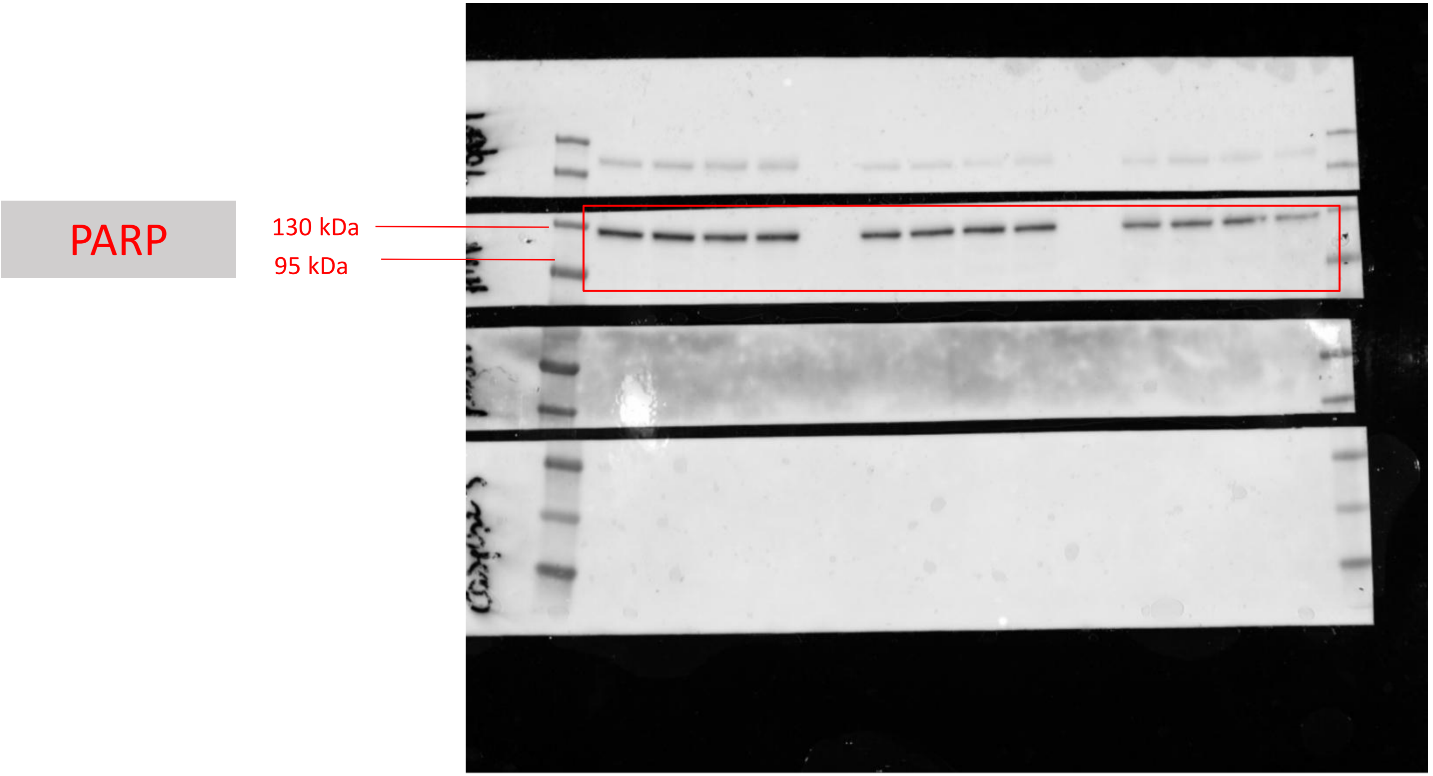

Chemiluminescence bands for PARP (low exp)

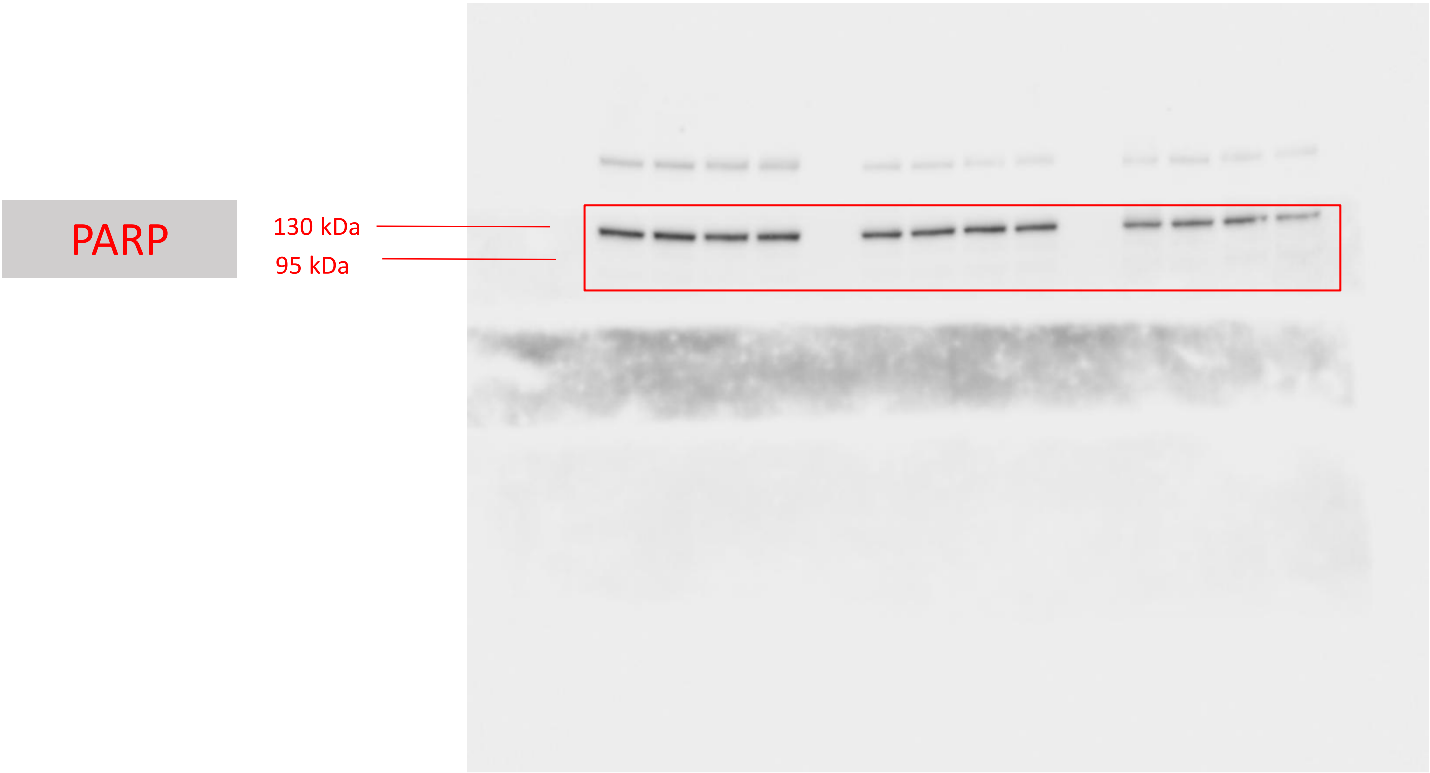

Merge chemiluminescence bands/colorimetric for PARP cleaved (high exp)

PARP

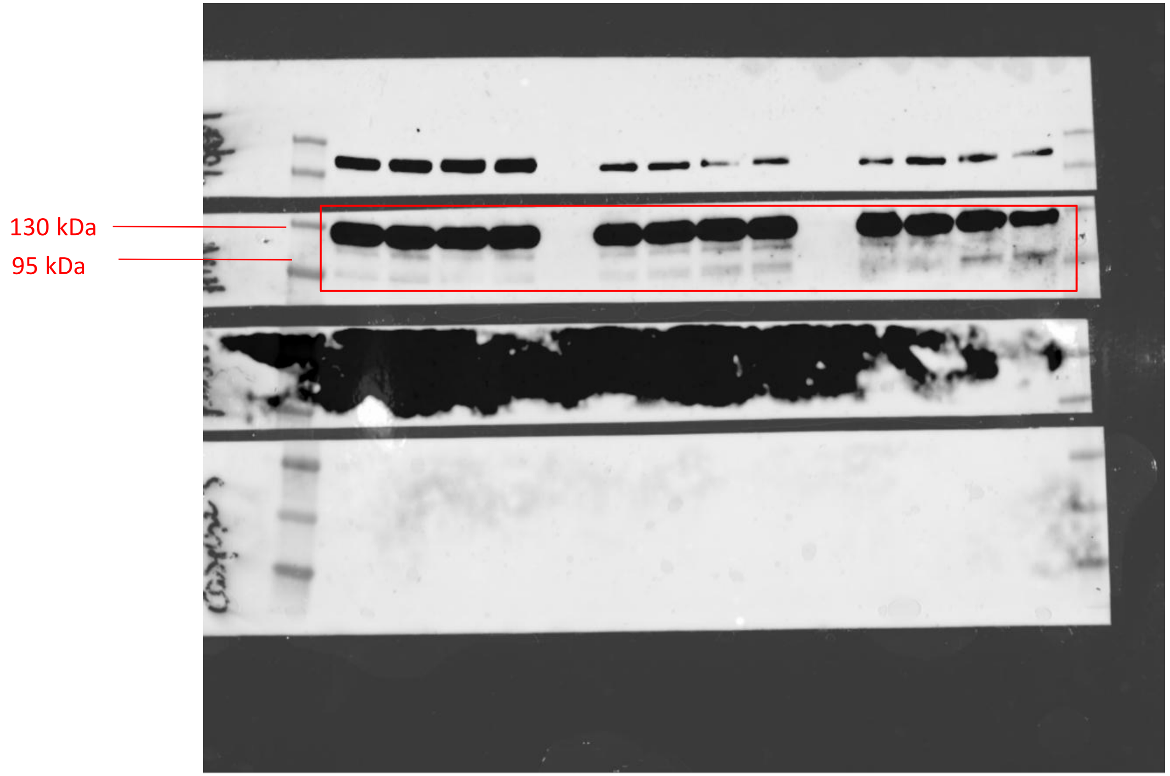

Chemiluminescence bands for PARP (high exp)

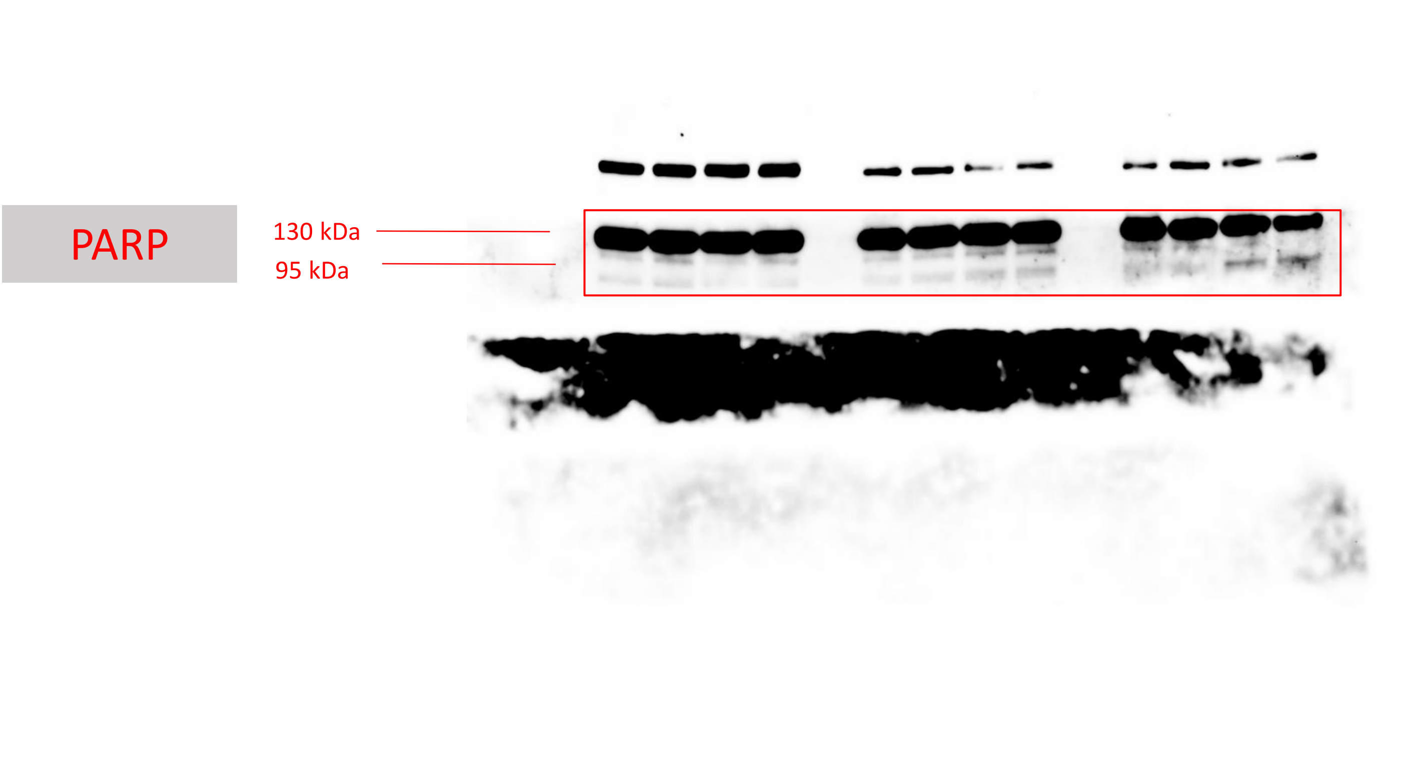

Colorimetric for TopBP1 & PARP

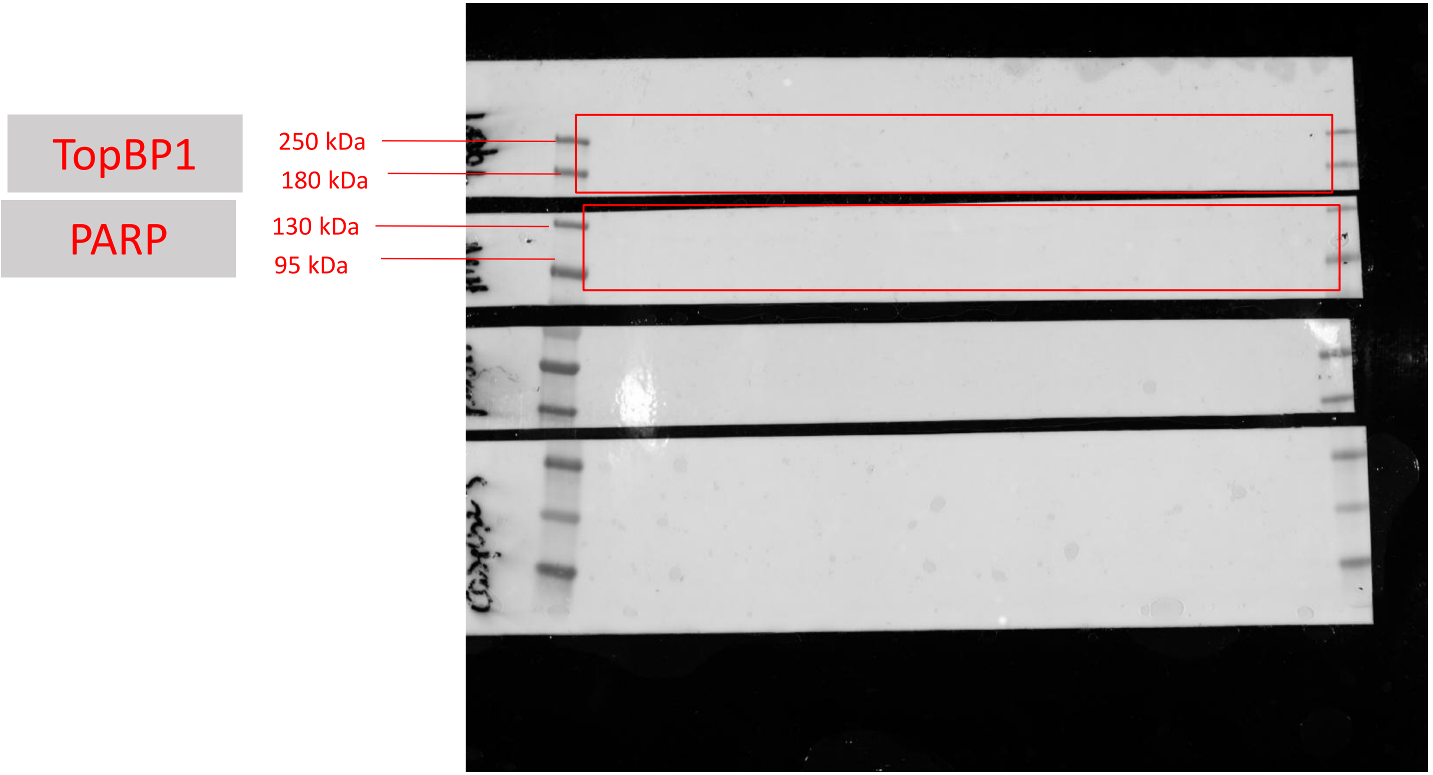

Colorimetric for pATR & γH2AX

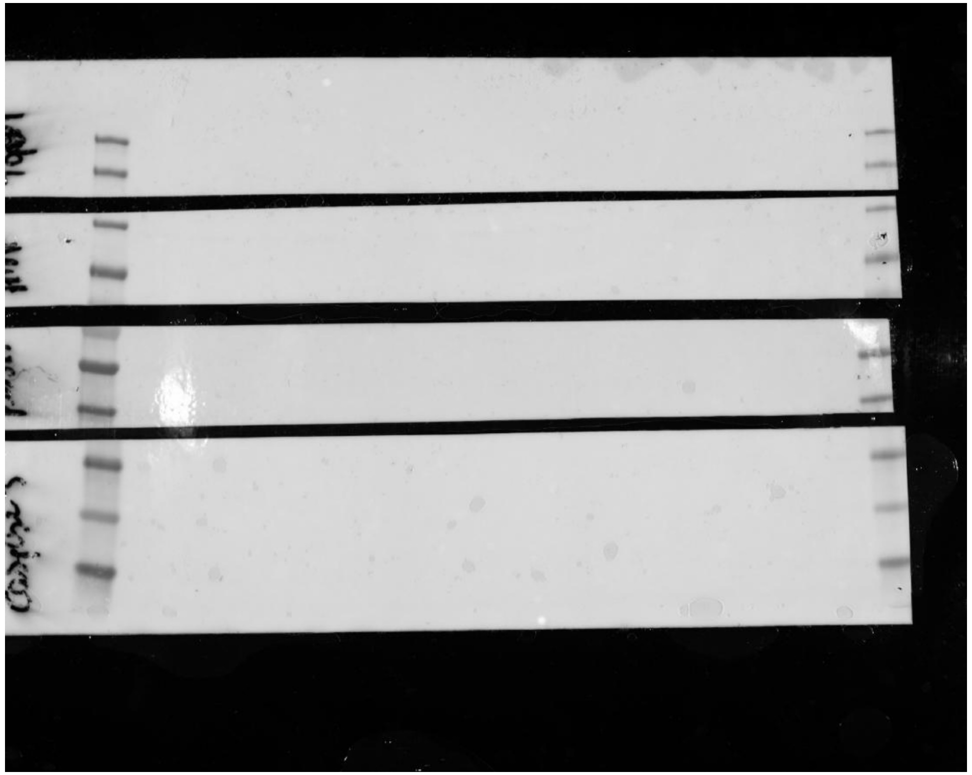

Merge chemiluminescence bands/colorimetric for CHK1 & Vinculin

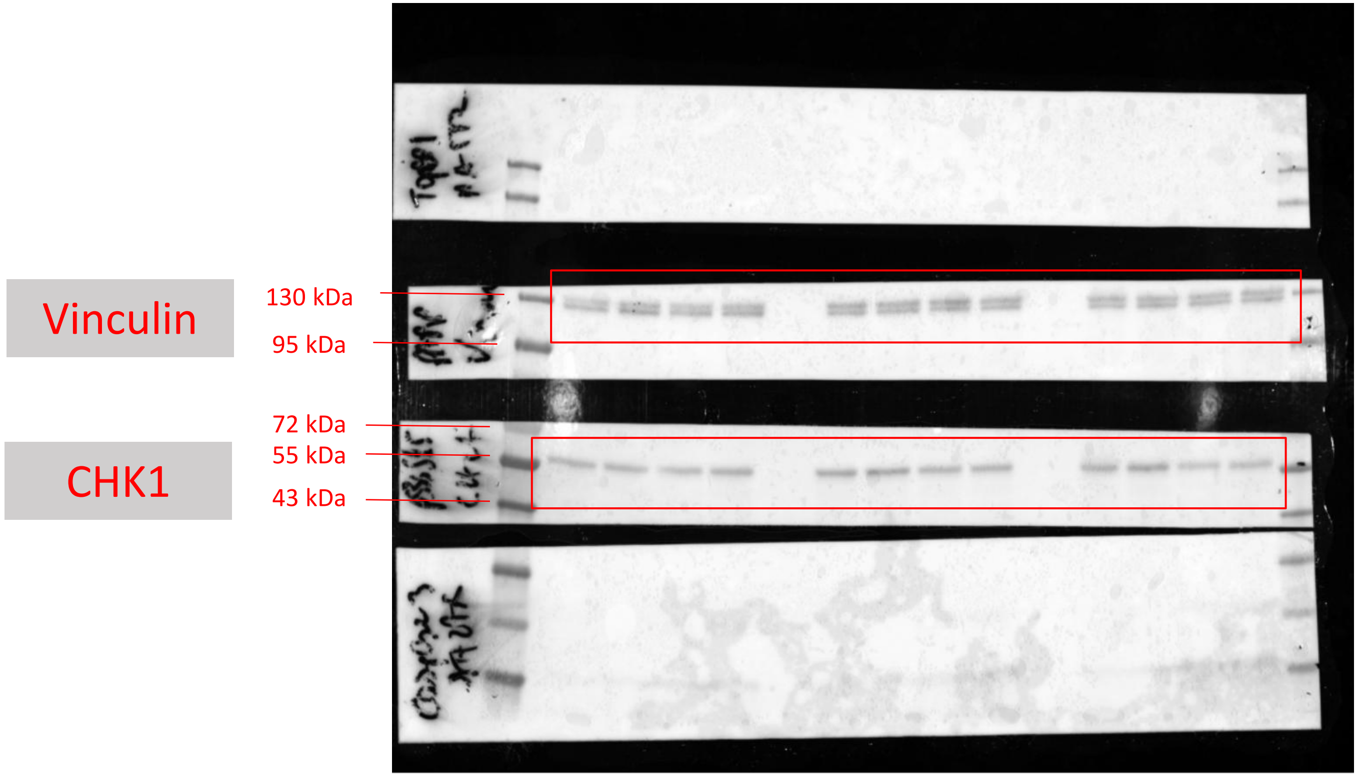

Chemiluminescence bands for CHK1 & Vinculin

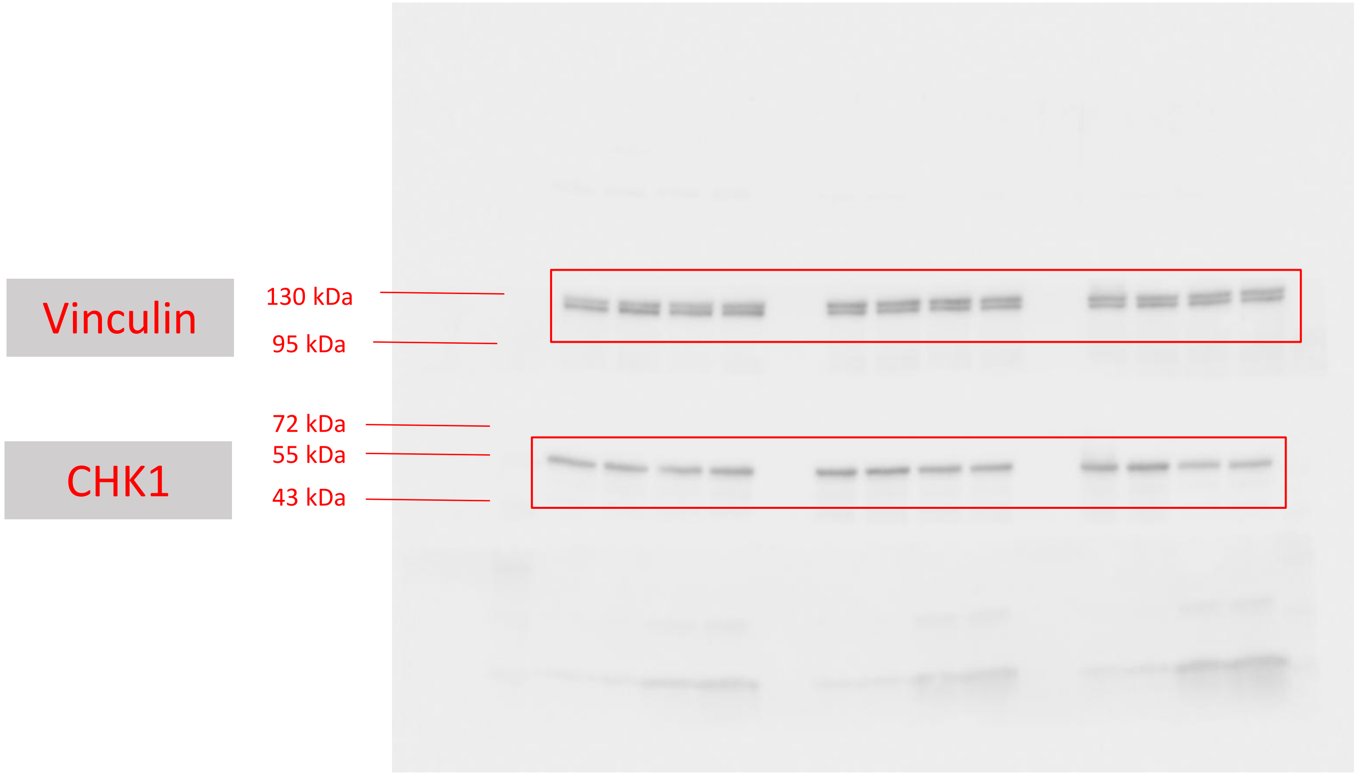

Merge chemiluminescence bands/colorimetric for γH2AX

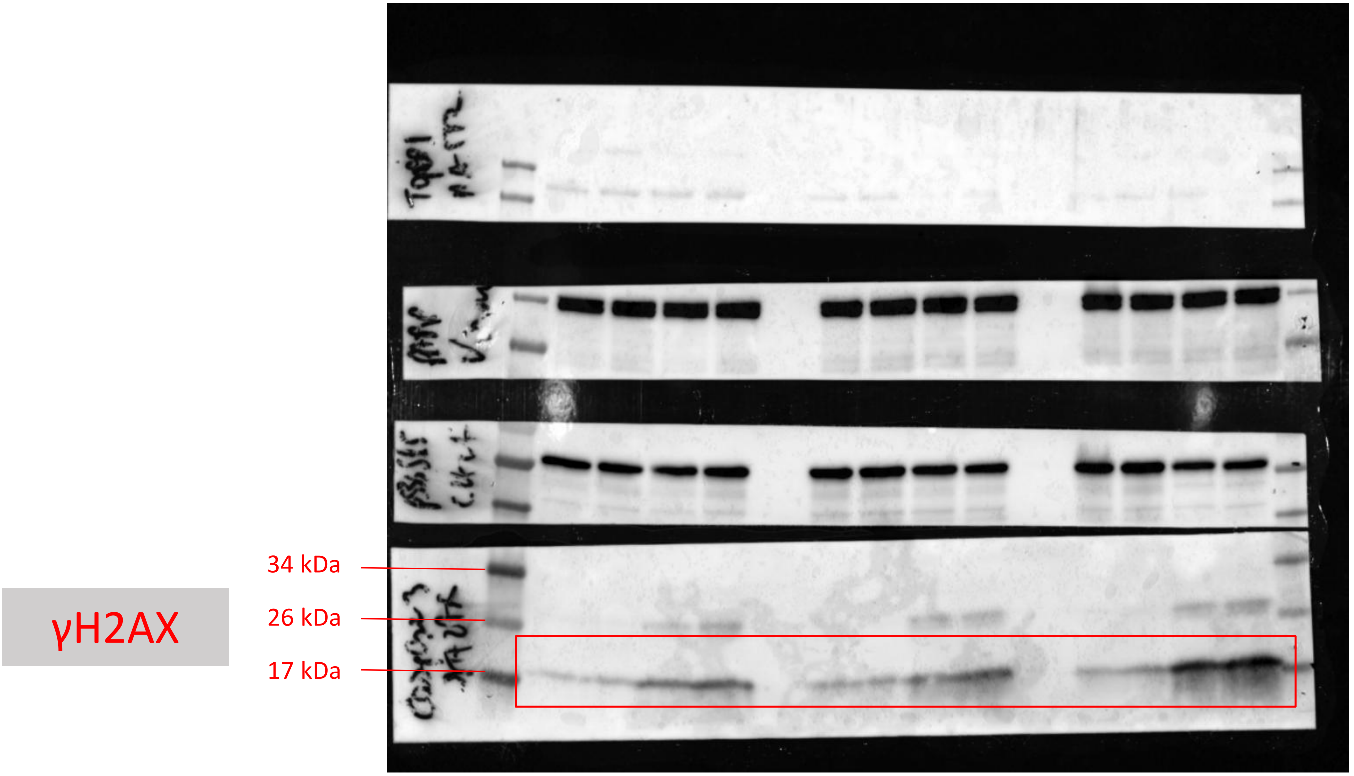

Chemiluminescence bands for γH2AX

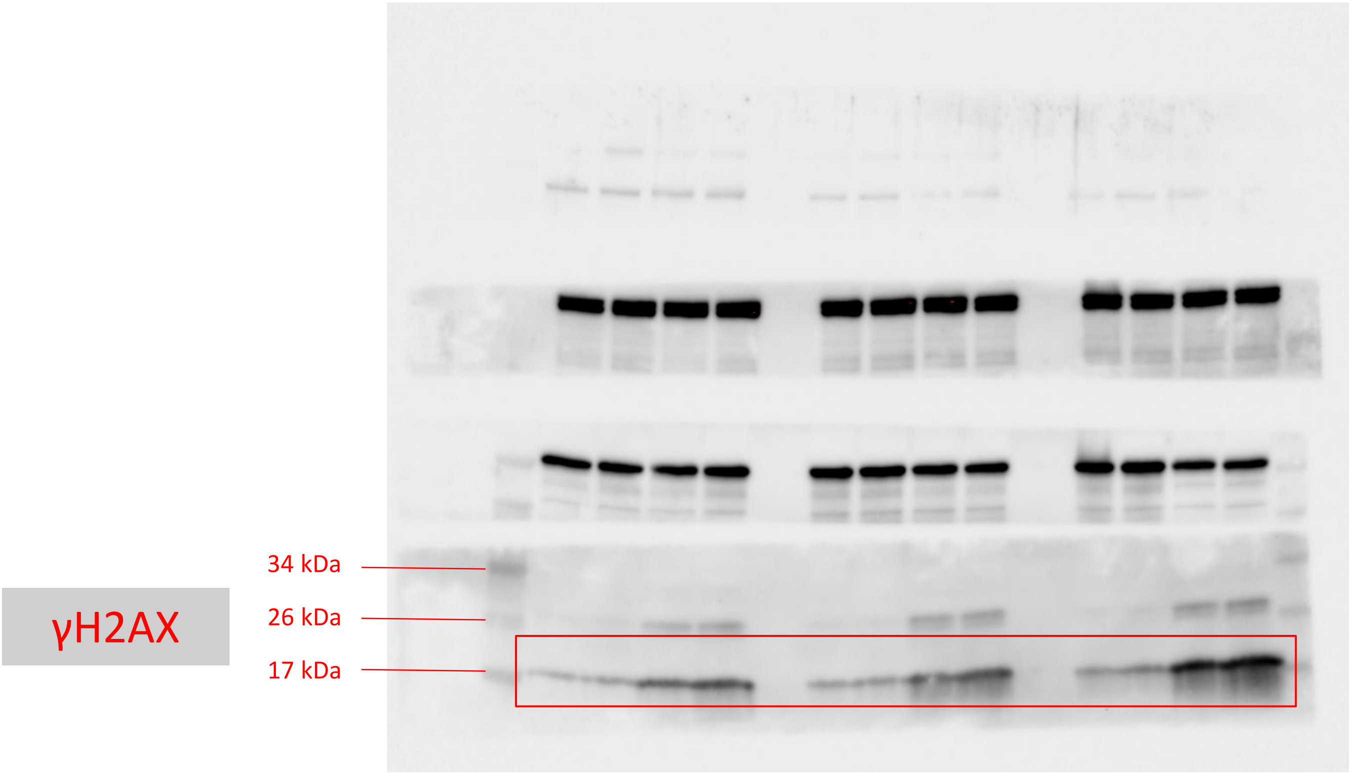

Colorimetric for CHK1, γH2AX, vinculin

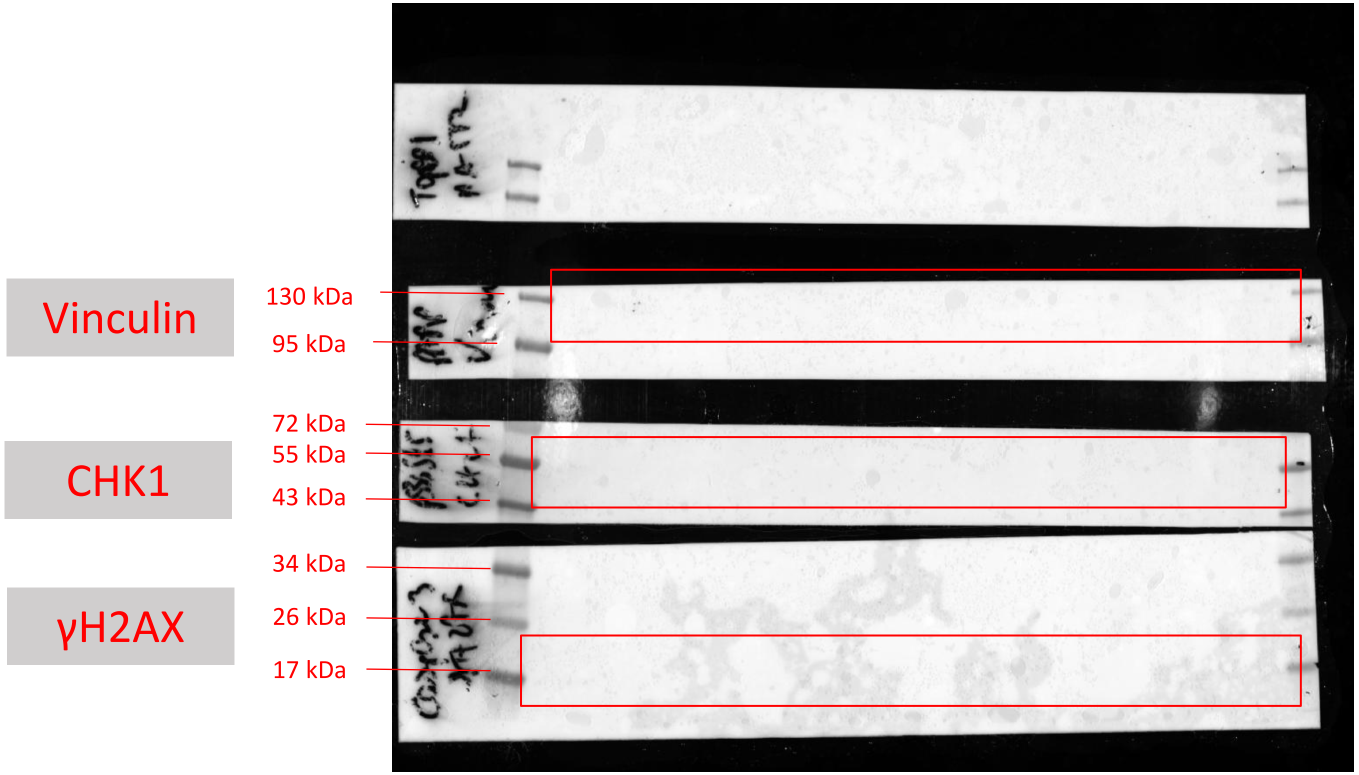

Supplement: Figure 3—source data 1. [file elife-106196-fig3-data1.zip › Fig 3D and E- Source Data 1/Fig 3E-Source Data 1.pdf]
