# Supplementary material for: TopBP1 biomolecular condensates as a new therapeutic target in advanced-stage colorectal cancer
Source: eLife. 2025 Oct 21;14:RP106196. doi: 10.7554/eLife.106196 (PMC12539802; doi:10.7554/eLife.106196)
Supplement: Figure 3—source data 1. [file elife-106196-fig3-data1.zip › Fig 3D and E- Source Data 1/Fig3D-Source Data 1.pdf]

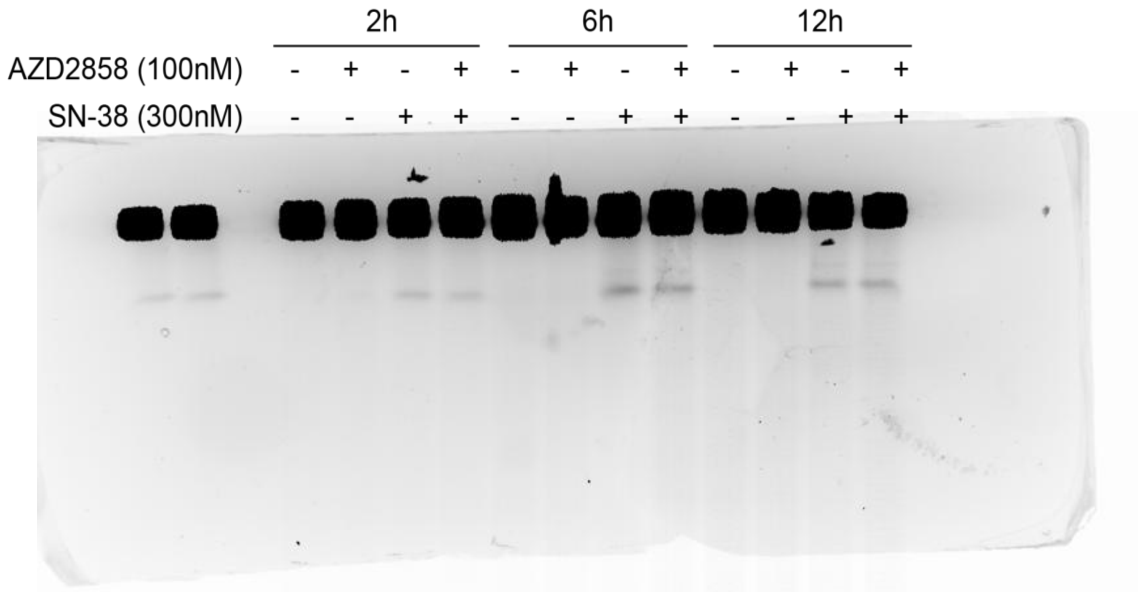


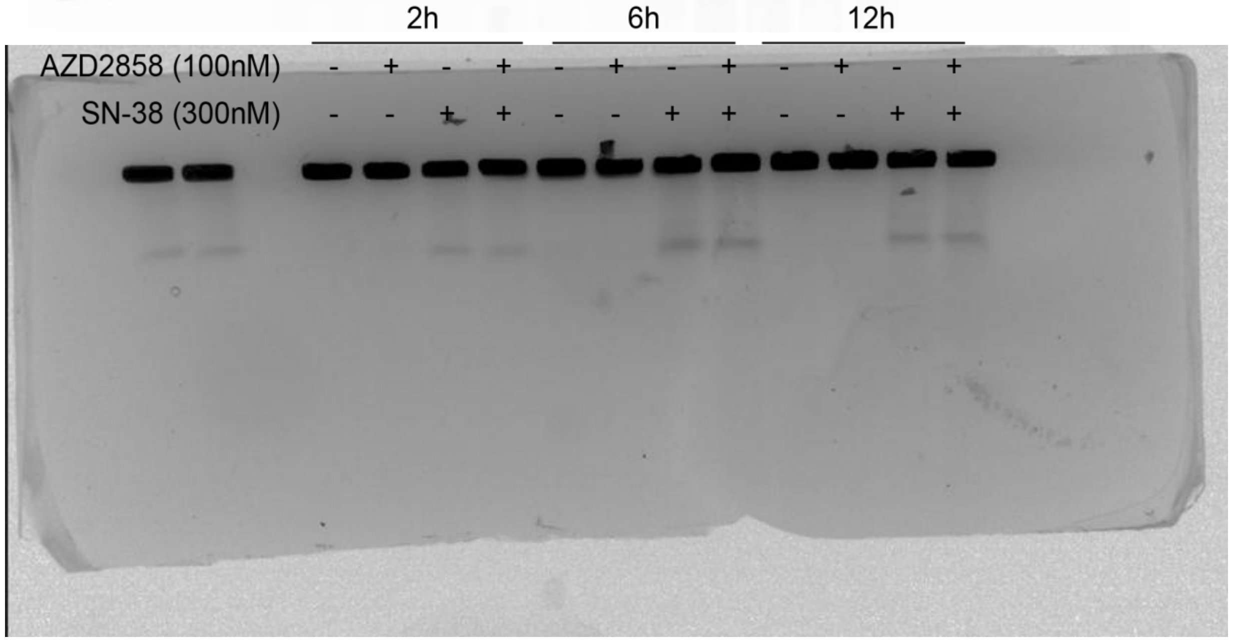


**Figure 3, Source Data 1.** Original membranes corresponding to Figure 3D

PFGE analysis of DNA damage in HCT116 cells incubated with AZD2858 (100 nM) and/or SN-38 (300 nM) for the indicated times. The experiment was replicated 3 times, and a representative replicate is shown
