# Supplementary material for: TopBP1 biomolecular condensates as a new therapeutic target in advanced-stage colorectal cancer
Source: eLife. 2025 Oct 21;14:RP106196. doi: 10.7554/eLife.106196 (PMC12539802; doi:10.7554/eLife.106196)

Figure 4.C

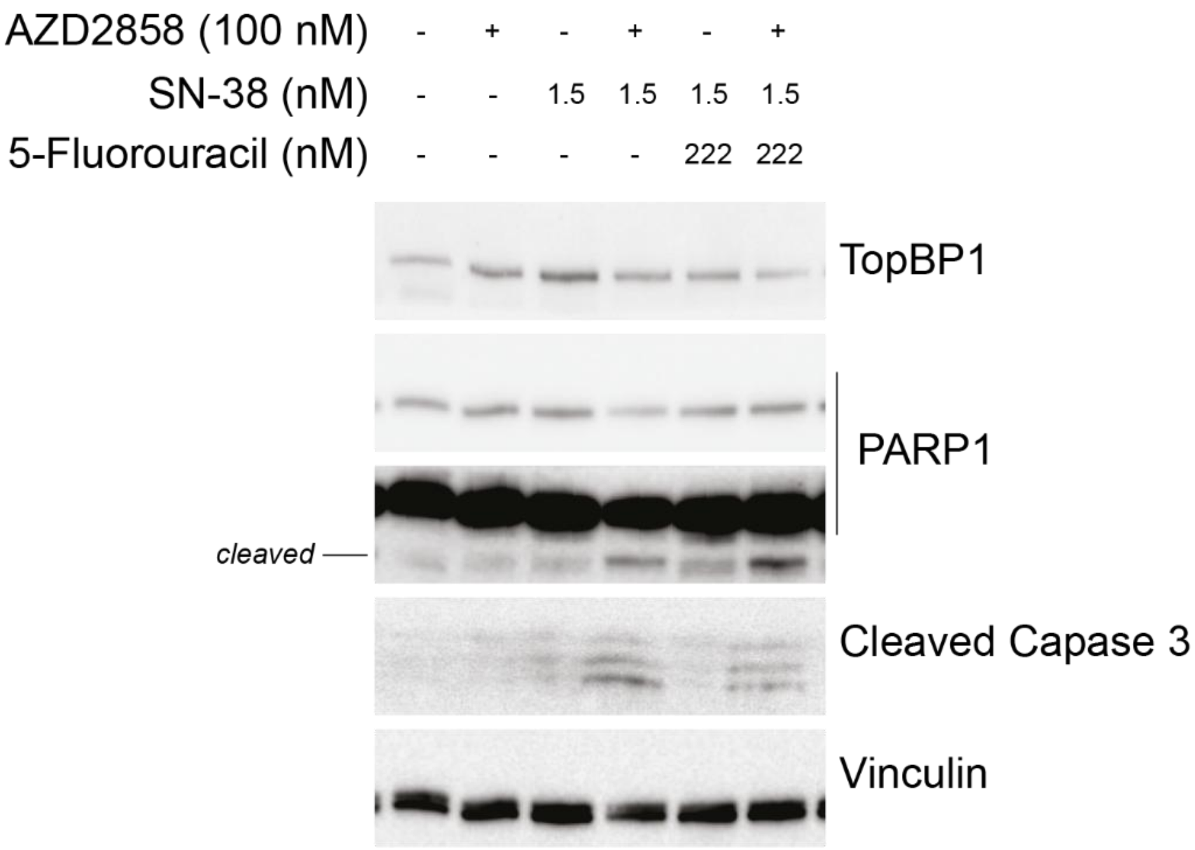

Figure 4C, Source Data 1. Below are the Original membranes corresponding to Figure 4C.
Immunoblot of the indicated proteins in HCT116 cells after 48-h incubation as described in Figure 4A.

Merge chemiluminescence bands/colorimetric for Vinculin

Vinculin

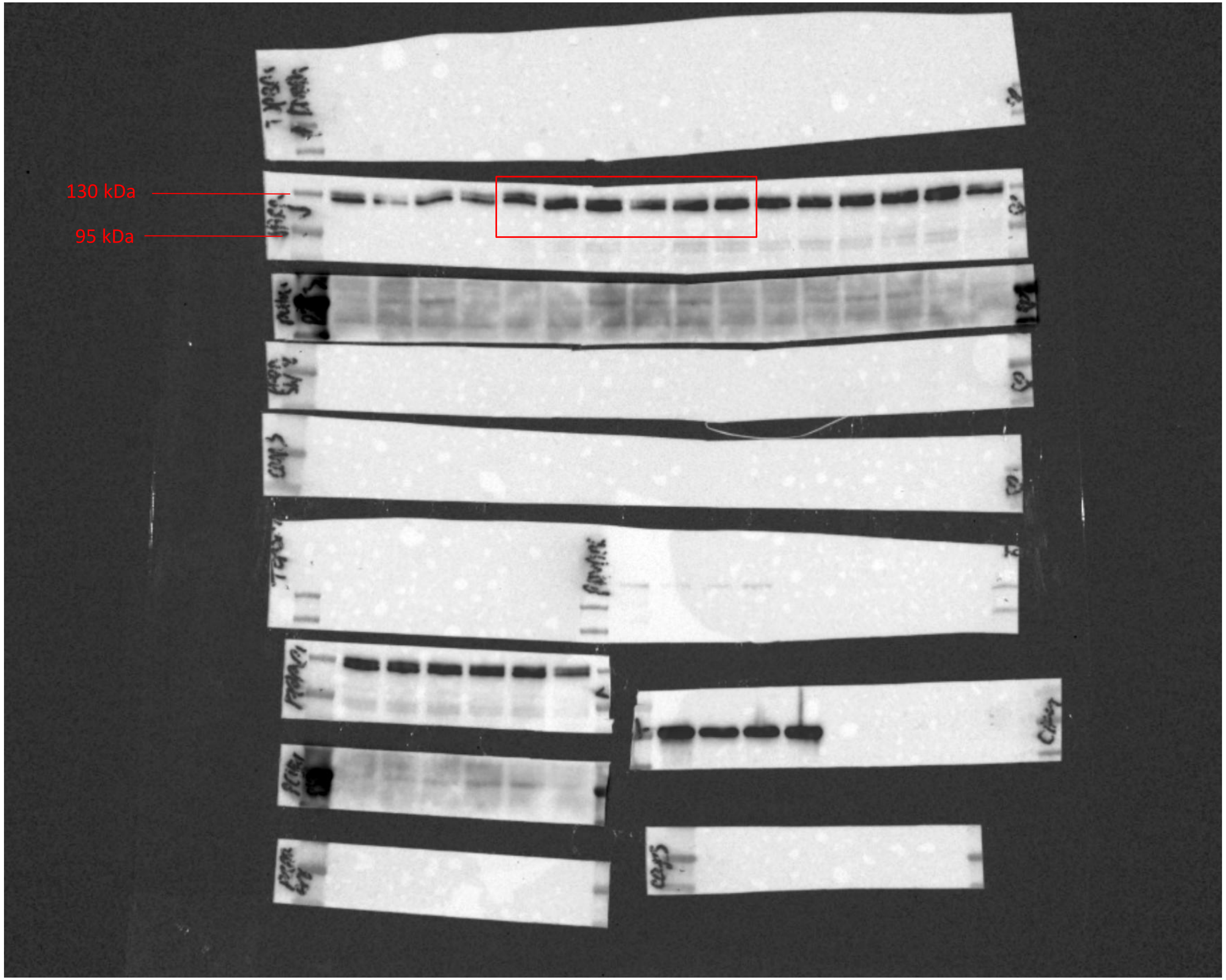

Chemiluminescence bands for vinculin

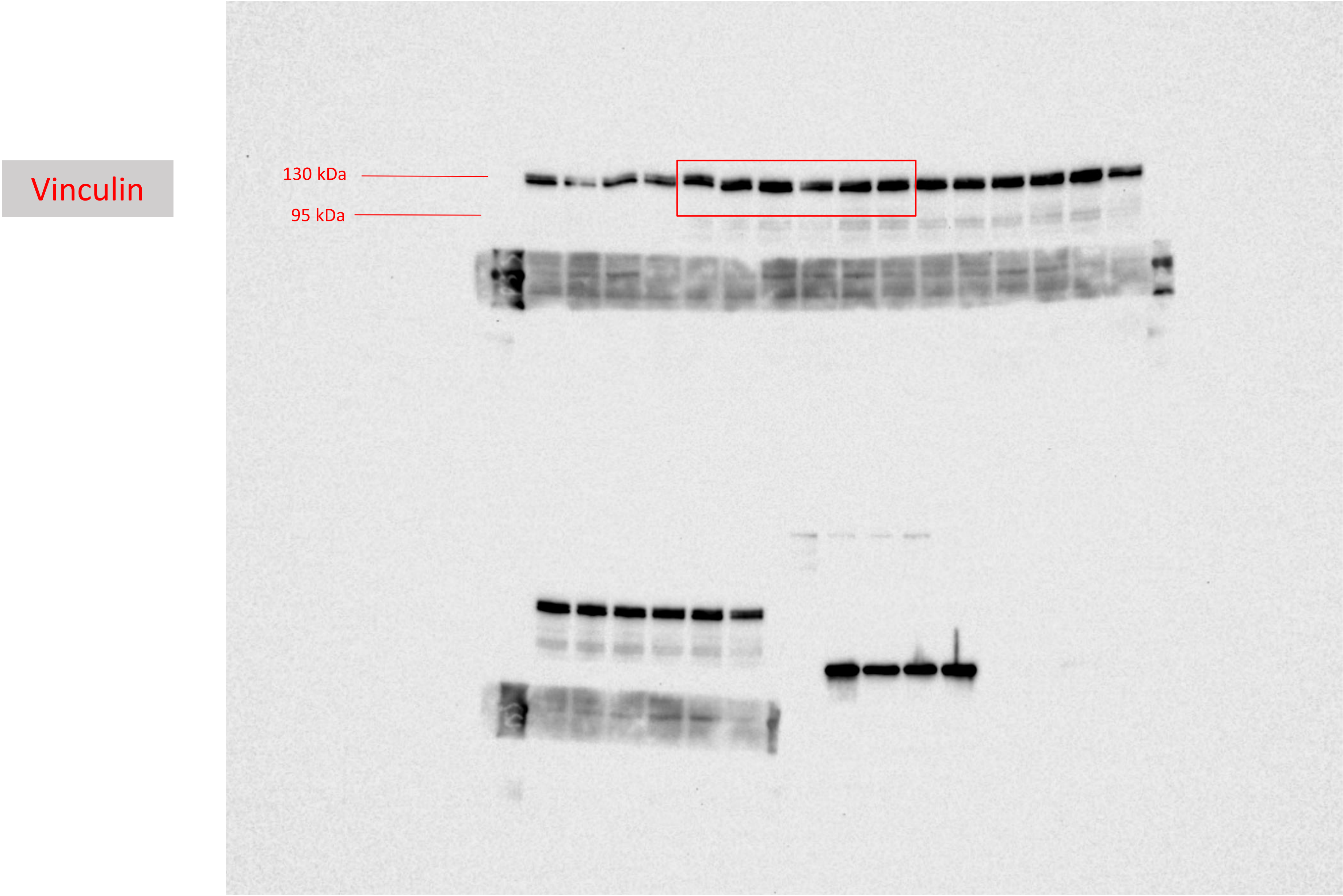

Colorimetric for Vinculin

Vinculin

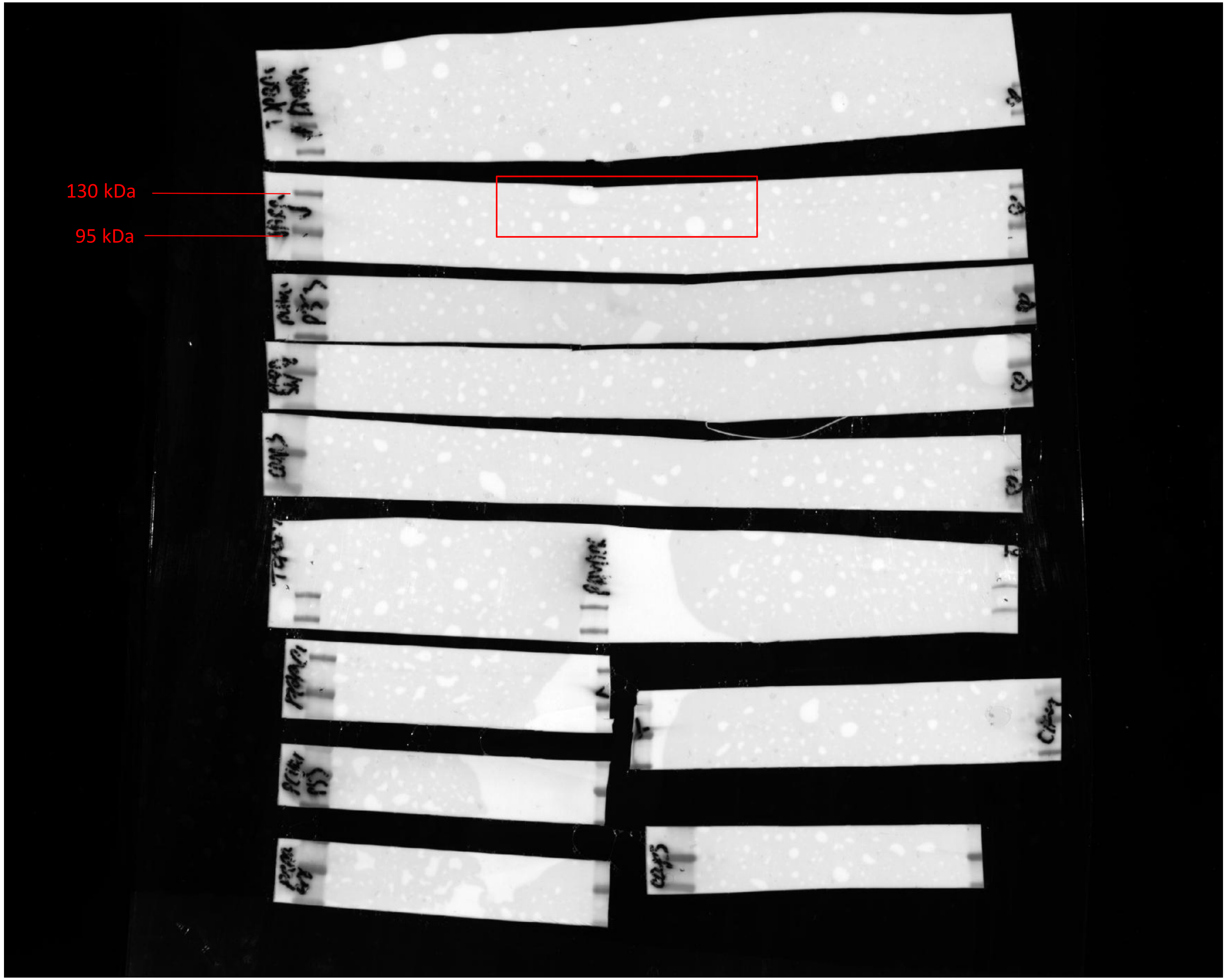

Merge chemiluminescence bands/colorimetric for TopBP1

TopBP1

250 kDa

180 kDa

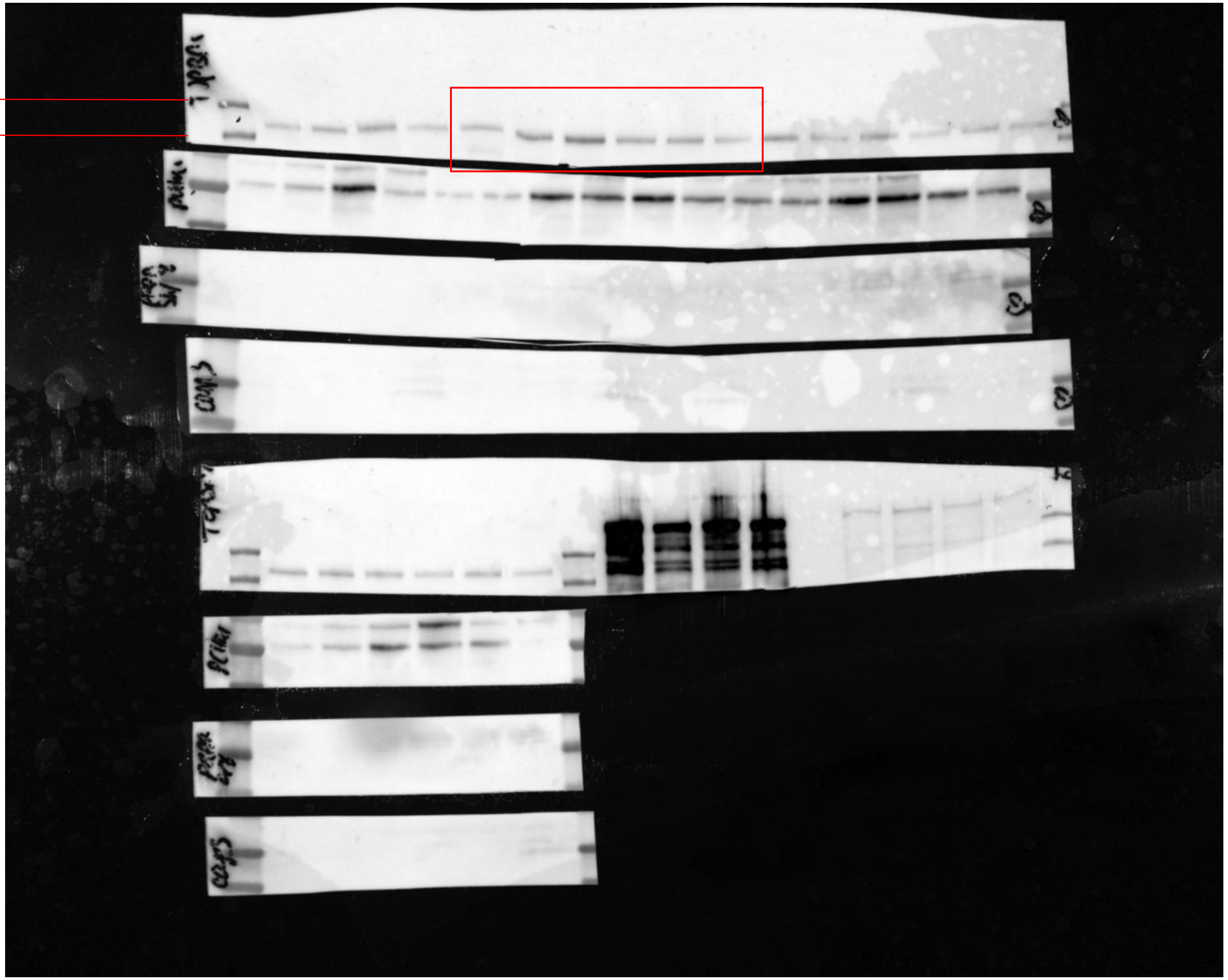

Chemiluminescence bands for ToPBP1

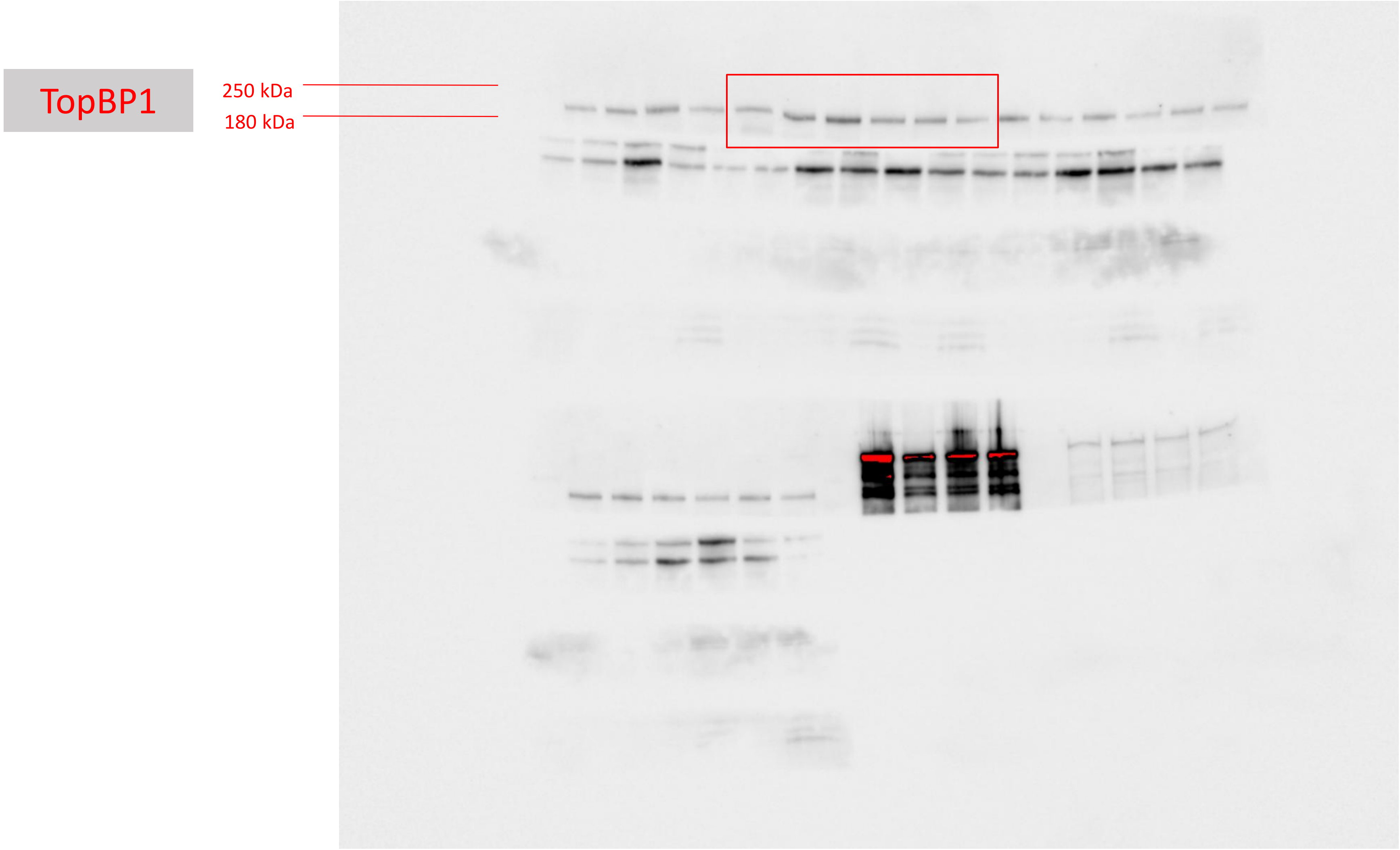

Merge chemiluminescence bands/colorimetric for cleaved caspase-3

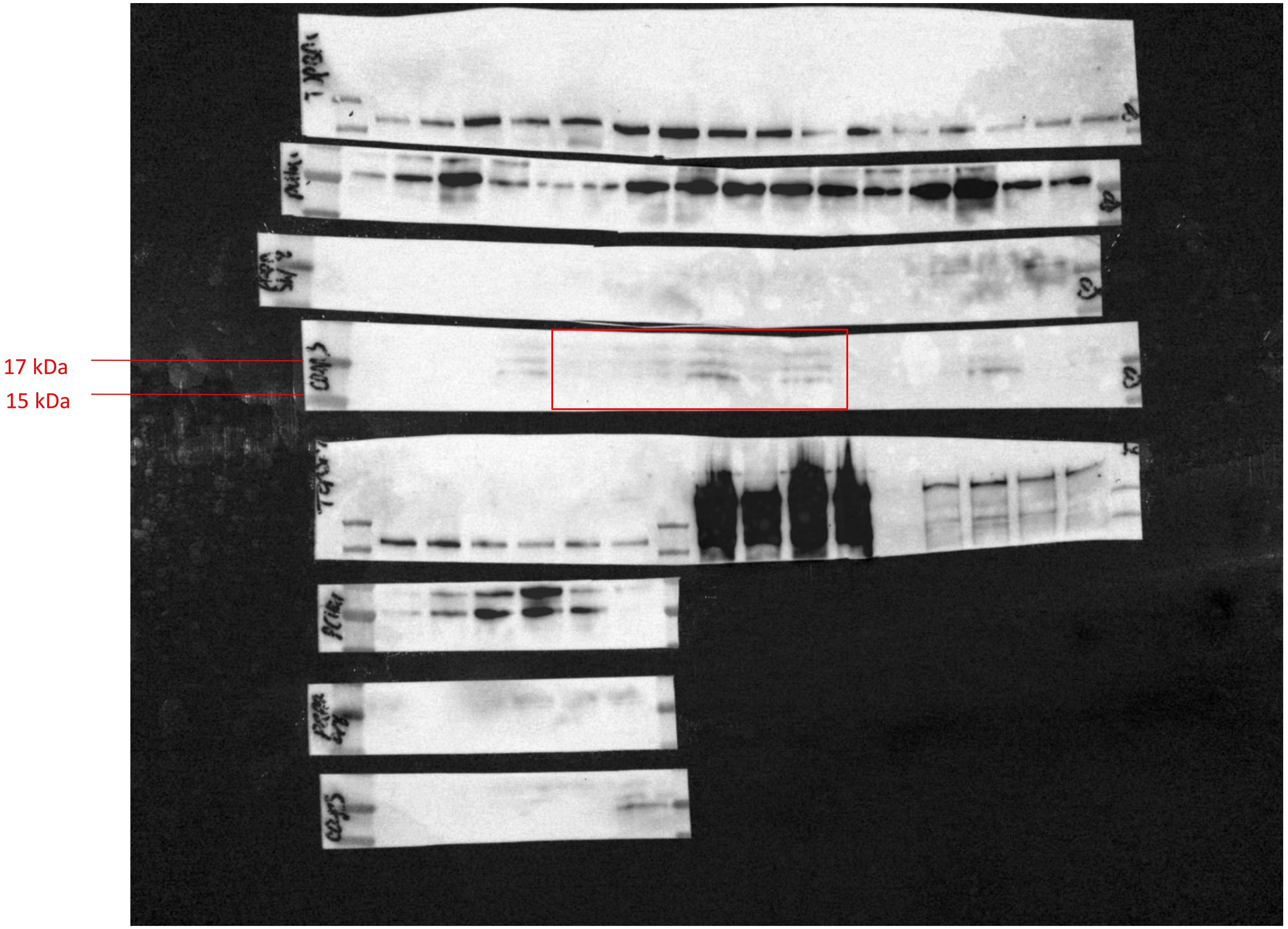

Cleaved caspase3

17 kDa

15 kDa

Chemiluminescence bands for cleaved caspase 3

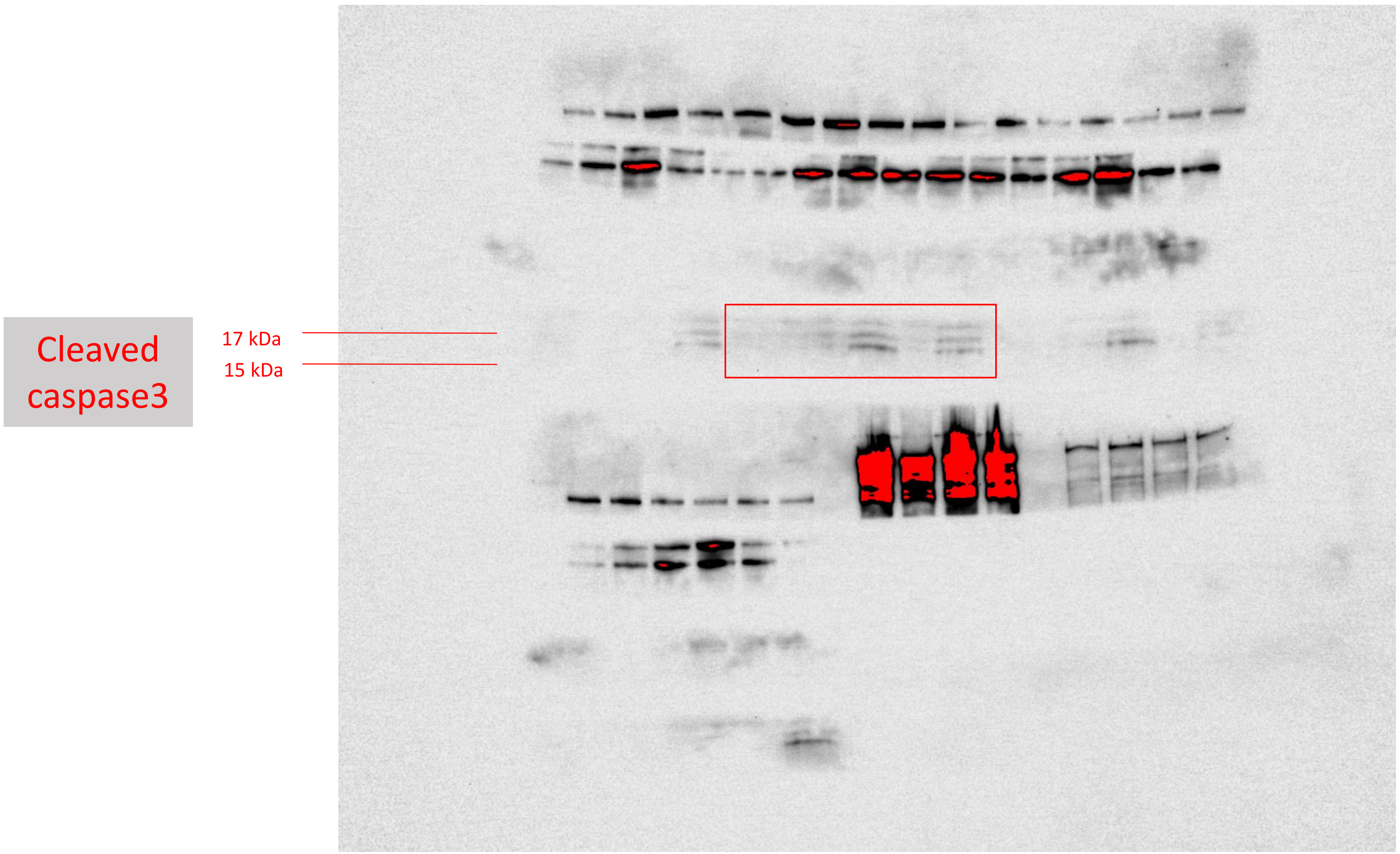

Colorimetric for TopBP1 & cleaved caspase 3

TopBP1

250 kDa

180 kDa

Cleaved caspase3

17 kDa

15 kDa

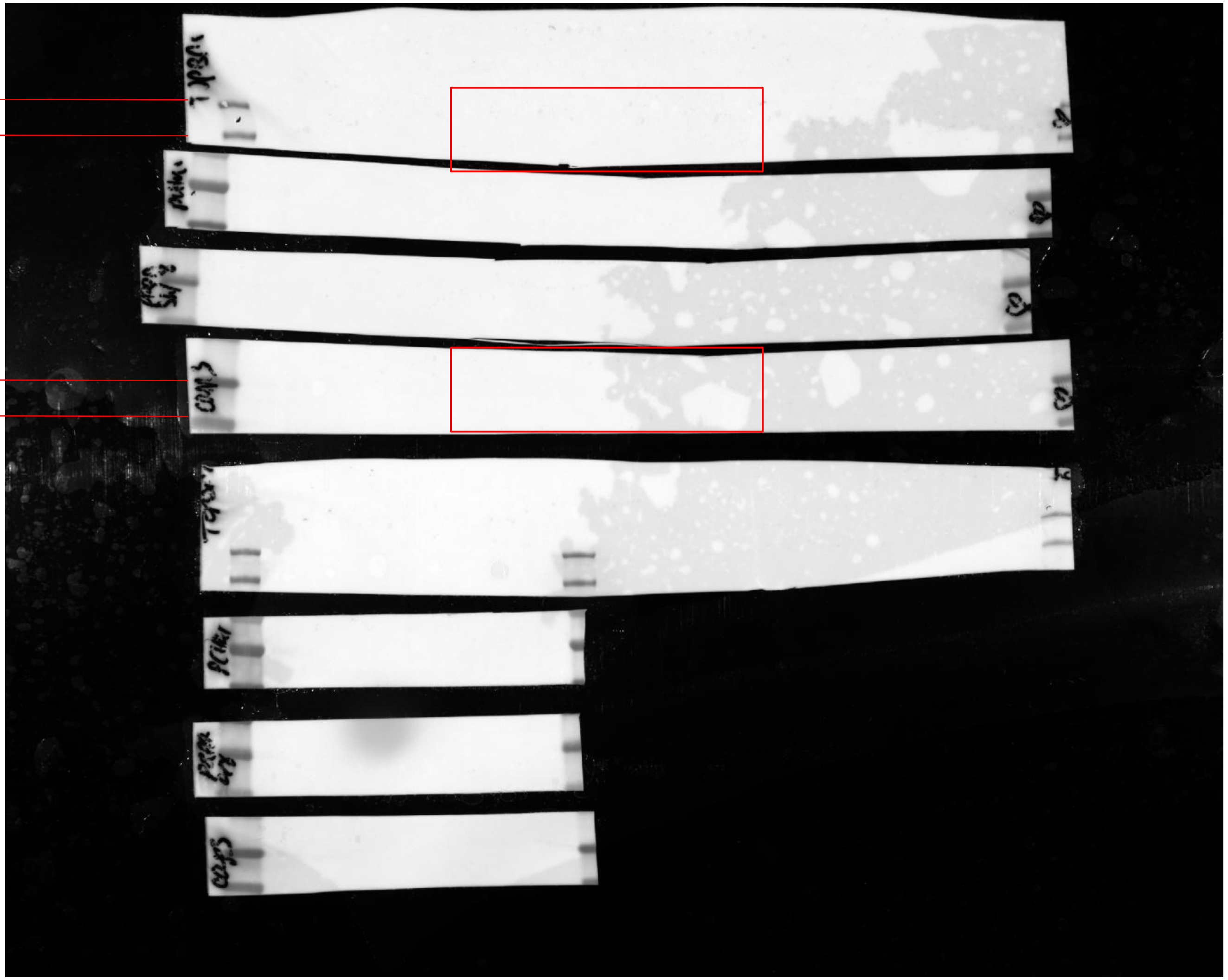

Merge chemiluminescence bands/colorimetric for PARP1 (low)

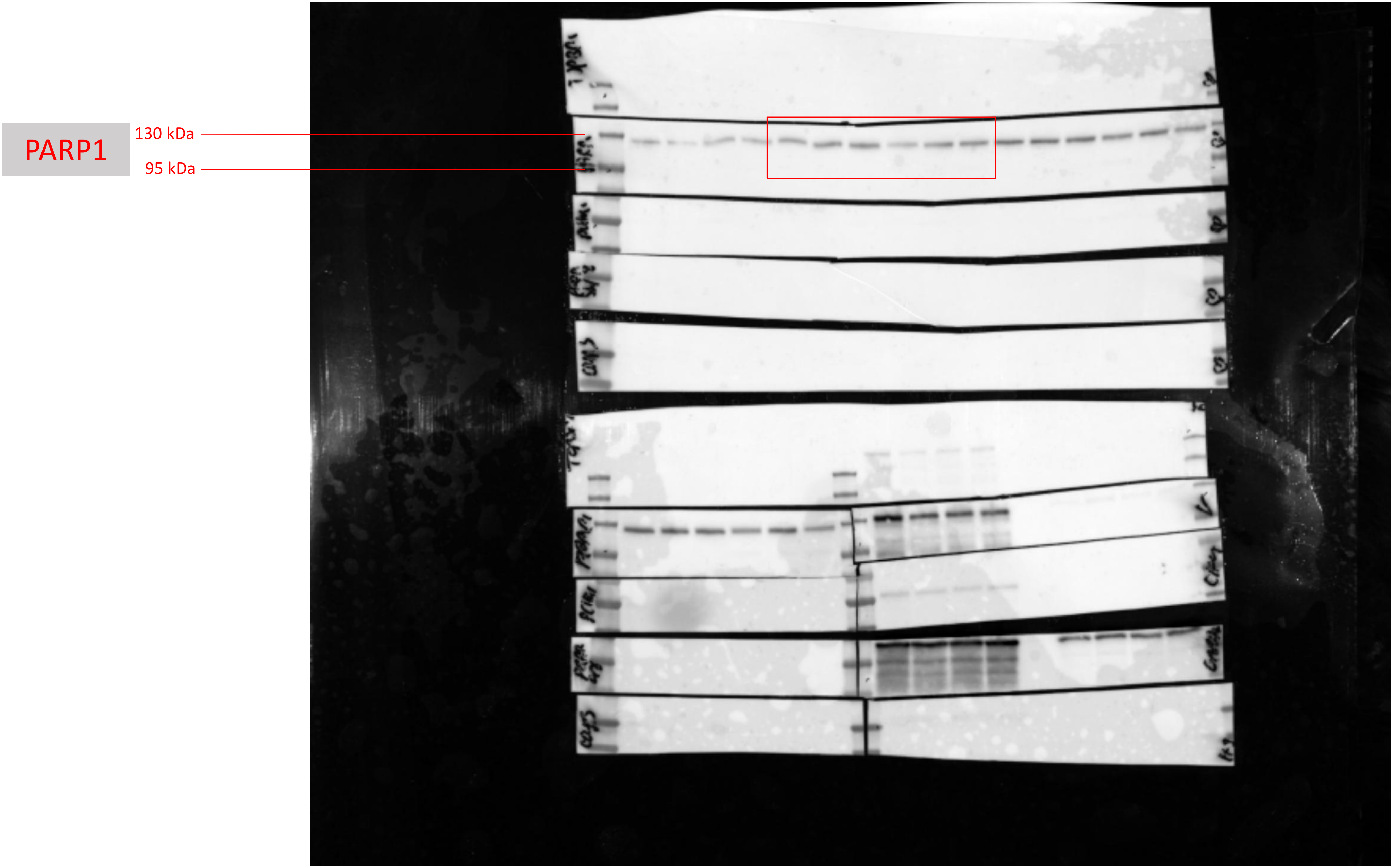

chemiluminescence for PARP1 (low)

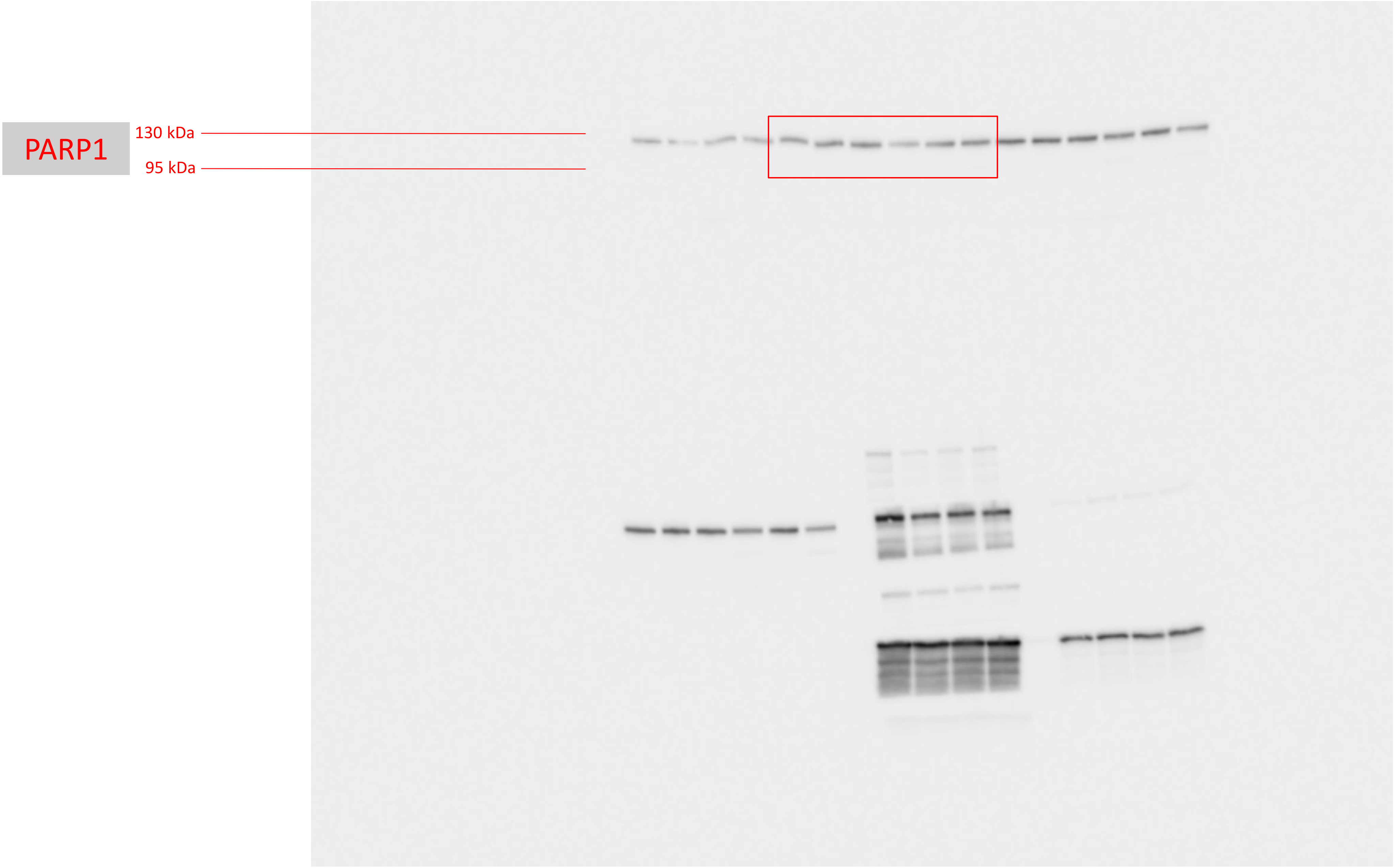

Merge chemiluminescence bands/colorimetric for PARP1 (high -> cleaved)

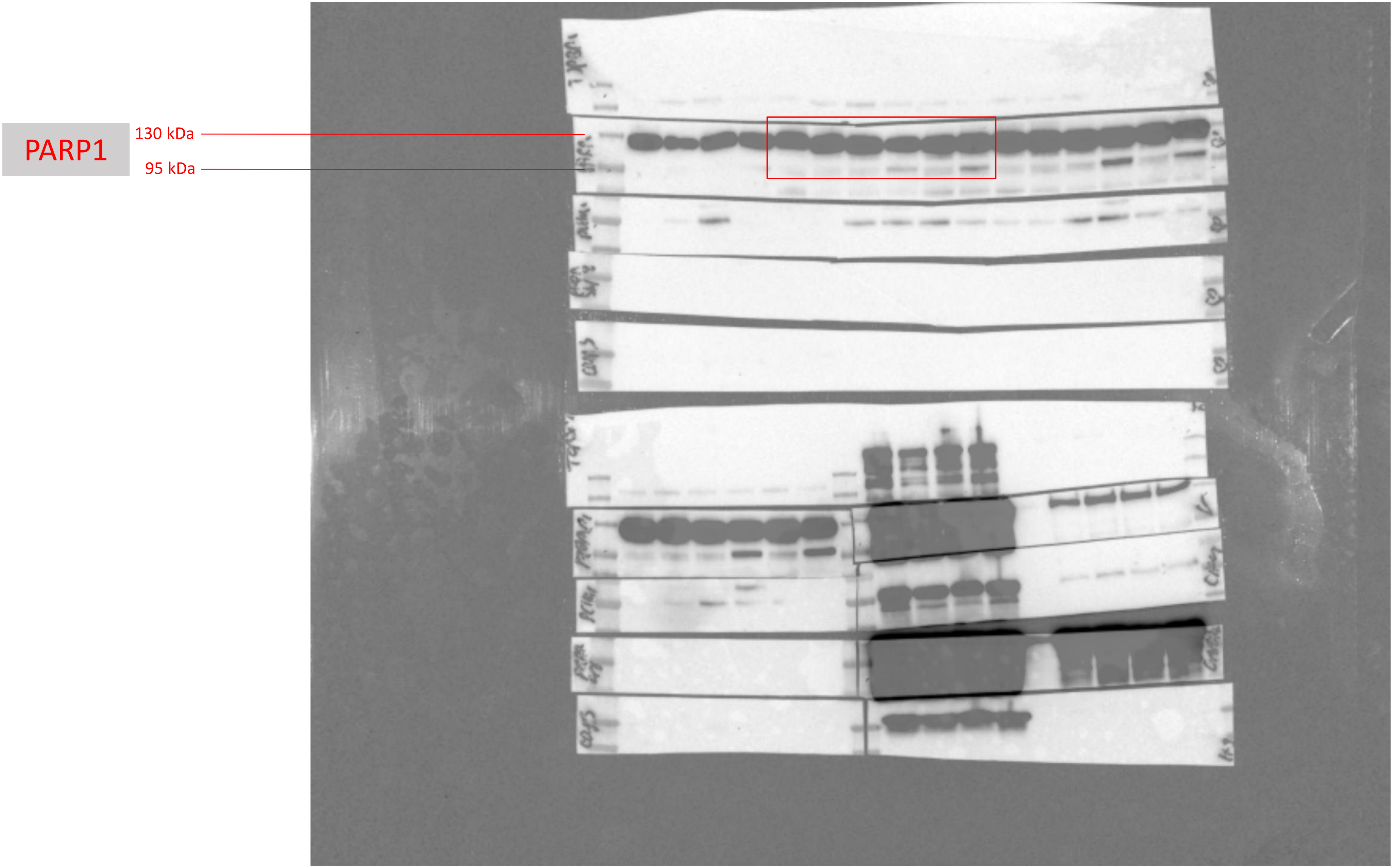

chemiluminescence for PARP1 (high -> cleaved)

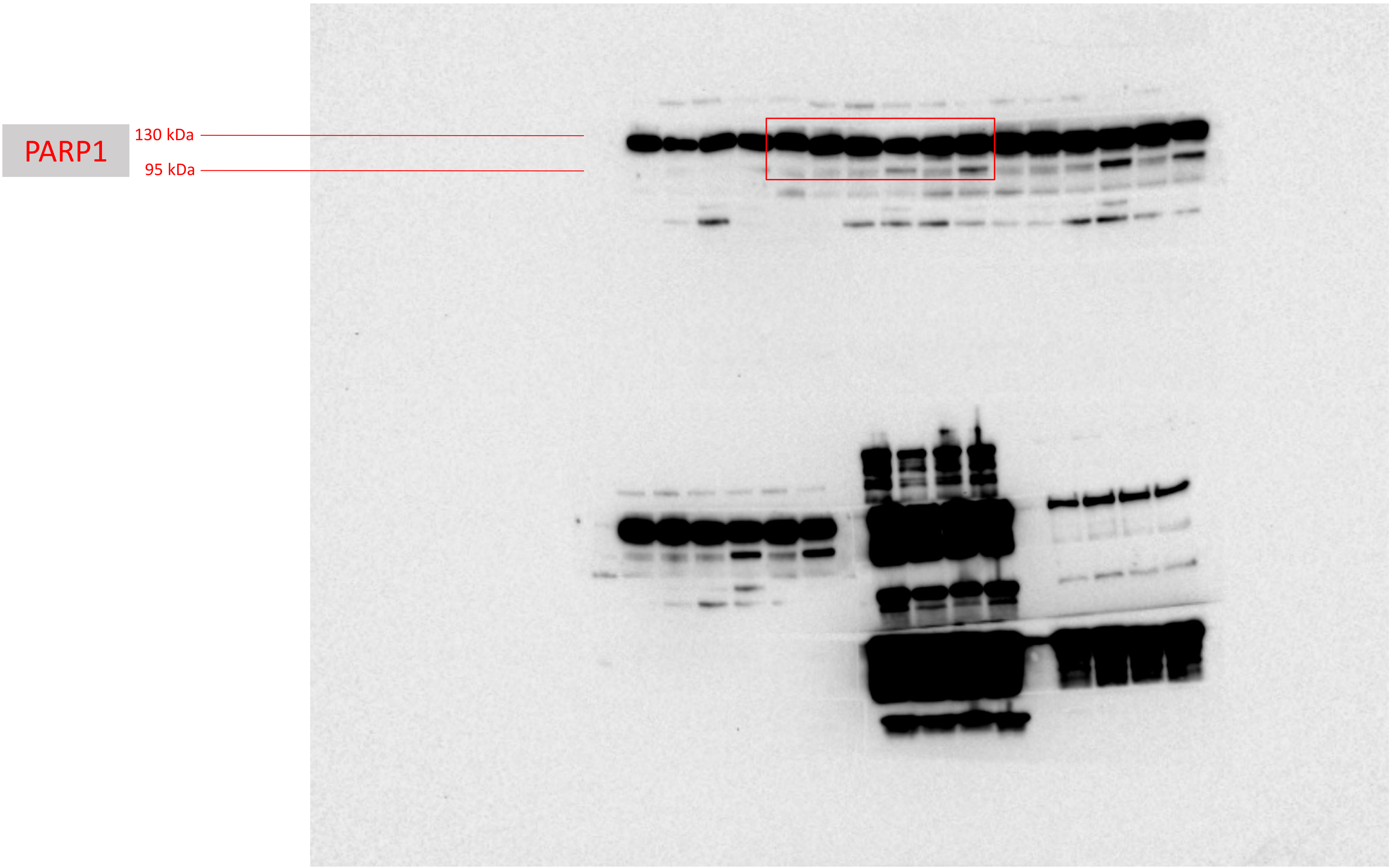

Colorimetric for PARP1

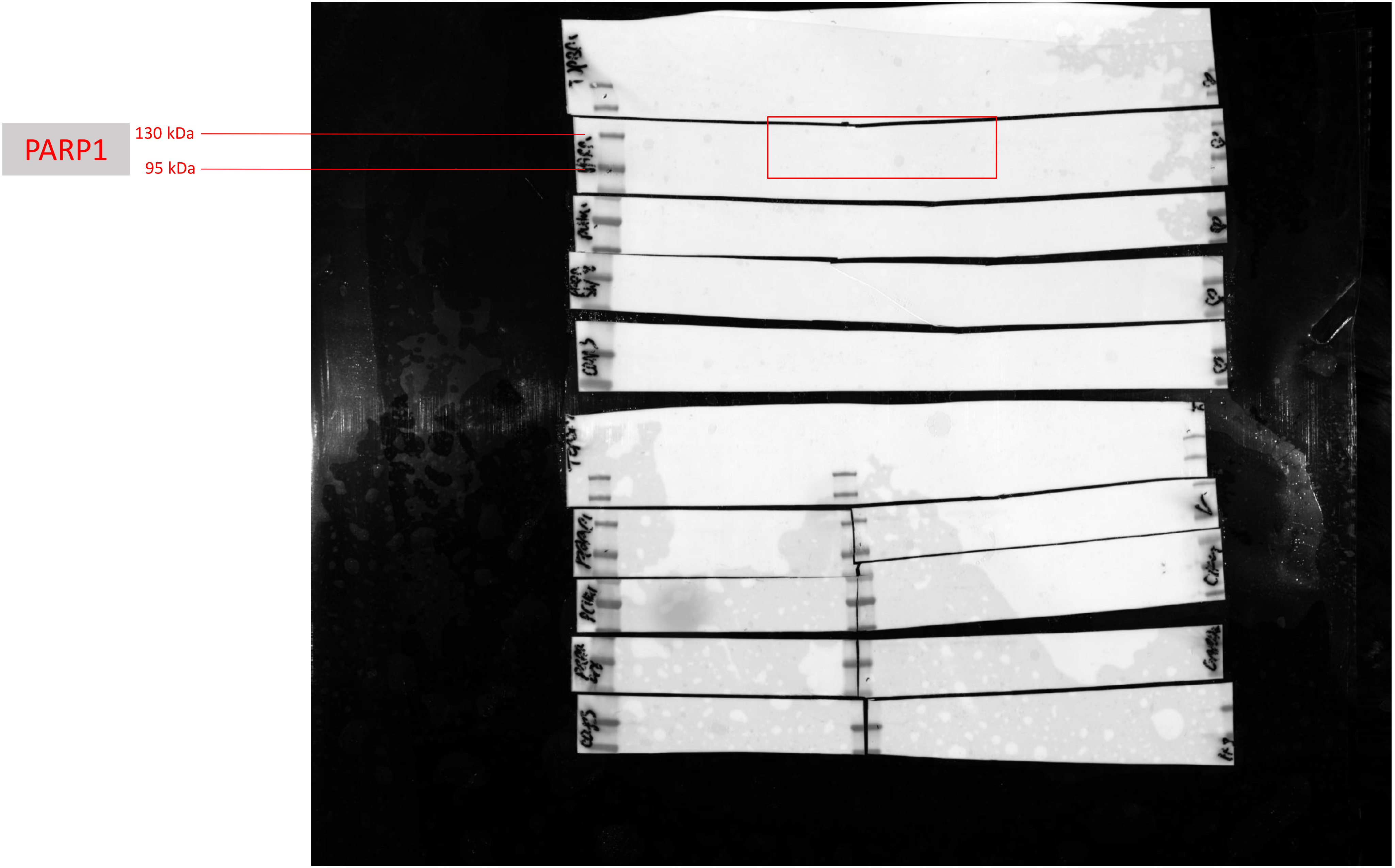

Supplement: Figure 4—source data 1. [file elife-106196-fig4-data1.zip › Fig 4C and D- Source Data 1/Fig 4C- Source Data 1.pdf]
