# Supplementary material for: TopBP1 biomolecular condensates as a new therapeutic target in advanced-stage colorectal cancer
Source: eLife. 2025 Oct 21;14:RP106196. doi: 10.7554/eLife.106196 (PMC12539802; doi:10.7554/eLife.106196)

Figure 4.D

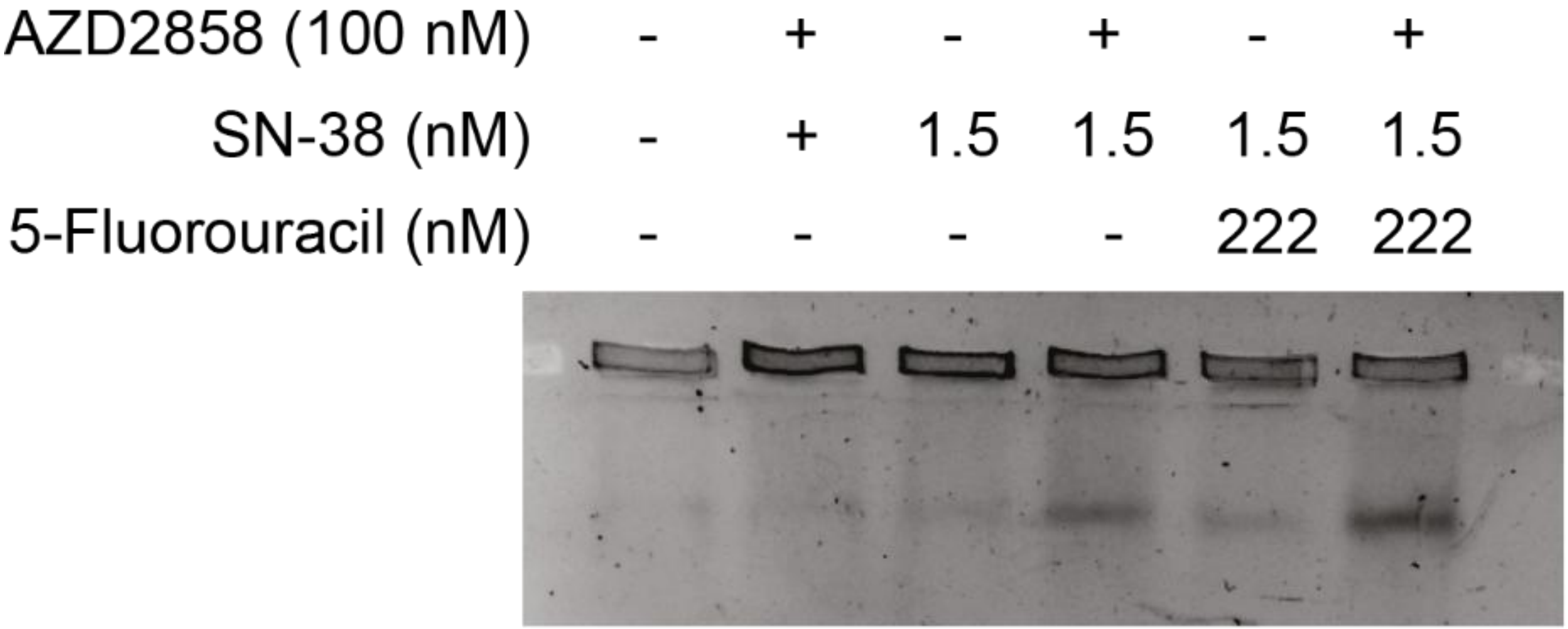

Figure 4D, Source Data 1. Below are the Original membranes corresponding to Figure 4D.
PFGE analysis of DNA damage in HCT116 cells incubated for 48 h as described in Figure 4A

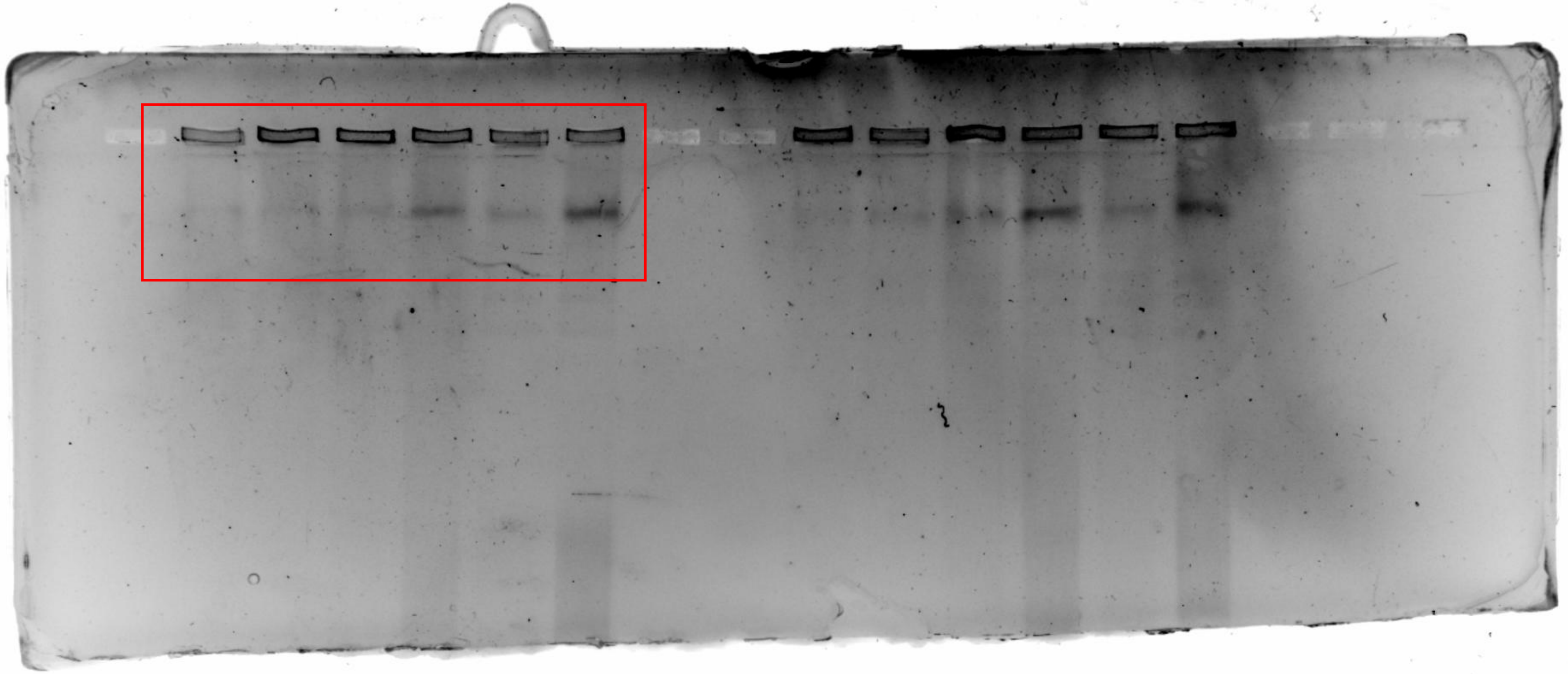

Supplement: Figure 4—source data 1. [file elife-106196-fig4-data1.zip › Fig 4C and D- Source Data 1/Fig 4D- Source Data 1.pdf]
