# Supplementary material for: TopBP1 biomolecular condensates as a new therapeutic target in advanced-stage colorectal cancer
Source: eLife. 2025 Oct 21;14:RP106196. doi: 10.7554/eLife.106196 (PMC12539802; doi:10.7554/eLife.106196)
Supplement: Supplementary file 1. — The specificity column indicates the IC50 values available in the literature corresponding to 50% of the maximal concentration needed to inhibit the GSK-3 target (except for GSK3i XIII, where only the inhibition constant, Ki, value was available). The GSK-3β specificity column indicates the specificity toward this isoform, if available. If specificity was determined, but the GSK-3β isoform was not clearly specified, a question mark is used to indicate this uncertainty (for VP3.15 dihydrobromide and AT 7519 hydrochloride salt). ++++: <1 nM; +++: between 1 and 10 nM; ++: between 10 and 40 nM; +: >40 nM. Molecules that inhibited light-induced optoTopBP1 foci in the present screen are highlighted in red, and the z-score is indicated. The SN-38-induced Chk1 phosphorylation (pChk1) inhibition column indicates whether the potential GSK-3 inhibitors from the initial screen inhibit SN-38-induced Chk1 phosphorylation at S345 in HCT116 cells. N/A: not available. [file elife-106196-supp1.pdf]

inhibitors of GSK3 in the bank	specificity	specificity to GSK-3β (IC50)	TopBP1 foci light induced inhibition	pChk1 SN-38 induced inhibition	source for IC50 data
**6-BIO**	5 nM (α/β)	+++	no		selleckchem
**9-ING-41**	N/A	N/A	no		selleckchem
**AR-A014418**	38 nM (β)	++	no		selleckchem
**AT 7519 hydrochloride salt**	89 nM (α/β?)	+ (?)	yes (-3.4)	N/A	raybiotech
**AT7519**	N/A	N/A	no		selleckchem
**AZD1080**	31 nM (β) 6.9 nM(α)	++	no		selleckchem
**AZD2858**	5 nM (β) 0,9 nM (α)	+++	yes (-3.6)	yes	medchemexpress
**Bikinin**	N/A	N/A	no		selleckchem
**BIO-acetoxime**	10 nM (α/β)	++	yes (-3.7)	no	selleckchem
**BRD0705**	515 nM (β)	+	No		selleckchem
**CHIR98014**	0.58 nM(β) 0.65 nM (α)	++++	yes (-2.8)	N/A	selleckchem
**CHIR99021**	6.7 nM (β) 10 nM (α)	+++	no		selleckchem
**CP21R7**	N/A	N/A	no		selleckchem
**Ginsenoside Rg2**	N/A	N/A	no		N/A
**GSK3i XIII**	24 nM (?) Ki	++	yes (-4.3)	N/A	sigma
**GSK-3β inhibitor 1**	4.19 nM	+++	no		selleckchem
**IM12**	53 nM (β)	+	no		selleckchem
**Indirubin**	600 nM (β)	+	no		selleckchem
**Indirubin-3'-monoxime**	190 nM	+	yes (-2.2)	N/A	tocris
**KenPaullone**	230 nM (β)	+	no		selleckchem
**KY19382**	10 nM(β)	++	no		medchemexpress+selleckchem
**Lithium citrate tribasic tetrahydrate**	N/A	N/A	no		N/A
**LY2090314**	0.9 nM (β) 1.5 nM (α)	++++	yes (-3.4)	no	selleckchem
**PHA767491 HCl**	N/A	N/A	no		selleckchem
**SB216763**	34.3 nM (α/β)	++	no		selleckchem
**SB415286**	78 nM (α/β)	+	no		selleckchem
**TBB**	N/A	N/A	no		N/A
**TDZD8**	200 nM (β)	+	no		selleckchem
**Tideglusib**	60 nM (β)	+	no		selleckchem
**TWS119**	30 nM (β)	++	yes (-2.8)	N/A	selleckchem
**VP3.15 dihydrobromide**	880 nM (α/β ?)	+ (?)	no		medchemexpress
