# Supplementary material for: TopBP1 biomolecular condensates as a new therapeutic target in advanced-stage colorectal cancer
Source: eLife. 2025 Oct 21;14:RP106196. doi: 10.7554/eLife.106196 (PMC12539802; doi:10.7554/eLife.106196)
Supplement: Supplementary file 2. — AZD2858: 100 nM. SN-38: 300 nM. [file elife-106196-supp2.pdf]

Supp File 2 (data from Figure 3A)

N1

2h Endpoint	G0/G1	S	G2/M
Non treated	51,25	34,24	13,65
AZD2858	50,42	34,94	13,71
SN38	52,94	36,38	9,8
AZD2858+SN38	52,19	35,36	11,39

N2

2h Endpoint	G0/G1	S	G2/M
Non treated	31,21	52,19	15,8
AZD2858	31,74	52,68	14,55
SN38	29,49	56,13	12,91
AZD2858+SN38	30,66	56,73	11,81

N3

2h Endpoint	G0/G1	S	G2/M
Non treated	40,81	36,04	22,25
AZD2858	40,42	37,87	20,64
SN38	38,84	31,44	27,08
AZD2858+SN38	39,13	38,3	21,28
