# Supplementary material for: TopBP1 biomolecular condensates as a new therapeutic target in advanced-stage colorectal cancer
Source: eLife. 2025 Oct 21;14:RP106196. doi: 10.7554/eLife.106196 (PMC12539802; doi:10.7554/eLife.106196)
Supplement: Supplementary file 3. — AZD2858: 100 nM. SN-38: 300 nM. [file elife-106196-supp3.pdf]

Figure 3C and Figure 3-figure supplement 1

N1

6h Endpoint	G0/G1	S BrdU-	G2/M	S
Non treated	26,73	0,66	12,54	59,61
AZD2858	24	0,82	10,72	63,98
SN38	41,7	12,19	17,93	27,12
AZD2858+SN38	38,39	3,14	13,71	44,02

12h Endpoint	G0/G1	S BrdU-	G2/M	S
Non treated	23,03	5,04	16,5	38,29
AZD2858	24,39	1,04	17,6	51,38
SN38	31,19	19,51	16,05	10,98
AZD2858+SN38	35,96	4,06	13,65	44,08

6h Pulse Chase	G0/G1	SE	G2.b	G2	S	N.G1
Non treated	41,41	23,12	2,66	17,46	9,38	5,24
AZD2858	39,82	23,1	1,96	14,72	10,13	9,62
SN38	44,99	0,63	19,13	8,2	24,56	1,49
AZD2858+SN38	42,52	1,93	17,65	12,16	23,94	0,25

12h Pulse Chase	G0/G1	SE	G2.b	G2	S	N.G1
Non treated	15,43	18,49	26,93	2,14	16,85	18,49
AZD2858	21,39	21,94	19,8	1,51	22,01	11,3
SN38	43,22	1,35	18,17	11,43	20,32	3,1
AZD2858+SN38	47,17	7,55	12,59	15,28	15,11	1,11

N2

6h Endpoint	G0/G1	S BrdU-	G2/M	S
Non treated	22,21	0,7	12,5	64,17
AZD2858	11,53	0,82	12,19	75,17
SN38	59,08	14,88	10,4	15,25
AZD2858+SN38	45,1	2,66	10,7	41,01

12h Endpoint	G0/G1	S BrdU-	G2/M	S
Non treated	30,87	0,8	19,05	48,92
AZD2858	31,65	0,99	18,29	48,74
SN38	59,5	9,55	9,95	20,24
AZD2858+SN38	53,73	2,93	9,88	32,79

6h Pulse Chase	G0/G1	SE	G2.b	G2	S	N.G1
Non treated	18,77	35,33	1,41	25,03	10,7	8,41
AZD2858	16,63	38,28	1,01	25,07	10,05	8,73
SN38	46,73	0,51	7,57	4,25	39,64	0,87
AZD2858+SN38	49,32	2,52	6,23	7,01	33,43	0,86

12h Pulse Chase	G0/G1	SE	G2.b	G2	S	N.G1
Non treated	12,13	26,94	22,42	2,41	14,36	21,09
AZD2858	13,02	15,95	28,57	2,48	23,19	16,34
SN38	58,87	2,61	5,42	4,2	26,14	1,59
AZD2858+SN38	47,98	13,04	5,55	10,97	21,11	0,5

N3

6h Endpoint	G0/G1	S BrdU-	G2/M	S
Non treated	24,21	0,59	14,64	60,03
AZD2858	22,85	0,6	14,02	61,97
SN38	54,22	18,78	13,08	13,36
AZD2858+SN38	40,45	2,53	10,43	45,72

12h Endpoint	G0/G1	S BrdU-	G2/M	S
Non treated	26,13	0,61	17,01	54,7
AZD2858	27,47	0,54	13,65	57,1
SN38	54,9	15,27	12,37	12,11
AZD2858+SN38	48,86	3,3	7,17	38,84

6h Pulse Chase	G0/G1	SE	G2.b	G2	S	N.G1
Non treated	27,67	31,74	1,26	14,37	17,75	6,84
AZD2858	24,79	33,13	0,88	16,57	15,52	8,69
SN38	45,67	2,38	8,6	4,79	37,39	0,87
AZD2858+SN38	45,77	4,58	7,66	7,11	34,1	0,25

12h Pulse Chase	G0/G1	SE	G2.b	G2	S	N.G1
Non treated	14,04	13,25	16,65	1,99	22,64	30,91
AZD2858	19,36	11,6	16,19	2,13	27,62	22,3
SN38	49,88	1,81	7,52	5,41	31,15	2,94
AZD2858+SN38	51,79	5,64	5,64	8,74	25,98	1,03
