# Supplementary material for: TopBP1 biomolecular condensates as a new therapeutic target in advanced-stage colorectal cancer
Source: eLife. 2025 Oct 21;14:RP106196. doi: 10.7554/eLife.106196 (PMC12539802; doi:10.7554/eLife.106196)
Supplement: Supplementary file 4. — NT: non-treated. A: AZD2858. S: SN-38. F: FOLFIRI (5-FU+SN-38). The p-values of interest from Figure 4B are indicated in red. [file elife-106196-supp4.pdf]

Estimation of effect (amplitude and *p*-value) by linear modeling:

Effect of experimental condition (compared to "NT") :

Condition	Effect on percentage of subG1	*p*-value
A	-0.05329	0.99220
AF	+56.43905	9.19e-07
AS	+46.09911	5.79e-06
F	+17.95105	0.00706
S	+9.60998	0.10089

Effect of experimental condition (compared to "S") :

Condition	Effect on percentage of subG1	*p*-value
NT	-9.610	0.10089
A	-9.663	0.09926
AF	+46.829	5.03e-06
AS	+36.489	4.40e-05
F	+8.341	0.14786

Effect of experimental condition (compared to "F") :

Condition	Effect on percentage of subG1	*p*-value
S	-8.341	0.147855
NT	-17.951	0.007057
A	-18.004	0.006939
AF	+38.488	2.8e-05
AS	+28.148	0.000351

Effect of replicate identity (compared to replicate #1) :

Replicate	Effect on percentage of subG1	*p*-value
2	+12.45762	0.00784
3	+11.50860	0.01204
