# Supplementary material for: TopBP1 biomolecular condensates as a new therapeutic target in advanced-stage colorectal cancer
Source: eLife. 2025 Oct 21;14:RP106196. doi: 10.7554/eLife.106196 (PMC12539802; doi:10.7554/eLife.106196)
Supplement: Supplementary file 5. — All the dilutions are based on the dilution 1/1 corresponding to 12 µM 5-fluorouracil (5-FU) and 100 nM SN-38. [file elife-106196-supp5.pdf]

FOLFIRI concentration range<br>(dilution 1/1 = 12 µM 5-FU + 100 nM SN-38)						
HCT116 (2D & 3D)	**FOLFIRI (dilution factor)**	**1296**	**648**	**324**	**162**	**81**
	5-FU (µM)	0,009	0,019	0,037	0,074	0,148
	SN-38 (nM)	0,077	0,154	0,309	0,617	1,235
CT26 (2D & 3D)	**FOLFIRI (dilution factor)**	**144**	**72**	**36**	**18**	**9**
	5-FU (µM)	0,083	0,167	0,333	0,667	1,333
	SN-38 (nM)	0,694	1,389	2,778	5,556	11,111
HT29 (2D)	**FOLFIRI (dilution factor)**	**432**	**216**	**108**	**54**	**27**
	5-FU (µM)	0,028	0,056	0,111	0,222	0,444
	SN-38 (nM)	0,231	0,463	0,926	1,852	3,704
SW620 (2D)	**FOLFIRI (dilution factor)**	**864**	**432**	**216**	**108**	**54**
	5-FU (µM)	0,014	0,028	0,056	0,111	0,222
	SN-38 (nM)	0,116	0,231	0,463	0,926	1,852
SW480 (2D)	**FOLFIRI (dilution factor)**	**864**	**432**	**216**	**108**	**54**
	5-FU (µM)	0,014	0,028	0,056	0,111	0,222
	SN-38 (nM)	0,116	0,231	0,463	0,926	1,852
HCT116-SN6 (2D)<br>HCT116-SN50 (2D)	**FOLFIRI (dilution factor)**	**32**	**16**	**8**	**4**	**2**
	5-FU (µM)	0,375	0,750	1,500	3,000	6,000
	SN-38 (nM)	3,125	6,250	12,500	25,000	50,000
HCT116-SN6 (3D)	**FOLFIRI (dilution factor)**	**192**	**96**	**48**	**24**	**12**
	5-FU (µM)	0,063	0,125	0,250	0,500	1,000
	SN-38 (nM)	0,521	1,042	2,083	4,167	8,333
HCT116-SN50 (3D)	**FOLFIRI (dilution factor)**	**48**	**24**	**12**	**6**	**3**
	5-FU (µM)	0,250	0,500	1,000	2,000	4,000
	SN-38 (nM)	2,083	4,167	8,333	16,667	33,333

Table S5
