# Supplementary material for: TopBP1 biomolecular condensates as a new therapeutic target in advanced-stage colorectal cancer
Source: eLife. 2025 Oct 21;14:RP106196. doi: 10.7554/eLife.106196 (PMC12539802; doi:10.7554/eLife.106196)
Supplement: Supplementary file 6. — WB, western blotting, IF, immunofluorescence analyses. [file elife-106196-supp6.pdf]

Target	Compagny	Reference	Species	Dilution Celigo	Dilution WB	Dilution IF
H3	Cell Signaling Technology	#4499	Rabbit		1/1000	
α-Tubulin	Sigma	#T5168	Mouse		1/10000	
53BP1	Abcam	#ab175933	Rabbit			1/100
ATR	Bethyl	#A300-137A	Rabbit		1/1000	
Chk1	Santa Cruz Biotechnology	#sc-8408	Mouse		1/1000	
Chk2	Milipore	#05-649	Mouse		1/1000	
Cleaved caspase-3	Cell Signaling Technology	#9661	Rabbit		1/1000	
GSK-3β	Cell Signaling Technology	#9832	Mouse		1/1000	
GSK-3β (S9)	Cell Signaling Technology	#9336	Rabbit		1/1000	
H2B	Abcam	#ab1790	Rabbit		1/2500	
pATM (T1981)	Santa Cruz Biotechnology	#sc-47739	Mouse	1/100		
pATM (T1981)	Rockland	#200-301-40	Mouse		1/1000	
pChk1 (S345)	Cell Signaling Technology	#2348	Rabbit	1/50	1/1000	
pChk2 (T68)	Cell signaling Technology	#2661	Rabbit	1/50	1/1000	
PARP1	Santa Cruz Biotechnology	#sc-8007	Mouse		1/1000	
PML	Santa Cruz Biotechnology	#sc-966	Mouse			1/100
Pol ε	Kind gift from Shou Waga	#N/A	Rabbit		1/1000	
pRPA32 (S33)	Abcam	#ab2118877	Rabbit		1/1000	
RPA32	Abcam	#ab2175	Mouse		1/1000	
RPA34	Kind gift from Marcel Méchali		Rabbit		1/1000	
TopBP1	Santa Cruz	#sc-271043	Mouse			1/100
TopBP1	Euromedex	#A300-111A	Rabbit		1/1000	
TopBP1	Kind gift from Larry Karnitz		Rabbit		1/1000	
Vinculin	Merck	#V9131	Mouse		1/10000	
γH2AX (S139)	Cell Signaling Technology	#9718S	Rabbit		1/1000	
γH2AX (S139)	Abcam	#ab1195189	Rabbit	1/1000		
Goat anti-rabbit IgG (H+L) AF568	Thermo Fisher Scientific	#A11011	Secondary		1/5000	
Goat anti-mouse IgG (H+L) AF488	Thermo Fisher Scientific	#A11029	Secondary		1/5000	
